# Substrate-controlled C–H or C–C alkynylation of cyclopropanes: generation of aryl radical cations by direct light activation of hypervalent iodine reagents†

**DOI:** 10.1039/d2sc04344k

**Published:** 2022-10-12

**Authors:** Tin V. T. Nguyen, Matthew D. Wodrich, Jerome Waser

**Affiliations:** Laboratory of Catalysis and Organic Synthesis, Institute of Chemistry and Chemical Engineering, Ecole Polytechnique Fédérale de Lausanne Ch-1015 Lausanne Switzerland Jerome.waser@epfl.ch

## Abstract

We report the first oxidative C–H alkynylation of arylcyclopropanes. Irradiation of ethynylbenziodoxolone (EBX) reagents with visible light at 440 nm promoted the reaction. By the choice of the aryl group on the cyclopropane, it was possible to completely switch the outcome of the reaction from the alkynylation of the C–H bond to the oxyalkynylation of the C–C bond, which proceeded without the need for a catalyst, in contrast to previous works. The oxyalkynylation could also be extended to aminocyclopropanes as well as styrenes. Computations indicated that the C–H activation became a favoured nearly barrierless process in the presence of two *ortho* methyl groups on the benzene ring.

## Abstract 2

C–C or C–H alkynylation of aryl cyclopropanes was realized by direct activation of EBX reagents with visible light. With *ortho* methyl groups on the benzene, C–H functionalization became favoured.

## Graphical abstract

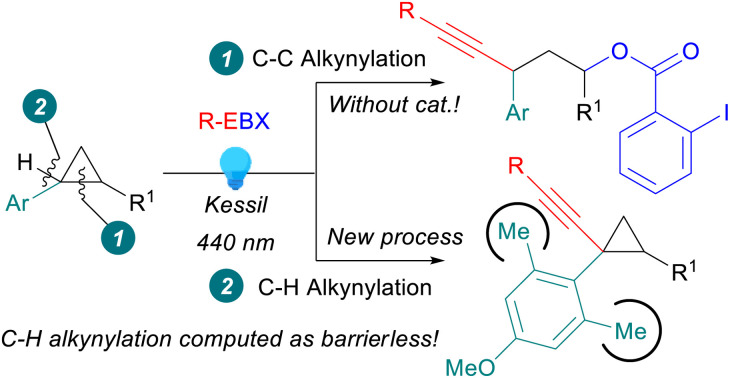

## Introduction

**Scheme 1:**
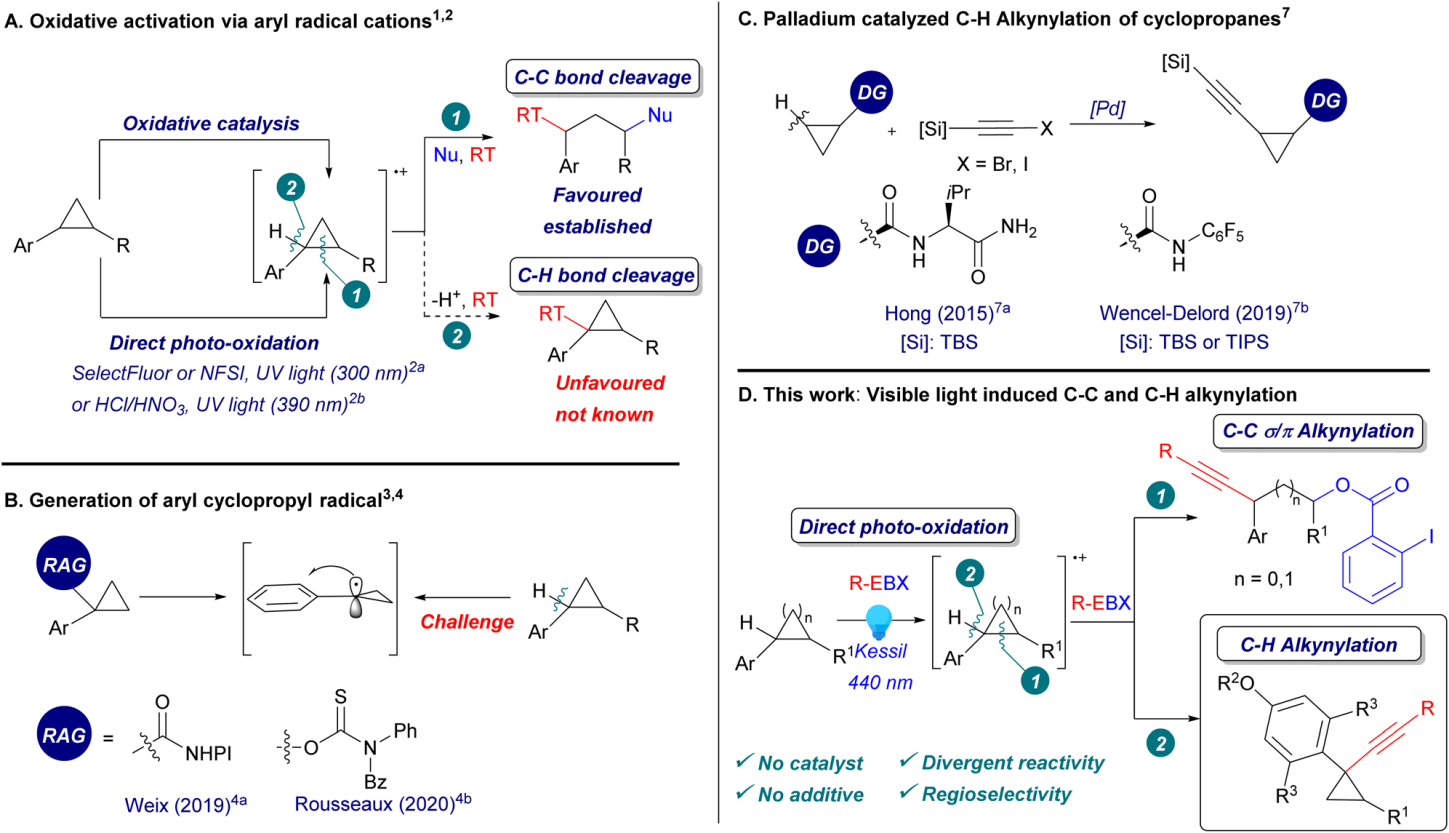
(A) Oxidative activation *via* aryl radical cations. (B) Generation of aryl cyclopropyl radicals. (C) Palladium catalysed C–H alkynylation of cyclopropanes. (D) This work. Nu: nucleophiles. RT: radical trap. RAG: redox active group. DG: directing group.

Cyclopropanes play a key role in synthetic chemistry, both as structural elements and reactive building blocks. Recently, the functionalization of arylcyclopropanes through single electron oxidation has been the focus of intensive research (Scheme 1A, eqn (1)).^1^ The formation of a reactive radical cation can be achieved using either a copper catalyst with a strong oxidant^1*d*–*f*^ or a photoredox metal catalyst.^1*g*–*j*^ In addition, there are two examples of the direct light-mediated oxidation of arylcyclopropanes reported with nitrogen-based and chloride radicals using high energy UV light irradiation or a strong acid solution.^2^ However, all these methods result in ring-opening reactions promoted by the release of ring strain.

In contrast to C–C activation, C–H bond functionalization of arylcyclopropanes *via* radical cations has not been reported yet (Scheme 1A, eqn (2)). Although aryl cyclopropyl radicals are known to be more stable than alkyl-substituted cyclopropyl radicals due to the conjugation with the aromatic system,^3^ a direct method to generate them from the corresponding arylcyclopropanes *via* C–H bond cleavage has never been reported before. Instead, redox active leaving groups were often used to generate aryl cyclopropyl radicals, as reported by Weix^4*a*^ and Rousseaux^4*b*^ (Scheme 1B). In order to perform a direct C–H functionalization on cyclopropanes, palladium catalysis with fine-tuned directing groups has been most successful.^5*a*,*b*^ However, the regioselectivity could not be controlled when several benzylic C–H bonds were present in *ortho* position.^5*c*^ In particular, 1,1-aryl-alkynyl cyclopropanes are useful building blocks in synthetic and medicinal chemistry.^6^ They have never been accessed *via* C–H alkynylation, which has been realized mostly on unsubstituted positions using directing group mediated transition metal catalysis (Scheme 1C).^7^ Therefore, the development of a selective C–H functionalization of arylcyclopropanes under mild conditions would complement significantly existing methodologies.

Hypervalent iodine reagents are now well-established for the alkynylation of nucleophiles,^8^ and more recently, of radicals,^9–12^ enabling the synthesis of alkynes otherwise difficult to access. Efficient access toward structurally diverse alkynes is urgently needed, due to their numerous applications in synthetic and medicinal chemistry, chemical biology and organic materials.^13^ Our group recently discovered the direct visible light activation of aryl-substituted ethynylbenziodoxolones (ArEBX) reagents.^14^ The generated excited species could be used for the oxidative alkynylation of several functional groups and alkenes. A key advantage of this approach is the simplicity of the procedure, requiring only the irradiation of a mixture of substrates and EBX reagents without the need for fine-tuned photocatalysts and additives. We wondered therefore if this approach would be also successful in the case of alkynylation of arylcyclopropanes.

Herein, we report the first C–H alkynylation of arylcyclopropanes through the direct photoexcitation of EBX reagents (Scheme 1D). The alkynylated cyclopropanes products were obtained with full regioselectivity when several benzylic C–H bonds were present. In addition, we show that the reaction outcome could be changed from C–H to C–C alkynylation in dependence of the aryl group on the cyclopropane. The 1,3-oxyalkynylation products were also obtained with full regioselectivity, in yields comparable to those of photocatalytic methods.^1*j*^ The same conditions were also used for the 1,3-oxyalkynylation of aminocyclopropanes and the 1,2-oxyalkynylation of styrenes.

## Results and discussion

To start our studies, we choose *para*-methoxybenzene substituted cyclopropane 1a, as it can be oxidized at a relatively low potential (*E*_1/2_ = +1.35 V).^2*b*^ As we had estimated the oxidation potential of photoexcited Ph-EBX* (2a*) to be +1.8 V,^14^ the generation of a radical cation should be possible. Indeed, efficient oxyalkynylation to give 3a was observed (Table 1). The best results were obtained using chloroform as solvent, 2.5 equivalents of 2a and two Kessil lamps for irradiation. Under these conditions, 3a was isolated in 68% yield (entry 1). The only observed side product was double addition of 2-iodobenzoate in 10% yield, probably resulting from over-oxidation of the benzylic radical to the cation. During completion of our work on the oxyalkynylation,^15^ Zuo and Studer reported that a photocatalyst and BIOAc as additive are needed when using blue LED strips to obtain 3a in 67% isolated yield.^1*j*^ A lower yield was obtained with two equivalents 2a, and using three equivalents did not improve the yield (entries 3 and 4). This agrees with what we observed in our previous work,^14^ indicating that one equivalent of 2a is probably acting as oxidant, and a second one as alkynylation reagent. In contrast to Zuo and Studer's work, no additive was needed in the reaction and adding 50% of BI-OAC gave no improvement (entry 5). No product was obtained when heating the reaction mixture at 50 °C in the dark (entry 6).

**Table 1:** Optimization of the oxyalkynylation and C–H alkynylation^a^

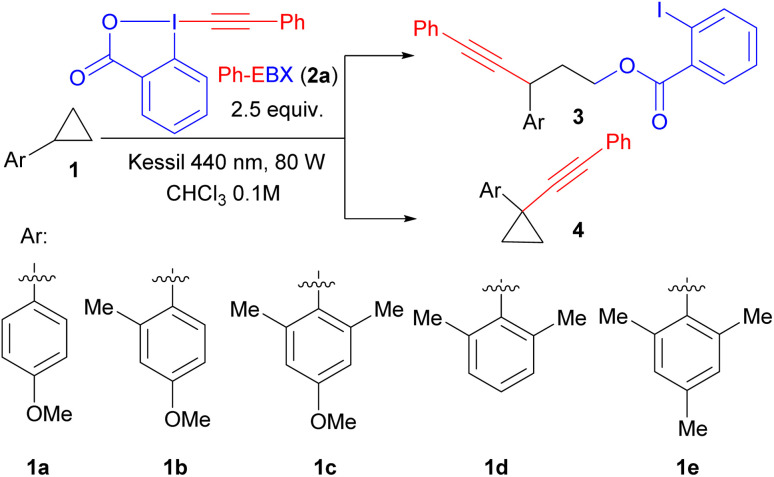				
Entry	Substrate	Deviation from conditions	Yield % (3)^b^	Yield % (4)^b^
1	1a	None	72 (68)	—
2	1a	In CH_2_Cl_2_/DCE	54/20	—
3	1a	2 equiv. 2a	58	—
4	1a	3 equiv. 2a	73	—
5	1a	0.5 equiv. BI-OAc, 2 equiv. 2a	52	—
6	1a	In the dark at 50 °C	<5	—
7	1b	None	(61)	—
8	1c	None	Trace	85
9	1d	None	—	—
10	1e	None	—	<10%
11	1c	1.5 equiv. 2a	Trace	83 (83)
12	1c	1 equiv./2 equiv. 2a	Trace	63/84
13	1c	1.5 equiv. 2a, DCM	—	74
14	1c	1.5 equiv. 2a, MeOH/DMF/CH_3_CN	—	<65%

a Reaction conditions: 0.2 mmol 1 (1 equiv.), 0.5 mmol 2a (2.5 equiv.), two Kessil lamps (440 nm, 2 × 40 W), in 2 mL CHCl_3_.

b ^1^H NMR yield and conversion were determined with CH_2_Br_2_ as an internal standard. Isolated yield after chromatography is given in brackets.

While expanding the scope of substrates for oxyalkynylation, we observed a complete switch of reactivity from C–C alkynylated product to C–H alkynylation. Particularly, while only the oxyalkynylation product was isolated in 61% yield with substrate 1b having one *ortho* methyl group (entry 7), substrate 1c bearing two *ortho* methyl groups gave selectively C–H alkynylation product 4a in 85% yield and only trace amount of ring-opening product was observed (entry 8). The *para* methoxy group is important for the reaction to happen as no conversion was observed in the case of substrate 1d and less than 10% NMR yield of C–H alkynylation product was observed with trimethyl substituted cyclopropane 1e (entries 9 and 10). It is worth mentioning that only 1.5 equivalents of 2a was required to obtain 83% of product 4a (entry 11), indicating a more efficient reaction compared to the C–C oxyalkynylation. A lower yield was obtained with one equivalent 2a, and using two equivalents did not improve the yield (entry 12). Further screening of solvents showed that chloroform is the optimal choice (entries 13 and 14).

**Scheme 2:**
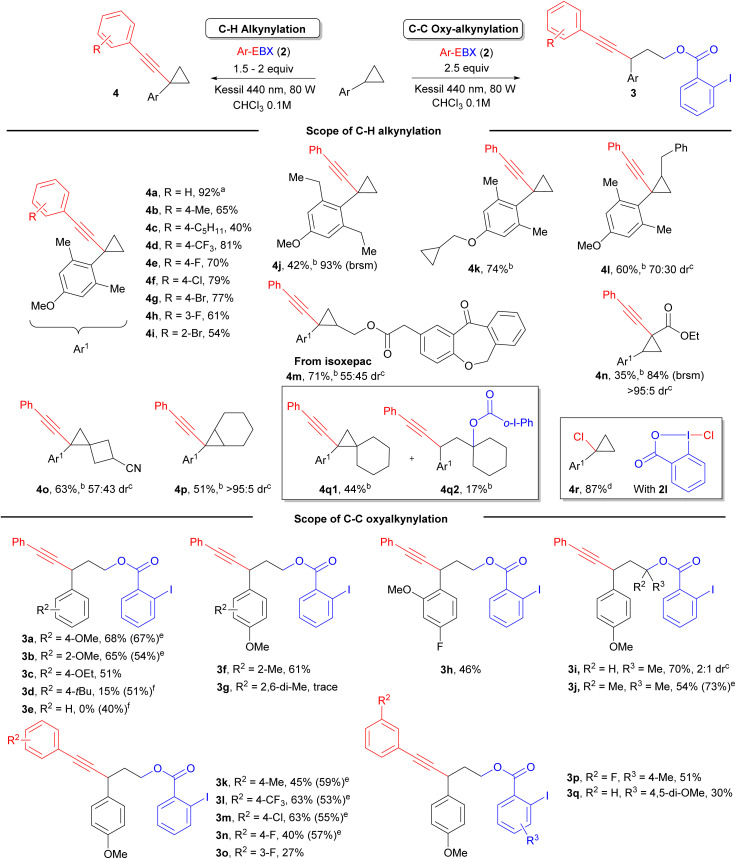
Scope of reaction. Isolated yield after column chromatography is given. ^a^ Yield reported on 2 mmol scale. ^b^ 2 equiv. of Ph-EBX was used. ^c^ The stereochemistry of the major stereoisomer could not be unequivocally assigned. ^d^ 1.5 equiv. of Cl-BX reagent under Kessil lamp 427 nm. ^e^ Yield from ref. ^1*j*^ using 4-CzIPN as photocatalyst and BI-OAc as additive. ^f^Yield using Ph-Acr-MesBF_4_ as photocatalyst, 2 equiv. PhEBX, Kessil lamp 467 nm. brsm: based on recovered starting material. *o*-I-Ph: *ortho*-iodo phenyl.

With optimized conditions in hand, we first studied the scope of the C–H alkynylation of arylcyclopropanes (Scheme 2). We were pleased to see that Ph-EBX (2a) mediated alkynylation of 1c was efficient and easily scalable: product 4a was obtained in 92% on a 2 mmol scale. The transformation was also successful with functionalized EBX reagents, considering that only aryl-substituted EBXs are photoactive.^14^ Control experiments showed that no product can be obtained with alkyl- or silyl-substituted EBX reagents, with the exception of the cyclopropylalkynyl product, which was obtained in 19% yield (see ESI† for details). Alkyl substituents in *para* position on the benzene ring gave products 4b and 4c in 65% and 40% yield. A trifluoromethyl group and halogens were well tolerated at this position, giving products 4d–g in 70–81% yield. Finally, products 4h and 4i, bearing a *meta* fluoro and an *ortho* bromo group respectively, were obtained in 61% and 54% yield. These results contrasted with our previous work were only Ph- and tolyl-EBX gave useful yields of products.^14^ Introduction of two *ortho* ethyl groups or an unactivated cyclopropylmethyl ether group on the aryl ring led to 4j and 4k with 42% and 74% yield, respectively. For benzyl substituted substrate 4l, the product was obtained with 60% yield with complete regioselective alkynylation of the cyclopropane ring. Product 4m derived from Isoxepac, a non-steroidal anti-inflammatory drug, was obtained in 71% yield. It is worth mentioning that the reaction was tolerant to several other acidic or benzylic C–H bonds present in the structure of Isoxepac, indicating that the transformation is highly selective towards arylcyclopropanes. The reaction also works with donor–acceptor cyclopropane 1k, but the alkynylation happened on the carbon adjacent to the ester group giving product 4n. The transformation was also successful with trisubstituted bridged or spiro structures, giving product 4o and 4p in 63% and 51% yield, respectively. However, starting from substrate 1i bearing a spiro[2,5]octane, both C–H and C–C alkynylation products were observed, yielding 4q1 and 4q2 in 44% and 17% yield, respectively. When treating substrate 1c with reagent 2l under irradiation with a Kessil lamp at 427 nm, C–H chlorination product 4r was obtained in 87% yield. 2l is known as a precursor of chlorine radicals under irradiation,^16*a*^ but other benzylic C–H bonds on 1c were still tolerated, confirming the high regioselectivity of the transformation towards the cyclopropane C–H bond.

We then further investigated the scope of the 1,3 oxyalkynylation under catalyst/additive free conditions. As expected, electron-rich substituents were required to promote the reaction: oxyalkynylation products 3a–c bearing a methoxy or an ethoxy substituent were obtained in 51–68% yield, whereas a *tert*-butyl substituent gave 3d in only 15% yield and no product was obtained for an unsubstituted benzene ring 3e. For 3a and 3b, the yields obtained were comparable or slightly superior to the photocatalytic method developed by Zuo and Studer.^1*j*^ However, the photocatalyst approach is superior for less electron-rich substrates not accessible *via* direct photoexcitation of EBXs: Products 3d and 3e where obtained in 51% and 40% respectively using an acridinium dye as photocatalyst. A methyl group in *ortho* position was well tolerated to give product 3f. In the presence of a methoxy group, an electron-deficient fluoro substituent was tolerated to give product 3h in 46% yield. Starting from β-substituted cyclopropanes, products 3i and 3j were obtained in 70% and 54% yield with selective attack of the iodobenzoate at the most encumbered position. We then examined the scope of EBX reagents. A tolyl-substituted EBX gave 3k in 45% yield. Introduction of a *para*-trifluoromethyl or a chloro group on the aryl ring of the alkyne led to 3l and 3m in 63% yield. Fluoro substituents were also tolerated, but gave only moderate yields of 3n and 3o. The yield of transfer of the 3-F-benzene substituted alkyne could be increased from 27% to 51% by introducing a methyl group *para* to the carboxylic acid (product 3p). A dimethoxy-substituted reagent could also be used to give 3q in 30% yield.

**Scheme 3:**
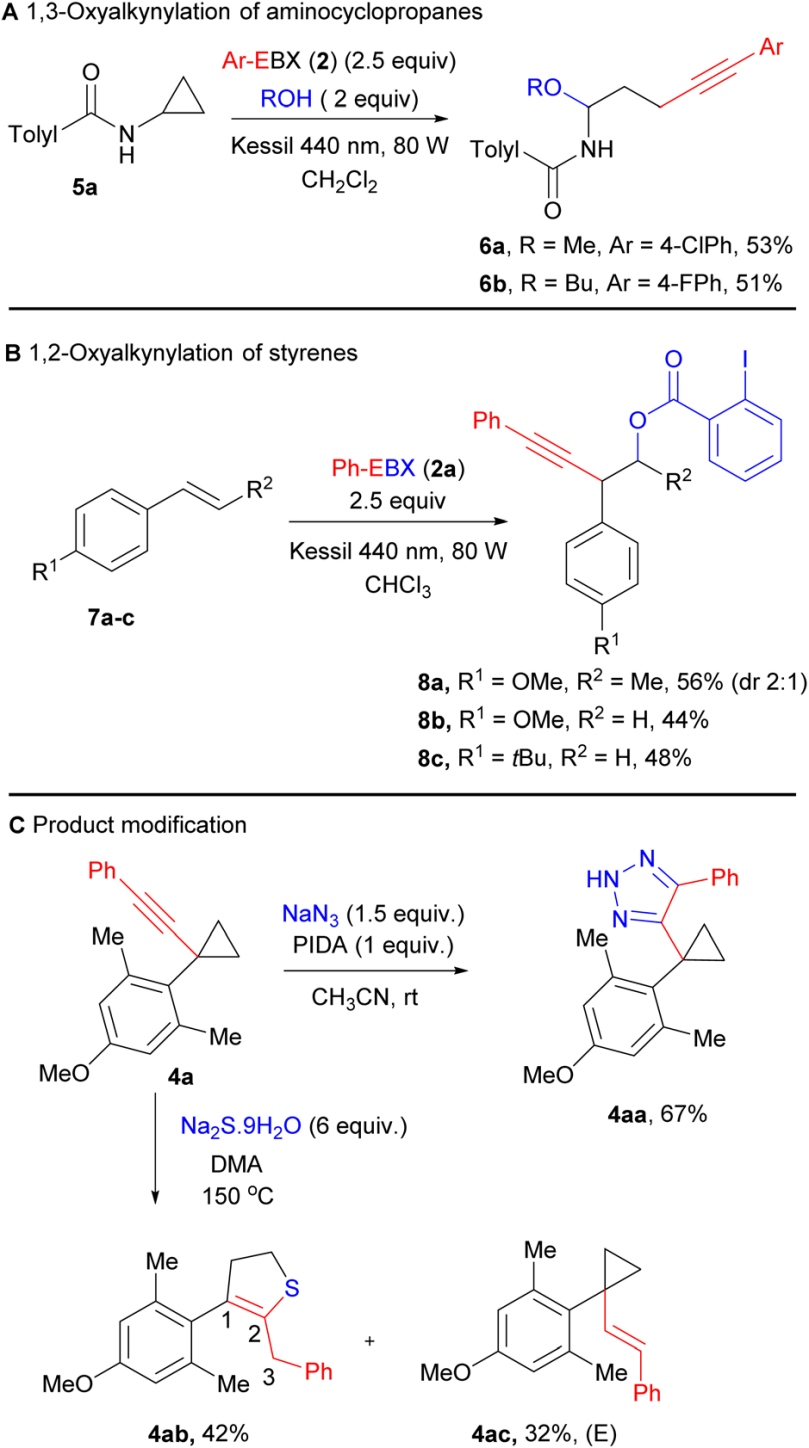
(A) 1,3-Oxyalkynylation of aminocyclopropanes, (B) 1,2-oxyalkynylation of styrene derivatives. (C) Product modification of substrate 4a.

The same conditions can be also applied to the oxyalkynylation of aminocyclopropane 5a giving products 6a and 6b in 53% and 51% yield (Scheme 3A). As reported by Chen for the photocatalytic reaction,^16*b*^ a complete inversion of the regioselectivity can be observed in this case. This is probably due to the lower stability of the radical cation, leading to ring-opening prior to attack of the nucleophile.

The oxyalkynylation is not limited to σ–C–C bonds. Indeed, electron-rich styrene derivatives 7a–c could be converted to products 8a–c in 44–56% yield with complete regioselectivity *via* 1,2-oxyalkynylation of the π bond (Scheme 3B). In this case, the same regioselectivity is observed as for enamides.^12*b*^

As the synthetic utility of the oxyalkynylation products had been already demonstrated by Studer and Chen,^1*j*,16*b*^ we focused on functionalization of product 4a containing the alkynyl cyclopropane motif. We carried out first an oxidative cycloaddition reaction of 4a with sodium azide, producing 1,2,3-triazole product 4aa in 67% yield.^17^ The cyclopropane ring remains intact under these oxidative conditions. The cyclopropane ring could also be expanded to form 2,3-dihydrothiophene derivative 4ab in the presence of sodium sulfide.^18*a*^ The position of the C=C double bond was shifted from C2–C3 to C1–C2. This product could potentially undergo oxidation to form highly functionalized thiophene derivatives.^18*b*^ In addition, we also isolated vinyl cyclopropane 4ac as a by-product with full *E* stereoselectivity, resulting most probably from reduction of the alkyne *via* a one-electron pathway.

**Scheme 4:**
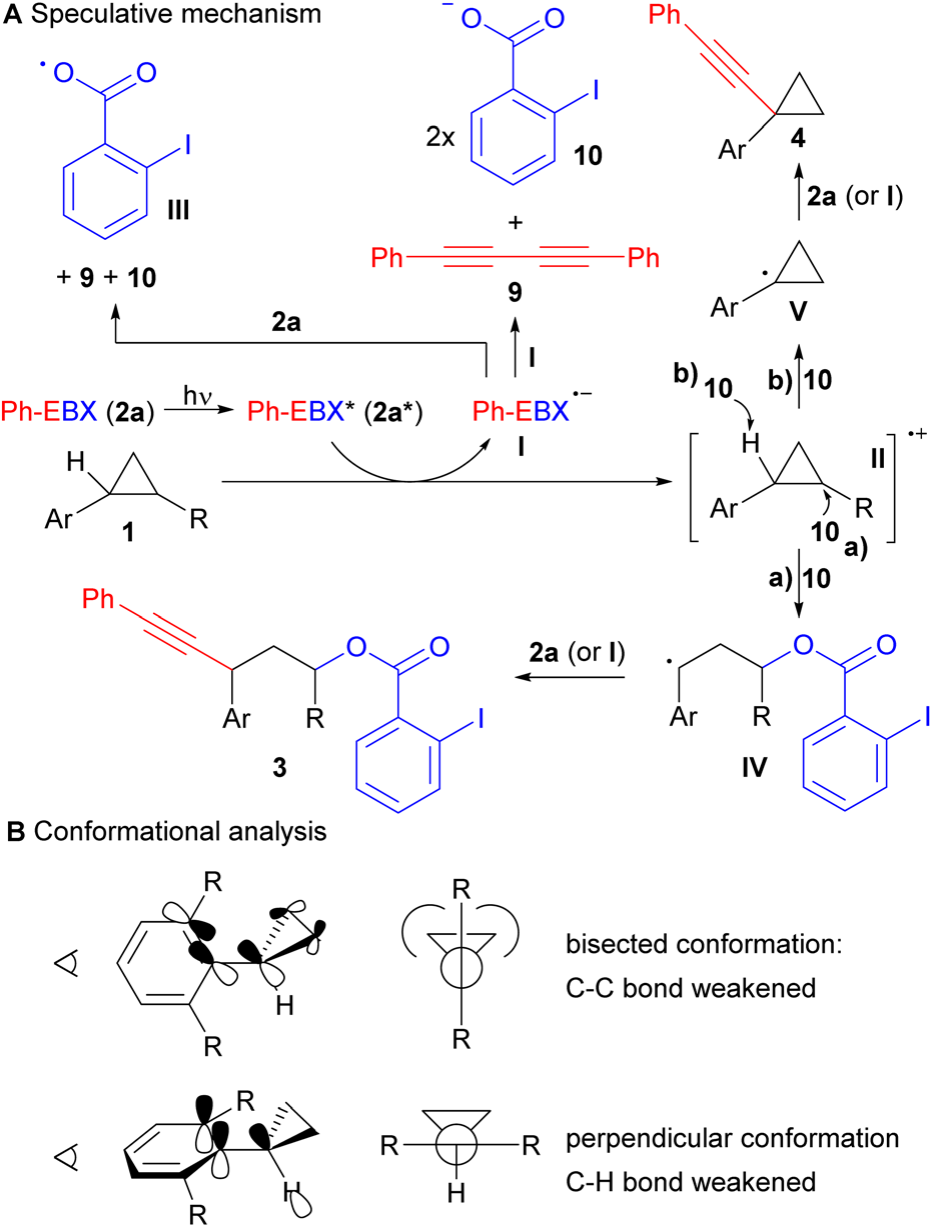
Speculative mechanism for the C–C oxyalkynylation and the C–H alkynylation reactions (A) and conformational analysis of arylcyclopropanes (B).

Based on our experimental results and our previous studies, a highly speculative reaction mechanism can be proposed (Scheme 4A).^14^ Direct light activation of Ph-EBX (2a) would lead to the highly oxidizing species 2a*. Single electron transfer from arylcyclopropane 1 would give then radical anion I and radical cation II. In a previous work, we showed by computation that I was relatively stable to monomolecular decomposition.^19^ The major side product observed in the photoactivation of 2a is diyne 9, probably formed in either of two bimolecular pathway: Reaction with another molecule of I to give 9 and 2 equivalents of 2-iodobenzoate (10), or with 2a to give 9, 10 and radical III.

From radical cation II, two pathways can be considered. Ring opening with benzoate 10 to give the more stable radical IV, followed by reaction with 2a (or I) would lead to the oxyalkynylation product 3 (pathway a). Deprotonation with benzoate 10 would give radical V (pathway b). A support for the proposed deprotonation pathway can be found in the switch of regioselectivity observed in the formation of product 4n: cleavage of the more acidic α C–H ester bond was obtained rather than the C–H benzylic bond. This result also suggested a fully delocalized electron system for intermediate II. Radical V would be then alkynylated by either 2a or I resulting in the formation of 4. In general, we would favour alkynylation with 2a, as it would be present in higher concentration, but an involvement of I cannot be excluded, especially in the case of C–H alkynylation, which is surprisingly efficient. For C–H alkynylation, a radical chain mechanism involving radical III should also be considered. In fact, H abstraction from 1 by III would give directly radical V. However, we think that this mechanism may be less probable, as no alkynylation was observed with arylcyclopropanes lacking the methoxy group such as 1d, showing that oxidation of the substrate is probably needed for the reaction to occur. Furthermore, direct HAT on cyclopropanes is generally difficult, due to the stronger C–H bond/lower stability of the radical,^20^ and the 2-iodobenzoyloxy radical (III) is stabilized by a I–O interaction, making it less prone to HAT.^21^

A striking result of our studies was the complete switch of chemoselectivity when introducing a second *ortho* methyl group on the arene ring. A first tentative explanation may be found in the conformation analysis of the cyclopropane (Scheme 4B). It is known that arylcyclopropanes favour a bisected conformation to enable overlap between the π-Walsh orbitals and the π* of the benzene ring.^22^ Another effect of this interaction is also the weakening of the C–C bond that favour ring-opening. With one *ortho* group, a low energy bisected conformer is still available. However, when a second *ortho* group is present, strong steric interactions with the cyclopropane cannot be avoided anymore. Therefore, the usually less favoured perpendicular conformation becomes lower in energy. In this conformation, there is nearly no effect of the benzene ring on the strength of the C–C bonds. In contrast, one may envision an interaction between the π system of the benzene and the σ* orbital of the C–H bond, favouring deprotonation/H abstraction.

Of course, this analysis of the conformation of the neutral arylcyclopropanes may be misleading. Therefore, we turned to computation to analyse the radical cation conformations (see ESI Fig. S1†). The only minima on the potential energy surface for 1a correspond to (two) bisected conformations that are separated by a perpendicular transition state lying ∼7 kcal mol^−1^ above the lower energy bisected conformations. In contrast in 1c the perpendicular (as well as the two bisected conformations) are minima and are easily interconvertible with a rotational barrier of less than 1 kcal mol^−1^. While the bisected conformations (in both 1a and 1c) favour C–C activation through weakening of the cyclopropane C–C bond *via* donation of electron density from the aromatic ring to the C–C σ* orbital, the existence of an easily accessible perpendicular conformation in 1c analogously facilitates C–H activation through a negative hyperconjugative interaction in which electron density is donated from the aromatic ring π-system to the (now) well-aligned C–H σ* orbital of the cyclopropane moiety. Indeed, the presence of this interaction can easily be seen as a lengthening of the C–H bond lengths of 1c in the perpendicular (1.096 Å) relative to the bisected (1.085 Å) conformation. Note that 1a is unlikely to adopt this conformation, as it represents a transition state structure between the two roughly isoenergetic bisected conformations.

**Fig. 1:**
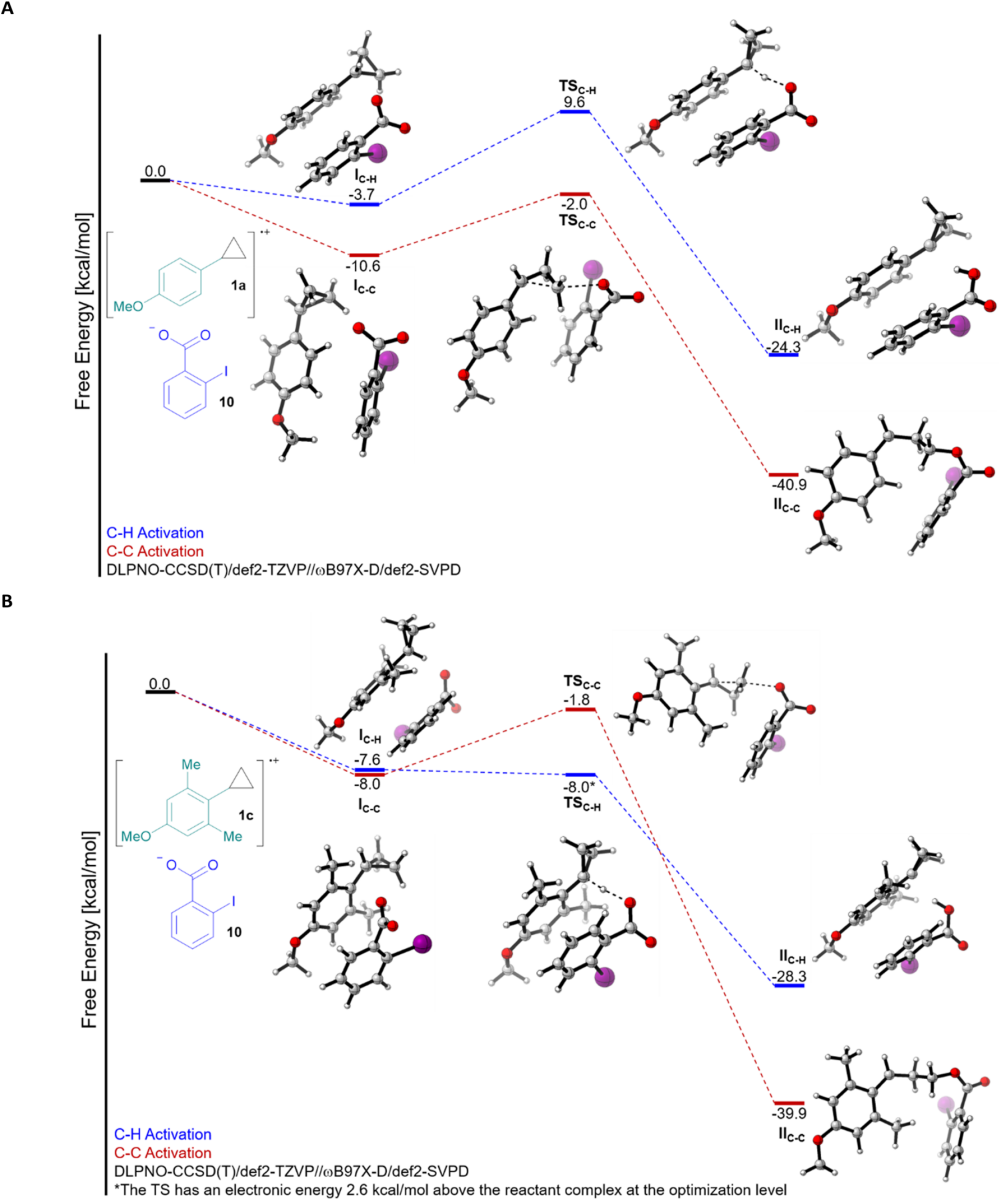
Free energy profile of the C–H and C–C activation of the radical cations of cyclopropanes 1a (A) and 1c (B) with 2-iodobenzoate (10).

Having established a better understanding of the substrates, we then computed the free energy profile for both C–H and C–C bond activation for the reaction of 1a and 1c with 2-iodobenzoate (10) (Fig. 1A and B). Computing this process required careful consideration of the electronic structure, we therefore turned to energies computed at the DLPNO-CCSD(T)/def2-TZVP level on geometries optimized at the ωB97X-D/def2-SVPD level (see ESI† for full computational details) to accurately describe the reaction free energies. On the level of the first interaction complex I, the pathway leading to C–C bond cleavage was favoured in both cases, although the difference was larger for 1a. The activation barrier for C–C bond cleavage was around 7–9 kcal mol^−1^ for both 1a and 1c (transition state TS_C–C_), and the reaction was highly exergonic, probably due to the release of ring strain. For C–H bond cleavage, a completely different picture emerged: A barrier of 13.3 kcal mol^−1^ made this process less favourable than C–C cleavage in the case of 1a, but in the case of 1c, the process became nearly barrierless (optimization level electronic energy +2.6 kcal mol^−1^), making C–H cleavage predominant (transition state TS_C–H_). The transition step itself is even 17.6 kcal mol^−1^ lower in energy for 1c than for 1a. While this is in full accordance with the experimental results, such an impressive effect is nevertheless surprising. From the steric/conformation point of view, we can already see that nearly no conformational change is needed going from I_C–H_ to TS_C–H_ for 1c, whereas it is not the case for 1a. This is in agreement with the fact that the perpendicular conformation is a transition state for 1a, but a minima for 1c, as discussed above. In addition, the resulting radical II_C–H_ is more stable by 4.0 kcal mol^−1^ for 1c, which could also make the process more favourable. However, the magnitude of the energy difference is surprising, even combining these two effects.^23^

## Conclusions

In summary, we have reported the first alkynylation of the C–H bond of arylcyclopropanes *via* the direct photoactivation of aryl-EBX reagents with visible light. We discovered a complete switch of the reaction outcome from C–C to C–H alkynylation when using arylcyclopropanes bearing two *ortho* substituents on the benzene ring, a result that could be reproduced computationally. The activating effect of this aryl ring enabled selective cyclopropane C–H bond functionalization in the presence of other weak benzylic or ethereal C–H bonds. We tentatively attributed this effect to the conformational constrains induced by the aryl ring, and we think this result will pave the way for the development of new selective transformations of cyclopropanes.

## Data availability

Raw data is available at zenodo.org: https://doi.org/10.5281/zenodo.7123955.

## Author contributions

T. V. T. N. planned the research and performed all the experiments described, prepared the material for the redaction of the manuscript and the supporting information. M. D. W. designed and performed the computation work, as well as prepared the related part of the manuscript and supporting information. J. W. supervised the research, participated to the redaction and finalization of the manuscript, as well as proof-read the supporting information.

## Conflicts of interest

There are no conflicts to declare.

## Supplementary Material

SC-013-D2SC04344K-s001
