# Supplementary material for: Substrate-controlled C–H or C–C alkynylation of cyclopropanes: generation of aryl radical cations by direct light activation of hypervalent iodine reagents
Source: Chem Sci. 2022 Oct 12;13(43):12831–9. doi: 10.1039/d2sc04344k (PMC9645386; doi:10.1039/d2sc04344k)
Supplement: SC-013-D2SC04344K-s001 [file SC-013-D2SC04344K-s001.pdf]

Electronic Supplementary Material (ESI) for Chemical Science.
This journal is © The Royal Society of Chemistry 2022

Substrate-Controlled C-H or C-C Alkynylation of Cyclopropanes: Generation of Aryl Radical Cations by Direct Light Activation of Hypervalent Iodine Reagents

Tin V. T. Nguyen, Matthew D. Wodrich and Jerome Waser*

SUPPORTING INFORMATION

Contents

General methods S2

Photochemical experimental set-ups S3

Synthesis of arylcyclopropanes, aminocyclopropanes. S4

Synthesis of hypervalent iodine reagents S18

Optimization S29

Results with other hypervalent iodine reagents S30

Synthesis of alkynylated products S31

Computational Details. S64

NMR spectra S68

References S134

General methods

For quantitative flash chromatography, distilled technical grade solvents were used. THF, Et_2O, toluene, hexane and CH_2Cl_2 were dried by passage over activated alumina under nitrogen atmosphere (H_2O content < 7 ppm, Karl-Fischer titration). All chemicals were purchased and used as received unless stated otherwise. Chromatographic purification was performed as flash chromatography using Macherey-Nagel silica 40-63, 60 Å, using the solvents indicated as eluent with 0.1-0.5 bar pressure. TLC was performed on Merck silica gel 60 F254 TLC plastic or aluminium plates and visualized with UV light, permanganate stain. Melting points were measured on a calibrated Büchi B-540 melting point apparatus using open glass capillaries. ^{1}H-NMR spectra were recorded at room temperature on a Brucker DPX-400 400 MHz spectrometer in $CDCl_3$, Acetone-d_6, CD_3CN or CD_3OD, all signals are reported in ppm with the internal chloroform signal at 7.26 ppm, the internal acetone signal at 2.09 ppm, the internal acetonitrile signal at 1.94 ppm and the internal methanol signal at 3.34 ppm as standard. The data is being reported as (s = singlet, d = doublet, t = triplet, q = quadruplet, p = quintet, m = multiplet or unresolved, br = broad signal, integration, coupling constant(s) in Hz, interpretation). ^{13}C-NMR spectra were recorded with 1H-decoupling on a Brucker DPX-400 101 MHz spectrometer in $CDCl_3$, Acetone-d_6, CD_3CN or CD_3OD, all signals are reported in ppm with the internal chloroform signal at 77.0 ppm, Acetone-d_6 signal at 29.8 ppm, CD_3CN signal at 1.3 ppm or CD_3OD signal at 49.0 ppm as standard. Infrared spectra were recorded on a JASCO FT-IR B4100 or a Bruker Alpha-P spectrophotometer with an ATR device and a ZnSe prism and are reported as cm-1 (w = weak, m = medium, s = strong). High resolution mass spectrometric measurements were performed by the mass spectrometry service of ISIC at the EPFL on a MICROMASS (ESI) Q-TOF Ultima API. HPLC measurements were done on a Agilent 1260 Infinity autosampler using a CHIRALPAK IA, IB, IC or IF column from DAICEL Chemical. The specific solvents and concentrations (in g/100 mL) are indicated.

All photocatalyzed reactions were carried out in oven dried glassware and under inert atmosphere (freeze pump thaw solvent stored on molecular sieves and under argon for maximum one week) unless specified otherwise. They were placed on a stirring plate with Kessil lamps (440 nm, 40 W) irradiating from both sides (the hood was free and coated with aluminum foil for personal protection). The distance between the Kessil lamps and the vials was approximatively 10 cm. Long irradiation resulted in temperature increasing up to 50 °C during overnight reactions unless a fan was used in which case the temperature raised to 30-35°C.

Photochemical experimental set-ups

0.2 mmol scale reactions (both set-ups can be used without affecting the yields)

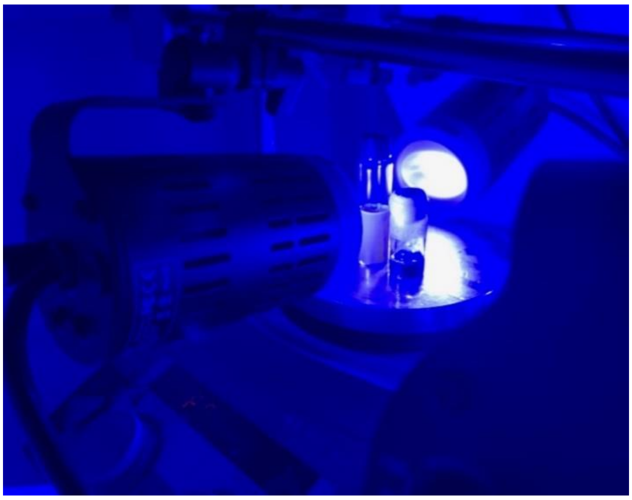

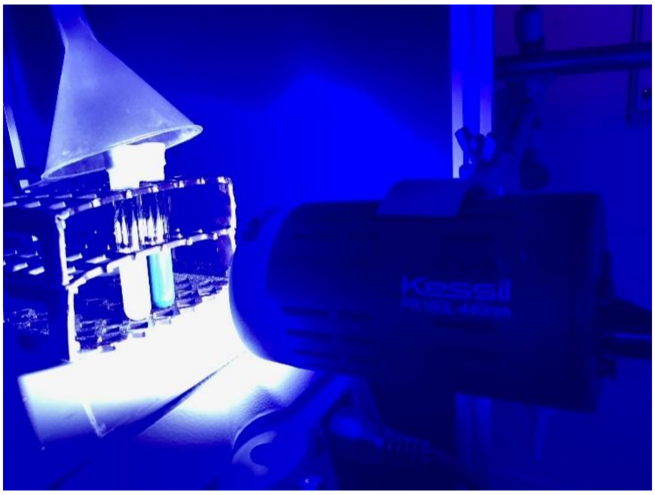

2 mmol scale reaction

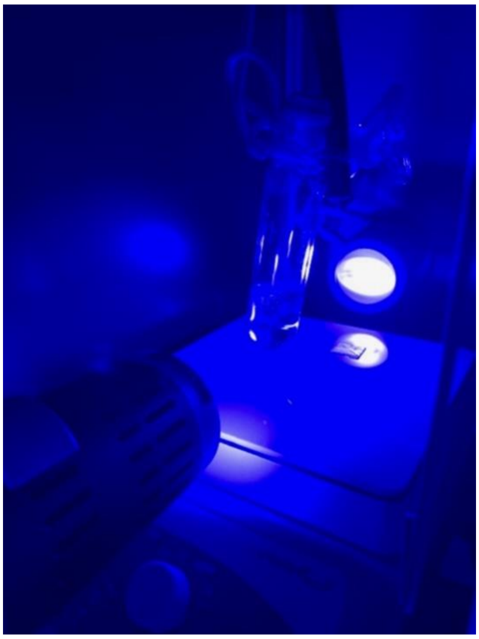

Synthesis of arylcyclopropanes, aminocyclopropanes.

- Compound **1u** (cyclopropyl benzene), **7a**, **7b**, **7c** are commercially available and were used as received without further purification.

General procedure A

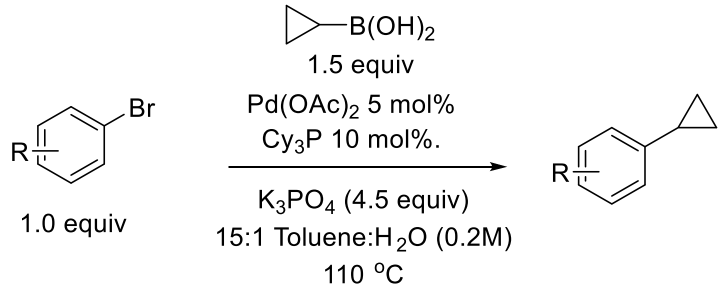

Following reported procedure,[1] to a solution of aryl bromide (5.00 mmol, 1.00 equiv) in 15:1 toluene:water (32 mL) was added potassium phosphate tribasic potassium (4.78 g, 22.5 mmol, 4.50 equiv), cyclopropylboronic acid (644 mg, 7.50 mmol, 1.50 equiv), 0.5 ml tricyclohexylphosphine (1M solution in toluene) and palladium(II) diacetate (56.1 mg, 250 µmol, 0.0500 equiv). The resulting mixture was heated to 110 °C and was stirred at this temperature overnight. The reaction mixture was then cooled to room temperature, diluted with DCM (100 mL) and washed with water (100 mL). The aqueous layer was extracted with DCM (3 × 100 mL). The combined organic layers were washed with brine (50 mL), dried over Na_2SO_4, filtered and concentrated under reduced pressure. The crude mixture was purified by column chromatography on Biotage (Büchi flashpure cartridge 40 g, gradient of pentane: ethyl acetate from 99:1 to 95:5). to obtain the product.

1-Cyclopropyl-4-methoxybenzene (1a)

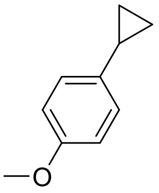

Following the general procedure A, starting from 1-bromo-4-methoxybenzene (935 mg, 5.00 mmol, 1.00 equiv), 1-cyclopropyl-4-methoxybenzene **1a** (580 mg, 3.91 mmol, 78% yield) was obtained as a colorless liquid. **^{1}H NMR** (400 MHz, $CDCl_3$) δ 7.09 – 6.97 (m, 2H, Ar*H*), 6.90 – 6.77 (m, 2H, Ar*H*), 3.78 (s, 3H, OC*H*$_3$), 1.86 (m, 1H, C*H*CH$_2$), 0.98 – 0.84 (m, 2H, CHC*H*$_2$), 0.71 – 0.57 (m, 2H, CHC*H*$_2$). **^{13}C NMR** (101 MHz, $CDCl_3$) δ 157.7, 136.0, 127.0, 113.9, 55.4, 14.7, 8.6. Consistent with reported data.[1]

1-Cyclopropyl-4-methoxy-2-methylbenzene (1b)

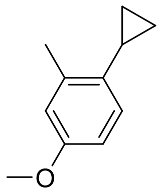

Following the general procedure A, starting from 1-bromo-4-methoxy-2-methylbenzene (1.01 g, 5.00 mmol, 1.00 equiv), obtained 1-cyclopropyl-4-methoxy-2-methylbenzene **1b** (622 mg, 3.83 mmol, 77% yield) as colorless oil**.** **Rf** = 0.44 (SiO_2, 40:1 pentane:ethyl acetate) **^{1}H NMR** (400 MHz, $CDCl_3$) δ 6.94 (d, *J* =8.4 Hz, 1H), 6.73 (d, *J* =2.8 Hz, 1H), 6.66 (dd, *J* =8.4, 2.8 Hz, 1H, Ar*H*), 3.78 (s, 3H, Ar*H*), 2.41 (s, 3H, ArC*H₃*), 1.81 (tt, *J* =8.4, 5.4 Hz, 1H, C*H*CH_2), 0.99 – 0.79 (m, 2H, CHC*H₂*), 0.67 – 0.52 (m, 2H, CHC*H₂*). **^{13}C NMR** (101 MHz, $CDCl_3$) δ 157.8, 139.5, 133.6, 127.2, 115.6, 110.6, 55.3, 19.9, 13.0, 6.5. **HRMS** (APPI/LTQ-Orbitrap) m/z: $[M]^+$ Calcd for $C_{11}H_{14}O^+$ 162.1039; Found 162.1038.

2-Cyclopropyl-5-methoxy-1,3-dimethylbenzene (1c)

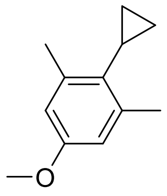

Following the general procedure A, starting from 2-bromo-5-methoxy-1,3-dimethylbenzene (1.08 g, 5.00 mmol, 1.00 equiv), obtained 2-cyclopropyl-5-methoxy-1,3-dimethylbenzene **1c** (630 mg, 3.57 mmol, 71% yield) as a colorless oil. **Rf** = 0.47 (SiO_2, 40:1 pentane:ethyl acetate) **^{1}H NMR** (400 MHz, $CDCl_3$) δ 6.57 (s, 2H, Ar*H*), 3.77 (s, 3H, OC*H₃*), 2.42 (s, 6H, ArC*H₃*), 1.71 – 1.58 (m, 1H, ArC*H*CH_2), 1.05 – 0.89 (m, 2H, CHC*H₂*), 0.57 – 0.43 (m, 2H, CHC*H₂*). **^{13}C NMR** (101 MHz, $CDCl_3$) δ 157.7, 140.4, 131.7, 113.2, 55.2, 21.0, 11.5, 8.2. **HRMS** (APPI/LTQ-Orbitrap) m/z: $[M]^+$ Calcd for $C_{12}H_{16}O^+$ 176.1196; Found 176.1193.

2-Cyclopropyl-1,3-dimethylbenzene (1d)

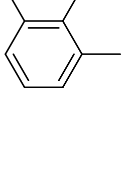

Following the general procedure A, starting from 2-bromo-1,3-dimethylbenzene (925 mg, 5.00 mmol, 1.00 equiv), 2-cyclopropyl-1,3-dimethylbenzene (**1d**) (530 mg, 3.62 mmol, 72% yield) was obtained as a colorless oil. **^{1}H NMR** (400 MHz, $CDCl_3$) δ 7.09 – 6.95 (m, 3H, Ar*H*), 2.43 (s, 6H, ArC*H₃*), 1.71 (ddd, *J* =14.4, 8.4, 6.0 Hz, 1H, ArC*H*C*H₃*), 1.10 – 0.97 (m, 2H, CH_2), 0.60 – 0.49 (m, 2H, CH_2). **^{13}C NMR** (101 MHz, $CDCl_3$) δ 139.1, 139.0, 127.9, 126.1, 20.7, 12.2, 8.1. **HRMS** (APPI/LTQ-Orbitrap) m/z: $[M]^+$ Calcd for $C_{11}H_{14}^+$ 146.1090; Found 146.1088 Consistent with reported data.[2]

2-Cyclopropyl-1,3,5-trimethylbenzene (1e)

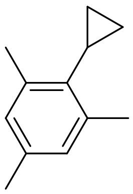

Following the reported procedure.[3] 2-cyclopropyl-1,3,5-trimethylbenzene (**1e**) was obtained as a colorless oil (504 mg, 63%). **^{1}H NMR** (400 MHz, $CDCl_3$) δ 6.87 (s, 2H. Ar*H*), 2.43 (s, 6H, C*H₃*Ar), 2.30 (s, 3H, C*H₃*Ar), 1.70 (m, 1H, C*H*CH_2), 1.08 – 0.98 (m, 2H, CHC*H₂*), 0.55 (m, 2H, CHC*H₂*). Consistent with reported data.[3]

General procedure B

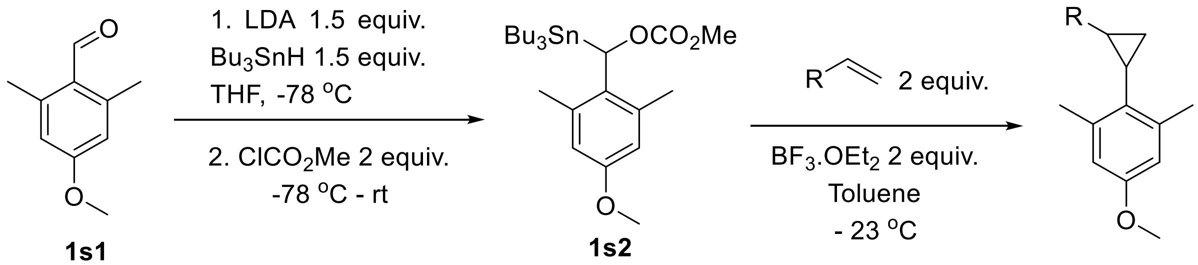

1s1 **1s2**

Following slightly modified reported procedure,[4] dry tetrahydrofuran (80 mL) was cooled to -78 °C, then a solution of lithium diisopropylamide (2.0 M in heptane/tetrahydrofuran/ethylbenzene, 19.00 mL, 37.50 mmol) was added, followed by tri-butyltin hydride (9.81 mL, 37.5 mmol) dropwise. After 5 minutes, the mixture was warm to 0 °C for 0.5 hours, then recooled to -78 °C. 2,6-Dimethyl-4-methoxybenzaldehyde **1s1** (30 mmol) was added dropwise, and the reaction mixture was stirred at this temperature overnight. Afterwards, methyl chloroformate (3.41 mL, 44.1 mmol) was added dropwise, the

cooling bath was removed, and the mixture was allowed to stir overnight at room temperature. Then a solution of saturated aqueous ammonium chloride (100 mL) was added followed by extraction with ethyl acetate. The organic extract was dried, filtered and concentrated. The residue was purified by chromatography (silica gel, ethyl acetate in hexanes) which afforded (6.90 g, 13.5 mmol, 45% yield) of the desired product **1s2**

Following a reported procedure,[4] to a 50 mL oven-dried round-bottom flask equipped with a stirring bar was added the solution of **1s2** (1.0 mmol, 1.0 equiv) and the corresponding alkene (2 mmol, 2 equiv) in toluene (3.5 mL) under nitrogen atmosphere at room temperature. The reaction was cooled to -23 °C. $BF_3·OEt_2$ (156 mg, 1.10 mmol, 1.10 equiv) was added dropwise and the reaction mixture was stirred at this temperature overnight. After the reaction reached completion according to the TLC analysis, the reaction mixture was quenched with sat. $NaHCO_3$ and extracted with EtOAc for 3 times. The combined organic layers were washed with brine, dried over Na_2SO_4 and filtered. After concentration under reduced pressure, the crude residue was purified by column chromatography (PE : EtOAc) to afford the arylcyclopropanes.

2-(-2-Benzylcyclopropyl)-5-methoxy-1,3-dimethylbenzene (1f)

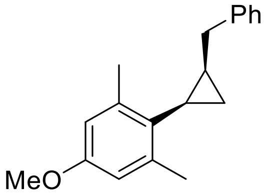

Following the general procedure B, starting from **1s2** (513 mg, 1.00 mmol, 1.00 equiv) and allyl benzene (236 mg, 2.00 mmol, 2.00 equiv), 2-(-2-benzylcyclopropyl)-5-methoxy-1,3-dimethylbenzene **(1f)** was obtained as a colorless oil (152 mg, 0.570 mmol, 57% yield). dr. > 20:1. **Rf** = 0.34 (SiO_2, 40:1 pentane:ethyl acetate) **^{1}H NMR** (400 MHz, $CDCl_3$) δ 7.32 – 7.24 (m, 2H, Ar*H*), 7.19 (m, 3H, Ar*H*), 6.62 (s, 2H, Ar*H*), 3.80 (s, 3H, ArOC*H₃*), 2.89 (dd, *J* =14.8, 3.8 Hz, 1H, CHC*H₂*Ar), 2.43 (s, 6H, ArC*H₃*), 1.93 (q, *J* =8.0 Hz, 1H, C*H*Ar), 1.68 – 1.55 (m, 1H, C*H*CH₂Ar), 1.54 – 1.44 (m, 1H, CHC*H₂*Ar), 1.29 (tdd, *J* =8.4, 5.0, 1.2 Hz, 1H, CHC*H₂*Ar), 0.68 (dt, *J* =6.8, 5.1 Hz, 1H, CHC*H₂*Ar). **^{13}C NMR** (101 MHz, $CDCl_3$) δ 157.5, 142.3, 128.5, 128.3, 125.8, 113.5, 55.2, 35.6, 21.3, 18.3, 18.3, 14.6. 2 carbon signals were not resolved. **IR** (vmax, cm^{-1}) 3023 (m), 2993 (m), 2955 (m), 2919 (m), 1578 (m), 1499 (m), 1483 (s), 1468 (m), 1450 (s), 1439 (m), 1309 (s),

1190 (m), 1172 (s), 1147 (s), 1074 (s), 848 (m). **HRMS (ESI/QTOF) m/z:** [M + H]+ Calcd for $C_{19}H_{23}O+$ 267.1743; Found 267.1741.

7-(4-Methoxy-2,6-dimethylphenyl)bicyclo[4.1.0]heptane (1g)

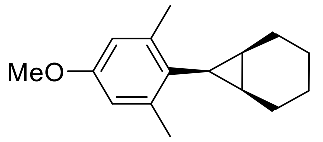

Following the general procedure B, starting from **1s2** (513 mg, 1.00 mmol, 1.00 equiv) and cyclohexene (164 mg, 2.00 mmol, 2.00 equiv), 7-(4-methoxy-2,6-dimethylphenyl)bicyclo[4.1.0]heptane **1g** was obtained as colorless oil (80.6 mg, 0.350 mmol, 35% yield). **Rf** = 0.50 (SiO_2, 40:1 pentane:ethyl acetate). dr. : 86:14. **^{1}H NMR** (400 MHz, $CDCl_3$) δ 6.58 (s, 2H, Ar*H*), 3.77 (s, 3H, ArOC*H*$_3$), 2.37 (s, 6H, ArC*H*$_3$), 1.87 – 1.77 (m, 2H,C*H*$_2$), 1.71 (t, *J* =8.5 Hz, 1H, C*H*CHCH$_2$), 1.28 – 1.24 (m, 3H, CHC*H*C*H*$_2$), 1.12 – 1.05 (m, 2H, C*H*$_2$), 1.03 – 0.93 (m, 2H, C*H*$_2$), 0.86 (t, *J* =7.2 Hz, 1H, CHC*H*CH$_2$).**^{13}C NMR** (101 MHz, $CDCl_3$) δ 157.4, 141.2, 127.5, 113.1, 55.1, 20.7, 20.7, 20.5, 20.1, 14.6. **IR** (vmax, cm^{-1}) 2999 (m), 2991 (m), 2922 (s), 2851 (s), 1603 (s), 1577 (m), 1484 (s), 1464 (s), 1313 (s), 1189 (m), 1154 (m), 1145 (s), 1064 (s), 850 (m), 833 (m). **HRMS (Sicrit plasma/LTQ-Orbitrap) m/z**: $[M]^+$ Calcd for $C_{16}H_{22}O^+$ 230.1665; Found 230.1662.

1-(4-Methoxy-2,6-dimethylphenyl)spiro[2.3]hexane-5-carbonitrile (1h)

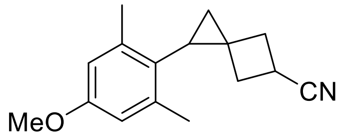

Following the general procedure B, starting from **1s2** (513 mg, 1.00 mmol, 1.00 equiv) and 3-Methylenecyclobutanecarbonitrile (186 mg, 0.200 mmol, 2.00 equiv). 1-(4-methoxy-2,6-dimethylphenyl)spiro[2.3]hexane-5-carbonitrile **1h** was obtained as pale yellow oil (65 mg, 0.27 mmol, 27%). dr. 53:47. **Rf** = 0.58 (SiO_2, 10:1 pentane:ethyl acetate). **^{1}H NMR** (400 MHz, $CDCl_3$) δ 6.58 (s, 2H, Ar*H*), 3.76 (s, 3H, ArOC*H*$_3$), 3.33 (tt, *J* =9.4, 8.1 Hz, 1H, C*H*$_2$), 2.90 (dd, *J* =11.8, 8.1 Hz, 1H, C*H*$_2$), 2.53 (ddt, *J* =11.5, 9.4, 1.7 Hz, 1H, C*H*$_2$), 2.40 (d, *J* =0.7 Hz, 6H, ArC*H*$_3$), 2.33 – 2.30 (m, 1H, CH$_2$C*H*), 2.06 – 1.99 (m, 1H, CH$_2$C*H*), 1.73 – 1.68 (m, 1H, C*H*$_2$), 1.30 – 1.26

(m, 1H, C*H₂*), 0.88 – 0.85 (m, 1H, C*H₂*). **^{13}C NMR** (101 MHz, $CDCl_3$) δ 157.7, 140.0, 127.5, 122.7, 113.7, 55.2, 35.6, 33.2, 23.7, 23.3, 22.4, 21.4, 17.4. **IR** (vmax, cm-1) 2938 (m), 2859 (w), 2838 (w), 2236 (w), 1734 (w), 1603 (s), 1486 (s), 1453 (m), 1313 (s), 1197 (m), 1150 (s), 1067 (s), 856 (m). **HRMS (ESI/QTOF) m/z:** [M + H]+ Calcd for $C_{16}H_{20}NO^+$ 242.1539; Found 242.1539.

1-(4-Methoxy-2,6-dimethylphenyl)spiro[2.5]octane (1i)

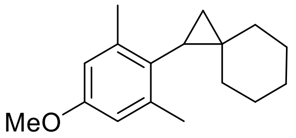

Following the general procedure B, starting from **1s2** (513 mg, 1.00 mmol, 1.00 equiv) and methylenecyclohexane (192 mg, 2.00 mmol, 2.00 equiv). 1-(4-methoxy-2,6-dimethylphenyl)spiro[2.5]octane **1i** was obtained as colorless oil (139 mg, 0.570 mmol, 57% yield). **Rf** = 0.62 (SiO_2, 40:1 pentane:ethyl acetate). **^{1}H NMR** (400 MHz, $CDCl_3$) δ 6.54 (s, 2H, Ar*H*), 3.76 (s, 3H, ArOC*H₃*), 2.34 (s, 6H, ArC*H₃*), 1.98 (tdd, *J* =12.8, 3.7, 1.6 Hz, 1H, C*H₂*), 1.78 – 1.68 (m, 2H,C*H₂*), 1.62 – 1.59 (m, 1H, C*H*CH₂), 1.59 – 1.55 (m, 1H, C*H₂*), 1.53 – 1.44 (m, 1H, C*H₂*), 1.44 – 1.37 (m, 1H, C*H₂*), 1.36 – 1.15 (m, 2H, C*H₂*), 1.05 – 0.96 (m, 1H, C*H₂*), 0.89 (ddd, *J* =8.7, 4.5, 1.8 Hz, 1H, C*H₂*), 0.58 (ddd, *J* =6.3, 4.5, 1.6 Hz, 1H, C*H₂*), 0.53 – 0.46 (m, 1H, C*H₂*). **^{13}C NMR** (101 MHz, $CDCl_3$) δ 157.1, 129.2, 113.2, 55.1, 36.8, 31.9, 26.8, 26.4, 25.8, 25.6, 21.4, 20.5. Two carbon signals were not resolved. **IR** (vmax, cm-1) 2923 (s), 2848 (s), 1605 (m), 1482 (m), 1448 (m), 1318 (m), 1314 (s), 1193 (m), 1150 (s), 1072 (m), 852 (m) **HRMS** (nanochip-ESI/LTQ-Orbitrap) m/z: [M]+ Calcd for $C_{17}H_{24}O^+$ 244.1822; Found 244.1821.

Ethyl 2-(4-methoxy-2,6-dimethylphenyl)cyclopropane-1-carboxylate (1k)

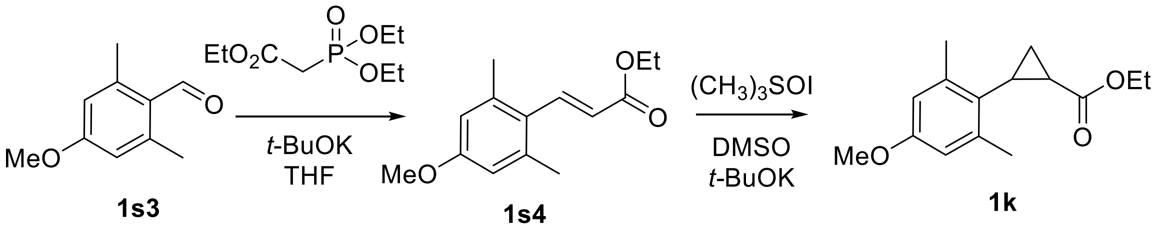

Following a reported procedure,[5] to a solution of potassium tertbutoxide (5.31 g, 45.0 mmol, 2.00 equiv), triethyl phosphonoacetate (9.30 mL, 45.0 mmol, 2.00 equiv) in freshly distilled THF (120 mL), was added 4'-methoxy-2',6'-dimethylbenzaldehyde **1s3** (4.14 g, 25.2 mmol, 1.00 equiv) in one portion at room temperature. The reaction was stirred at room

temperature for 2 h. Then AcOEt and H_2O were added to the solution, the organic layer was evaporated in vacuo and the residue was purified by silica-gel column chromatography to give product **1s4**. NMR analysis was consistent with reported value.[5]

Following a reported procedure with a little modification,[6] a suspension of trimethylsulfoxonium iodide (264 mg, 1.20 mmol, 1.20 equiv.) and potassium *tert*-butoxide (168 mg, 1.50 mmol, 1.50 equiv) in anhydrous DMSO (5.00 mL) was stirred for 1 h. A DMSO solution (5.00 mL) of **1s4** (234 mg, 1.00 mmol, 1.00 equiv) was added at 0 °C. The reaction mixture was stirred at 50 °C for 2 h. Another suspension of trimethylsulfoxonium iodide (66 mg, 0.30 mmol, 0.30 equiv.) and potassium *tert*-butoxide (33 mg, 0.30 mmol, 0.30 equiv) in DMSO (2.00 mL) was added to the reaction mixture and the reaction was stirred at 50 °C for 24 h. The solution was poured into a brine solution (10 mL) and extracted with ethyl acetate (3x10 mL). The combined organic layers were washed with water and dried over $MgSO_4$, concentrated and purified by Colum chromatography (SiO_2, 20:1 pentane:ethyl acetate) to afford cyclopropane **1k** (trans) as sticky solid (37 mg, 0.15 mmol, 15% yield). **Rf** = 0.41 (SiO_2, 20:1 pentane:ethyl acetate). **^{1}H NMR** (400 MHz, $CDCl_3$) δ 6.56 (s, 2H, Ar*H*), 4.31 – 4.14 (m, 2H, O$CH_2$$CH_3$), 3.76 (s, 3H, O$CH_3$), 2.36 (s, 6H, Ar$CH_3$), 2.27 (ddd, *J* =8.8, 7.2, 4.8 Hz, 1H, ArC*H*CH_2), 1.70 (dd, *J* =8.9, 4.1 Hz, 1H, COC*H*CH_2), 1.65 (ddd, *J* =9.0, 5.3, 4.1 Hz, 1H, CHCH_2), 1.31 (t, *J* =7.1 Hz, 3H, OCH_2CH_3), 1.12 (ddd, *J* =8.1, 7.1, 3.9 Hz, 1H, CHCH_2). **^{13}C NMR** (101 MHz, $CDCl_3$) δ 174.5, 158.2, 140.0, 128.7, 113.5, 60.7, 55.2, 23.4, 23.0, 21.0, 17.8, 14.6.; Found 248.1403. **IR** (vmax, cm-1) 2980 (w), 2838 (w), 1724 (s), 1605 (m), 1449 (m), 1313 (s), 1191 (s), 1173 (s), 1149 (s), 1068 (m), 860 (m). **HRMS** (Sicrit plasma/LTQ-Orbitrap) m/z: [M]+ Calcd for $C_{15}H_{20}O_3^+$ 248.1407

2-Cyclopropyl-1,3-diethyl-5-methoxybenzene (1l)

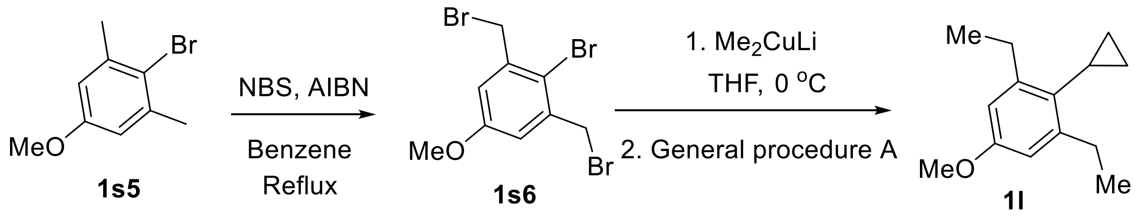

Following a reported procedure,[7] to a solution of compound **1s5** (1.90 g, 8.88 mmol, 1.00 equiv) in benzene (25 mL) was added N-bromosuccinimide (3.32 g, 18.6 mmol, 2.00 equiv) and azobisisobutyronitrile (145 mg, 0.890 mmol, 0.10 equiv). The reaction mixture was

heated to reflux for 3 hours, then NBS (0.330 g, 1.86 mmol, 0.20 equiv) and azobisisobutyronitrile (14.5 mg, 0.0890 mmol, 0.01 equiv) were added and the reflux continued for 30 minutes. The solvent was removed, and the residue was extracted with ethyl acetate, washed with brine, and dried over anhydrous Na_2SO_4. The solvent was concentrated in vacuo, and the residue was purified by column chromatography (pentane/ethyl acetate=25:1) to obtain **1s6** as a white solid (0.910 g, 2.44 mmol, 27% yield). Analytical data was consistent with reported value.[7]

Following a reported procedure,[8] a solution of lithium dimethylcuprate which had been prepared from cuprous iodide (609.0 mg, 3.20 mmol, 1.00 equiv) and methyllithium (4.0 mL, 1.6 M in Et_2O, 6.4 mmol, 2 equiv) in dry THF was added to a solution of **1s6** (744 mg, 2.00 mmol, 1.00 equiv) in dry THF (10 mL) at 0 °C. The mixture was stirred at 0 °C for 2 h, then quenched with methanol. Saturated NH_4Cl (10 mL) was added, extracted with ethyl acetate (3x10 mL), the combined organic layers were dried over Na_2SO_4, concentrated in vacuo, purified by flash column chromatography (SiO_2, Gradient 100:0 to 40:1 pentane:ethyl acetate) to give the desired product (1.120 mmol, 270.0 mg, 56%). The product was directly used for the next step.

Following general procedure A, starting from the corresponding aryl bromide from previous step, 2-cyclopropyl-1,3-diethyl-5-methoxybenzene (**1l**) was obtained as colorless oil (97.9 mg, 0.480 mmol, 24% yield for 2 steps). **Rf** = 0.65 (SiO_2, 40:1 pentane:ethyl acetate). **^{1}H NMR** (400 MHz, $CDCl_3$) δ 6.60 (s, 2H, Ar*H*), 3.79 (s, 3H, ArOC*H₃*), 2.87 (q, *J* =7.5 Hz, 4H, ArC*H₂*CH₃), 1.71 – 1.59 (m, 1H, C*H*CH₂), 1.23 (t, *J* =7.6 Hz, 6H, ArCH₂C*H₃*), 1.06 – 0.95 (m, 2H, CHC*H₂*), 0.57 – 0.46 (m, 2H, CHC*H₂*). **^{13}C NMR** (101 MHz, $CDCl_3$) δ 158.2, 146.6, 130.6, 111.4, 55.2, 26.6, 15.2, 10.8, 8.3. **IR** (ν_{max}, cm^{-1}) 2965 (s), 2935 (m), 2874 (m), 2835 (w), 1603 (s), 1469 (s), 1333 (s), 1288 (m), 1146 (s), 1145 (s), 1087 (m), 1080 (m), 1033 (s). **HRMS** (ESI/QTOF) m/z: $[M + H]^+$ Calcd for $C_{14}H_{21}O^+$ 205.1587; Found 205.1593.

2-Cyclopropyl-5-(cyclopropylmethoxy)-1,3-dimethylbenzene (1m)

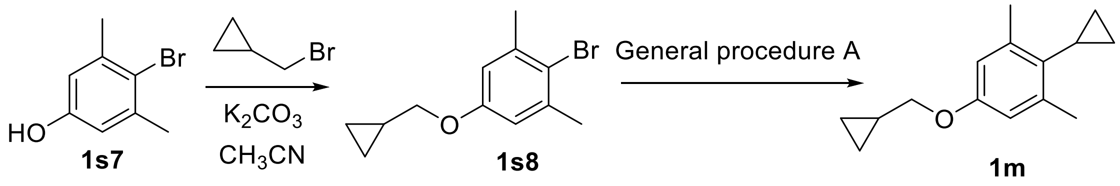

Following a slightly modified reported procedure, to a solution of **1s7** (402 mg, 2.00 mmol, 1.00 equiv) in acetonitrile (20 mL) was added K_2CO_3 (0.910 g, 6.57 mmol, 3.20 equiv) at room temperature. Then a solution of (bromomethyl)cyclopropane (810 mg, 6.00 mmol, 3.00 equiv) in acetonitrile (10 mL) was added at room temperature and the reaction mixture was stirred at 50 ^oC overnight. Then, the reaction mixture was filtrated and concentrated under vacuum. The product **1s8** was directly used for the next step without further purification.

Following general procedure A, 2-cyclopropyl-5-(cyclopropylmethoxy)-1,3-dimethylbenzene **1m** was obtained as colorless oil (142.7 mg, 0.660 mmol, 33% yield). **Rf** = 0.56 (SiO_2, 40:1 pentane:ethyl acetate). **^{1}H NMR** (400 MHz, $CDCl_3$) δ 6.56 (s, 2H, Ar*H*), 3.75 (d, *J* =7.0 Hz, 2H, ArOC*H₂*), 2.39 (s, 6H, ArC*H₃*), 1.62 (ddd, *J* =14.1, 8.4, 5.7 Hz, 1H, C*H*CH₂), 1.32 – 1.20 (m, 1H, C*H*CH₂), 1.02 – 0.91 (m, 2H, CHC*H₂*), 0.67 – 0.57 (m, 2H, CHC*H₂*), 0.48 (td, *J* =6.0, 4.2 Hz, 2H, CHC*H₂*), 0.32 (dt, *J* =6.0, 4.6 Hz, 2H, CHC*H₂*). **^{13}C NMR** (101 MHz, $CDCl_3$) δ 157.1, 140.3, 131.6, 113.9, 72.7, 21.0, 11.5, 10.5, 8.2, 3.3. **IR** (vmax, cm-1) 3080 (m), 3004 (m), 2865 (m), 1605 (s), 1487 (s), 1311 (s), 1147 (s), 1069 (s), 1046 (s), 1023 (s). **HRMS** (Sicrit plasma/LTQ-Orbitrap) m/z: $[M + H]^+$ Calcd for $C_{15}H_{21}O^+$ 217.1587; Found 217.1583.

4-Methoxy-2,6-dimethylphenyl)cyclopropyl)methyl 2-(11-oxo-6,11 dihydrodibenzo[b,e]oxepin-2-yl)acetate (1n)

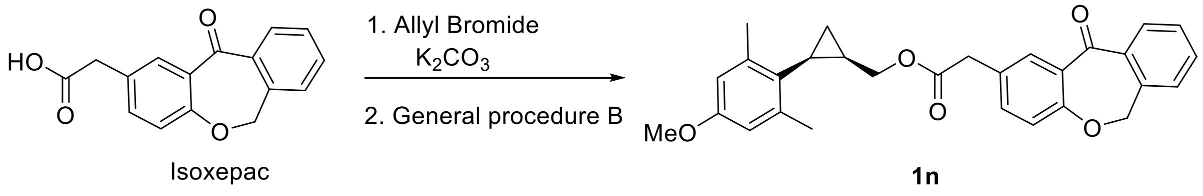

To a solution of Isoxepac (536 mg, 2.00 mmol, 1.00 equiv) in DMF (20 mL) was added K_2CO_3 (817 mg, 6.00 mmol, 3.00 equiv) then allyl bromide (0.52 mL, 6.0 mmol, 3.00 equiv). The mixture was stirred overnight, then the mixture was extracted with Et_2O (3x20 mL) and H_2O (20 mL), and the organics were washed with brine, dried over Na_2SO_4, and the solvent was evaporated. The product was directly used for the next step without further purification.

Following general procedure B, product **1n** was obtained as sticky solid (301 mg, 0.660 mmol, 33% yield for 2 steps). dr. >20:1. **Rf** = 0.26 (SiO_2, 10:1 pentane:ethyl acetate). **^{1}H NMR** (400 MHz, $CDCl_3$) δ 8.10 (d, *J* =2.4 Hz, 1H, Ar*H*), 7.89 (dd, *J* =7.7, 1.4 Hz, 1H, Ar*H*), 7.56 (td, *J* =7.4, 1.4 Hz, 1H, Ar*H*), 7.47 (td, *J* =7.6, 1.3 Hz, 1H, Ar*H*), 7.42 – 7.34 (m, 2H, Ar*H*), 7.01 (d, *J* =8.5 Hz,

1H, Ar*H*), 6.52 (s, 2H, Ar*H*), 5.18 (s, 2H, ArC*H₂*), 4.14 (dd, *J* =11.1, 5.6 Hz, 1H, COOC*H₂*CH), 3.74 (s, 3H, ArOC*H₃*), 3.59 (s, 2H, ArC*H₂*), 3.36 (dd, *J* =11.6, 8.7 Hz, 1H, , COOC*H₂*CH), 2.35 (s, 6H, ArC*H₃*), 1.96 (q, *J* =8.1 Hz, 1H, , C*H*CH₂), 1.61 – 1.53 (m, 1H, C*H*CH₂), 1.34 – 1.28 (m, 1H, C*H₂*CHAr), 0.79 (dt, *J* =7.0, 5.3 Hz, 1H, C*H₂*CHAr). **^{13}C NMR** (101 MHz, $CDCl_3$) δ 191.0, 171.6, 160.5, 157.7, 140.6, 140.1, 136.4, 135.7, 132.9, 132.5, 129.6, 129.4, 128.0, 127.9, 126.8, 125.2, 121.1, 113.6, 73.7, 66.6, 55.2, 40.4, 21.2, 18.1, 15.9, 12.7. **IR** (vmax, cm-1) 2931 (w), 2366 (w), 1733 (s), 1648 (s), 1605 (m), 1489 (s), 1414 (m), 1300 (s), 1141 (s), 1015 (m), 761 (m). **HRMS** (ESI/QTOF) m/z: $[M + Na]^+$ Calcd for $C_{29}H_{28}NaO_5$+ 479.1829; Found 479.1833.

1-Cyclopropyl-2-methoxybenzene (1o)

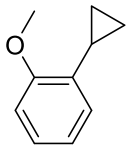

Following the general procedure A, starting from 1-bromo-2-methoxybenzene (935 mg, 5.00 mmol, 1.00 equiv), obtained 1-cyclopropyl-2-methoxybenzene **(1o)** (620 mg, 4.18 mmol, 84% yield) as colorless oil. **^{1}H NMR** (400 MHz, $CDCl_3$) δ 7.57 - 7.50 (m, 1H, Ar*H*), 7.37 – 7.17 (m, 3H, Ar*H*), 4.26 (s, 3H, OC*H₃*), 2.60 - 2.53 (m, 1H, C*H*CH₂), 1.41 – 1.25 (m, 2H, CHC*H₂*), 1.13 – 1.00 (m, 2H, CHC*H₂*). **^{13}C NMR** (101 MHz, $CDCl_3$) δ 158.4, 132.1, 126.4, 124.9, 120.7, 110.3, 55.7, 9.4, 7.8. HRMS (APPI/LTQ-Orbitrap) m/z: $[M]^+$ Calcd for $C_{10}H_{12}O^+$ 148.0883; Found 148.0880. Consistent with reported data.[9]

1-cyclopropyl-4-ethoxybenzene (1p)

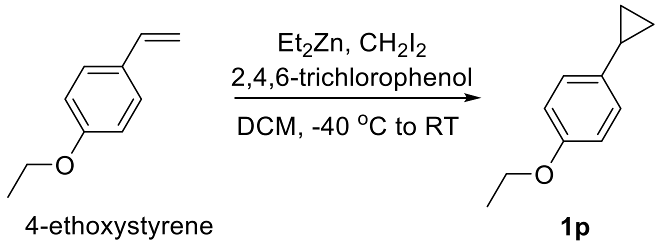

Following reported procedure,[1] in a 150 mL oven-dried round-bottom flask, was added 2,4,6-trichlorophenol (1.18 g, 6.0 mmol, 2.5 equiv) under nitrogen atmosphere. DCM (60 mL, 0.1 M) was added into the flask and the reaction mixture was cooled to -40 °C. $ZnEt_2$ (1.0 M, 6.0 mL, 6.0 mmol, 2.50 equiv) was added slowly into the flask by syringe and the reaction mixture was stirred at this temperature for 15 minutes. CH_2I_2 (2.57 g, 9.06 mmol, 4.00 equiv) was

added slowly by syringe and the reaction mixture was stirred at this temperature for another 15 minutes. Next, the corresponding solution of 4-ethoxystyrene (355.2 mg, 2.40 mmol, 1.00 equiv) in DCM (10 mL) was added by syringe and the reaction mixture was allowed to warm to room temperatureand and was stirred overnight. After the reaction reached completion (as judged by ^{1}H-NMR), the reaction mixture was quenched with sat. NH_4Cl (30 mL) in 30 minutes and extracted with DCM (50 mL) for 3 times. The combined organic layers were washed with aq. NaOH (1.0 M, 30 mL) and brine (20 mL), dried over Na_2SO_4 and filtered. The mixture was concentrated under reduced pressure, the crude residue was purified by column chromatography on Biotage (Büchi flashpure cartridge 40 g, gradient of pentane: ethyl acetate from 99:1 to 95:5) to afford the desired compound 1-cyclopropyl-4-ethoxybenzene (243.2 mg, 1.50 mmol, 63%) as colorless oil. **Rf** = 0.50 (SiO_2, 40:1 pentane:ethyl acetate. **^{1}H NMR** (400 MHz, $CDCl_3$) δ 7.08 – 6.97 (m, 2H), 6.86 – 6.77 (m, 2H), 4.01 (q, *J* =7.0 Hz, 2H), 1.86 (tt, *J* =8.5, 5.1 Hz, 1H), 1.41 (t, *J* =7.0 Hz, 3H), 0.95 – 0.87 (m, 2H), 0.63 (dt, *J* =6.5, 4.5 Hz, 2H). **^{13}C NMR** (101 MHz, $CDCl_3$) δ 157.0, 135.8, 126.9, 114.5, 63.6, 15.0, 14.7, 8.6. **HRMS** (APCI/QTOF) m/z: $[M + H]^+$ Calcd for $C_{11}H_{15}O^+$ 163.1117; Found 163.1113

1-Tert-butyl-4-cyclopropylbenzene (1q)

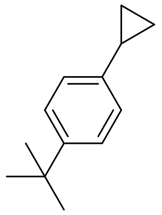

Following the general procedure A, starting from 1-bromo-4-tert-butylbenzene (1.07 g, 5.00 mmol, 1.00 equiv), 1-*tert*-butyl-4-cyclopropylbenzene (**1q**) (710 mg, 4.07 mmol, 81% yield) was obtained as a colorless oil. **^{1}H NMR** (400 MHz, $CDCl_3$) δ 7.33 – 7.27 (m, 2H, ArH), 7.07 – 7.00 (m, 2H, ArH), 1.88 (tt, *J* =8.4, 5.1 Hz, 1H, ArC*H*), 1.31 (s, 9H, $C(CH_3)_3$), 0.97 – 0.89 (m, 2H, CHC*H₂*), 0.74 – 0.64 (m, 2H, CHC*H₂*). **^{13}C NMR** (101 MHz, $CDCl_3$) δ 148.4, 141.0, 125.5, 125.3, 34.5, 31.5, 15.0, 9.1. **HRMS** (APPI/LTQ-Orbitrap) m/z: $[M]^+$ Calcd for $C_{13}H_{18}^+$ 174.1403; Found 174.1400. Consistent with reported data.[10]

1-cyclopropyl-4-fluoro-2-methoxybenzene (1r)

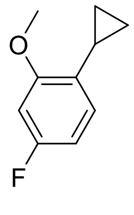

Following the general procedure A, starting from 1-bromo-4-fluoro-2-methoxybenzene (1.03 g, 5.00 mmol, 1.00 equiv), obtained 1-cyclopropyl-4-fluoro-2-methoxybenzene **1r** (540 mg, 3.25 mmol, 65% yield) as a colorless oil. **Rf** = 0.53 (SiO_2, 40:1 pentane:ethyl acetate). **^{1}H NMR** (400 MHz, $CDCl_3$) δ 6.89 – 6.75 (m, 1H, Ar*H*), 6.64 – 6.52 (m, 2H, Ar*H*), 3.85 (s, 3H, OC*H3*), 2.13 – 1.99 (m, 1H, C*H*CH2), 0.97 – 0.83 (m, 2H, CHC*H2*), 0.66 – 0.53 (m, 2H, CHC*H2*). **^{13}C NMR** (101 MHz, $CDCl_3$) δ 161.8 (d, *J* =242.6 Hz), 159.4 (d, *J* =9.5 Hz), 127.5 (d, *J* =3.2 Hz), 125.9 (d, *J* =9.7 Hz), 106.5 (d, *J* =20.9 Hz), 98.7 (d, *J* =25.7 Hz), 55.8, 9.3, 7.4. **^{19}F NMR** (376 MHz, $CDCl_3$) δ -115.4. **HRMS** (APPI/LTQ-Orbitrap) m/z: $[M]^+$ Calcd for $C_{10}H_{11}FO^+$ 166.0788; Found 166.0787

1-methoxy-4-(2-methylcyclopropyl)benzene (1s)

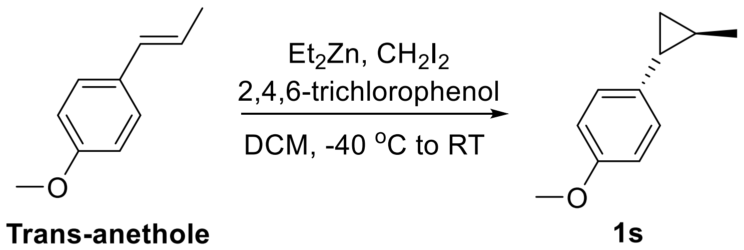

Following reported procedure,[10] in a 250 mL oven-dried round-bottom flask was added 2,4,6-trichlorophenol (2.46 g, 12.50 mmol, 2.5 equiv) under nitrogen atmosphere. DCM (120 mL) was added into the flask and the reaction mixture was cooled to -40 °C. $ZnEt_2$ (1.0 M, 12.5 mL, 12.50 mmol, 2.50 equiv) was added slowly into the flask by syringe and the reaction mixture was stirred at this temperature for 15 minutes. CH_2I_2 (5.29 g, 20.00 mmol, 4.00 equiv) was added slowly by syringe and the reaction mixture was stirred at this temperature for another 15 minutes. Next, the corresponding solution of *trans*-anethole (741.0 mg, 5.00 mmol, 1.00 equiv) in DCM (20 mL) was added by syringe and the reaction mixture was allowed to warm to room temperature and was stirred at this temperature overnight. After the reaction reached completion (as judged by 1H-NMR), the reaction mixture was quenched with sat. NH_4Cl (60 mL) for 30 minutes and extracted with DCM (100 mL) for 3 times. The combined organic layers were washed with aq. NaOH (1.0 M, 60 mL) and brine (40 mL), dried over Na_2SO_4 and filtered. The resulted mixture was concentrated under reduced pressure, the crude residue was purified by column chromatography on Biotage (Büchi flashpure cartridge 40 g, gradient of pentane: ethyl acetate from 99:1 to 95:5) to afford the desired compound 1-methoxy-4-(2-methylcyclopropyl)benzene **1s** (388.76 mg, 2.40 mmol, 48%) as colorless oil.

^{1}**H** NMR (400 MHz, $CDCl_3$) δ 7.01 – 6.96 (m, 2H, ArH), 6.84 – 6.78 (m, 2H, ArH), 3.80 (s, 3H, OCH_3), 1.60 – 1.51 (m, 1H, ArC*H*CH_2), 1.19 (d, *J* =5.9 Hz, 3H, CH_2CHC*H*$_3$), 1.04 – 0.93 (m, 1H, C*H*CH_3) 0.84 – 0.79 (m, 1H, ArCHC*H*$_2$), 0.69 – 0.62 (m, 1H, ArCHC*H*$_2$). ^{13}**C NMR** (101 MHz, $CDCl_3$) δ 157.6, 136.1, 126.7, 113.8, 55.4, 23.5, 19.3, 17.3, 16.9. Consistent with reported data.[10]

Synthesis of 1-(2,2-dimethylcyclopropyl)-4-methoxybenzene (1t)

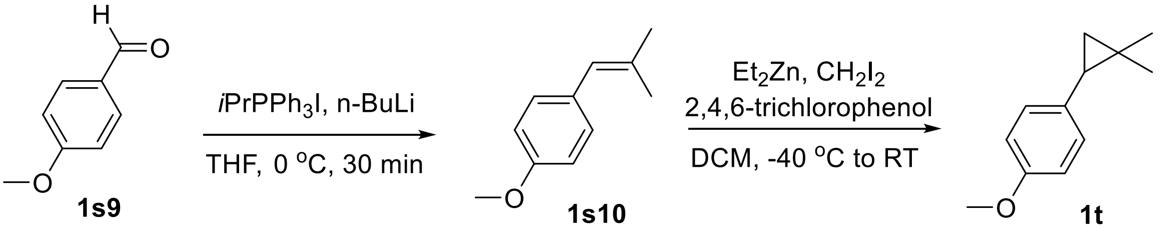

Following reported procedure,[10] to a 250 mL oven-dried round-bottom flask, isopropyltriphenylphosphonium iodide (6.0 g, 14 mmol,1.2 equiv) and anhydrous THF (70 mL) were added. The reaction flaskwas capped with rubber septum and three cycles of evacuate-refill with nitrogen were performed, then the reaction mixture was cooled to 0 °C. *n*-BuLi (2.5 M, 5.6 mL, 14 mmol, 1.2 equiv) was added dropwise by syringe and the reaction mixture was stirred at this temperature for 30 minutes. A solution of aldehyde **1s9** (1577 mg, 11.60 mmol, 1.0 equiv) in THF (20 mL) was added by syringe and the reaction mixture was allowed to warm to room temperature, and then stirred overnight. After the reaction reached completion (as judged by TLC analysis), the reaction mixture was quenched by sat. NH_4Cl (30 mL) and extracted with EtOAc (100mL) for 3 times. The combined organic layers were washed with H_2O_2 (10 wt% in water, 10 mL) and brine (20 mL), dried over Na_2SO_4, and filtered. After that, the mixture was concentrated under reduced pressure, the crude residue was purified by column chromatography (Pentant:EtOAc = 50:1 to 10:1) to afford the desired alkene **1s10** as colorless oil.

Following reported procedure,[10] to a 150 mL oven-dried round-bottom flask, was added 2,4,6-trichlorophenol (1.23 g, 6.25 mmol, 2.5 equiv) under nitrogen atmosphere. DCM (60 mL) was added into the flask and the reaction mixture was cooled to -40 °C. $ZnEt_2$ (1.0 M, 6.25 mL, 2.5 equiv) was added slowly into the flask by syringe and the reaction mixture was stirred at this temperature for 15 minutes. CH_2I_2 (2.39 g, 10.0 mmol, 4.0 equiv) was added slowly by syringe and the reaction mixture was stirred at this temperature for another 15 minutes. Next, a

solution of alkene **1s10** (405 mg, 2.50 mmol, 1.0 equiv) in DCM (10 mL) was added by syringe and the reaction mixture was allowed to warm to room temperature and was stirred at this temperature overnight. After the reaction reached completion, the reaction mixture was quenched with sat. NH_4Cl (30 mL) in 30 minutes and extracted with DCM (50 mL) for 3 times. The combined organic layers were washed with aq. NaOH (1.0 M, 30 mL) and brine, dried over Na_2SO_4 and filtered. After the volatile materials were removed under reduced pressure, the crude residue was purified by column chromatography on Biotage (Büchi flashpure cartridge 40 g, gradient of pentane: ethyl acetate from 99:1 to 95:5) to afford 1-(2,2-dimethylcyclopropyl)-4-methoxybenzene (**1t**) (228.82 mg, 1.30 mmol, 52%) as colorless oil
^{1}H NMR (400 MHz, $CDCl_3$) δ 7.1 – 7.0 (m, 2H), 6.9 – 6.8 (m, 2H), 3.8 (d, *J* =1.6 Hz, 3H), 1.9 – 1.7 (m, 1H), 1.2 (d, *J* =1.6 Hz, 3H), 0.8 (d, *J* =1.7 Hz, 3H), 0.8 – 0.6 (m, 2H). **^{13}C NMR** (101 MHz, $CDCl_3$) δ 157.7, 132.6, 130.0, 113.4, 55.4, 29.0, 27.5, 20.6, 18.6, 18.5. Consistent with reported data.[10]

N-cyclopropyl-4- methylbenzamide (5a)

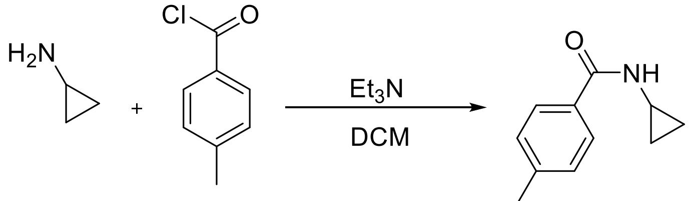

1s11 **1s12** **5a**

Following a reported procedure,[11] to a solution of cyclopropylamine (**1s11**) (0.70 mL, 10 mmol, 1.1 equiv.) and triethylamine (1.40 mL, 10.0 mmol, 1.1 equiv.) in dichloromethane (10 mL) was slowly added a solution of 4-methylbenzoyl chloride (**1s12**) (1.41 g, 9.09 mmol) in dichloromethane (10 mL) at 0 °C. The reaction mixture was stirred at room temperature for 16 hours. Upon completion, the mixture was quenched by the addition of 1 N HCl (10 mL). The aqueous layer was then extracted with dichloromethane. The organic extract was washed with 1 M NaOH (10 mL) and brine (10 mL), dried over Na_2SO_4, filtered and concentrated in vacuo. The crude product **5a** was pure enough to be used as such, without further purification.

^{1}H NMR (400 MHz, $CDCl_3$): δ = 7.72 – 7.55 (m, 2H, Ar*H*), 7.23 – 7.11 (m, 2H, Ar*H*), 6.33 (d, *J* =39.2 Hz, 1H, N*H*), 2.88 (tt, *J* =7.2, 3.5 Hz, 1H, C*H*), 2.37 (d, *J* =3.1 Hz, 3H, C*H₃*), 0.92 – 0.75 (m, 2H, C*H₂*), 0.68 – 0.54 (m, 2H, C*H₂*). Data correspond to the reported values.[11]

Synthesis of hypervalent iodine reagents

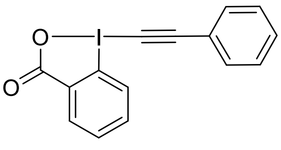

2a

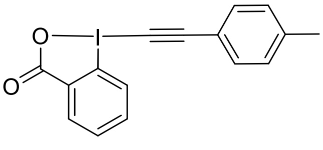

2b

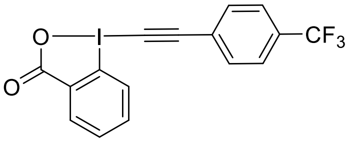

2c

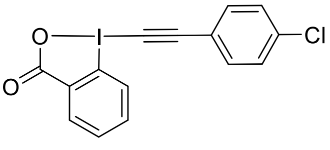

2d

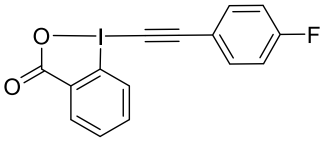

2e

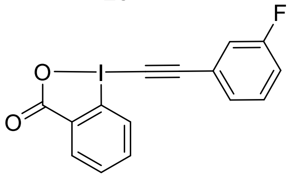

2f

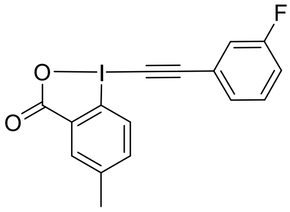

2g

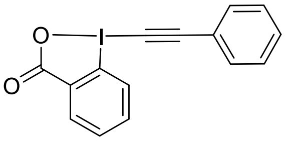

2h

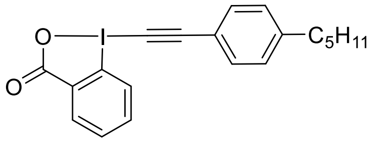

2i

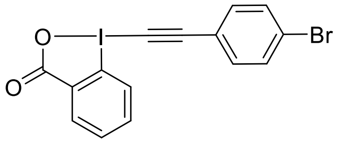

2j

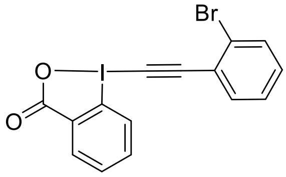

2k

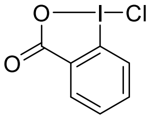

2l

1-Hydroxy-1,2-benziodoxol-3-(1*H*)-one (BIOH, 2s2)

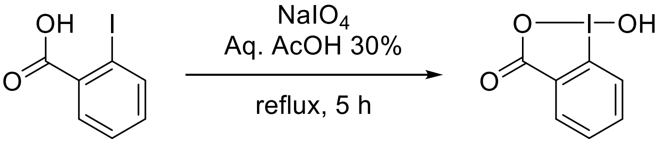

2s1 **2s2**

Following a reported procedure,[12] $NaIO_4$ (40.5 g, 189 mmol, 1.05 equiv) and 2-iodobenzoic acid **(2s1)** (44.8 g, 180 mmol, 1.0 equiv) were suspended in 30% (v:v) aq. AcOH (350 mL). The mixture was vigorously stirred and refluxed for 5 h. The reaction mixture was then diluted with cold water (250 mL) and allowed to cool to rt, protecting it from light. After 1 h, the crude product was collected by filtration, washed on the filter with ice water (3 x 150 mL) and acetone (3 x 150 mL), and air-dried in the dark overnight to afford 1-hydroxy-1,2-benziodoxol-3-(1*H*)-one BIOH (**2s2**) (44.3 g, 168 mmol, 93% yield) as a white solid. **^{1}H NMR** (400 MHz, DMSO-d_6) δ 8.02 (dd, *J* =7.7, 1.4 Hz, 1H, Ar*H*), 7.97 (m, 1H, Ar*H*), 7.85 (dd, *J* =8.2, 0.7 Hz, 1H, Ar*H*), 7.71 (td, *J* =7.6, 1.2 Hz, 1H, Ar*H*). **^{13}C NMR** (100 MHz, DMSO-d_6) δ 167.7, 134.5, 131.5, 131.1, 130.4, 126.3, 120.4. Consistent with reported data.[12]

1-[Phenylethynyl]-1,2-benziodoxol-3(1H)-one (PhEBX) (2a)

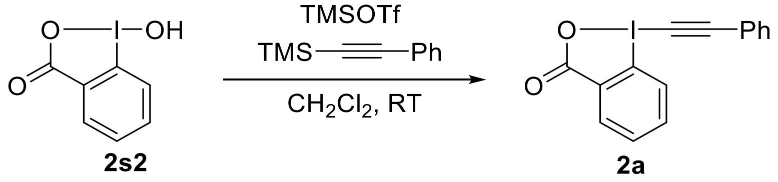

Trimethylsilyltriflate (9.1 mL, 50 mmol, 1.1 equiv) was added dropwise to a suspension of 2-iodosylbenzoic acid (**2s2**) (12.1 g, 45.8 mmol, 1.0 equiv) in CH_2Cl_2 (120 mL) at 0 °C. The mixture was stirred for 1 h, followed by the dropwise addition of trimethyl(phenylethynyl)silane (8.8 mL, 50 mmol, 1.1 equiv) (slightly exothermic). The resulting suspension was stirred for 6 h at RT, during this time a white solid was formed. A saturated solution of $NaHCO_3$ (120 mL) was added and the mixture was stirred vigorously for 30 min. The mixture was extracted with H_2O and DCM (3x50 mL), washed with brine, then dried over Na_2SO_4, filtered and evaporated under reduced pressure. The resulting solid was recrystalized in EtOAc:MeOH (2:1) (40 mL/grams). The solution was left to cool to RT overnight. The slow recrystallization is important to obtain pure product **2a** (photo of crystal obtained below). The crystal formed was filtered and dried under high vacuum to afford PhEBX (**2a**) (5.89 g, 16.95 mmol, 37 % yield) as colorless crystals.**^{1}H NMR** (400 MHz, $CDCl_3$) δ 8.47 – 8.38 (m, 1H, Ar*H*), 8.31 – 8.20 (m, 1H, Ar*H*), 7.83 – 7.72 (m, 2H, Ar*H*), 7.64 – 7.57 (m, 2H, Ar*H*), 7.54 – 7.39 (m, 3H, Ar*H*). **^{13}C NMR** (101 MHz, $CDCl_3$) δ 166.6, 135.1, 133.0, 132.7, 131.8, 131.5, 130.9, 128.9, 126.4, 120.7, 116.3, 106.7, 50.4. Consistent with reported data.[13]

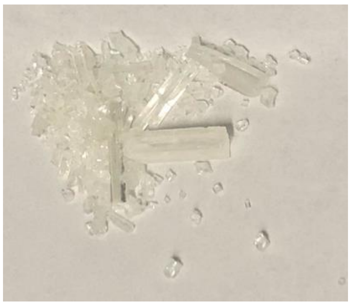

PhEBX with crystallization at RT

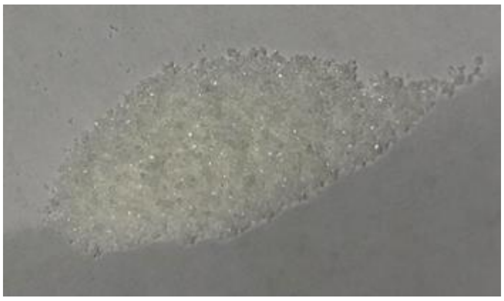

PhEBX with fast crystallization in the fridge

1-(p-Tolylethynyl)-1,2-benziodoxol-3(1H)-one (2b)

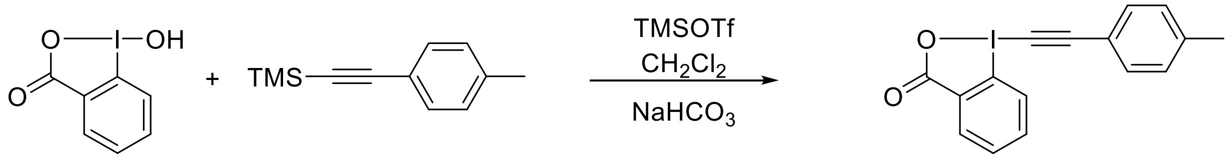

2s2 **2s3** **2b**

Following a reported procedure,[14] trimethylsilyl triflate (1.0 mL, 5.5 mmol, 1.1 equiv) was added to a suspension of 2-iodosylbenzoic acid (**2s2**) (1.32 g, 5.00 mmol, 1.00 equiv) in CH_2Cl_2 (15 mL) at room temperature. The resulting suspension was stirred for 3 h, followed by the drop wise addition of trimethyl(p-tolylethynyl)silane (**2s3**) (1.04 g, 5.50 mmol, 1.10 equiv). The resulting suspension was stirred for 6 h at room temperature. A saturated solution of $NaHCO_3$ (20 mL) was then added and the mixture was stirred vigorously for 30 minutes. The mixture was extracted with H_2O and DCM (3x20 mL), washed with brine, then dried over Na_2SO_4, filtered and evaporated under reduced pressure. The resulting solid was recrystalized in EtOAc:MeOH (2:1) (40 ml/gram). The solution was left to cool to RT overnight.. The crystal formed was filtered and dried under high vacuum to afford **2b** (0.620 g, 1.71 mmol, 34%) as a white crystals. **^{1}H NMR** (400 MHz, $CDCl_3$) δ 8.47 – 8.38 (m, 1H, Ar*H*), 8.30 – 8.20 (m, 1H, Ar*H*), 7.82 – 7.71 (m, 2H, Ar*H*), 7.53 – 7.46 (m, 2H, Ar*H*), 7.28 – 7.20 (m, 2H, Ar*H*), 2.43 (s, 3H, ArC*H*$_3$). **^{13}C NMR** (100 MHz, CDCl3): δ 166.7, 141.7, 135.0, 133.0, 132.6, 131.7, 131.5, 129.7, 126.4, 117.6, 116.4, 107.3, 49.5, 21.9. Consistent with reported data.[14]

1-(*p*-trifluoromethylethynyl)-1,2-benziodoxol-3(1H)-one (2c)

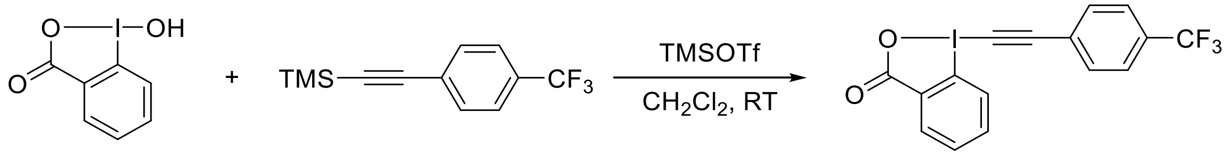

2s2 **2s4** **2c**

Following a reported procedure,[14] trimethylsilyl triflate (1.0 mL, 5.5 mmol, 1.1 equiv) was added to a suspension of BIOH (**2s2**) (1.3 g, 5.0 mmol, 1.0 equiv) in CH_2Cl_2 (15 mL) at RT The resulting suspension was stirred for 1 h, followed by the dropwise addition of trimethyl((4-(trifluoromethyl)phenyl)ethynyl)silane (**2s4**) (1.3 mL, 5.5 mmol, 1.1 equiv), which was dissolved in CH_2Cl_2 (1 mL). The resulting suspension was stirred for 6 h at RT. A saturated solution of $NaHCO_3$ (20 mL) was then added and the mixture was stirred vigorously for 30 min, the two layers were separated and the organic layer was washed with sat. $NaHCO_3$ (20 mL), dried over $MgSO_4$, filtered and evaporated under reduced pressure. The resulting solid

was boiled in CH_3CN (20 mL). The mixture was cooled down, filtered and dried under high vacuum to afford **2c** (1.12 g, 2.69 mmol, 54% yield) as a pale yellow solid. **^{1}H NMR** (400 MHz, $CDCl_3$) δ 8.51 – 8.40 (m, 1H, Ar*H*), 8.29 – 8.18 (m, 1H, Ar*H*), 7.85 – 7.76 (m, 2H, Ar*H*), 7.76 – 7.67 (m, 4H, Ar*H*). **^{13}C NMR** (101 MHz, $CDCl_3$) δ 166.6, 135.0, 133.0, 132.6, 132.2 (q, *J* =33.0 Hz), 131.7, 131.2, 126.3, 125.7 (q, *J* =3.6 Hz), 124.4, 123.4 (q, *J* =272.6 Hz), 116.1, 104.2, 53.7. Consistent with reported data.[14]

1-[4-Chlorophenylethynyl]-1,2-benziodoxol-3(1H)-one (2d)

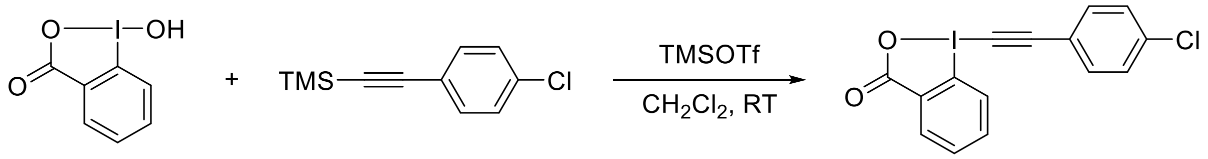

2s2 **2s5** **2d**

Following a reported procedure,[15] Trimethylsilyl triflate (1.0 mL, 5.5 mmol, 1.1 equiv) was added to a suspension of BIOH (**2s2**) (1.32 g, 5.00 mmol, 1.0 equiv) in CH_2Cl_2 (15 mL) at RT. The resulting suspension was stirred for 1 h, followed by the dropwise addition of ((4-Chlorophenyl)ethynyl)trimethylsilane **2s5** (0.68 mL, 5.00 mmol, 1.0 equiv), which was dissolved in CH_2Cl_2 (5 mL). The resulting suspension was stirred for 6 h at RT. A saturated solution of $NaHCO_3$ (20 mL) was then added and the mixture was stirred vigorously for 30 minutes, the two layers were separated. The mixture was extracted with H_2O and DCM (3x20 mL), washed with brine, then dried over Na_2SO_4 filtered and evaporated under reduced pressure. The resulting solid was recrystalized in EtOAc:MeOH (2:1) (40 mL/gram). The solution was left to cool to RT overnight. The crystal formed was filtered and dried under high vacuum to afford **(2d)** as a white solid (658 mg, 1.72 mmol, 34%). **^{1}H NMR** (400 MHz, $CDCl_3$) δ 8.48 – 8.39 (m, 1H, Ar*H*), 8.27 – 8.17 (m, 1H, Ar*H*), 7.84 – 7.69 (m, 2H, Ar*H*), 7.57 – 7.49 (m, 2H, Ar*H*), 7.46 – 7.38 (m, 2H, Ar*H*). **^{13}C NMR** (100 MHz, CDCl3) δ 166.9, 137.4, 135.1, 134.2, 132.8, 131.9, 131.4, 129.4, 126.3, 119.1, 116.3, 105.3, 51.8. Consistent with reported data.[15]

1-[4-Fluorophenylethynyl]-1,2-benziodoxol-3(1H)-one (2e)

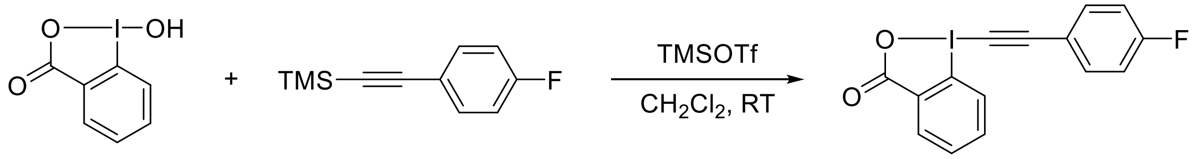

2s2 **2s6** **2e**

Following a reported procedure,[15] Trimethylsilyl triflate (1.0 mL, 5.5 mmol, 1.1 equiv) was added to a suspension of 2-iodosylbenzoic acid (**2s2**) (1.32 g, 5.00 mmol, 1.0 equiv) in CH_2Cl_2 (15 mL) at RT. The resulting suspension was stirred for 1 h, followed by the drop wise addition of ((4-fluorophenyl)ethynyl)trimethylsilane (**2s6**) (1.1 mL, 5.5 mmol, 1.1 equiv), which was dissolved in CH_2Cl_2 (1 mL). The resulting suspension was stirred for 6 h at RT. A saturated solution of $NaHCO_3$ (20 mL) was then added and the mixture was stirred vigorously for 30 minutes, the two layers were separated. The mixture was extracted with H_2O and DCM (3x20 mL), washed with brine, then dried over Na_2SO_4 filtered and evaporated under reduced pressure. The resulting solid was recrystalized in EtOAc:MeOH (2:1) (40 mL/gram). The solution was left to cool to RT overnight. The crystal formed was filtered and dried under high vacuum to afford product **2e** (739 mg, 2.02 mmol, 40%) as a white solid. **^{1}H NMR** (400 MHz, $CDCl_3$) δ 8.49 – 8.38 (m, 1H, Ar*H*), 8.28 – 8.18 (m, 1H, Ar*H*), 7.83 – 7.73 (m, 2H, Ar*H*), 7.65 – 7.55 (m, 2H, Ar*H*), 7.19 – 7.09 (m, 2H, Ar*H*). **^{19}F NMR** (376 MHz, $CDCl_3$) δ -105.85. Consistent with reported data**.**[15]

1-[3-Fluorophenylethynyl]-1,2-benziodoxol-3(1H)-one (2f)

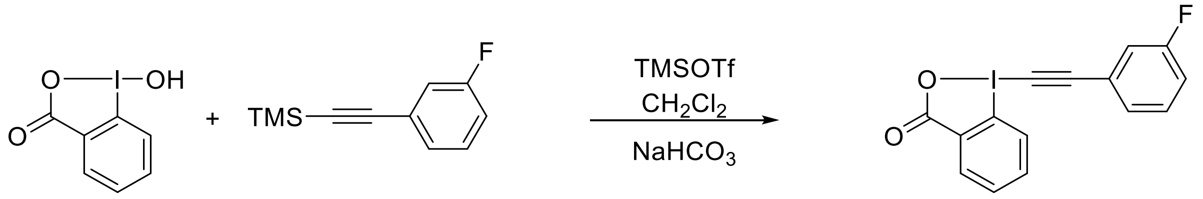

2s2 **2s7** **2f**

Following reported procedure,[14] Trimethylsilyl triflate (0.44 mL, 2.5 mmol, 1.1 equiv) was added to a suspension of 2-iodosylbenzoic acid (**2s2**) (0.589 g, 2.23 mmol, 1.00 equiv) in CH_2Cl_2 (6.8 mL) at RT The resulting suspension was stirred for 1 h, followed by the dropwise addition of ((3-fluorophenyl)ethynyl)trimethylsilane (**2s7**) (0.50 mL, 2.5 mmol, 1.1 equiv). The resulting suspension was stirred for 6 h at RT. A saturated solution of $NaHCO_3$ (10 mL) was then added and the mixture was stirred vigorously for 30 minutes, resulting in a suspension.

The mixture was extracted with H_2O and DCM (3x10 mL), washed with brine, then dried over Na_2SO_4, filtered and evaporated under reduced pressure. The resulting solid was recrystalized in EtOAc:MeOH (2:1) (40 mL/gram). The solution was left to cool to RT and kept in fridge overnight. The crystal formed was filtered and dried under high vacuum to afford product **2f** (384 mg, 1.05 mmol, 47% yield) as colorless crystals. **^{1}H NMR** (400 MHz, DMSO-d_6) δ 8.35 (dd, *J* =8.3, 0.9 Hz, 1H, Ar*H*), 8.14 (dd, *J* =7.4, 1.7 Hz, 1H, Ar*H*), 7.93 (ddd, *J* =8.5, 7.2, 1.7 Hz, 1H, Ar*H*), 7.82 (td, *J* =7.3, 0.9 Hz, 1H, Ar*H*), 7.68 – 7.60 (m, 1H, Ar*H*), 7.63 – 7.52 (m, 2H, Ar*H*), 7.50 – 7.39 (m, 1H, Ar*H*). **^{19}F NMR** (376 MHz, DMSO) δ -111.75. Consistent with reported data.[14]

Synthesis of 2g

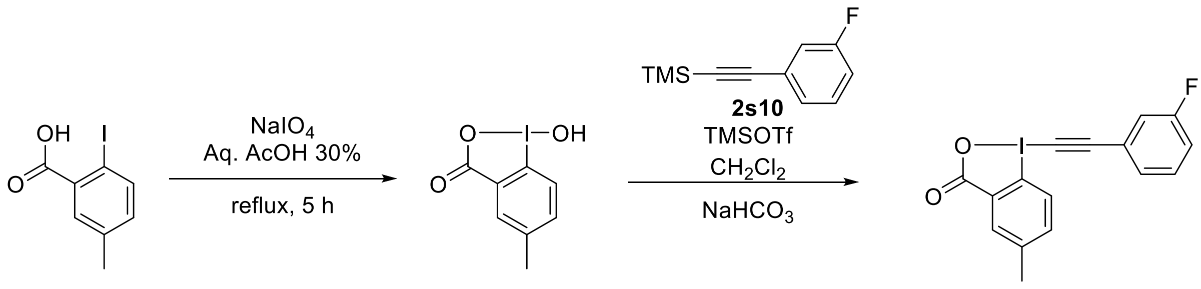

2s8 2s9 2g

Following reported procedure,[14] $NaIO_4$ (4.05 g, 18.9 mmol, 1.05 equiv) and 2-iodo-5-methylbenzoic acid (**2s8**) (4.7 g, 18 mmol, 1.0 equiv) were suspended in 30% (v:v) aq. AcOH (35 mL). The mixture was vigorously stirred and refluxed for 5 h. The reaction mixture was then diluted with cold water (25 mL) and allowed to cool to rt, protecting it from light. After 1 h, the crude product was collected by filtration, washed on the filter with ice water (3 x 15 mL) and acetone (3 x 15 mL), and air-dried in the dark overnight to afford BIOH-a **(2s9)** (3.95 g, 14.2 mmol, 79% yield) as a white solid.

Trimethylsilyl triflate (0.44 mL, 2.5 mmol, 1.1 equiv) was added to a suspension BIOH-a (**2s9**) (0.620 g, 2.23 mmol, 1.00 equiv) in CH_2Cl_2 (6.8 mL) at RT The resulting suspension was stirred for 1 h, followed by the dropwise addition of ((3-fluorophenyl)ethynyl)trimethylsilane (**2s10**) (0.50 mL, 2.5 mmol, 1.1 equiv). The resulting suspension was stirred for 6 h at RT. A saturated solution of $NaHCO_3$ (10 mL) was then added and the mixture was stirred vigorously for 30 minutes, resulting in a suspension. The mixture was extracted with H_2O and DCM (3x10 mL), washed with brine, then dried over Na_2SO_4, filtered and evaporated under reduced pressure. The resulting solid was recrystalized in EtOAc:MeOH (2:1) (40 mL/gram). The solution was left

to cool to RT and kept in fridge overnight. The crystal formed was filtered and dried under high vacuum to afford product **2g** (354 mg, 0.932 mmol, 42% yield) as colorless crystals.

^{1}H NMR (400 MHz, $CDCl_3$) δ 8.23 (d, *J* =2.1 Hz, 1H, Ar*H*), 8.05 (d, *J* =8.5 Hz, 1H, Ar*H*), 7.58 (dd, *J* =8.6, 2.2 Hz, 1H, Ar*H*), 7.45 – 7.35 (m, 2H, Ar*H*), 7.30 – 7.26 (m, 1H, Ar*H*), 7.24 – 7.15 (m, 1H, Ar*H*), 2.51 (s, 3H, ArC*H3*). **^{19}F NMR** (376 MHz, $CDCl_3$) δ -111.26,

^{13}C NMR (101 MHz, $CDCl_3$) δ 166.7, 162.3 (d, *J* =248.6 Hz), 142.7, 136.0, 133.2, 131.0, 130.5 (d, *J* =8.5 Hz), 128.7 (d, *J* =3.3 Hz), 125.9, 122.4 (d, *J* =9.4 Hz), 119.6 (d, *J* =23.2 Hz), 118.2 (d, *J* =21.2 Hz), 112.3, 104.4 (3.3 Hz), 51.8, 20.8.

HRMS (ESI/QTOF) m/z: $[M + H]^+$ Calcd for $C_{16}H_{11}FIO_2^+$ 380.9782; Found 380.9784**.**

Synthesis of 2h

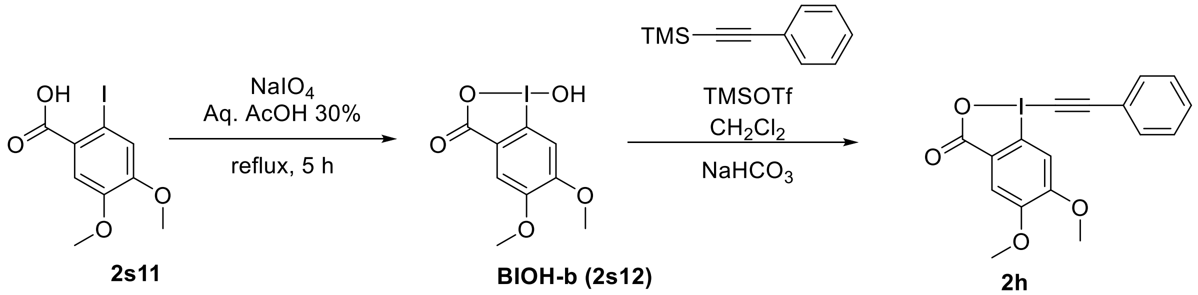

Following reported procedure,[16] $NaIO_4$ (1.25 g, 5.84 mmol, 1.05 equiv) and 2-iodo 4,5-dimethoxybenzoic acid (**2s11**) (1.71 g, 5.56 mmol, 1.00 equiv) were suspended in 30% (v:v) aq. AcOH (15 mL). The mixture was vigorously stirred and refluxed for 4 h. The reaction mixture was then diluted with cold water (40 mL) and allowed to cool to RT, protecting it from light. The crude product was collected by filtration, washed on the filter with ice water (3x4 mL) and acetone (3x4 mL), and air-dried in the dark to give the pure product BIOH-b (**2s12**) (1.51 g, 4.67 mmol, 84%) as a colorless solid. Trimethylsilyl triflate (400 µL, 2.20 mmol, 1.10 equiv) was added to a suspension of BIOH-b (**2s12**) (648 mg, 2.00 mmol, 1.00 equiv) in CH_2Cl_2 (10 mL) at RT. The resulting yellow mixture was stirred for 1 h, followed by the dropwise addition of trimethyl(phenylethynyl)silane (430 µL, 2.20 mmol, 1.10 equiv). The resulting suspension was stirred for 6 h at RT, during this time a yellow suspension was formed. A saturated solution of $NaHCO_3$ (10 mL) was then added. The two layers were separated and the aqueous layer was extracted with DCM (10 mL). The combined organic extracts were dried over $MgSO_4$, filtered and evaporated under reduced pressure. The resulting solid was

recrystallized in CH_3CN (50 mL) and a few EtOH to afford **2h** (306 mg, 0.752 mmol, 38%) as a colorless solid.

^{1}H NMR (400 MHz, $CDCl_3$) δ 7.84 (s, 1 H, Ar*H*), 7.66 (s, 1 H, Ar*H*), 7.61 – 7.53 (m, 2 H, Ar*H*), 7.53 – 7.38 (m, 3 H, Ar*H*), 4.00 (s, 3 H, OC*H₃*), 3.94 (s, 3 H; OC*H₃*). **^{13}C NMR** (101 MHz, CDCl3) δ 167.0, 155.0, 152.3, 132.7, 130.9, 129.0, 124.6, 120.7, 113.4, 107.8, 106.4, 105.5, 56.8, 56.6, 51.4. Consistent with reported data.[16]

1-[4-Pentylphenylethynyl]-1,2-benziodoxol-3(1H)-one (2i)

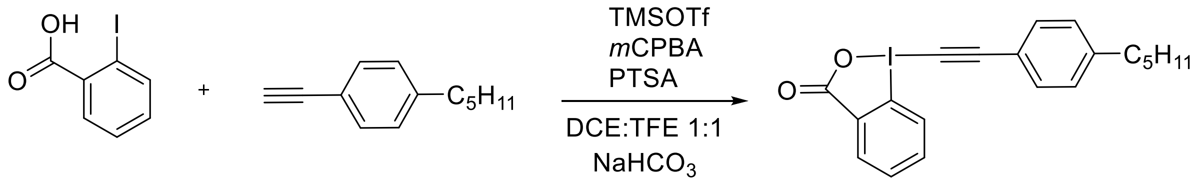

2s1 **2s13** **2i**

Following a reported procedure,[17] in a sealed tube, 2-iodobenzoic acid (**2s1**) (1.00 g, 4.03 mmol, 1.00 equiv), 4-methylbenzenesulfonic acid (775 mg, 4.03 mmol, 1.00 equiv) and mCPBA (994 mg, 4.44 mmol, 1.10 equiv) were suspended in DCE:TFE 1:1 (12 mL) and stirred for 1 h at 55 °C. After 1 h, 1-ethynyl-4-pentylbenzene (**2s13**) (1.1 mL, 5.6 mmol, 1.4 equiv) was added and the reaction was stirred at 55 °C for 24 h. After 24 h, the solvent was evaporated and the residue was redissolved in CH_2Cl_2 (20 mL) and stirred vigorously with $NaHCO_3$ sat. (30 mL). After 1 h, the reaction mixture was transferred into a separating funnel and the layers were separated. The aqueous layer was extracted with CH_2Cl_2 (2x50 mL). The combined organic layers were washed with sat. $NaHCO_3$, dried over MgSO4, filtered and concentrated under vacuum. The resulting solid was boiled in MeCN (20 mL), then filtered and the collected solid was further purified by column chromatography using pure ethyl acetate. Trituration in pentane afforded **2i** (176 mg, 0.421 mmol, 10%) as a pale yellow solid. **^{1}H NMR** (400 MHz, Chloroform-d) δ 8.48 – 8.38 (m, 1H, Ar*H*), 8.30 – 8.20 (m, 1H, Ar*H*), 7.82 – 7.71 (m, 2H, Ar*H*), 7.55 – 7.48 (m, 2H, Ar*H*), 7.26 – 7.23 (m, 2H, Ar*H*), 2.72 – 2.56 (m, 2H, ArC*H₂*), 1.70 – 1.57 (m, 2H, $ArCH_2CH_2$), 1.42 – 1.24 (m, 4H, $CH_2CH_2CH_3$), 0.90 (t, *J* =6.8 Hz, 3H, CH_2CH_3). **^{13}C NMR** (101 MHz, Chloroform-d) δ 166.6, 146.7, 135.0, 133.0, 132.6, 131.7, 131.5, 129.0, 126.3, 117.7, 116.4, 107.4, 49.4, 36.2, 31.5, 31.0, 22.6, 14.1. Consistent with reported data.[17]

1-[4-Bromophenylethynyl]-1,2-benziodoxol-3(1H)-one (2j)

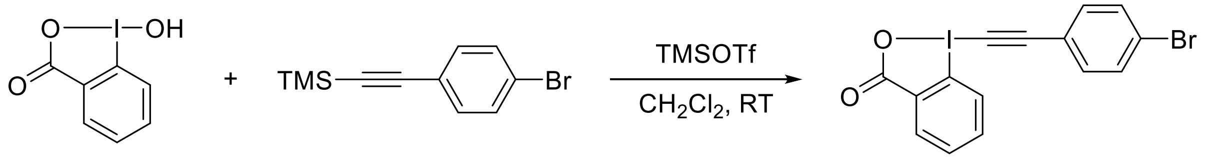

2s2 **2s14** **2j**

Following reported procedure,[12] trimethylsilyl triflate (1.0 mL, 5.5 mmol, 1.1 equiv) was added to a suspension of 2-iodosylbenzoic acid (**2s2**) (1.3 g, 5.0 mmol, 1.0 equiv) in CH_2Cl_2 (15 mL) at RT The resulting suspension was stirred for 1 h, followed by the dropwise addition of ((4 bromophenyl)ethynyl)trimethylsilane (**2s14**) (1.39 g, 5.5 mmol, 1.1 equiv), which was dissolved in CH_2Cl_2 (1 mL). The resulting suspension was stirred for 6 h at RT A saturated solution of $NaHCO_3$ (20 mL) was then added and the mixture was stirred vigorously for 30 min, the two layers were separated and the organic layer was washed with sat. The mixture was extracted with H_2O and DCM (3x20 mL), washed with brine, then dried over Na_2SO_4, filtered and evaporated under reduced pressure. The resulting solid was recrystalized in EtOAc:MeOH (2:1) (40 mL/gram). The solution was left to cool to RT overnight. The crystal formed was filtered and dried under high vacuum to afford product **2j** (1.35 g, 3.15 mmol, 63% yield) as a colorless crystal.**^{1}H NMR** (400 MHz, $CDCl_3$) δ 8.47 – 8.39 (m, 1H, Ar*H*), 8.27 – 8.17 (m, 1H, Ar*H*), 7.84 – 7.73 (m, 2H, Ar*H*), 7.63 – 7.54 (m, 2H, Ar*H*), 7.51 – 7.42 (m, 2H, Ar*H*). **^{13}C NMR** (101 MHz, $CDCl_3$) δ 166.6, 135.1, 134.3, 132.8, 132.3, 131.9, 131.4, 126.3, 125.7, 119.6, 116.3, 105.4, 52.2. Consistent with reported data.[12]

1-[2-Bromophenylethynyl]-1,2-benziodoxol-3(1H)-one (2k)

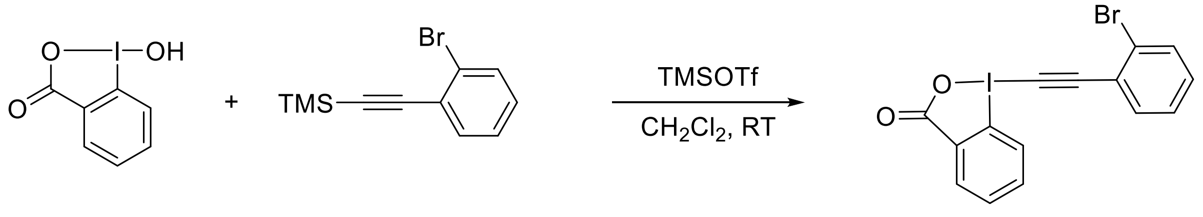

2s2 **2s15** **2k**

Following reported procedure,[12] trimethylsilyl triflate (0.42 mL, 2.4 mmol, 1.1 equiv) was added to a suspension of BIOH (**2s2**) (0.562 g, 2.13 mmol, 1.00 equiv) in CH_2Cl_2 (6 mL) at RT The resulting suspension was stirred for 1 h, followed by the drop wise addition of ((2-bromophenyl)ethynyl)trimethylsilane (**2s15**) (607.2 mg, 2.4 mmol, 1.1 equiv). The resulting

suspension was stirred for 6 h at RT. A saturated solution of $NaHCO_3$ (10 mL) was then added and the mixture was stirred vigorously for 1 h resulting in a persistent emulsion/suspension. The mixture was diluted with $CHCl_3$ (10 mL), water (5 mL) and MeOH (ca. 2 mL) to afford 2 distinct layers. The two layers were separated, and the organic layer was washed with sat. $NaHCO_3$ (5 mL), dried over Na_2SO_4, filtered, and evaporated under reduced pressure. The resulting solid was recrystallized in EtOAc:MeOH (7:3 v:v) (ca. 20 mL). The solution was left to cool to RT then was placed in the freezer (-20 °C) overnight. The crystals were filtered and washed with Et_2O affording **2k** (465 mg, 1.09 mmol, 51% yield) as a pale yellow solid. **^{1}H NMR** (400 MHz, $CDCl_3$) δ 8.50 – 8.39 (m, 2H, Ar*H*), 7.85 – 7.72 (m, 2H, Ar*H*), 7.72 – 7.65 (m, 1H, Ar*H*), 7.65 – 7.58 (m, 1H, Ar*H*), 7.45 – 7.29 (m, 2H, Ar*H*). **^{13}C NMR** (101 MHz, $CDCl_3$) 7 δ 166.6, 135.2, 134.7, 133.0, 132.7, 131.9, 131.8, 131.3, 127.6, 126.8, 126.4, 123.2, 116.5, 104.3, 55.4. Consistent with reported data.[12]

1-Chloro-1,2- benziodoxol-3(1H)-one (2l)

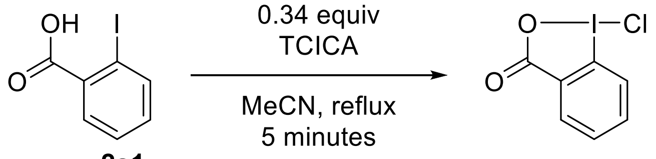

2s1 **2l**

Following reported procedure,[18] a 250 mL three-necked, round-bottom flask equipped with a massive magnetic stirring bar, under Ar, condenser, and dropping funnel. The flask was charged under Ar with solid 2- iodobenzoic acid **2s1** (20.0 g, 0.0790 mol, 1.00 equiv), and anhydrous MeCN (150 mL) was added. The resulting stirred suspension was heated to 75 °C in an oil bath. The dropping funnel was charged with a solution of trichloroisocyanuric acid (6.37 g, 0.027 mol, 1.02 Cl+ equiv) in 30 mL of anhydrous MeCN. The solution of trichloroisocyanuric acid was dropped into the vigorously stirred reaction mixture within 5 min. During the addition of the trichloroisocyanuric acid solution, formation of insoluble isocyanuric acid became apparent. The dropping funnel was rinsed with further anhydrous MeCN (100 mL). After addition was complete, the reaction mixture was refluxed for an additional 5 min. The reaction mixture was vacuum-filtered with a tightly packed pad of Celite (1 cm thick), and the filter cake was rinsed with additional hot MeCN (100−200 mL). The combined filtrates were evaporated, and the resulting yellow solid was filtered and washed with a small amount of cold MeCN. The mother liquor from filtration was partially

concentrated on a rotavap, giving a second crop of crystals. The combined crops were dried for 2 h under high vacuum to give product as free-flowing light yellow crystals (16.70 g, 0.059 mol, 75% yield). **^{1}H NMR** (400 MHz, $CDCl_3$) δ 8.30 – 8.18 (m, 2H), 8.00 (ddd, *J* =8.6, 7.2, 1.7 Hz, 1H), 7.80 (td, *J* =7.4, 0.8 Hz, 1H).Consistent with reported data.[18]

Optimization

In a 12*75 mm Borosilicate glass tube, **PhEBX 2a** was added. The tube was then closed with a rubber septum and sealed off with parafilm. Three cycle of evacuate-refill with nitrogen were performed to remove O_2 and $CHCl_3$ (0.1M) was added, followed by the addition of **1a**. The reaction mixture was stirred at room temperature under irradiation of Kessil lamps (440 nm). It should be noted that chloroform can be used from commercially sealed-cap bottle under inert atmosphere or it is recommended to conduct three cycle of Freeze-Pump-Thaw for other sources of chloroform before use. The reaction was monitored by NMR with CH_2Br_2 as internal standard. NMR yield was determined by integration of ArC*H* NMR of products.

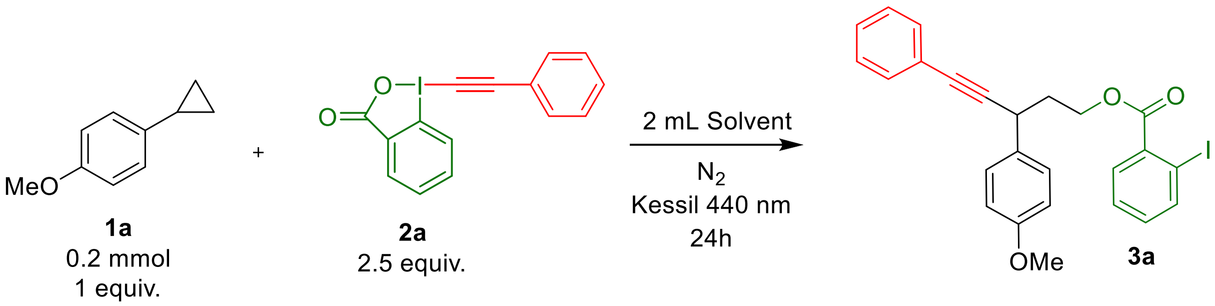

Entry	Variation from standard condition	Conversion (%)	NMR yield (%)
1	$CHCl_3$	100	72
2	DCE	62	20
3	MeOH	64	12
4	CH_3CN/EtOAc	42/19	5/3
5	DMSO or THF	<5	0
6	4 mL $CHCl_3$	100	50
7	1 mL $CHCl_3$	84	63
8	$CHCl_3$, only 1a, without 2a	0	0
9	$CHCl_3$, with 1.5 equiv. of BIOH	100	63
10	$CHCl_3$, with 1.5 equiv. of K_2CO_3	58	24

Results with other hypervalent iodine reagents.

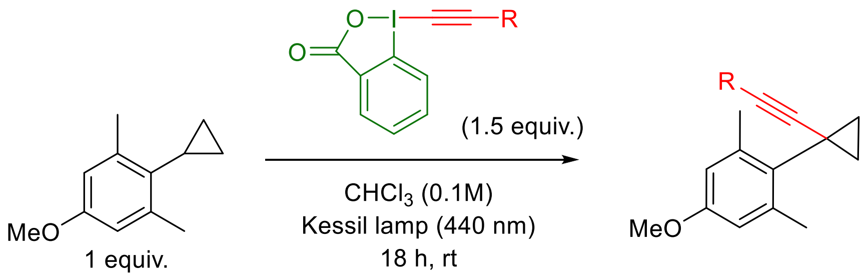

Entry	R	Conversion (%)	NMR yield (%)
1	-TIPS	3	0
2	-CH_2CH_2Cl	5	0
3	-Cyclopropyl	24	21 (product **4s1,** isolated 19%)

Photocatalysis protocol develop by Studer and co-workers (Ref 1j)

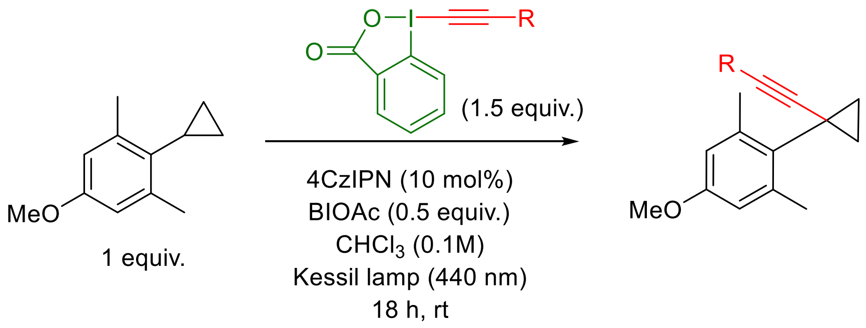

Entry	R	Conversion (%)	NMR yield (%)
1	-TIPS	78	Trace
2	-CH_2CH_2Cl	75	trace
3	-Cyclopropyl	93	20

Synthesis of alkynylated products

General Procedure C (GP C):

In a 12*75 mm Borosilicate glass tube, aryl ethynyl benziodoxolone (0.5 mmol, 2.5 equiv) was added. The tube was then closed with a rubber septum and sealed off with parafilm. Three cycle of evacuate-refill with nitrogen were performed to remove O_2 and $CHCl_3$ (2.0 mL, 0.1M) was added, followed by the addition of starting material (0.2 mmol). The reaction mixture was stirred at room temperature under irradiation of Kessil lamps (440 nm). It should be noted that chloroform can be used from commercially sealed-cap bottle under inert atmosphere or it is recommended to conduct three cycle of Freeze-Pump-Thaw for other sources of chloroform before use. The reaction was monitored by NMR with CH_2Br_2 as internal standard. Upon completion by either full conversion of starting material or hypervalent iodine reagents, the mixture was concentrated in vacuo and purified by on Biotage (Büchi flashpure cartridge 25 g) to obtained the products.

3-(4-Methoxyphenyl)-5-phenylpent-4-yn-1-yl 2-iodobenzoate (3a)

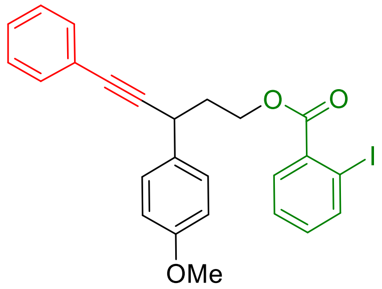

Following the general procedure C, starting from phenyl ethynyl benziodoxolone (**2a**) (174 mg, 500 µmol, 2.50 equiv) and 1-cyclopropyl-4-methoxybenzene (**1a**) (29.6 mg, 200 µmol, 1.00 equiv). The crude mixture was purified by column chromatography on Biotage (Büchi flashpure cartridge 25 g, gradient of Pentane : EtOAc from 5 : 95 to 88 : 12), affording 3-(4-methoxyphenyl)-5-phenylpent-4-yn-1-yl 2-iodobenzoate (**3a**) (67.5 mg, 136 µmol, 68% yield) as colorless oil. **Rf** = 0.29 (SiO_2, 20:1 Pentane/ethyl acetate). **^{1}H NMR** (400 MHz, $CDCl_3$) δ 7.99 (d, *J* =7.9 Hz, 1H, Ar*H*), 7.77 (dd, *J* =7.7, 1.9 Hz, 1H, Ar*H*), 7.44 (td, *J* =4.3, 1.7 Hz, 2H, Ar*H*), 7.40 – 7.34 (m, 3H, Ar*H*), 7.32 – 7.27 (m, 3H, Ar*H*), 7.15 (t, *J* =7.7 Hz, 1H, Ar*H*), 6.92 – 6.86 (m, 2H, Ar*H*), 4.57 (dt, *J* =12.7, 6.6 Hz, 1H, OCH_2CH_2), 4.48 (dt, *J* =11.3, 5.8 Hz, 1H, OCH_2CH_2), 4.11 (t, *J* =7.4 Hz, 1H, C*H*CH_2), 3.81 (s, 3H, OCH_3), 2.29 (q, *J* =6.8 Hz, 2H, OCH_2CH_2). **^{13}C NMR** (101 MHz, $CDCl_3$) δ 166.6, 158.8, 141.4, 135.4, 133.2, 132.8, 131.8, 131.2, 128.6, 128.4, 128.1, 128.0,

123.5, 114.2, 94.2, 90.5, 84.0, 63.8, 55.5, 37.3, 34.7. **IR (ν_{max}, cm^{-1})** 2923 (m), 2852 (w), 2358 (w), 1727 (s), 1677 (m), 1601 (s), 1462 (m), 1292 (s), 1250 (s), 1173 (m), 743 (m). **HRMS** (APCI/QTOF) m/z: $[M + H]^+$ Calcd for $C_{25}H_{22}IO_3^+$ 497.0608; Found 497.0604.

3-(2-Methoxyphenyl)-5-phenylpent-4-yn-1-yl 2-iodobenzoate (3b)

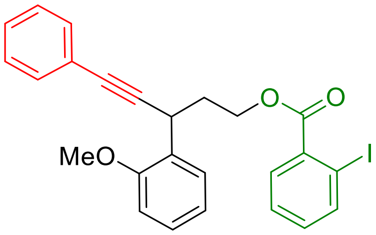

Following the general procedure C, starting from 1-cyclopropyl-2-methoxybenzene (**1o**) (29.6 mg, 200 µmol, 1.00 equiv) and phenyl ethynyl benziodoxolone (**2a**) (174 mg, 500 µmol, 2.50 equiv). The crude mixture was purified by column chromatography on Biotage (Büchi flashpure cartridge 25 g, gradient of Pentane:EtOAc from 2:98 to 90:10) affording 3-(2-methoxyphenyl)-5-phenylpent-4-yn-1-yl 2-iodobenzoate (**3b**) (64.7 mg, 130 µmol, 65% yield) as colorless oil. **Rf** = 0.59 (SiO_2, 10:1 Pentane/ethyl acetate). **^{1}H NMR** (400 MHz, $CDCl_3$) δ 7.95 (dd, *J* =8.0, 1.2 Hz, 1H, Ar*H*), 7.79 (dd, *J* =7.8, 1.7 Hz, 1H, Ar*H*), 7.62 (dd, *J* =7.6, 1.8 Hz, 1H, Ar*H*), 7.44 – 7.38 (m, 2H, Ar*H*), 7.31 (td, *J* =7.6, 1.2 Hz, 1H, Ar*H*), 7.27 – 7.18 (m, 4H, Ar*H*), 7.09 (td, *J* =7.6, 1.8 Hz, 1H, Ar*H*), 6.95 (td, *J* =7.5, 1.1 Hz, 1H, Ar*H*), 6.83 (dd, *J* =8.2, 1.1 Hz, 1H, Ar*H*), 4.56 (dd, *J* =8.7, 5.3 Hz, 1H, C*H*CH_2), 4.53 – 4.43 (m, 2H, OC*H*$_2$$CH_2$), 3.78 (s, 3H, OC*H3*), 2.31 (dtd, *J* =14.0, 7.0, 5.3 Hz, 1H, OCH_2C*H*$_2$), 2.17 (ddt, *J* =14.3, 8.8, 5.8 Hz, 1H, OCH_2C*H*$_2$). **^{13}C NMR** (101 MHz, $CDCl_3$) δ 166.4, 156.3, 141.5, 135.1, 132.7, 131.8, 131.3, 129.4, 128.7, 128.4, 128.3, 128.3, 128.0, 123.7, 121.0, 110.6, 94.3, 90.7, 83.5, 64.1, 55.5, 35.5, 29.0. **IR** (ν_{max}, cm^{-1}) 2925 (w), 2237 (m), 1496 (s), 1487 (m), 1302 (m), 1255 (m), 1234 (m), 1207 (s), 1127 (m), 1114 (m), 1108 (m), 1056 (s), 1046 (m), 755 (s). **HRMS** (ESI/QTOF) m/z: $[M + Na]^+$ Calcd for $C_{25}H_{21}INaO_3^+$ 519.0428; Found 519.0429.

3-(4-Ethoxyphenyl)-5-phenylpent-4-yn-1-yl 2-iodobenzoate (3c)

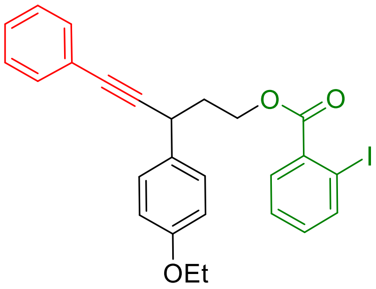

Following the general procedure C, starting from 1-cyclopropyl-4-ethoxybenzene (**1p**) (32.4 mg, 200 µmol, 1.00 equiv) and phenyl ethynyl benziodoxolone (**2a**) (174 mg, 500 µmol, 2.50 equiv.). The crude mixture was purified by column chromatography on Biotage (Büchi flashpure cartridge 25 g, gradient of Pentane:EtOAc from 98:2 to 90:10, affording 3-(4-ethoxyphenyl)-5-phenylpent-4-yn-1-yl 2-iodobenzoate (**3c**) (54.1 mg, 103 µmol, 51% yield) as pale yellow oil. **Rf** = 0.27 (SiO_2, 20:1 Pentane/ethyl acetate). **^{1}H NMR** (400 MHz, $CDCl_3$) δ 7.99 (d, *J* =7.9 Hz, 1H, Ar*H*), 7.77 (dd, *J* =7.8, 1.7 Hz, 1H, Ar*H*), 7.44 (dd, *J* =6.7, 3.0 Hz, 2H, Ar*H*), 7.40 – 7.33 (m, 3H, Ar*H*), 7.33 – 7.27 (m, 3H, Ar*H*), 7.15 (td, *J* =7.7, 1.7 Hz, 1H, Ar*H*), 6.88 (d, *J* =8.5 Hz, 2H, Ar*H*), 4.56 (dt, *J* =11.1, 6.6 Hz, 1H, OCH_2CH_2CH), 4.48 (dt, *J* =11.4, 5.9 Hz, 1H, OCH_2CH_2CH), 4.10 (t, *J* =7.4 Hz, 1H, $CCCHCH_2$), 4.02 (q, *J* =7.0 Hz, 2H, OCH_2CH_3), 2.36 – 2.20 (m, 2H, OCH_2CH_2CH), 1.41 (t, *J* =7.0 Hz, 3H, OCH_2CH_3). **^{13}C NMR** (101 MHz, $CDCl_3$) δ 166.6, 158.2, 141.4, 135.4, 133.0, 132.8, 131.8, 131.2, 128.6, 128.4, 128.1, 128.0, 123.6, 114.8, 94.2, 90.6, 84.0, 63.8, 63.6, 37.4, 34.7, 15.0. **IR** (ν_{max}, cm^{-1}) 2957 (w), 2924 (m), 2227 (w), 1725 (s), 1713 (m), 1508 (m), 1289 (s), 1247 (s), 1205 (m), 1133 (m), 1043 (m), 1013 (m), 758 (m), 741 (m). **HRMS** (ESI/QTOF) m/z: $[M + Na]^+$ Calcd for $C_{26}H_{23}INaO_3^+$ 533.0584; Found 533.0591.

3-(4-(tert-butyl)phenyl)-5-phenylpent-4-yn-1-yl 2-iodobenzoate (3d)

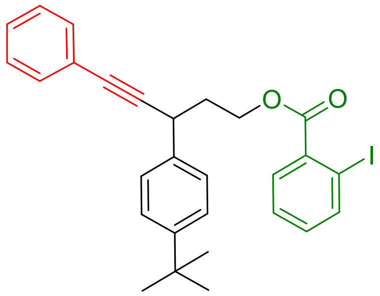

Following the general procedure C, starting from 1-*tert*-butyl-4-cyclopropylbenzene (**1q**) (34.9 mg, 200 µmol, 1.00 equiv), phenyl ethynyl benziodoxolone (**2a**) (139 mg, 400 µmol, 2.00 equiv) and 10-phenyl-9-(2,4,6-trimethylphenyl)acridin-10-ium tetrafluoroborate (1.85 mg, 4.00 µmol, 0.0200 equiv) under kessil lamp 467 nm. The crude mixture was purified by column chromatography on Biotage (Büchi flashpure cartridge 25 g, gradient of Pentane:EtOAc from 99:1 to 92:8) affording 3-(4-(tert-butyl)phenyl)-5-phenylpent-4-yn-1-yl 2-iodobenzoate **(3d)** (53.2 mg, 102 µmol, 51% yield) as pale yellow oil. **R_f:** 0.55 (SiO_2, 20:1 Pentane/ethyl acetate). **^{1}H NMR** (400 MHz, $CDCl_3$) δ 8.00 (dd, *J* =7.9, 1.2 Hz, 1H, Ar*H*), 7.81 (dd, *J* =7.8, 1.7 Hz, 1H, Ar*H*), 7.48 – 7.44 (m, 2H, ArH), 7.41 – 7.36 (m, 5H, Ar*H*), 7.33-7.27 (m, 3H, Ar*H*), 7.15 (td, *J* =7.7, 1.7

Hz, 1H, Ar*H*), 4.60 (ddd, *J* =11.1, 7.4, 6.1 Hz, 1H, OCH_2CH_2), 4.51 (dt, *J* =11.4, 5.9 Hz, 1H, OCH_2CH_2), 4.18 – 4.11 (m, 1H, C*H*CH_2), 2.40 – 2.24 (m, 2H, OCH_2CH_2), 1.33 (s, 9H, C(CH_3)$_3$). **^{13}C NMR** (101 MHz, $CDCl_3$) δ 166.6, 150.1, 142.2, 141.4, 138.0, 135.4, 132.7, 131.8, 131.2, 128.9, 128.3, 128.0, 127.3, 125.8, 125.5, 123.6, 94.1, 90.5, 84.0, 63.9, 37.2, 35.0, 34.6, 31.5. **IR** (ν_{max}, cm^{-1}) 2953 (m), 2868 (w), 2196 (w), 1711 (s), 1289 (s), 1269 (s), 1249 (s), 1228 (m), 1125 (s), 1105 (s), 1014 (s), 752 (s), 741 (s). **HRMS** (ESI/QTOF) m/z: $[M + H]^+$ Calcd for $C_{28}H_{28}IO_2^+$ 523.1129; Found 523.1136.

3,5-Diphenylpent-4-yn-1-yl 2-iodobenzoate (3e)

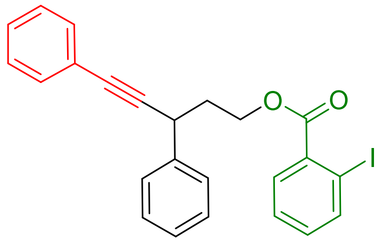

Following the general procedure C, starting from cyclopropylbenzene (23.6 mg, 200 µmol, 1.00 equiv), phenyl ethynyl benziodoxolone (**2a**) (139 mg, 400 µmol, 2.00 equiv) and 10-phenyl-9-(2,4,6-trimethylphenyl)acridin-10-ium tetrafluoroborate (1.85 mg, 4.00 µmol, 0.0200 equiv) under kessil lamp 467 nm. The crude mixture was purified by column chromatography on Biotage (Büchi flashpure cartridge 25 g, gradient of Pentane:EtOAc from 99:1 to 92:8) affording 3,5-diphenylpent-4-yn-1-yl 2-iodobenzoate (**3e**) (37.2 mg, 79.8 µmol, 40% yield) as colorless oil. **R_f:** 0.46 (SiO_2, 20:1 Pentane/ethyl acetate). **^{1}H NMR** (400 MHz, $CDCl_3$) δ 8.00 (dd, *J* =8.0, 1.2 Hz, 1H, Ar*H*), 7.77 (dd, *J* =7.8, 1.7 Hz, 1H, Ar*H*), 7.49 (d, *J* =1.6 Hz, 1H, Ar*H*), 7.47 – 7.43 (m, 3H, Ar*H*), 7.37 (dddd, *J* =7.8, 5.9, 4.8, 1.4 Hz, 3H, Ar*H*), 7.33 – 7.26 (m, 4H, Ar*H*), 7.15 (td, *J* =7.6, 1.7 Hz, 1H, Ar*H*), 4.59 (ddd, *J* =11.2, 7.5, 6.0 Hz, 1H, OCH_2CH_2), 4.50 (dt, *J* =11.3, 5.9 Hz, 1H, OCH_2CH_2), 4.17 (dd, *J* =8.5, 6.3 Hz, 1H, C*H*CH_2), 2.39 – 2.25 (m, 2H, OCH_2CH_2). **^{13}C NMR** (101 MHz, $CDCl_3$): ^{13}C NMR (101 MHz, $CDCl_3$) δ 166.6, 141.4, 141.2, 135.4, 132.8, 131.8, 131.2, 128.9, 128.4, 128.1, 128.0, 127.6, 127.2, 123.5, 94.1, 90.2, 84.2, 63.8, 37.3, 35.5. **IR** (ν_{max}, cm^{-1}) 3061 (w), 2957 (w), 2198 (w), 1711 (s), 1706 (m), 1289 (s), 1271 (s), 1249 (s), 1231 (m), 1133 (s), 1102 (m), 1013 (s), 757 (s), 741 (s), 714 (s). **HRMS** (ESI/QTOF) m/z: $[M + Na]^+$ Calcd for $C_{24}H_{19}INaO_2^+$ 489.0322; Found 489.0331.

3-(4-Methoxy-2-methylphenyl)-5-phenylpent-4-yn-1-yl 2-iodobenzoate (3f)

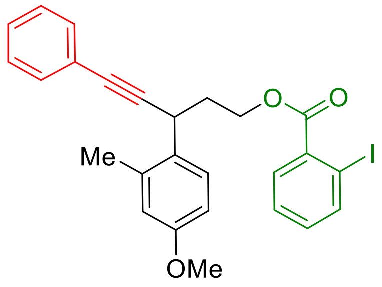

Following the general procedure C, starting from 1-cyclopropyl-4-methoxy-2-methylbenzene **1b** (32.4 mg, 200 µmol, 1.00 equiv) and **PhEBX** (174 mg, 500 µmol, 2.50 equiv), The crude mixture was purified by column chromatography on Biotage (Büchi flashpure cartridge 25 g, gradient of Pentane:EtOAc from 97:3 to 90:10) affording 3-(4-methoxy-2-methylphenyl)-5-phenylpent-4-yn-1-yl 2-iodobenzoate (**3f**) (62.3 mg, 122 µmol, 61% yield) as colorless oil. **Rf** = 0.31 (SiO_2, 20:1 Pentane/ethyl acetate). **^{1}H NMR** (400 MHz, $CDCl_3$) δ 8.00 (dd, *J* =7.9, 1.2 Hz, 1H, Ar*H*), 7.79 (dd, *J* =7.8, 1.7 Hz, 1H, Ar*H*), 7.51 (d, *J* =8.5 Hz, 1H, Ar*H*), 7.46 – 7.41 (m, 2H, Ar*H*), 7.38 (td, *J* =7.6, 1.2 Hz, 1H, Ar*H*), 7.29 (dp, *J* =4.6, 1.7 Hz, 3H, Ar*H*), 7.15 (td, *J* =7.6, 1.7 Hz, 1H, Ar*H*), 6.78 (dd, *J* =8.5, 2.8 Hz, 1H, Ar*H*), 6.72 (d, *J* =2.8 Hz, 1H, Ar*H*), 4.61 (ddd, *J* =11.1, 8.0, 5.7 Hz, 1H, $OC\mathit{H_2}CH_2$), 4.54 (dt, *J* =11.2, 5.6 Hz, 1H, $OC\mathit{H_2}CH_2$), 4.27 (dd, *J* =9.1, 5.6 Hz, 1H, $CC\mathit{H}CH_2$), 3.79 (s, 3H, $OC\mathit{H_3}$), 2.39 (s, 3H, $ArC\mathit{H_3}$), 2.25 (dqd, *J* =14.0, 8.4, 5.6 Hz, 2H, $OCH_2C\mathit{H_2}CH$). **^{13}C NMR** (101 MHz, $CDCl_3$) δ 166.6, 158.5, 141.5, 136.5, 135.3, 134.2, 132.8, 131.8, 131.6, 131.1, 128.8, 128.4, 128.0, 123.6, 116.3, 111.8, 94.2, 90.9, 83.4, 64.0, 55.4, 36.0, 31.4, 19.7. **IR** (ν_{max}, cm^{-1}) 2957 (m), 2363 (w), 1727 (s), 1721 (s), 1609 (m), 1582 (m), 1502 (s), 1288 (s), 1267 (m), 1250 (s), 758 (s), 742 (s). **HRMS** (ESI/QTOF) m/z: $[M + H]^+$ Calcd for $C_{26}H_{24}IO_3^+$ 511.0765; Found 511.0764.

3-(4-Fluoro-2-methoxyphenyl)-5-phenylpent-4-yn-1-yl 2-iodobenzoate (3h)

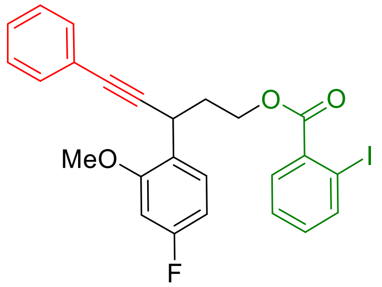

Following the general procedure C, starting from 1-cyclopropyl-4-fluoro-2-methoxybenzene (**1r**) (33.2 mg, 200 µmol, 1.00 equiv) and phenyl ethynyl benziodoxolone (**2a**) (174 mg, 500 µmol, 2.50 equiv). The crude mixture was purified by column chromatography on Biotage (Büchi flash pure cartridge 25 g, gradient of Pentane:EtOAc from 95:5 to 88:12) affording 3-(4-fluoro-2-methoxyphenyl)-5-phenylpent-4-yn-1-yl 2-iodobenzoate (**3h**) (47.1 mg, 91.6 µmol, 46% yield) as pale yellow oil. **Rf** = 0.33 (SiO_2, 10:1 Pentane/ethyl acetate). **^{1}H NMR** (400 MHz, $CDCl_3$) δ 8.00 (dd, *J* =7.9, 1.2 Hz, 1H, Ar*H*), 7.81 (dd, *J* =7.8, 1.7 Hz, 1H, Ar*H*), 7.59 (dd, *J* =8.5, 6.7 Hz, 1H, Ar*H*), 7.49 – 7.42 (m, 2H, Ar*H*), 7.36 (td, *J* =7.6, 1.2 Hz, 1H, Ar*H*), 7.30 (m, 3H, Ar*H*), 7.14 (td, *J* =7.7, 1.7 Hz, 1H, Ar*H*), 6.68 (td, *J* =8.3, 2.5 Hz, 1H, Ar*H*), 6.60 (dd, *J* =10.8, 2.5 Hz, 1H, Ar*H*), 4.60 – 4.45 (m, 3H, OCH_2CH_2 and $CCCHCH_2$), 3.80 (s, 3H, OCH_3), 2.30 (m, 1H, CH_2CH_2CH), 2.18 (m, 1H, CH_2CH_2CH). **^{13}C NMR** δ 166.4, 162.9 (d, *J* =245.1 Hz), 157.3 (d, *J* =9.7 Hz), 141.5, 135.0, 132.8, 131.8, 131.3, 129.7, 129.5 (d, *J* =9.8 Hz), 128.4, 128.0 (d, *J* =17.2 Hz), 125.0 (d, *J* =3.1 Hz), 123.5, 107.1 (d, *J* =21.2 Hz), 99.0 (d, *J* =26.0 Hz), 94.4, 90.4, 83.6, 64.0, 55.8, 35.5, 28.7. **^{19}F NMR** (376 MHz, $CDCl_3$) δ -113.0. **IR** (ν_{max}, cm^{-1}) 2957 (w), 2843 (w), 2227 (w), 1724 (s), 1601 (s), 1496 (s), 1282 (s), 1248 (s), 1151 (s), 1133 (s), 1104 (s), 952 (s), 835 (s), 758 (s), 741 (s). **HRMS** (ESI/QTOF) m/z: $[M + H]^+$ Calcd for $C_{25}H_{21}FIO_3^+$ 515.0514; Found 515.0527.

4-(4-Methoxyphenyl)-6-phenylhex-5-yn-2-yl 2-iodobenzoate (3i)

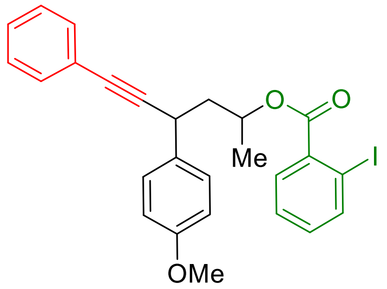

Following the general procedure C, starting from 1-methoxy-4-(2-methylcyclopropyl)benzene (**1s**) (32.4 mg, 200 µmol, 1.00 equiv) and phenyl ethynyl benziodoxolone (**2a**) (174 mg, 500 µmol, 2.50 equiv) The crude mixture was purified by column chromatography on Biotage (Büchi flashpure cartridge 25 g, gradient of Pentane:EtOAc from 95:5 to 90:10) affording 4-(4-methoxyphenyl)-6-phenylhex-5-yn-2-yl 2-iodobenzoate (**3i**) as a colorless oil (mixture of two diastereomers, 71.5 mg, 140 µmol, 70% yield, dr 2:1, the ratio was determined by integration

of the ^{1}H NMR signals for the benzylic protons ArC*H*). **Rf** = 0.3 (SiO_2, 10:1 Pentane/ethyl acetate). **^{1}H NMR** (400 MHz, $CDCl_3$ signals for major diastereomer) δ 7.98 (d, *J* =7.9 Hz, 1H, Ar*H*), 7.76 (dd, *J* =7.7, 1.7 Hz, 1H, Ar*H*), 7.48 – 7.43 (m, 2H, Ar*H*), 7.39 – 7.36 (m, 3H, Ar*H*), 7.29 – 7.27 (m, 3H, Ar*H*), 7.16 – 7.13 (m, 1H, Ar*H*), 6.91 – 6.87 (m, 2H, Ar*H*), 5.54 (dqd, *J* =9.6, 6.2, 3.3 Hz, 1H, OC*H*(CH_3)CH_2), 4.10 (dd, *J* =10.5, 5.0 Hz, 1H, CH_2C*H*CC), 3.80 (s, 3H, OCH_3), 2.27 (ddd, *J* =14.5, 9.7, 5.0 Hz, 1H, CHCH_2CH(CH_3)), 2.10 – 2.05 (m, 1H, , CHCH_2CH(CH_3)), 1.47 (d, *J* =6.2 Hz, 3H,OCHCH_3). **^{13}C NMR** (101 MHz, $CDCl_3$ signals for major diastereomer) δ 166.3, 158.7, 141.3, 136.2, 133.7, 132.5, 131.9, 130.8, 128.7, 128.5, 128.3, 128.0, 123.6, 114.2, 93.9, 90.4, 84.2, 71.5, 55.4, 45.0, 34.7, 20.6. **IR** (ν_{max}, cm^{-1}) 2931 (w), 2836 (w), 2250 (w), 1726 (s), 1705 (m), 1511 (s), 1464 (m), 1287 (s), 1249 (s), 1177 (m), 1130 (s), 1101 (m), 1065 (m), 1038 (s), 1014 (m), 829 (m), 758 (s), 740 (s). **HRMS** (ESI/QTOF) m/z: $[M + Na]^+$ Calcd for $C_{26}H_{23}INaO_3^+$ 533.0584; Found 533.0589.

4-(4-Methoxyphenyl)-2-methyl-6-phenylhex-5-yn-2-yl 2-iodobenzoate (3j)

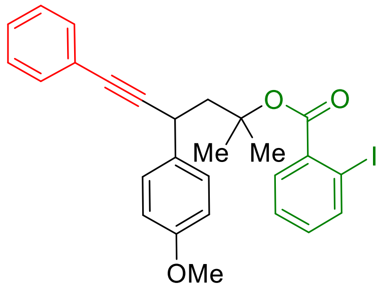

Following the general procedure C, starting from 1-(2,2-dimethylcyclopropyl)-4-methoxybenzene (**1t**) (35.3 mg, 200 µmol, 1.00 equiv) and phenyl ethynyl benziodoxolone (**2a**) (174 mg, 500 µmol, 2.50 equiv). The crude mixture was purified by column chromatography on Biotage (Büchi flashpure cartridge 25 g, gradient of Pentane:EtOAc from 95:5 to 88:12) affording 4-(4-methoxyphenyl)-2-methyl-6-phenylhex-5-yn-2-yl 2-iodobenzoate (**3j**) (56.8 mg, 108 µmol, 54% yield) as colorless oil. **R_f** = 0.33 (SiO_2, 20:1 Pentane/ethyl acetate). **^{1}H NMR :** (400 MHz, $CDCl_3$) δ 7.94 (dd, *J* =7.9, 1.2 Hz, 1H, Ar*H*), 7.69 (dd, *J* =7.8, 1.7 Hz, 1H, Ar*H*), 7.38 (d, *J* =8.7 Hz, 2H, Ar*H*), 7.36 – 7.32 (m, 2H, Ar*H*), 7.31 – 7.26 (m, 3H, Ar*H*), 7.25 (d, *J* =1.7 Hz, 1H, Ar*H*), 7.09 (dd, *J* =7.8, 1.7 Hz, 1H, Ar*H*), 6.89 – 6.84 (m, 2H, Ar*H*), 4.12 – 4.05 (m, 1H, CH_2C*H*CC), 3.79 (s, 3H, OCH_3), 2.54 – 2.46 (m, 2H, OCCH_2CH), 1.82 (s, 3H, CH_2C(CH_3)$_2$), 1.76 (s, 3H, CH_2C(CH_3)$_2$). **^{13}C NMR** (101 MHz, $CDCl_3$) δ 166.0, 158.5, 141.1,

136.7, 134.9, 132.2, 131.6, 131.0, 128.6, 128.3, 127.9, 127.8, 123.8, 114.2, 93.8, 92.5, 84.2, 83.6, 55.4, 49.2, 33.4, 27.2, 26.6. **IR** ($ν_{max}$, cm^{-1}) 2977 (w), 2932 (w), 2835 (w), 2359 (w), 1723 (s), 1610 (w), 1583 (w), 1512 (s), 1299 (s), 1251 (s), 1122 (s), 1103 (m), 758 (m), 741 (s). **HRMS** (ESI/QTOF) m/z: $[M + Na]^+$ Calcd for $C_{27}H_{25}INaO_3^+$ 547.0741; Found 547.0740.

3-(4-Methoxyphenyl)-5-(p-tolyl)pent-4-yn-1-yl 2-iodobenzoate (3k)

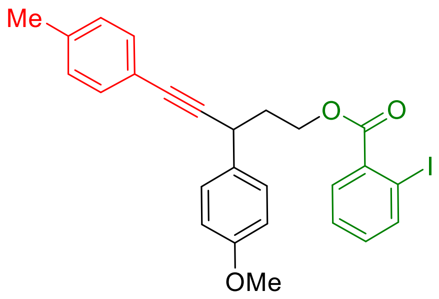

Following the general procedure C, starting from 1-cyclopropyl-4-methoxybenzene (**1a**) (29.6 mg, 200 µmol, 1.00 equiv) and 1-(*p*-tolylethynyl)-1,2-benziodoxol-3(1H)-one (**2b**) (181 mg, 500 µmol, 2.50 equiv) The crude mixture was purified by column chromatography on Biotage (Büchi flashpure cartridge 25 g, gradient of Pentane:EtOAc from 99:5 to 88:12) affording 3-(4-methoxyphenyl)-5-(p-tolyl)pent-4-yn-1-yl 2-iodobenzoate (**3k**) (45.8 mg, 89.7 µmol, 45% yield) as pale yellow oil. $\mathbf{R_f}$ = 0.4 (SiO_2, 10:1 Pentane/ethyl acetate). ^{1}**H NMR** (400 MHz, $CDCl_3$) δ 7.99 (dd, *J* =7.9, 1.2 Hz, 1H, Ar*H*), 7.77 (dd, *J* =7.8, 1.7 Hz, 1H, Ar*H*), 7.41 – 7.35 (m, 3H, Ar*H*), 7.35 – 7.31 (m, 2H, Ar*H*), 7.15 (td, *J* =7.7, 1.7 Hz, 1H, Ar*H*), 7.12 – 7.08 (m, 2H, Ar*H*), 6.93 – 6.86 (m, 2H, Ar*H*), 4.57 (dt, *J* =11.1, 6.6 Hz, 1H, OCH_2CH_2), 4.48 (dt, *J* =11.3, 5.9 Hz, 1H, OCH_2CH_2), 4.10 (dd, *J* =8.1, 6.7 Hz, 1H, $CCCHCH_2$), 3.80 (s, 3H, OCH_3), 2.34 (s, 3H, $ArCH_3$), 2.28 (dtd, *J* =8.0, 6.1, 1.8 Hz, 2H, CH_2CH_2CH). ^{13}**C NMR** (101 MHz, $CDCl_3$) δ 166.6, 158.8, 141.4, 138.1, 135.4, 133.3, 132.7, 131.7, 131.2, 129.1, 128.6, 128.0, 120.5, 114.2, 94.1, 89.7, 84.1, 63.8, 55.4, 37.4, 34.7, 21.6. **IR** ($ν_{max}$, cm^{-1}) 2956 (w), 2923 (w), 1728 (m), 1610 (w), 1584 (w), 1512 (s), 1462 (m), 1288 (m), 1251 (s), 1133 (m), 820 (m), 743 (m). **HRMS** (APPI/LTQ-Orbitrap) m/z: $[M + H]^+$ Calcd for $C_{26}H_{24}IO_3^+$ 511.0765; Found 511.0782.

3-(4-Methoxyphenyl)-5-(4-(trifluoromethyl)phenyl)pent-4-yn-1-yl 2-iodobenzoate (3l)

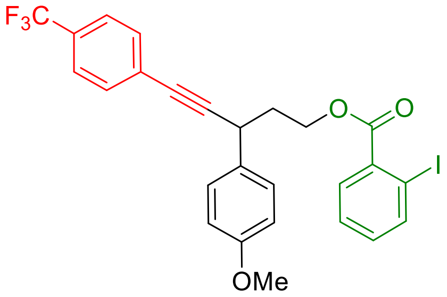

Following the general procedure C, starting from 1-cyclopropyl-4-methoxybenzene (**1a**) (29.6 mg, 200 µmol, 1.00 equiv) and 1-[4-trifluoromethylphenylethynyl]-1,2-benziodoxol-3(1H)-one (**2c**) (208 mg, 500 µmol, 2.50 equiv). The crude mixture was purified by column chromatography on Biotage (Büchi flashpure cartridge 25 g, gradient of Pentane:EtOAc from 97:3 to 88:12) affording 3-(4-methoxyphenyl)-5-(4-(trifluoromethyl)phenyl)pent-4-yn-1-yl 2-iodobenzoate (**3l**) (71.4 mg, 127 µmol, 63% yield) as pale yellow oil. **R_f** = 0.29 (SiO_2, 20:1 Pentane/ethyl acetate). **^{1}H NMR** (400 MHz, $CDCl_3$) δ 8.00 (dd, *J* =8.0, 1.2 Hz, 1H, Ar*H*), 7.76 (dd, *J* =7.8, 1.8 Hz, 1H, Ar*H*), 7.57 – 7.49 (m, 4H, Ar*H*), 7.40 – 7.33 (m, 3H, Ar*H*), 7.16 (dd, *J* =7.7, 1.8 Hz, 1H, Ar*H*), 6.93 – 6.85 (m, 2H, Ar*H*), 4.56 (dt, *J* =11.1, 6.6 Hz, 1H, OCH_2CH_2), 4.47 (dt, *J* =11.4, 5.9 Hz, 1H, OCH_2CH_2), 4.12 (t, *J* =7.4 Hz, 1H, $CCCHCH_2$), 3.81 (s, 3H, OCH_3), 2.40 – 2.25 (m, 2H, CH_2CH_2CH). **^{13}C NMR** (101 MHz, $CDCl_3$) δ 166.6, 158.9, 141.5, 135.3, 132.8, 132.6, 132.1, 131.2, 130.0, 129.7, 128.6, 128.0, 127.4, 125.3 (q, *J* =3.8 Hz), 114.4, 94.2, 93.4, 82.7, 63.6, 55.5, 37.1, 34.7. **^{19}F NMR** (377 MHz, $CDCl_3$) δ -62.8. **IR** ((ν_{max}, cm^{-1}) 2956 (w), 2932 (w), 2838 (w), 2233 (w), 1511 (m), 1323 (s), 1251 (s), 1176 (m), 1167 (m), 1127 (s), 1105 (m), 1067 (m). **HRMS** (ESI/QTOF) m/z: $[M + H]^+$ Calcd for $C_{26}H_{21}F_3IO_3^+$ 565.0482; Found 565.0477.

5-(4-Chlorophenyl)-3-(4-methoxyphenyl)pent-4-yn-1-yl 2-iodobenzoate (3m)

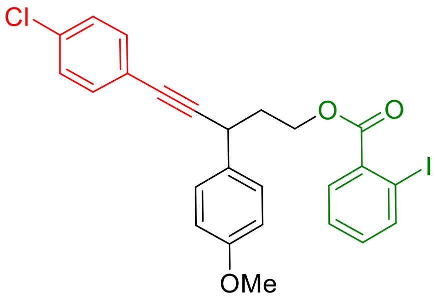

Following the general procedure C, starting from 1-cyclopropyl-4-methoxybenzene (**1a**) (29.6 mg, 200 µmol, 1.00 equiv) and 1-[4-chlorophenylethynyl]-1,2-benziodoxol-3(1H)-one (**2d**) (191 mg, 500 µmol, 2.50 equiv). The crude mixture was purified by column chromatography on Biotage (Büchi flashpure cartridge 25 g, gradient of pentane: ethyl acetate from 95:5 to 85:15) affording 5-(4-chlorophenyl)-3-(4-methoxyphenyl)pent-4-yn-1-yl 2-iodobenzoate (**3m**) (67.2 mg, 127 µmol, 63% yield) as pale yellow oil. **Rf** = 0.2 (SiO_2, 10:1 pentane:ethyl acetate). **^{1}H NMR** (400 MHz, $CDCl_3$) δ 8.00 (dd, *J* =7.9, 1.2 Hz, 1H, Ar*H*), 7.76 (dd, *J* =7.8, 1.7 Hz, 1H, Ar*H*), 7.42 – 7.33 (m, 5H, Ar*H*), 7.27 (s, 1H, Ar*H*), 7.25 (d, *J* =1.7 Hz, 1H, Ar*H*), 7.15 (td, *J* =7.7, 1.7 Hz, 1H, Ar*H*), 6.93 – 6.86 (m, 2H, Ar*H*), 4.55 (dt, *J* =11.1, 6.6 Hz, 1H, OCH_2CH_2), 4.46 (dt, *J* =11.4, 5.9 Hz, 1H, OCH_2CH_2), 4.09 (m, 1H, CCC*H*CH_2), 3.80 (s, 3H, OCH_3), 2.36 – 2.21 (m, 2H, CH_2CH_2CH). **^{13}C NMR** (101 MHz, $CDCl_3$) δ 166.6, 158.8, 141.5, 135.3, 134.0, 133.1, 132.9, 132.8, 131.2, 128.7, 128.6, 128.0, 122.0, 114.3, 94.2, 91.6, 82.9, 63.7, 55.5, 37.2, 34.7. **IR** (ν_{max}, cm^{-1}) 2956 (w), 2925 (w), 2838 (w), 2229 (w), 1725 (m), 1512 (m), 1488 (m), 1289 (s), 1250 (s), 1133 (m), 1094 (m), 1014 (m), 829 (m), 742 (m). **HRMS** (ESI/QTOF) m/z: $[M + H]^+$ Calcd for $C_{25}H_{21}ClIO_3^+$ 531.0218; Found 531.0223.

5-(4-Fluorophenyl)-3-(4-methoxyphenyl)pent-4-yn-1-yl 2-iodobenzoate (3n)

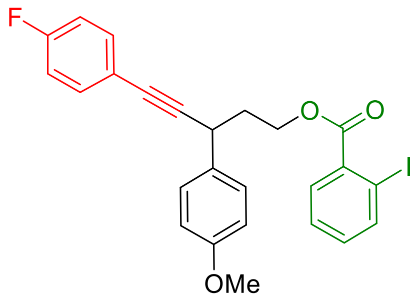

Following the general procedure C, starting from 1-cyclopropyl-4-methoxybenzene (**1a**) (29.6 mg, 200 µmol, 1.00 equiv) and 1-[4-fluorophenylethynyl]-1,2-benziodoxol-3(1H)-one (**2e**) (183 mg, 500 µmol, 2.50 equiv). The crude mixture was purified by column chromatography on Biotage (Büchi flashpure cartridge 25 g, gradient of pentane: ethyl acetate from 95:5 to 85:15) affording 5-(4-fluorophenyl)-3-(4-methoxyphenyl)pent-4-yn-1-yl 2-iodobenzoate **(3n)** (41.4 mg, 80.5 µmol, 40% yield) as pale yellow oil. **Rf** = 0.29 (SiO_2, 20:1 pentane:ethyl acetate). **^{1}H NMR** (400 MHz, $CDCl_3$) δ 8.00 (dd, *J* =8.0, 1.2 Hz, 1H, Ar*H*), 7.76 (dd, *J* =7.8, 1.7 Hz, 1H, Ar*H*), 7.42 – 7.34 (m, 5H, Ar*H*), 7.15 (td, *J* =7.7, 1.7 Hz, 1H, Ar*H*), 6.98 (t, *J* =8.7 Hz, 2H, Ar*H*), 6.92 – 6.85 (m, 2H, Ar*H*), 4.55 (dt, *J* =11.3, 6.6 Hz, 1H, OCH_2CH_2), 4.46 (dt, *J* =11.3, 5.9 Hz, 1H,

O$CH_2$$CH_2$), 4.09 (t, *J* =7.4 Hz, 1H, CCC*H*CH_2), 3.80 (s, 3H, OC*H₃*), 2.34 – 2.23 (m, 2H, CH_2C*H₂*CH). **^{13}C NMR** (201 MHz, $CDCl_3$) δ 166.6, 162.4 (d, *J* =248.8 Hz), 158.8, 141.5, 135.4, 133.7 (d, *J* =8.4 Hz), 133.1, 132.8, 131.2, 128.6, 128.0, 119.6, 115.6 (d, *J* =22.2 Hz), 114.3, 94.2, 90.2, 82.9, 63.8, 55.5, 37.3, 34.6. **^{19}F NMR** (377 MHz, $CDCl_3$) δ -111.6. **IR** (ν_{max}, cm^{-1}) 2959 (m), 2929 (w), 2228 (w), 2150 (w), 1721 (m), 1600 (m), 1505 (s), 1288 (s), 1265 (s), 1255 (s), 1249 (s), 1231 (s), 1094 (m), 1030 (m), 1015 (s), 837 (s), 740 (s). **HRMS** (APCI/QTOF) m/z: $[M + H]^+$ Calcd for $C_{25}H_{21}FIO_3^+$ 515.0514; Found 515.0515.

5-(3-Fluorophenyl)-3-(4-methoxyphenyl)pent-4-yn-1-yl 2-iodobenzoate (3o)

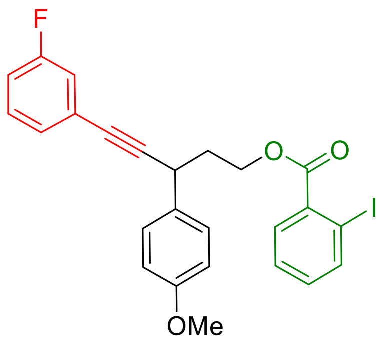

Following the general procedure C, starting from 1-cyclopropyl-4-methoxybenzene (**1a**) (29.6 mg, 200 µmol, 1.00 equiv) and 1-[3-fluorophenylethynyl]-1,2-benziodoxol-3(1H)-one (**2f**) (183 mg, 500 µmol, 2.50 equiv). The crude mixture was purified by column chromatography on Biotage (Büchi flashpure cartridge 25 g, gradient of pentane: ethyl acetate from 95:5 to 85:15) affording 5-(3-fluorophenyl)-3-(4-methoxyphenyl)pent-4-yn-1-yl 2-iodobenzoate (**3o**) (28.2 mg, 54.8 µmol, 27% yield) as pale yellow oil. **Rf** = 0.26 (SiO_2, 10:1 pentane:ethyl acetate).

^{1}H NMR (400 MHz, $CDCl_3$) δ 8.00 (dd, *J* =7.9, 1.2 Hz, 1H, Ar*H*), 7.77 (dd, *J* =7.8, 1.7 Hz, 1H, Ar*H*), 7.41 – 7.33 (m, 3H, Ar*H*), 7.26 – 7.19 (m, 2H, Ar*H*), 7.18 – 7.10 (m, 2H, Ar*H*), 7.03 – 6.96 (m, 1H, Ar*H*), 6.93 – 6.87 (m, 2H, Ar*H*), 4.59 – 4.51 (m, 1H, OC*H₂*CH_2), 4.50 – 4.42 (m, 1H, OC*H₂*CH_2), 4.10 (t, *J* =7.4 Hz, 1H, CCC*H*CH_2), 3.81 (s, 3H, OC*H₃*), 2.29 (dt, *J* =7.3, 6.2 Hz, 2H, CH_2C*H₂*CH). **^{13}C NMR** (101 MHz, $CDCl_3$) δ 166.5, 162.3 (d, *J* =246.2 Hz), 158.7, 141.3, 135.2, 134.1, 132.7, 131.1, 129.8 (d, *J* =8.7 Hz), 128.5, 127.9, 127.6 (d, *J* =3.2 Hz), 125.2 (d, *J* =9.5 Hz), 118.5 (d, *J* =22.6 Hz), 115.3 (d, *J* =21.2 Hz), 114.2, 94.0, 91.6, 82.7 (d, *J* =3.4 Hz), 63.5, 55.3, 37.1, 34.5. **^{19}F NMR** (377 MHz, $CDCl_3$) δ -113.2. **IR** (ν_{max}, cm^{-1}) 2931 (w), 2836 (w), 2229 (w), 1726 (m), 1581 (m), 1510 (s), 1288 (s), 1247 (s), 1176 (m), 1015 (m), 785 (m), 740 (s).

HRMS (APCI/QTOF) m/z: $[M + H]^+$ Calcd for $C_{25}H_{21}FIO_3^+$ 515.0514; Found 515.0528.

5-(3-Fluorophenyl)-3-(4-methoxyphenyl)pent-4-yn-1-yl 2-iodo-4-methylbenzoate (3p)

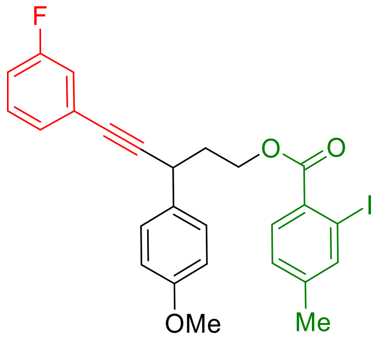

Following the general procedure C, starting from 1-cyclopropyl-4-methoxybenzene (**1a**) (29.6 mg, 200 µmol, 1.00 equiv) and 1-[3-fluorophenylethynyl]-5-methyl-1,2-benziodoxol-3(1H)-one (**2g**) (190 mg, 500 µmol, 2.50 equiv). The crude mixture was purified by column chromatography on Biotage (Büchi flashpure cartridge 25 g, gradient of pentane: ethyl acetate from 95:5 to 85:15) affording 5-(3-fluorophenyl)-3-(4-methoxyphenyl)pent-4-yn-1-yl 2-iodo-4-methylbenzoate (**3p**) (54.3 mg, 103 µmol, 51% yield) as pale yellow oil. **Rf** = 0.32 (SiO_2, 20:1 pentane:ethyl acetate). **^{1}H NMR** (400 MHz, $CDCl_3$) δ 7.84 (d, *J* =8.1 Hz, 1H, Ar*H*), 7.57 (dd, *J* =2.2, 0.8 Hz, 1H, Ar*H*), 7.40 – 7.33 (m, 2H, Ar*H*), 7.26 – 7.18 (m, 2H, Ar*H*), 7.12 (ddd, *J* =9.5, 2.6, 1.4 Hz, 1H, Ar*H*), 6.96 – 7.02 (m, 2H, Ar*H*), 6.92 – 6.88 (m, 2H, Ar*H*), 4.54 (dt, *J* =11.1, 6.7 Hz, 1H, OCH_2CH$_2$), 4.46 (dt, *J* =11.3, 6.0 Hz, 1H, OCH_2CH$_2$), 4.10 (t, *J* =7.4 Hz, 1H, CCC*H*CH$_2$), 3.80 (s, 3H, OCH_3), 2.32 (s, 3H, ArCH_3), 2.32 – 2.26 (m, 2H, CH$_2$CH_2CH). **^{13}C NMR** (101 MHz, $CDCl_3$) δ 166.8, 162.5 (d, *J* =246.2 Hz), 158.8, 141.2, 138.3, 135.2, 133.8, 132.8, 131.9, 129.9 (d, *J* =8.6 Hz), 128.6, 127.7 (d, *J* =3.0 Hz), 125.4 (d, *J* =9.4 Hz), 118.6 (d, *J* =22.7 Hz), 115.4 (d, *J* =21.3 Hz), 114.3, 91.7, 90.0, 82.8 (d, *J* =3.3 Hz), 63.6, 55.4, 37.2, 34.6, 20.9. ^{19}F NMR (377 MHz, $CDCl_3$) δ -113.2. **IR** (ν_{max}, cm^{-1}) 2958 (w), 2836 (w), 2363 (w), 2232 (w), 1724 (s), 1611 (m), 1579 (m), 1512 (s), 1294 (s), 1249 (s), 1203 (s), 1173 (m), 1108 (m), 1036 (m), 1015 (m), 779 (m). **HRMS** (APCI/QTOF) m/z: $[M + H]^+$ Calcd for $C_{26}H_{23}FIO_3^+$ 529.0670; Found 529.0679.

3-(4-Methoxyphenyl)-5-phenylpent-4-yn-1-yl 2-iodo-4,5-dimethoxybenzoate (3q)

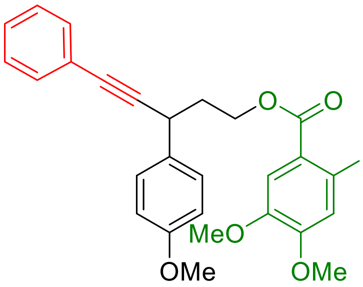

Following the general procedure C, starting from 1-cyclopropyl-4-methoxybenzene (**1a**) (29.6 mg, 200 μmol, 1.00 equiv) and 4,5-dimethoxy-1-[phenylethynyl]-1,2-benziodoxol-3(1H)-one (**2h**) (204 mg, 500 μmol, 2.50 equiv). The crude mixture was purified by column chromatography on Biotage (Büchi flashpure cartridge 25 g, gradient of pentane: ethyl acetate from 85:15 to 70:30) affording 3-(4-methoxyphenyl)-5-phenylpent-4-yn-1-yl 2-iodo-4,5-dimethoxybenzoate **(3q)** (33.7 mg, 60.6 μmol, 30% yield) as colorless oil. **Rf** = 0.14 (SiO_2, 10:1 pentane:ethyl acetate). **^{1}H NMR** (400 MHz, $CDCl_3$) δ 7.46 – 7.41 (m, 3H, Ar*H*), 7.38 (d, *J* =8.3 Hz, 3H, Ar*H*), 7.32 – 7.27 (m, 3H, Ar*H*), 6.92 – 6.86 (m, 2H, Ar*H*), 4.61 – 4.51 (m, 1H, OCH_2CH_2), 4.51 – 4.43 (m, 1H, OCH_2CH_2), 4.11 (dd, *J* =8.2, 6.6 Hz, 1H, $CCCHCH_2$), 3.92 (s, 3H, OCH_3), 3.88 (s, 3H, OCH_3), 3.80 (s, 3H, OCH_3), 2.35 – 2.22 (m, 2H, CH_2CH_2CH). **^{13}C NMR** (201 MHz, $CDCl_3$) δ 165.7, 158.8, 152.0, 148.8, 133.2, 131.8, 128.6, 128.4, 128.1, 126.6, 123.8, 123.5, 114.2, 114.2, 90.6, 84.6, 84.0, 63.7, 56.4, 56.2, 55.5, 37.4, 34.7. **IR** (ν_{max}, cm^{-1}) 2960 (m), 2924 (m), 2849 (w), 1720 (m), 1510 (s), 1261 (s), 1246 (s), 1204 (s), 1174 (s), 1111 (m), 1024 (s), 795 (m). **HRMS** (ESI/QTOF) m/z: $[M + Na]^+$ Calcd for $C_{27}H_{25}INaO_5^+$ 579.0639; Found 579.0658.

5-Methoxy-1,3-dimethyl-2-(1-(phenylethynyl)cyclopropyl)benzene (4a)

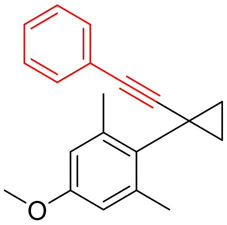

Following the general procedure C, starting from **1c** (35.3 mg, 200 μmol, 1.00 equiv) and **2a** (104 mg, 300 μmol, 1.50 equiv). The crude mixture was purified by column chromatography on Biotage (Büchi flashpure cartridge 25 g, gradient of pentane: ethyl acetate from 99:1 to

95:5) affording (**4a**) (46.1 mg, 167 µmol, 83% yield) as white solid. **MP**: 60 - 62°C. **Rf** = 0.55 (SiO_2, 40:1 pentane:ethyl acetate). **^{1}H NMR** (400 MHz, $CDCl_3$) δ 7.37 – 7.31 (m, 2H, Ar*H*), 7.23 (m, 3H, Ar*H*), 6.59 (s, 2H, Ar*H*), 3.78 (s, 3H, OC*H₃*), 2.53 (s, 6H, 2xArC*H₃*), 1.55 – 1.49 (m, 2H, $CH^aH^bCH^aH^b$), 1.17 – 1.09 (m, 2H, $CH^aH^bCH^aH^b$). **^{13}C NMR** (101 MHz, $CDCl_3$) δ 158.3, 140.1, 131.8, 131.1, 128.2, 127.4, 124.2, 113.6, 94.5, 75.6, 55.2, 20.9, 19.8, 13.0. **IR** (ν_{max}, cm^{-1}) 3005 (w), 2955 (w), 2922 (w), 2836 (w), 2226 (w), 1597 (m), 1484 (m), 1329 (m), 1313 (m), 1158 (s), 1058 (m), 756 (s). **HRMS** (ESI/QTOF) m/z: $[M + H]^+$ Calcd for $C_{20}H_{21}O^+$ 277.1587; Found 277.1587.

***Scale-up experiment**

In a schlenk tube 50 ml, **2a** (3 mmol, 1.5 equiv) was added. The tube was then closed with a rubber septum and sealed off with parafilm. Three cycles of evacuate-refill with nitrogen were performed to remove O_2 and $CHCl_3$ (20 mL) was added, followed by three cycles of Freeze Pump Thaw to completely remove O_2. After that, **1c** (353 mg, 2.00 mmol, 1.00 equiv) was added under N_2 atmosphere and the top of the schlenk tube was sealed again with parafilm. The reaction was monitored by NMR with CH_2Br_2 as internal standard. Upon complete conversion of the starting material, the mixture was concentrated in vacuo and purified by column chromatography on Biotage (Büchi flashpure cartridge 40 g, gradient of pentane: ethyl acetate from 99:1 to 95:5) to give product **4a** (511 mg, 1.85 mmol, 92% yield).

5-Methoxy-1,3-dimethyl-2-(1-(p-tolylethynyl)cyclopropyl)benzene (4b)

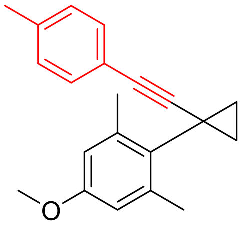

Following the general procedure C, starting from **1c** (35.3 mg, 200 µmol, 1.00 equiv) and 1-[tolylethynyl]-1,2-benziodoxol-3(1H)-one (**2b**) (109 mg, 300 µmol, 1.50 equiv). The crude mixture was purified by column chromatography on Biotage (Büchi flashpure cartridge 25 g, gradient of pentane: ethyl acetate from 99:1 to 95:5) affording **4b** (37.9 mg, 131 µmol, 65% yield) as pale yellow oil. **Rf** = 0.6 (SiO_2, 20:1 pentane:ethyl acetate). **^{1}H NMR :** ^{1}H NMR (400 MHz, $CDCl_3$) δ 7.22 (d, *J* =8.0 Hz, 2H, Ar*H*), 7.04 (d, *J* =8.0 Hz, 2H, Ar*H*), 6.58 (s, 2H, Ar*H*), 3.77

(s, 3H, OCH_3), 2.53 (s, 6H, $ArCH_3$), 2.31 (s, 3H), 1.51 (m, 2H, $CH^aH^bCH^aH^b$), 1.13 – 1.05 (m, 2H, $CH^aH^bCH^aH^b$). **^{13}C NMR** ^{13}C NMR (101 MHz, $CDCl_3$) δ 158.3, 140.1, 137.4, 131.7, 131.3, 128.9, 121.1, 113.6, 93.7, 75.6, 55.2, 21.5, 20.9, 19.8, 13.1. **IR** (film): IR (ν_{max}, cm^{-1}) 2921 (m), 2838 (w), 2226 (w), 1604 (s), 1507 (m), 1484 (m), 1468 (m), 1462 (m), 1327 (s), 1311 (s), 1193 (m), 1157 (s), 1057 (m), 818 (s). **HRMS** (APPI/LTQ-Orbitrap) m/z: $[M]^+$ Calcd for $C_{21}H_{22}O^+$ 290.1665; Found 290.1671.

5-Methoxy-1,3-dimethyl-2-(1-((4-pentylphenyl)ethynyl)cyclopropyl)benzene (4c)

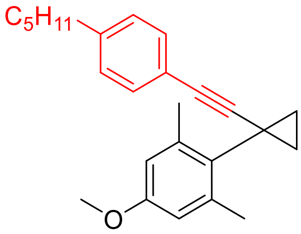

Following the general procedure C, starting from **1c** (35.3 mg, 200 µmol, 1.00 equiv) and 1-[4-*n*-pentylphenylethynyl]-1,2-benziodoxol-3(1H)-one (**2i**) (125 mg, 300 µmol, 1.50 equiv). The crude mixture was purified by column chromatography on Biotage (Büchi flashpure cartridge 25 g, gradient of pentane: ethyl acetate from 99:1 to 95:5) affording **4c** (27.9 mg, 80.5 µmol, 40% yield) as pale yellow oil. **Rf** = 0.57 (SiO_2, 40:1 pentane:ethyl acetate). **^{1}H NMR :** ^{1}H NMR (400 MHz, $CDCl_3$) δ 7.26 – 7.20 (m, 2H, Ar*H*), 7.07 – 7.01 (m, 2H, Ar*H*), 6.58 (s, 2H, Ar*H*), 3.77 (s, 3H, OCH_3), 2.57 – 2.53 (m, 2H, $ArCH_2CH_2$), 2.52 (s, 6H, $ArCH_3$) 1.61 – 1.54 (m, 2H, $ArCH_2CH_2$), 1.53 – 1.47 (m, 2H, $CH^aH^bCH^aH^b$), 1.35 – 1.23 (m, 4H, $ArC_2H_4C_2H_4$), 1.13 – 1.03 (m, 2H, $CH^aH^bCH^aH^b$), 0.87 (t, J =7.0 Hz, 3H, $ArC_4H_8CH_3$). **^{13}C NMR** : ^{13}C NMR (101 MHz, $CDCl_3$) δ 158.3, 142.5, 140.1, 131.7, 131.3, 128.3, 121.3, 113.5, 93.7, 75.6, 55.2, 35.9, 31.5, 31.1, 22.6, 20.9, 19.8, 14.1, 13.1. **IR** (film): IR (ν_{max}, cm^{-1}) 2957 (s), 2924 (s), 2856 (m), 2224 (w), 1604 (s), 1508 (s), 1485 (s), 1462 (m), 1328 (s), 1312 (s), 1155 (s), 1061 (s), 855 (m), 835 (s). **HRMS** (APPI/LTQ-Orbitrap) m/z: $[M]^+$ Calcd for $C_{25}H_{30}O^+$ 346.2291; Found 346.2304.

5-Methoxy-1,3-dimethyl-2-(1-((4-(trifluoromethyl)phenyl)ethynyl)cyclopropyl)benzene (4d)

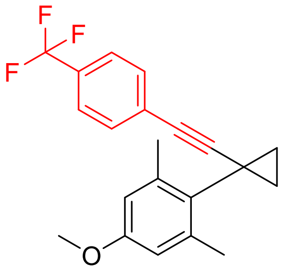

Following the general procedure C, starting from **1c** (35.3 mg, 200 µmol, 1.00 equiv) and 1-[4-trifluoromethylphenylethynyl]-1,2-benziodoxol-3(1H)-one **2c** (124 mg, 300 µmol, 1.50 equiv). The crude mixture was purified by column chromatography on Biotage (Büchi flashpure cartridge 25 g, gradient of pentane: ethyl acetate from 99:1 to 95:5) affording **4d** (55.8 mg, 162 µmol, 81% yield) as pale yellow oil.

Rf = 0.58 (SiO_2, 40:1 pentane:ethyl acetate). **^{1}H NMR** (400 MHz, $CDCl_3$) δ 7.59 – 7.35 (m, 4H, Ar*H*), 6.60 (s, 2H, Ar*H*), 3.78 (s, 3H, OC*H*$_3$), 2.53 (s, 6H, ArC*H*$_3$), 1.61 – 1.53 (m, 2H, CH^aH^{b}CH^aH^b), 1.20 – 1.09 (m, 2H, CH^aH^bCH^aH^b). **^{13}C NMR** (101 MHz, $CDCl_3$) δ 158.5, 140.1, 132.0, 130.6, 129.3, 128.1, 127.4, 125.1 (q, *J* =3.8 Hz), 113.7, 97.4, 74.5, 55.2, 20.9, 20.0, 13.0. **^{19}F NMR (**377 MHz, $CDCl_3$) δ -62.7. **IR** (film): IR (ν_{max}, cm^{-1}) 2956 (w), 2925 (w), 2838 (w), 2226 (w), 2118 (w), 1604 (m), 1487 (m), 1322 (s), 1159 (s), 1126 (s), 1066 (s), 842 (m). **HRMS** (APPI/LTQ-Orbitrap) m/z: $[M]^+$ Calcd for $C_{21}H_{19}F_3O^+$ 344.1383; Found 344.1386.

2-(1-((4-Fluorophenyl)ethynyl)cyclopropyl)-5-methoxy-1,3-dimethylbenzene (4e)

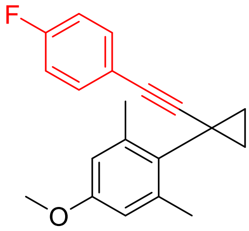

Following the general procedure C, starting from **1c** (35.3 mg, 200 µmol, 1.00 equiv) and 1-[4-Fluorophenylethynyl]-1,2-benziodoxol-3(1H)-one (**2e**) (110 mg, 300 µmol, 1.50 equiv). The crude mixture was purified by column chromatography on Biotage (Büchi flashpure cartridge 25 g, gradient of pentane: ethyl acetate from 99:1 to 95:5) affording **4e** (41.3 mg, 140 µmol, 70% yield) as pale yellow oil. **Rf** = 0.53 (SiO_2, 40:1 pentane:ethyl acetate). **^{1}H NMR :** ^{1}H NMR (400 MHz, $CDCl_3$) δ 7.34 – 7.27 (m, 2H, Ar*H*), 6.96 – 6.88 (m, 2H, Ar*H*), 6.58 (s, 2H, Ar*H*), 3.77 (s, 3H, OC*H*$_3$), 2.52 (s, 6H, ArC*H*$_3$), 1.53 – 1.48 (m, 2H, -CH^aH^{b}CH^aH^b-), 1.13 – 1.06 (m, 2H, -

CH^aH^bCH^aH^b-). **^{13}C NMR** : ^{13}C NMR (101 MHz, $CDCl_3$) δ 162.1 (d, *J* =249.5 Hz), 158.4, 140.1, 133.6, 133.6, 131.0, 120.22 (d, *J* =3.6 Hz), 115.5, 115.3, 113.6, 94.1, 74.5, 55.2, 20.9, 19.8, 13.0. **^{19}F NMR** (377 MHz, $CDCl_3$) δ -112.5. **IR** (ν_{max}, cm^{-1}) 3002 (w), 2955 (w), 2837 (w), 2228 (w), 1892 (w), 1603 (s), 1505 (s), 1484 (m), 1468 (m), 1329 (m), 1313 (s), 1223 (s), 1192 (m), 1158 (s), 1059 (m), 836 (s). **HRMS** (APPI/LTQ-Orbitrap) m/z: $[M]^+$ Calcd for $C_{20}H_{19}FO^+$ 294.1414; Found 294.1429.

2-(1-((4-Chlorophenyl)ethynyl)cyclopropyl)-5-methoxy-1,3-dimethylbenzene (4f)

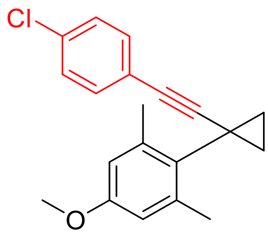

Following the general procedure C, starting from **1c** (35.3 mg, 200 µmol, 1.00 equiv) and 1-1-[4-chlorophenylethynyl]-1,2-benziodoxol-3(1H)-one (**2d**) (115 mg, 300 µmol, 1.50 equiv). The crude mixture was purified by column chromatography on Biotage (Büchi flashpure cartridge 25 g, gradient of pentane: ethyl acetate from 99:1 to 95:5) affording **4f** (49.2 mg, 158 µmol, 79% yield) as white solid. **MP** : 73 °C - 75 °C. **Rf** = 0.52 (SiO_2, 40:1 pentane:ethyl acetate). **^{1}H NMR** (400 MHz, $CDCl_3$) δ 7.26 – 7.16 (m, 4H, Ar*H*), 6.58 (s, 2H, Ar*H*), 3.77 (s, 3H, OC*H*$_3$), 2.52 (s, 6H, ArC*H*$_3$), 1.54 – 1.48 (m, 2H, -CH^aH^{b}CH^aH^b-), 1.14 – 1.07 (m, 2H, -CH^aH^bCH^aH^b-). **^{13}C NMR** (101 MHz, $CDCl_3$) δ 158.4, 140.1, 133.3, 133.1, 130.8, 128.5, 122.7, 113.6, 95.6, 74.5, 55.2, 20.9, 19.9, 13.0. **IR** (ν_{max}, cm^{-1}) 3089 (w), 3003 (m), 2954 (m), 2837 (w), 2226 (m), 1603 (s), 1488 (s), 1314 (s), 1158 (s), 828 (s). **HRMS** (APPI/LTQ-Orbitrap) m/z: $[M]^+$ Calcd for $C_{20}H_{19}ClO^+$ 310.1119; Found 310.1133.

2-(1-((4-Bromophenyl)ethynyl)cyclopropyl)-5-methoxy-1,3-dimethylbenzene (4g)

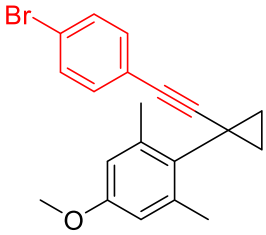

Following the general procedure C, starting from **1c** (35.3 mg, 200 µmol, 1.00 equiv) and 1-[4-bromophenylethynyl]-1,2-benziodoxol-3(1H)-one **2j** (128 mg, 300 µmol, 1.50 equiv). The crude mixture was purified by column chromatography on Biotage (Büchi flashpure cartridge 25 g, gradient of pentane: ethyl acetate from 99:1 to 95:5) affording **4g** (54.8 mg, 154 µmol, 77% yield) as white solid. **MP**: 74 °C – 76 °C. **Rf** = 0.57 (SiO_2, 20:1 pentane:ethyl acetate). **^{1}H NMR** (400 MHz, $CDCl_3$) δ 7.41 – 7.33 (m, 2H, Ar*H*), 7.22 – 7.15 (m, 2H, Ar*H*), 6.59 (s, 2H, Ar*H*), 3.77 (s, 3H, OC*H₃*), 2.52 (s, 6H, ArCH_3), 1.55 – 1.48 (m, 2H, -CH^aH^{b}CH^aH^b-), 1.16 – 1.08 (m, 2H, CH^aH^bCH^aH^b). **^{13}C NMR** (101 MHz, $CDCl_3$) δ 158.4, 140.1, 133.3, 131.4, 130.8, 123.2, 121.5, 113.6, 95.8, 74.6, 55.2, 20.9, 19.9, 13.0. **IR** (ν_{max}, cm^{-1}) 3001 (w), 2954 (m), 2837 (w), 2225 (w), 1602 (s), 1484 (s), 1326 (s), 1312 (s), 1157 (s), 1066 (s), 1058 (s), 823 (s). **HRMS** (APPI/LTQ-Orbitrap) m/z: $[M + H]^+$ Calcd for $C_{20}H_{20}BrO^+$ 355.0692; Found 355.0707.

2-(1-((3-Fluorophenyl)ethynyl)cyclopropyl)-5-methoxy-1,3-dimethylbenzene (4h)

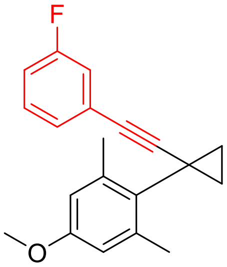

Following the general procedure C, starting from **1c** (35.3 mg, 200 µmol, 1.00 equiv) and 1-[3-fluorophenylethynyl]-1,2-benziodoxol-3(1H)-one **2f** (110 mg, 300 µmol, 1.50 equiv). The crude mixture was purified by column chromatography on Biotage (Büchi flashpure cartridge 25 g, gradient of pentane: ethyl acetate from 99:1 to 95:5) affording **4h** (36.2 mg, 123 µmol, 61% yield) as pale yellow oil. **Rf** = 0.3 (SiO_2, 40:1 pentane:ethyl acetate). **^{1}H NMR** (400 MHz, $CDCl_3$) δ 7.18 (td, *J* =8.0, 5.9 Hz, 1H, Ar*H*), 7.10 (dt, *J* =7.7, 1.3 Hz, 1H, Ar*H*), 7.02 (ddd, *J* =9.8, 2.7, 1.5 Hz, 1H, Ar*H*), 6.96 – 6.89 (m, 1H, Ar*H*), 6.59 (s, 2H, Ar*H*), 3.77 (s, 3H, OCH_3), 2.52 (s,

6H, ArC*H*$_3$), 1.55 – 1.49 (m, 2H, C$H^aH^bCH^aH^b$), 1.15 – 1.09 (m, 2H, $CH^aH^bCH^aH^b$). **^{13}C NMR** (101 MHz, $CDCl_3$) δ 162.4 (d, *J* =245.8 Hz), 158.4, 140.1, 130.8, 129.7 (d, *J* =8.7 Hz), 127.7 (d, *J* =2.9 Hz), 126.1 (d, *J* =9.6 Hz), 118.6 (d, *J* =22.5 Hz), 114.8 (d, *J* =21.1 Hz), 113.6, 95.7, 74.5 (d, *J* =3.4 Hz), 55.2, 20.9, 19.9, 13.0. **^{19}F NMR** (377 MHz, $CDCl_3$) δ -113.6. **IR** (ν_{max}, cm^{-1}) 2957 (m), 2837 (w), 2220 (m), 1606 (s), 1579 (s), 1487 (s), 1467 (m), 1315 (s), 1159 (s), 1059 (m), 920 (s), 783 (s).

HRMS (APPI/LTQ-Orbitrap) m/z: [M + H]$^+$ Calcd for $C_{20}H_{20}FO^+$ 295.1493; Found 295.1489.

2-(1-((2-Bromophenyl)ethynyl)cyclopropyl)-5-methoxy-1,3-dimethylbenzene (4i)

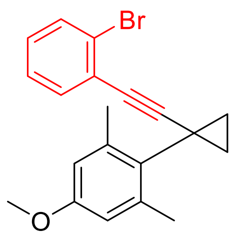

Following the general procedure C, starting from **1c** (35.3 mg, 200 µmol, 1.00 equiv) and 1-[2-bromophenylethynyl]-1,2-benziodoxol-3(1H)-one **2k** (128 mg, 300 µmol, 1.50 equiv). The crude mixture was purified by column chromatography on Biotage (Büchi flashpure cartridge 25 g, gradient of pentane: ethyl acetate from 99:1 to 95:5) affording **4i** (38.5 mg, 108 µmol, 54% yield) as pale yellow oil. **Rf** = 0.5 (SiO_2, 40:1 pentane:ethyl acetate). **^{1}H NMR** (400 MHz, $CDCl_3$) δ 7.51 (dd, *J* =8.0, 1.3 Hz, 1H, Ar*H*), 7.35 (dd, *J* =7.7, 1.7 Hz, 1H, Ar*H*), 7.17 (td, *J* =7.6, 1.3 Hz, 1H, Ar*H*), 7.07 (ddd, *J* =8.1, 7.4, 1.7 Hz, 1H, Ar*H*), 6.62 – 6.56 (m, 2H, Ar*H*), 3.77 (s, 3H, OCH_3), 2.54 (s, 6H, ArC*H*$_3$), 1.66 – 1.56 (m, 2H, C$H^aH^bCH^aH^b$), 1.20 – 1.09 (m, 2H, $CH^aH^bCH^aH^b$). **^{13}C NMR** (101 MHz, $CDCl_3$) δ 158.4, 140.2, 133.3, 132.3, 130.7, 128.6, 126.9, 126.1, 126.1, 113.6, 99.7, 74.4, 55.2, 20.9, 20.0, 13.2. **IR** (ν_{max}, cm^{-1}) 3001 (w), 2955 (w), 2836 (w), 2229 (w), 1750 (w), 1603 (s), 1485 (m), 1467 (s), 1330 (m), 1314 (s), 1158 (s), 1058 (m), 1028 (m), 754 (s).**HRMS** (APPI/LTQ-Orbitrap) m/z: [M]$^+$ Calcd for $C_{20}H_{19}BrO^+$ 354.0614; Found 354.0630.

1,3-Diethyl-5-methoxy-2-(1-(phenylethynyl)cyclopropyl)benzene (4j)

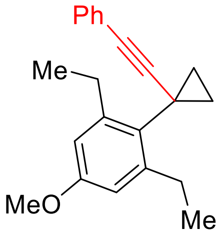

Following the general procedure C, starting from **1l** (40.8 mg, 200 µmol, 1.00 equiv) and **PhEBX** (139.2 mg, 400 µmol, 2.0 equiv). After reaction, **1l** (22.3 mg, 109 µmol, 55%) was recovered and 1,3-diethyl-5-methoxy-2-(1-(phenylethynyl)cyclopropyl)benzene **4j** (25.5 mg, 0.840 mmol, 42% yield) was obtained as off-white amorphos solid. **Rf** = 0.38 (SiO_2, 50:1 pentane:ethyl acetate). **^{1}H NMR (**400 MHz, $CDCl_3$) δ 7.32 – 7.28 (m, 2H, Ar*H*), 7.24 – 7.19 (m, 3H, Ar*H*), 6.64 (s, 2H, Ar*H*), 3.80 (s, 3H, ArOCH_3), 3.09 (dq, *J* =15.1, 7.6 Hz, 2H, ArCH_2CH$_3$), 2.89 (dq, *J* =14.9, 7.5 Hz, 2H, ArCH_2CH$_3$), 1.55 – 1.52 (m, 2H, ArCCH_2), 1.33 (t, *J* =7.5 Hz, 6H, ArCH$_2$CH_3), 1.16 – 1.08 (m, 2H, ArCCH_2). **^{13}C NMR** (101 MHz, $CDCl_3$) δ 158.9, 146.2, 131.7, 130.4, 128.2, 127.4, 124.2, 111.6, 95.7, 75.8, 55.2, 26.2, 20.2, 15.3, 12.4. **IR** (ν_{max}, cm^{-1}) 2964 (m), 2933 (m), 2226 (w), 1751 (w), 1601 (s), 1467 (s), 1331 (m), 1155 (s), 1102 (m), 1035 (m). **HRMS** (APCI/QTOF) m/z: [M + H]$^+$ Calcd for $C_{22}H_{25}O^+$ 305.1900; Found 305.1903.

5-(Cyclopropylmethoxy)-1,3-dimethyl-2-(1-(phenylethynyl)cyclopropyl)benzene (4k)

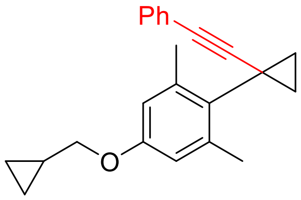

Following the general procedure C, starting from **1m** (43.2 mg, 200 µmol, 1.00 equiv) and **PhEBX** (139.2 mg, 400.0 µmol, 2.00 equiv). 5-(cyclopropylmethoxy)-1,3-dimethyl-2-(1-(phenylethynyl)cyclopropyl)benzene **4k** (46.8 mg, 0.148 mmol, 74% yield) was obtained as sticky solid. **Rf** = 0.40 (SiO_2, 40:1 pentane:ethyl acetate). **^{1}H NMR** (400 MHz, $CDCl_3$) δ 7.36 – 7.29 (m, 2H, Ar*H*), 7.22 (dd, *J* =5.1, 2.0 Hz, 3H, Ar*H*), 6.59 (s, 2H, Ar*H*), 3.76 (d, *J* =6.9 Hz, 2H, ArOCH_2), 2.51 (s, 6H, ArCH_3), 1.51 (q, *J* =4.2 Hz, 2H, CH_2), 1.31 – 1.22 (m, 1H, CHCH_2), 1.09 (q, *J* =4.2 Hz, 2H, CH_2), 0.69 – 0.58 (m, 2H, CH_2), 0.38 – 0.28 (m, 2H, CH_2). **^{13}C NMR** (101 MHz, $CDCl_3$) δ 157.8, 140.1, 131.8, 131.0, 128.2, 127.4, 124.2, 114.2, 94.6, 75.5, 72.7, 20.9, 19.8, 13.0, 10.5, 3.3. **IR** (ν_{max}, cm^{-1}) 3081 (w), 3007 (m), 2917 (m), 2226 (w), 1603 (s), 1486 (s), 1312

(s), 1157 (s), 1051 (s). **HRMS** (ESI/QTOF) m/z: $[M + H]^+$ Calcd for $C_{23}H_{25}O^+$ 317.1900; Found 317.1906.

2-Benzyl-1-(phenylethynyl)cyclopropyl)-5-methoxy-1,3-dimethylbenzene (4l)

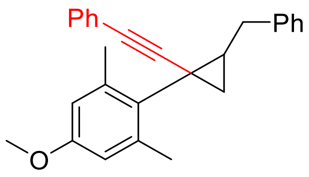

Following the general procedure C, starting from **1f** (73.0 mg, 200.0 µmol, 1.00 equiv) and **PhEBX** (139 mg, 400 µmol, 2.00 equiv). 2-Benzyl-1-(phenylethynyl)cyclopropyl)-5-methoxy-1,3-dimethylbenzene **4l** (43.6 mg, 119 µmol, 60% yield for two diastereomers combined) was obtained as a mixture of two diastereomers, pale yellow oil. dr. 70:30. NMR signal at Ar*CH₂* was used to determine dr. **Rf** = 0.47 (SiO_2, 40:1 pentane:ethyl acetate. **^{1}H NMR** ArCH_2 of minor diastereomers: 3.43 (dd, *J* =14.8, 4.8 Hz, 1H). Major diastereomers: **^{1}H NMR** (400 MHz, $CDCl_3$) δ 7.36 – 7.31 (m, 3H, Ar*H*), 7.29 (dd, *J* =7.0, 1.6 Hz, 2H, Ar*H*), 7.24 – 7.21 (m, 3H, Ar*H*), 7.18 – 7.13 (m, 2H, Ar*H*), 6.70 – 6.60 (m, 2H, Ar*H*), 3.81 (s, 3H, ArOCH_3), 3.19 (dd, *J* =14.7, 4.0 Hz, 1H, ArCH_2), 2.65 (s, 3H, ArCH_3), 2.49 (s, 3H, ArCH_3), 2.08 – 1.97 (m, 1H, ArCH_2C*H*), 1.80 – 1.72 (m, 1H, ArCCH_2), 1.65 (dd, *J* =14.7, 10.7 Hz, 1H, ArCH_2), 1.09 – 1.02 (m, 1H, ArCCH_2). **^{13}C NMR** (101 MHz, $CDCl_3$) δ 158.2, 141.1, 140.0, 131.8, 128.8, 128.5, 128.2, 127.4, 126.1, 124.2 114.0, 113.8, 95.2, 75.8, 55.2, 35.7, 30.7, 25.4, 21.5, 20.9, 19.1. **IR** (ν_{max}, cm^{-1}) 2947 (m), 2938 (m), 2837 (m), 2218 (w), 1601 (s), 1487 (s), 1447 (s), 1314 (s), 1308 (s), 1194 (m), 1153 (s), 1075 (m). **HRMS** (APPI/LTQ-Orbitrap) m/z: $[M + H]^+$ Calcd for $C_{27}H_{27}O^+$ 367.2056; Found 367.2060.

((1R,2S)-2-(4-Methoxy-2,6-dimethylphenyl)-2-(phenylethynyl)cyclopropyl)methyl 2-(11-oxo-6,11-dihydrodibenzo[b,e]oxepin-2-yl)acetate (4m)

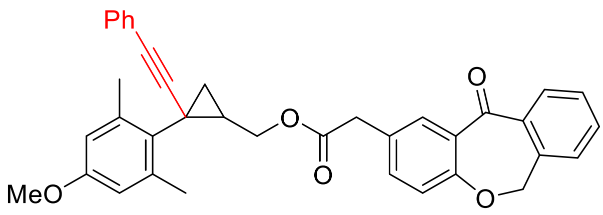

Following the general procedure C, starting from **1n** (91.3 mg, 200 µmol, 1.00 equiv) and **PhEBX** (139.2 mg, 400.0 µmol, 2.00 equiv). ((1R,2S)-2-(4-Methoxy-2,6-dimethylphenyl)-2-(phenylethynyl)cyclopropyl)methyl 2-(11-oxo-6,11-dihydrodibenzo[b,e]oxepin-2-yl)acetate

4m (79.0 mg, 0.142 mmol, 71% yield for two diastereomers combined) as sticky solid. **Rf** = 0.16 (SiO_2, 10:1 pentane:ethyl acetate). dr. 55:45. NMR signal at COOC*H₂*CH was used to determine dr. **^{1}H NMR** COOCH_2CH of minor diastereomer 4.53 (dd, *J* =7.3, 3.1 Hz, 2H). Major diastereomer: **^{1}H NMR** (400 MHz, $CDCl_3$) δ 8.11 (d, *J* =2.4 Hz, 1H, Ar*H*), 7.89 – 7.86 (m, 1H, Ar*H*), 7.50 – 7.45 (m, 2H, Ar*H*), 7.43 – 7.41 (m, 1H, Ar*H*), 7.38 – 7.36 (m, 1H, Ar*H*), 7.25 – 7.18 (m, 5H, Ar*H*), 7.02 (d, *J* =8.4 Hz, 1H, Ar*H*), 6.57 – 6.55 (m, 2H, Ar*H*), 5.19 (s, 2H, ArCH_2), 4.39 – 4.28 (m, 1H, COOCH_2CH), 3.75 (s, 3H, ArOCH_3), 3.60 (s, 2H, ArCH_2), 3.37 (dd, *J* =11.7, 8.7 Hz, 1H, COOCH_2CH), 2.50 (s, 3H, ArCH_3), 2.45 (s, 3H, ArCH_3), 2.16 – 2.03 (m, 1H, C*H*CH_2), 1.82 – 1.75 (m, 1H, CH_2CHAr), 1.16 (dd, *J* =6.5, 5.0 Hz, 1H, CH_2CHAr). **^{13}C NMR** (101 MHz, $CDCl_3$) δ 191.0, 171.5, 160.6, 158.4, 140.6, 140.0, 136.4, 135.7, 132.9, 132.6, 131.8, 129.6, 128.2, 127.9, 127.7, 125.3, 123.8, 121.2, 114.2, 113.8, 93.8, 76.4, 73.8, 65.3, 55.2, 40.3, 27.5, 23.2, 21.4, 20.5, 19.2. **IR** (ν_{max}, cm^{-1}) 2969 (m), 2251 (w), 1734 (s), 1647 (m), 1601 (s), 1490 (s), 1413 (m), 1300 (s), 1282 (s), 1221 (s), 1156 (s), 1139 (s), 1016 (s), 910 (m). **HRMS** (ESI/QTOF) m/z: $[M + H]^+$ Calcd for $C_{37}H_{33}O_5^+$ 557.2323; Found 557.2318.

2-(4-Methoxy-2,6-dimethylphenyl)-1-(phenylethynyl)cyclopropane-1-carboxylate (4n)

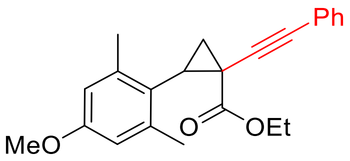

Following the general procedure C, starting from **1k** (25.0 mg, 100 µmol, 1.00 equiv) and **PhEBX** (69.9 mg, 200 µmol, 2.0 equiv). After reaction, **1k** (14.5 mg, 58 µmol, 58%) was recovered and Ethyl 2-(4-methoxy-2,6-dimethylphenyl)-1-(phenylethynyl)cyclopropane-1-carboxylate **4n** (12.2 mg, 35.0 µmol, 35% yield) was obtained as single diastereomer, sticky solid. **Rf** = 0.28 (SiO_2, 20:1 pentane:ethyl acetate). **^{1}H NMR** (400 MHz, $CDCl_3$) δ 7.37 – 7.30 (m, 2H, Ar*H*), 7.26 – 7.21 (m, 3H, Ar*H*), 6.60 – 6.52 (m, 2H, Ar*H*), 3.98 (q, *J* =7.1 Hz, 2H, CH_3CH_2CO), 3.75 (s, 3H, ArOCH_3), 2.58 (dd, *J* =8.4, 6.5 Hz, 1H, C*H*CH_2), 2.49 (s, 3H, ArCH_3), 2.44 (s, 3H, ArCH_3), 1.98 (dd, *J* =8.5, 4.6 Hz, 1H, CHCH_2), 1.92 (dd, *J* =6.5, 4.7 Hz, 1H, CHCH_2), 1.09 (t, *J* =7.1 Hz, 3H, C$H_3$$CH_2$CO). **^{13}C NMR** (101 MHz, $CDCl_3$) δ 170.4, 158.3, 140.2, 139.3, 131.9, 128.3, 128.0, 126.4, 123.4, 113.8, 113.7, 92.3, 60.8, 55.1, 31.8, 25.4, 23.5, 21.4, 20.2, 14.1. **IR** (ν_{max}, cm^{-1}) 2981 (w), 2359 (w), 2229 (w), 1728 (s), 1602 (m), 1487 (m), 1379 (m), 1322 (m), 1191 (s), 1159 (s). **HRMS** (ESI/QTOF) m/z: $[M + H]^+$ Calcd for $C_{23}H_{25}O_3^+$ 349.1798; Found 349.1803.

1-(4-Methoxy-2,6-dimethylphenyl)-1-(phenylethynyl)spiro[2.3]hexane-5-carbonitrile (4o)

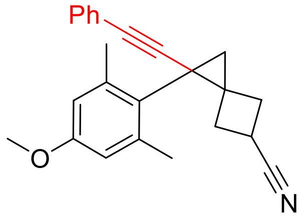

Following the general procedure C, starting from **1h** (48.2 mg, 200 µmol, 1.00 equiv) and **PhEBX** (139.2 mg, 400.0 µmol, 2.00 equiv). 1-(4-methoxy-2,6-dimethylphenyl)-1-(phenylethynyl)spiro[2.3]hexane-5-carbonitrile **4o** (43.1 mg, 0.127 mmol, 63% yield for two diastereomer combined) as colorless oil. dr. : 57:43. NMR signal was used to determine dr at -C*H₂* : 2.17 – 2.09 (m, 1st diastereomer) and 2.33 – 2.24 (m, 2nd diastereomer). **Rf** = 0.42 (SiO_2, 10:1 pentane:ethyl acetate). **HRMS** (Sicrit plasma/LTQ-Orbitrap) m/z: $[M + H]^+$ Calcd for $C_{24}H_{24}NO^+$ 342.1852; Found 342.1840. **IR** (ν_{max}, cm^{-1}) 2997 (w), 2936 (m), 2849 (w), 2237 (w), 1736 (m), 1601 (m), 1487 (m), 1303 (m), 1153 (s), 910 (s).

1st diastereomer (**4o1**): **^{1}H NMR** (400 MHz, $CDCl_3$) δ 7.35 – 7.29 (m, 2H, Ar*H*), 7.26 – 7.22 (m, 3H, Ar*H*), 6.65 – 6.56 (m, 2H, Ar*H*), 3.77 (s, 3H, ArOCH_3), 3.35 – 3.24 (m, 1H, C*H*CH_2), 3.12 (dd, *J* =12.8, 10.0 Hz, 1H, CH_2), 2.70 (dd, *J* =12.9, 6.5 Hz, 1H, CH_2), 2.48 (s, 3H, ArCH_3), 2.42 (s, 3H, ArCH_3), 2.33 – 2.24 (m, 2H, CH_2), 1.64 (d, *J* =5.6 Hz, 1H, CH_2), 1.28 (d, *J* =5.5 Hz, 1H, CH_2). **^{13}C NMR** (101 MHz, $CDCl_3$) δ 158.4, 131.8, 128.3, 127.8, 127.3, 123.6, 122.9, 114.4, 113.8, 90.9, 79.3, 55.2, 32.6, 32.4, 30.9, 30.8, 23.0, 21.3, 20.4, 17.3.

2nd diastereomer (**4o2**): **^{1}H NMR** (400 MHz, $CDCl_3$) δ 7.37 – 7.31 (m, 2H, Ar*H*), 7.26 – 7.22 (m, 3H, Ar*H*), 6.67 – 6.54 (m, 2H, Ar*H*), 3.77 (s, 3H, ArOCH_3), 3.39 – 3.27 (m, 1H, C*H*CH_2), 3.19 – 3.10 (m, 1H, CH_2), 2.64 (s, 3H, ArCH_3), 2.64 – 2.55 (m, 1H, CH_2), 2.50 – 2.42 (m, 1H, CH_2), 2.41 (s, 3H, ArCH_3) 2.17 – 2.09 (m, 1H, CH_2), 1.63 (d, *J* =5.3 Hz, 1H, CH_2), 1.28 (d, *J* =5.3 Hz, 1H, CH_2).**^{13}C NMR** (101 MHz, $CDCl_3$) δ 158.4, 140.8, 139.4, 131.9, 128.3, 127.8, 127.2, 123.6, 122.4, 114.4, 113.8, 90.8, 79.0, 55.2, 33.4, 33.1, 31.2, 30.9, 22.6, 21.3, 21.1, 16.1.

7-(4-Methoxy-2,6-dimethylphenyl)-7-(phenylethynyl)bicyclo[4.1.0]heptane (4p)

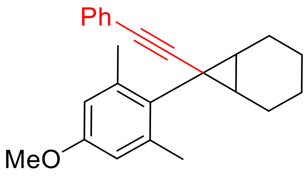

Following the general procedure C, starting from **1g** (45.9 mg, 200 µmol, 1.00 equiv) and **PhEBX** (139.2 mg, 400.0 µmol, 2.00 equiv). 7-(4-Methoxy-2,6-dimethylphenyl)-7-(phenylethynyl)bicyclo[4.1.0]heptane **4p** (33.7 mg, 102 µmol, 51% yield) was obtained as a single diastereomer, white solid. **Mp** : 77 – 79°C. **Rf** = 0.37 (SiO_2, 40:1 pentane:ethyl acetate). **^{1}H NMR** (400 MHz, $CDCl_3$) 7.33 – 7.27 (m, 2H, Ar*H*), 7.24 – 7.17 (m, 3H, Ar*H*), 6.61 (s, 2H, Ar*H*), 3.78 (s, 3H, ArOC*H₃*), 2.48 (s, 6H, ArC*H₃*), 2.00 – 1.89 (m, 2H, C*H₂*), 1.78 (m, 2H, ArCC*H*CH₂), 1.27 (m, 2H, C*H₂*), 1.17 – 1.07 (m, 2H, C*H₂*), 1.05 – 0.94 (m, 2H, C*H₂*). **^{13}C NMR** (101 MHz, $CDCl_3$) δ 158.1, 141.3, 131.8, 131.8, 128.2, 128.1, 127.2, 126.3, 124.6, 113.5, 96.8, 74.2, 55.1, 27.1, 20.5, 20.3, 20.2, 20.0. **IR** (ν_{max}, cm^{-1}) 2942 (s), 2936 (s), 2932 (s), 2863 (m), 2856 (m), 2213 (w), 1601 (s), 1488 (s), 1321 (s), 1193 (m), 1159 (s), 856 (m). **HRMS** (Sicrit plasma/LTQ-Orbitrap) m/z: $[M + H]^+$ Calcd for $C_{24}H_{27}O^+$ 331.2056; Found 331.2052.

1-(4-Methoxy-2,6-dimethylphenyl)-1-(phenylethynyl)spiro[2.5]octane (4q1)

Following the general procedure C, starting from **1i** (48.8 mg, 200 µmol, 1.00 equiv) and **PhEBX** (139.2 mg, 400.0 µmol, 2.00 equiv). 1-(4-methoxy-2,6-dimethylphenyl)-1-(phenylethynyl)spiro[2.5]octane **4q1** (30 mg, 0.088 mmol, 44% yield) as colorless oil and 1-(2-(4-methoxy-2,6-dimethylphenyl)-4-phenylbut-3-yn-1-yl)cyclohexyl 2-iodobenzoate **4q2** (20 mg, 0.034 mmol, 17% yield) as pale yellow oil.

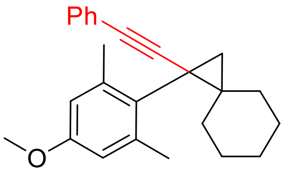

Rf = 0.28 (SiO_2, 40:1 pentane:ethyl acetate). **^{1}H NMR** (400 MHz, $CDCl_3$) δ 7.35 – 7.29 (m, 2H, Ar*H*), 7.24 – 7.16 (m, 3H, Ar*H*), 6.59 (d, *J* =2.9 Hz, 1H, Ar*H*), 6.54 (d, *J* =2.8 Hz, 1H, Ar*H*), 3.76 (s, 3H, ArOC*H₃*), 2.46 (s, 3H, ArC*H₃*), 2.44 (s, 3H, ArC*H₃*), 1.98 – 1.70 (m, 4H, C*H₂*), 1.65 –1.40 (m, 3H, C*H₂*), 1.32 – 1.19 (m, 3H, C*H₂*), 1.03 (dd, *J* =4.5, 1.4 Hz, 1H, C*H₂*), 0.57 – 0.48 (m, 1H,

CH_2). **^{13}C NMR** (101 MHz, $CDCl_3$) δ 157.8, 141.0, 139.6, 131.8, 129.4, 128.2, 127.2, 124.6, 113.6, 113.5, 93.3, 78.4, 55.1, 34.0, 33.1, 32.7, 30.1, 26.6, 26.1, 25.9, 24.9, 21.4, 20.7. **IR** (ν_{max}, cm^{-1}) 2939 (s), 2920 (s), 2851 (s), 2220 (w), 1727 (m), 1602 (s), 1486 (s), 1445 (s), 1309 (s), 1152 (s), 1069 (s), 855 (m). **HRMS** (APPI/LTQ-Orbitrap) m/z: $[M]^+$ Calcd for $C_{25}H_{28}O^+$ 344.2135; Found 344.2140.

1-(2-(4-Methoxy-2,6-dimethylphenyl)-4-phenylbut-3-yn-1-yl)cyclohexyl 2-iodobenzoate (4q2)

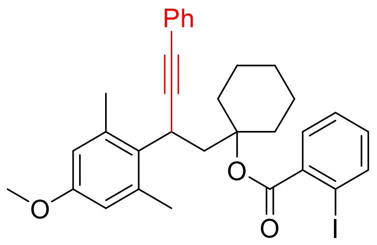

Rf = 0.28 (SiO_2, 40:1 pentane:ethyl acetate). **^{1}H NMR** (400 MHz, $CDCl_3$) δ 7.90 (ddd, *J* =8.0, 6.6, 1.4 Hz, 2H, Ar*H*), 7.30 - 7.23 (m, 1H, Ar*H*), 7.21 – 7.12 (m, 5H, Ar*H*), 7.03 (td, *J* =7.6, 1.8 Hz, 1H, Ar*H*), 6.62 – 6.46 (m, 2H, Ar*H*), 4.48 (dd, *J* =10.3, 3.8 Hz, 1H, ArC*H*), 3.74 (s, 3H, ArOCH_3), 3.03 (dd, *J* =14.7, 10.3 Hz, 1H, ArCHCH_2), 2.78 – 2.70 (m, 1H, CH_2), 2.65 (s, 3H, ArCH_3), 2.56 – 2.51 (m, 1H, CH_2), 2.32 (s, 3H, ArCH_3), 2.23 (dd, *J* =14.7, 3.8 Hz, 1H, ArCHCH_2), 1.81 – 1.70 (m, 2H, CH_2), 1.68 – 1.61 (m, 5H, CH_2), 1.42 – 1.33 (m, 1H, CH_2). **^{13}C NMR** (101 MHz, $CDCl_3$) δ 165.3, 157.7, 141.6, 139.0, 136.2, 135.7, 132.3, 131.4, 131.2, 128.1, 127.8, 127.4, 124.1, 115.4, 113.9, 94.5, 92.3, 85.9, 82.2, 55.2, 41.4, 35.1, 34.9, 27.2, 25.7, 22.3, 22.2, 21.6. **IR** (ν_{max}, cm^{-1}) 2934 (m), 2860 (m), 2251 (w), 1724 (s), 1602 (m), 1303 (s), 1289 (s), 1245 (s), 1129 (s). **HRMS** (ESI/QTOF) m/z: $[M + Na]^+$ Calcd for $C_{32}H_{33}INaO_3^+$ 615.1367; Found 615.1367

2-(1-chlorocyclopropyl)-5-methoxy-1,3-dimethylbenzene (4r)

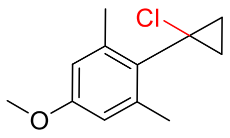

Following the general procedure C, starting from **1c** (35.3 mg, 200 µmol, 1.00 equiv) and Cl-HIR **2l** reagent (84.7 mg, 350 µmol, 1.5 equiv) under 427 nm kessil lamp. Product 2-(1-chlorocyclopropyl)-5-methoxy-1,3-dimethylbenzene **4r** was obatained as colorless oil (36.5 mg, 0.174 mmol, 87%). Note that the product is not very stable in silicagel, therefore, a short

pad of silicagel with 10% of ethylacetate in pentane were used for column chromatography to make sure the product was not staying too long in silicagel.

Rf = 0.35 (SiO_2, 40:1 pentane:ethyl acetate). **^{1}H NMR** (400 MHz, $CDCl_3$) δ 6.58 (s, 2H, Ar*H*), 3.77 (s, 3H, ArOC*H3*), 2.51 (s, 6H, ArC*H3*), 1.23 – 1.19 (m, 2H, C*H2*), 0.88 – 0.83 (m, 2H, C*H2*). **^{13}C NMR** (101 MHz, $CDCl_3$) δ 159.0, 140.3, 130.6, 113.8, 55.2, 54.0, 20.6, 16.1. **IR** (ν_{max}, cm^{-1}) 3004 (w), 2957 (m), 2922 (m), 2839 (w), 1731 (w), 1604 (s), 1486 (m), 1455 (m), 1377 (m), 1315 (s), 1216 (s), 1158 (s), 1099 (m), 1058 (m), 1025 (m), 939 (w), 861 (m), 741 (m). **HRMS** (APPI/LTQ-Orbitrap) m/z: $[M]^+$ Calcd for $C_{12}H_{15}ClO^+$ 210.0806; Found 210.0804.

2-(1-(cyclopropylethynyl)cyclopropyl)-5-methoxy-1,3-dimethylbenzene (4s1)

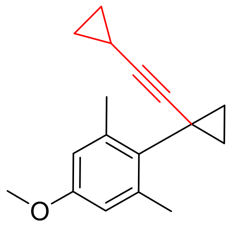

Following the general procedure C, starting from **1c** (17.8 mg, 100 µmol, 1.00 equiv). Product 2-(1-(cyclopropylethynyl)cyclopropyl)-5-methoxy-1,3-dimethylbenzene **4r** was obtained as off white amorphous solid (4.56 mg, 0.0189 mmol, 19%). **Rf** = 0.27 (SiO_2, pentane). **^{1}H NMR** (400 MHz, $CDCl_3$) δ 6.54 (s, 2H, Ar*H*), 3.75 (s, 3H, ArOC*H3*), 2.45 (s, 6H, ArC*H3*), 1.32 – 1.27 (m, 2H, CC*H2*), 1.13 (tt, *J* = 8.2, 5.0 Hz, 1H), 0.94 – 0.89 (m, 2H, CC*H2*), 0.69 – 0.61 (m, 2H, CHC*H2*), 0.57 – 0.48 (m, 2H, CHC*H2*). **^{13}C NMR** (101 MHz, $CDCl_3$) δ 158.4, 140.1, 132.1, 113.8, 80.1, 78.7, 55.5, 21.1, 19.6, 12.8, 8.7, 0.1. **IR** (ν_{max}, cm^{-1}) 3006 (m), 2959 (m), 2924 (m), 2837 (m), 2362 (m), 1747 (m), 1603 (s), 1485 (s), 1319 (s), 1160 (s). **HRMS** (Sicrit plasma/LTQ-Orbitrap) m/z: $[M + H]^+$ Calcd for $C_{17}H_{21}O^+$ 241.1587; Found 241.1578.

N-(5-(4-chlorophenyl)-1-methoxypent-4-yn-1-yl)-4-methylbenzamide (6a)

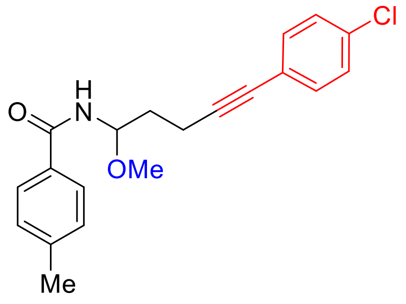

In a 12*75 mm Borosilicate glass tube, **2a** (95.6 mg, 250 µmol, 2.50 equiv) and N-cyclopropyl-4-methylbenzamide (**5a**) (17.5 mg, 100 µmol, 1.00 equiv) were added. The tube was then closed with a rubber septum and sealed off with parafilm. Three cycles of evacuate-refill with nitrogen were performed to remove O_2 and DCM (1.0 mL, 0.1M) was added, followed by the addition of methanol (6.41 mg, 200 µmol, 2.00 equiv). The reaction mixture was stirred at room temperature under irradiation of Kessil lamps (440 nm). The reaction was monitored by NMR with CH_2Br_2 as an internal standard. Upon completion by either full conversion of starting material or hypervalent iodine reagents, the mixture was concentrated in vacuo and purified by column chromatography to give product **6a** (18.1 mg, 52.7 µmol, 53% yield) as a white solid. **^{1}H NMR** (400 MHz, $CDCl_3$) δ 7.67 (d, *J* =8.2 Hz, 2H, Ar*H*), 7.25 (d, *J* =8.5 Hz, 2H, Ar*H*), 7.23 (d, *J* =8.7 Hz, 2H, Ar*H*), 7.15 (d, *J* =8.0 Hz, 2H, Ar*H*) 6.63 (d, *J* =9.6 Hz, 1H, N*H*), 5.53 (dt, *J* =9.6, 5.7 Hz, 1H, NHC*H*), 3.43 (s, 3H, OCH_3), 2.65 (dt, *J* =17.1, 7.0 Hz, 1H, CHCH$_2$CH_2), 2.54 (dt, *J* =17.1, 6.9 Hz, 1H, CHCH$_2$CH_2), 2.36 (s, 3H, ArCH_3), 2.09 – 1.94 (m, 2H, CHCH_2CH$_2$). **^{13}C NMR** (101 MHz, $CDCl_3$) δ 167.5, 142.6, 133.9, 133.0, 131.0, 129.4, 128.6, 127.2, 127.1, 122.1, 90.4, 80.7, 80.6, 56.3, 34.2, 21.6, 15.0. **HRMS** (ESI/QTOF) m/z: $[M + Na]^+$ Calcd for $C_{20}H_{20}ClNNaO_2^+$ 364.1075; Found 364.1075 Consistent with reported value.[19]

N-(1-butoxy-5-(4-fluorophenyl)pent-4-yn-1-yl)-4-methylbenzamide (6b)

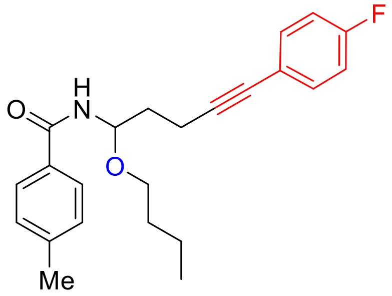

In a 12*75 mm Borosilicate glass tube, **2a** (91.5 mg, 250 µmol, 2.50 equiv) and N-cyclopropyl-4-methylbenzamide (**5a**) (17.5 mg, 100 µmol, 1.00 equiv) were added. The tube was then closed with a rubber septum and sealed off with parafilm. Three cycles of evacuate-refill with nitrogen were performed to remove O_2 and DCM (1.0 mL, 0.1 M) was added, followed by the addition of methanol (6.41 mg, 200 µmol, 2.00 equiv). The reaction mixture was stirred at room temperature irradiating with Kessil lamps (440 nm). The reaction was monitored by NMR with CH_2Br_2 as an internal standard. Upon completion by either full conversion of starting material or hypervalent iodine reagents, the mixture was concentrated in vacuo and

purified by column chromatography to give product **6b** as a white solid (18.7 mg, 50.9 µmol, 51% yield) **^{1}H NMR** (400 MHz, $CDCl_3$) δ 7.67 (d, *J* =8.1 Hz, 2H, Ar*H*), 7.35 – 7.29 (m, 2H, Ar*H*), 7.16 (d, *J* =8.0 Hz, 2H, Ar*H*), 6.99 – 6.91 (m, 2H, Ar*H*), 6.60 (d, *J* =9.5 Hz, 1H, N*H*), 5.61 (ddd, *J* =9.5, 6.3, 5.5 Hz, 1H, NHC*H*), 3.67 (dt, *J* =9.5, 6.6 Hz, 1H, OC*H₂*CH₂), 3.55 (dt, *J* =9.6, 6.6 Hz, 1H, OC*H₂*CH₂), 2.65 (dt, *J* =17.0, 6.9 Hz, 1H, CHCH₂C*H₂*), 2.54 (dt, *J* =17.1, 7.0 Hz, 1H, CHCH₂C*H₂*), 2.39 (s, 3H, ArC*H₃*), 2.12 – 1.93 (m, 2H, CHC*H₂*CH₂), 1.59 – 1.48 (m, 2H, OCH₂C*H₂*), 1.46 – 1.32 (m, 2H, OCH₂CH₂C*H₂*), 0.90 (t, *J* =7.4 Hz, 3H, CH₂C*H₃*). **^{13}C NMR** (101 MHz, $CDCl_3$) δ 167.1, 162.2 (d, *J* =248.5 Hz), 142.3, 133.4 (d, *J* =8.3 Hz), 131.1, 129.3, 127.0, 119.6 (d, *J* =3.4 Hz), 115.4 (d, *J* =21.9 Hz), 88.9, 80.3, 79.2, 68.4, 34.4, 31.8, 21.5, 19.4, 15.0, 13.9. **^{19}F NMR** (376 MHz, $CDCl_3$) δ -112.0. **HRMS** (ESI/QTOF) m/z: $[M + Na]^+$ Calcd for $C_{23}H_{26}FNNaO_2^+$ 390.1840; Found 390.1846. Consistent with reported value.[19]

3-(4-Methoxyphenyl)-5-phenylpent-4-yn-2-yl 2-iodobenzoate (8a)

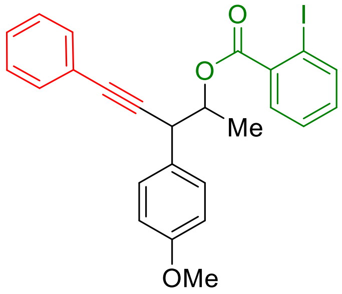

Following the general procedure C, starting from 1-methoxy-4-[(E)-prop-1-enyl]benzene (**7a**) (29.6 mg, 200 µmol, 1.00 equiv) and phenyl ethynyl benziodoxolone (**2a**) (174 mg, 500 µmol, 2.50 equiv). The crude mixture was purified by column chromatography on Biotage (Büchi flashpure cartridge 25 g, gradient of pentane: ethyl acetate from 95:5 to 88:12) affording 3-(4-methoxyphenyl)-5-phenylpent-4-yn-2-yl 2-iodobenzoate (**8a**) as colorless oil (55.8 mg, 112 µmol, 56% yield for two diastereomers combined, dr 2:1, the ratio was determined by integration nof the ^{1}H NMR peaks of the benzylic protons ArC*H*). **Rf** = 0.35 (SiO_2, 20:1 pentane:ethyl acetate). **^{1}H NMR** (400 MHz, $CDCl_3$, signals for major diastereomer) δ 7.98 (dt, *J* =7.9, 1.4 Hz, 1H, Ar*H*), 7.78 (dd, *J* =7.8, 1.7 Hz, 1H, Ar*H*), 7.44 – 7.41 (m, 2H, Ar*H*), 7.41 – 7.38 (m, 2H, Ar*H*), 7.36-7.37 (m, 1H, Ar*H*), 7.31 – 7.27 (m, 3H, Ar*H*), 7.13 (td, *J* =7.7, 1.7 Hz, 1H, Ar*H*), 6.93 – 6.86 (m, 2H, Ar*H*), 5.49 (p, *J* =6.3 Hz, 1H, OC*H(*CH₃)CH₂), 4.18 (d, *J* =5.9 Hz, 1H, ArC*H*CH(CH₃)), 3.81 (s, 3H, OC*H₃*), 1.42 (d, *J* =6.3 Hz, 3H, CH(CH₃)). **^{13}C NMR** (101 MHz, $CDCl_3$,

signals for major diastereomer) δ 165.8, 159.2, 141.4, 135.3, 132.7, 131.8, 131.1, 129.9, 129.7, 128.4, 128.2, 128.0, 123.4, 114.0, 94.3, 88.3, 84.6, 74.7, 55.4, 43.4, 17.7. **IR** (ν_{max}, cm^{-1}) 2987 (w), 2933 (w), 2836 (w), 1738 (m), 1721 (s), 1512 (m), 1289 (s), 1250 (s), 1179 (m), 1129 (m), 1101 (m), 1065 (m), 1057 (m), 1032 (m), 1012 (m), 833 (m), 758 (m), 741 (s). **HRMS** (ESI/QTOF) m/z: $[M + Na]^+$ Calcd for $C_{25}H_{21}INaO_3^+$ 519.0428; Found 519.0436.

2-(4-Methoxyphenyl)-4-phenylbut-3-yn-1-yl 2-iodobenzoate (8b)

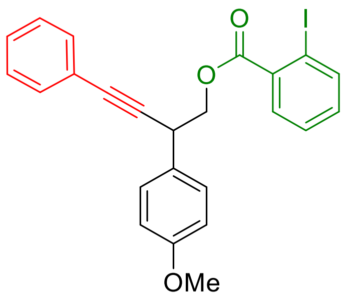

Following the general procedure C, starting from 1-ethenyl-4-methoxybenzene (**7b**) (26.8 mg, 200 µmol, 1.00 equiv) and phenyl ethynyl benziodoxolone (**2a**) (174 mg, 500 µmol, 2.50 equiv). The crude mixture was purified by column chromatography on Biotage (Büchi flashpure cartridge 25 g, gradient of pentane: ethyl acetate from 95:5 to 90:10) affording 2-(4-methoxyphenyl)-4-phenylbut-3-yn-1-yl 2-iodobenzoate (**8b**) (42.6 mg, 88.3 µmol, 44% yield) as colorless oil. **Rf** = 0.33 (SiO_2, 20:1 pentane:ethyl acetate). **^{1}H NMR** (400 MHz, $CDCl_3$) δ 7.99 (dd, *J* =7.9, 1.2 Hz, 1H, Ar*H*), 7.81 (dd, *J* =7.8, 1.7 Hz, 1H, Ar*H*), 7.49 – 7.42 (m, 4H, Ar*H*), 7.37 (td, *J* =7.6, 1.2 Hz, 1H, Ar*H*), 7.33 – 7.27 (m, 3H, Ar*H*), 7.15 (td, *J* =7.7, 1.7 Hz, 1H, Ar*H*), 6.95 – 6.88 (m, 2H, Ar*H*), 4.63 – 4.51 (m, 2H, OCH_2CH), 4.34 (t, *J* =7.2 Hz, 1H, OCH_2CHCC). 3.82 (s, 3H, OCH_3). **^{13}C NMR** (101 MHz, $CDCl_3$) δ 166.2, 159.2, 141.6, 134.8, 132.9, 131.9, 131.3, 129.7, 129.3, 128.4, 128.3, 128.0, 123.3, 114.3, 94.4, 88.2, 84.4, 69.1, 55.5, 37.6. **IR** (IR (ν_{max}, cm^{-1}) 3058 (w), 2954 (w), 2836 (w), 2061 (w), 1728 (s), 1610 (m), 1583 (m), 1511 (s), 1463 (m), 1307 (m), 1287 (m), 1246 (s), 1178 (m), 1132 (m), 1098 (m), 1033 (m), 1013 (m), 830 (m), 758 (m), 740 (s). **HRMS** (APPI/LTQ-Orbitrap) m/z: $[M + H]^+$ Calcd for $C_{24}H_{20}IO_3^+$ 483.0452; Found 483.0460.

2-(4-(*Tert*-butyl)phenyl)-4-phenylbut-3-yn-1-yl 2-iodobenzoate (8c)

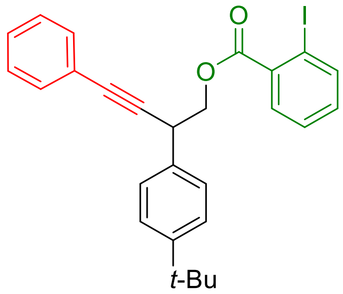

Following the general procedure C, starting from 1-*tert*-butyl-4-ethenylbenzene (**7c**) (32.1 mg, 200 µmol, 1.00 equiv) and phenyl ethynyl benziodoxolone (**2a**) (174 mg, 500 µmol, 2.50 equiv). The crude mixture was purified by column chromatography on Biotage (Büchi flashpure cartridge 25 g, gradient of pentane: ethyl acetate from 99:1 to 90:10) affording 2-(4-(*tert*-butyl)phenyl)-4-phenylbut-3-yn-1-yl 2-iodobenzoate (**8c**) (48.8 mg, 96.0 µmol, 48% yield) as colorless oil. **Rf** = 0.55 (SiO_2, 10:1 pentane:ethyl acetate). **^{1}H NMR :** (400 MHz, $CDCl_3$) δ 7.99 (dd, *J* =7.9, 1.2 Hz, 1H, Ar*H*), 7.80 (dd, *J* =7.8, 1.7 Hz, 1H, Ar*H*), 7.47 (d, *J* =1.9 Hz, 1H, Ar*H*), 7.46-7.44 (m, 2H, Ar*H*), 7.42 (s, 1H, Ar*H*), 7.40 – 7.34 (m, 3H, Ar*H*), 7.32 – 7.29 (m, 3H, Ar*H*), 7.18 – 7.13 (m, 1H, Ar*H*), 4.59 (d, *J* =7.2 Hz, 2H, OCH_2CH_2), 4.38 (t, *J* =7.2 Hz, 1H, CCHCH_2), 1.33 (s, 9H, $C(CH_3)_3$). **^{13}C NMR** (101 MHz, $CDCl_3$) δ 166.2, 150.8, 141.5, 134.9, 134.6, 132.9, 131.9, 131.4, 128.4, 128.2, 128.0, 127.9, 125.8, 123.4, 94.4, 88.2, 84.4, 69.1, 37.9, 34.7, 31.5. **IR** (ν_{max}, cm^{-1}) 2954 (m), 2868 (w), 2200 (w), 1729 (s), 1463 (m), 1288 (s), 1269 (s), 1249 (s), 1134 (s), 1100 (s), 1017 (m), 759 (s), 740 (s). **HRMS** (APPI/LTQ-Orbitrap) m/z: $[M + H]^+$ Calcd for $C_{27}H_{26}IO_2^+$ 509.0972; Found 509.0986.

4-(1-(4-methoxy-2,6-dimethylphenyl)cyclopropyl)-5-phenyl-2H-1,2,3-triazole (4aa)

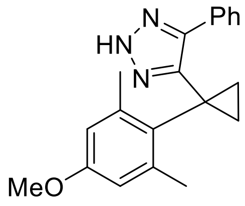

Following a reported procedure,[20] in a 12*75 mm Borosilicate glass tube, evacuated and backfilled with nitrogen 3 times, under a nitrogen atmosphere, a solution of alkynylcyclopropane **4a** (27.6 mg, 0.100 mmol, 1.00 equiv) in anhydrous acetonitrile (2.0 mL) was added followed by the addition of NaN_3 (9.80 mg, 0.150 mmol, 1.50 equiv) and $PhI(OAc)_2$ (32.2 mg, 0.100 mmol, 1.00 equiv). The reaction mixture was stirred at room temperature for

12 h. Upon completion of the reaction as indicated by TLC (40:1 pentane:ethyl acetate), the reaction mixture was concentrated under vacuum and purified by flash column chromatography (SiO_2, 4:1 pentane:ethyl acetate) to afford 4-(1-(4-methoxy-2,6-dimethylphenyl)cyclopropyl)-5-phenyl-2H-1,2,3-triazole (**4aa**) (21.3 mg, 0.067 mmol, 67% yield) as colorless oil. **Rf** = 0.18 (SiO_2, 4:1 pentane:ethyl acetate). **^{1}H NMR** (400 MHz, $CDCl_3$) δ 7.26 – 7.22 (m, 1H, Ar*H*), 7.19 – 7.14 (m, 2H, Ar*H*), 6.98 – 6.94 (m, 2H, Ar*H*), 6.38 (s, 2H, Ar*H*), 3.71 (s, 3H, ArOC*H₃*), 2.19 (s, 6H, ArC*H₃*), 1.68 – 1.61 (m, 2H, C*H₂*), 1.17 – 1.10 (m, 2H, C*H₂*). **^{13}C NMR** (101 MHz, $CDCl_3$) δ 158.5, 140.6, 131.4, 130.4, 129.7, 128.2, 127.8, 127.6, 113.7, 55.2, 21.1, 20.3, 18.9. One aromatic carbon not resolved. **IR** (ν_{max}, cm^{-1}) 2928 (m), 2247 (w), 2105 (m), 1603 (s), 1319 (s), 1162 (s), 1058 (s), 1034 (s), 1016 (s), 912 (m). **HRMS** (ESI/QTOF) m/z: $[M + H]^+$ Calcd for $C_{20}H_{22}N_3O^+$ 320.1757; Found 320.1761.

5-Benzyl-4-(4-methoxy-2,6-dimethylphenyl)-2,3-dihydrothiophene (4ab)

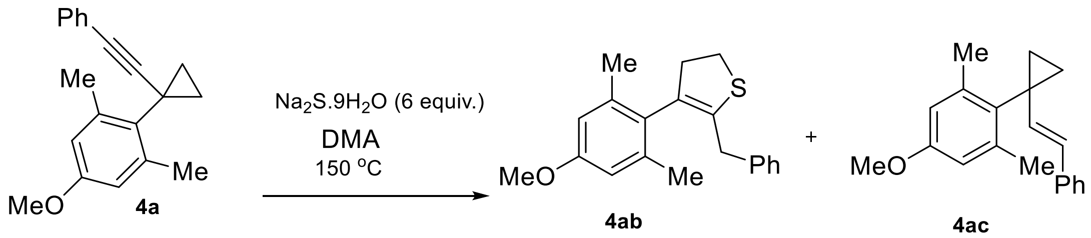

Following a reported procedure,[20] in a 12*75 mm Borosilicate glass tube, evacuated and backfilled with nitrogen 3 times, under a nitrogen atmosphere, a solution of alkynylcyclopropane **4a** (27.6 mg, 0.100 mmol, 1.00 equiv)and $Na_2S·9H_2O$ (0.14 g, 0.60 mmol, 6.00 equiv) in DMA (0.5 mL) was added. The reaction mixture was stirred at 150 °C for 12 h. Upon completion of the reaction as indicated by TLC (40:1 pentane:ethyl acetate),, ethyl acetate was added. The solution was washed with water and extracted with ethyl acetate. The organic layers were combined, dried over sodium sulfate, concentrated under vacuum and purified by flash column chromatography to afford the compound 5-benzyl-4-(4-methoxy-2,6-dimethylphenyl)-2,3-dihydrothiophene **4ab** (13.0 mg, 0.0419 mmol, 42% yield) and (E)-5-methoxy-1,3-dimethyl-2-(1-styrylcyclopropyl)benzene **4ac** (8.90 mg, 0.032 mmol, 32% yield).

5-benzyl-4-(4-methoxy-2,6-dimethylphenyl)-2,3-dihydrothiophene (4ab)

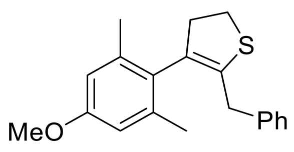

Rf = 0.50 (SiO_2, 40:1 pentane:ethyl acetate). **^{1}H NMR** (400 MHz, $CDCl_3$) δ 7.27 – 7.22 (m, 2H, Ar*H*), 7.21 – 7.16 (m, 1H, Ar*H*), 7.11 – 7.06 (m, 2H, Ar*H*), 6.63 (s, 2H, Ar*H*), 3.79 (s, 3H, ArOC*H₃*), 3.30 (s, 2H, PhC*H₂*), 3.27 (t, *J* =8.4 Hz, 2H, SC*H₂*), 2.91 (tt, *J* =8.4, 1.5 Hz, 2H, SCH_2CH_2), 2.18 (s, 6H, ArCH_3). **^{13}C NMR** (101 MHz, $CDCl_3$) δ 158.6, 138.8, 138.3, 135.8, 129.8, 128.8, 128.8, 128.4, 126.4, 112.9, 55.2, 40.6, 35.5, 31.1, 20.3. **IR** ($ν_{max}$, cm^{-1}) 2934 (m), 2834 (m), 1604 (s), 1489 (m), 1320 (s), 1193 (m), 1153 (s), 1072 (s), 855 (m). **HRMS** (Sicrit plasma/LTQ-Orbitrap) m/z: $[M + H]^+$ Calcd for $C_{20}H_{23}OS^+$ 311.1464; Found 311.1458.

(E)-5-Methoxy-1,3-dimethyl-2-(1-styrylcyclopropyl)benzene (4ac)

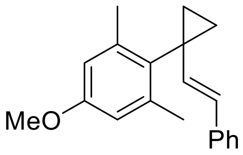

Rf = 0.56 (SiO_2, 40:1 pentane:ethyl acetate). **^{1}H NMR** (400 MHz, $CDCl_3$) δ 7.24 (d, *J* =1.3 Hz, 4H, Ar*H*), 7.14 (ddt, *J* =6.1, 4.5, 3.2 Hz, 1H, Ar*H*), 6.62 (s, 2H, Ar*H*), 5.84 (d, *J* =15.7 Hz, 1H, PhC*H*), 5.78 (d, *J* =15.8 Hz, 1H, PhCHC*H*), 3.80 (s, 3H, ArOC*H₃*), 2.34 (s, 6H, ArCH_3), 1.24 – 1.18 (m, 2H, CH_2), 1.12 – 1.06 (m, 2H, CH_2). **^{13}C NMR** (101 MHz, $CDCl_3$) δ 158.1, 140.6, 137.9, 137.0, 131.2, 128.6, 126.9, 126.6, 125.8, 113.5, 55.2, 24.6, 20.9, 18.4. **IR** (ν_{max}, cm^{-1}) 2998 (m), 2954 (m), 2836 (w), 1640 (m), 1603 (s), 1488 (s), 1449 (m), 1314 (s), 1193 (m), 1159 (s), 1106 (m), 1060 (s), 965 (m), 856 (m), 745 (s). **HRMS** (Sicrit plasma/LTQ-Orbitrap) m/z: $[M + H]^+$ Calcd for $C_{20}H_{23}O^+$ 279.1743; Found 279.1740.

Computational Details.

An initial pool of conformers for each C-H and C-C activation transition state were generated using CREST[21] with frozen bond distances corresponding to those bonds formed/broken at the TS. For each substrate/TS combination, ten unique conformers were generated. The geometries of each of these species was optimized at the ωB97X-D[22]/def2-SVPD[23] level in implicit chloroform solvent using the SMD solvation model[24] as implemented in Gaussian16.[25] Single point energies were then obtained on the optimized ωB97X-D/def2-SVPD geometries at the DLPNO-CCSD(T)[26]/def2-TZVP level using TightPNO[27] as implemented in Orca.[28] TS structures having the lowest energies at the DLPNO-CCSD(T)/def2-TZVP//ωB97X-D/def2-SVPD level were used for further analysis of the reaction pathways reported in the main text. All species were subsequently characterized as either minima (zero imaginary frequencies) or transition states (one imaginary frequency) by examining the vibrational frequencies. Free energy corrections were established using the rigid-rotor harmonic oscillator model and corrected for translational entropy in solution using the method of Martin, Hay, and Pratt[29] (12.47 mol/L for chloroform) in the GoodVibes package.[30] Reported free energies are include the sum of electronic energies at the DLPNO-CCSD(T)/def2-TZVP//ωB97X-D/def2-SVPD level with solvation and free energy corrections at the ωB97X-D/def2-SVPD level.

Rotational profiles of substrates **1a** and **1c** were obtained by constraining the C-C-C-H dihedral angle (see below) and partially optimizing the geometry at the ωB97X-D/def2-SVPD level. The plots shown in Figure S1 are electronic energies.

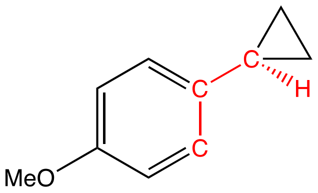

1a

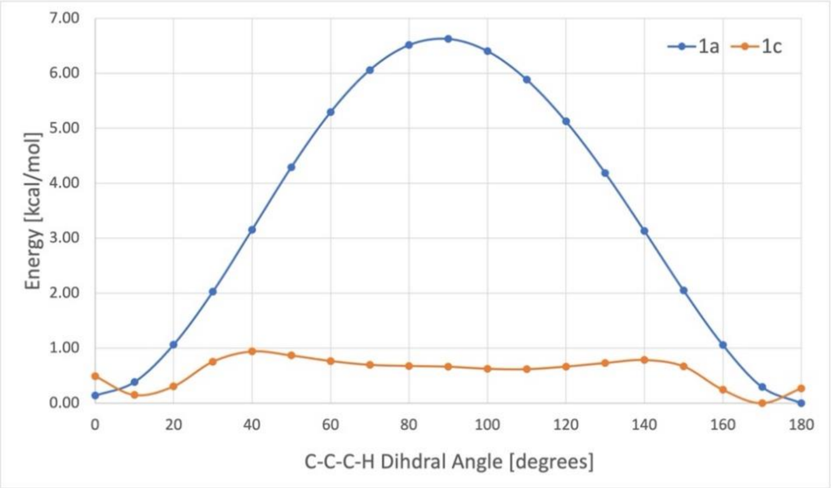

Figure S1. Rotational profiles of **1a** and **1c**. Computations at the ωB97X-D/def2-SVPD level.

Table S1. Relative free energies (from separated reactants) of relevant species. Reported free energies include single point electronic energies at the DLPNO-CCSD(T)/def2-TZVP//ωB97X-D/def2-SVPD level as well as free energy and solvation corrections (SMD model in chloroform) at the ωB97X-D/def2-SVPD level. Values in kcal/mol.

	**Substrate 1a**	**Substrate 1c**
CH – Reaction Complex	-3.65	-7.58
CH – Transition State	9.60	-7.95[a]
CH – Product Complex	-24.34	-28.34
CC – Reaction Complex	-10.59	-7.96
CC – Transition State	-2.04	-1.76
CC – Product Complex	-40.90	-39.93

a) The electronic energy of this transition state lies 2.55 kcal/mol above the reaction complex at the ωB97X-D/def2-SVPD (optimization) level.

Table S2. Electronic energies, free energy corrections, and solvation corrections of relevant species. DLPNO-CCSD(T)/def2-TZVP electronic energies were computed on optimized geometries at the ωB97X-D/def2-SVPD level. Values in Hartree.

	ωB97X-D/def2-SVPD Electronic Energies	ωB97X-D/def2-SVPD Free Energy Corrections	ωB97X-D/def2-SVPD Solvation Corrections	DLPNO-CCSD(T)/def2-TZVP//ωB97X-D/def2-SVPD Single Point Electronic Energies	Total Free Energy
Hypervalent Iodine – Anion	-717.097833	0.062295	-0.076164	-716.111566	-716.125435
**Substrate 1a**					
1a – Radical Cation	-462.810986	0.165593	-0.072085	-462.369607	-462.276099
I_{C-H}	-1179.944924	0.242751	-0.0490507	-1178.601057	-1178.407357
TS_{C-H}	-1179.935998	0.239369	-0.030426	-1178.595177	-1178.386234
II_{C-H}	-1179.960280	0.241498	-0.024318	-1178.657508	-1178.440328
I_{C-C}	-1179.946849	0.243449	-0.0467964	-1178.615057	-1178.418404
TS_{C-C}	-1179.931703	0.243275	-0.041447	-1178.606615	-1178.404787
II_{C-C}	-1179.991364	0.248053	-0.0250113	-1178.689757	-1178.466715
**Substrate 1c**					
1c – Radical Cation	-541.370297	0.219286	-0.069981	-540.855145	-540.705840
I_{C-H}	-1258.509751	0.296898	-0.042420	-1257.097840	-1256.843362
TS_{C-H}	-1258.505693	0.293706	-0.032572	-1257.105078	-1256.843944
II_{C-H}	-1258.527420	0.294619	-0.024339	-1257.146711	-1256.876431
I_{C-C}	-1258.506669	0.297009	-0.037950	-1257.103014	-1256.843955
TS_{C-C}	-1258.490202	0.296177	-0.043847	-1257.086407	-1256.834078
II_{C-C}	-1258.546794	0.300303	-0.026420	-1257.168792	-1256.894909

Table S3. Rotational profiles around the C-C-C-H dihedral angle for 1a and 1c. Computations at the ωB97X-D/def2-SVPD level.

Dihedral Angle	Electronic Energy **1a** (hartree)	Relative Energy **1a** (kcal/mol)	Electronic Energy **1c** (hartree)	Relative Energy **1c** (kcal/mol)
0	-462.7390033	0.14	-541.2998484	0.49
10	-462.7386084	0.38	-541.3003924	0.15
20	-462.7375261	1.06	-541.3001412	0.31
30	-462.7359897	2.03	-541.2994279	0.75
40	-462.7341918	3.16	-541.2991324	0.94

50	-462.7323808	4.29	-541.2992485	0.87
60	-462.7307856	5.29	-541.2994093	0.77
70	-462.7295661	6.06	-541.2995195	0.70
80	-462.7288425	6.51	-541.2995518	0.68
90	-462.728665	6.62	-541.2995728	0.66
100	-462.7290185	6.40	-541.2996335	0.63
110	-462.7298445	5.88	-541.2996453	0.62
120	-462.7310535	5.12	-541.2995705	0.66
130	-462.7325483	4.19	-541.2994670	0.73
140	-462.7342289	3.13	-541.2993799	0.78
150	-462.7359542	2.05	-541.2995641	0.67
160	-462.7375351	1.06	-541.3002406	0.24
170	-462.7387524	0.29	-541.3006297	0.00
180	-462.7392206	0.00	-541.3001999	0.27

NMR spectra

^{1}H-NMR (400 MHz, $CDCl_3$) (1b)

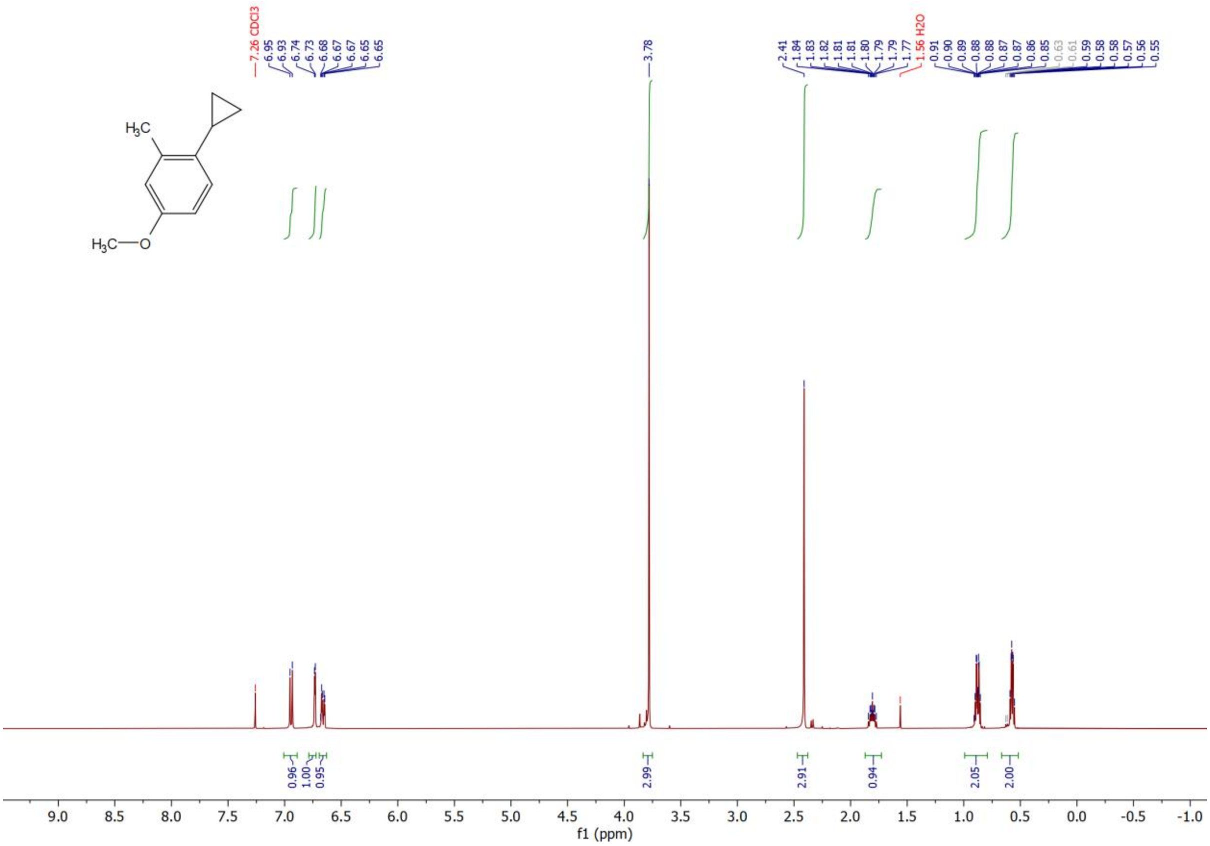

^{13}C NMR (101 MHz, $CDCl_3$) (1b)

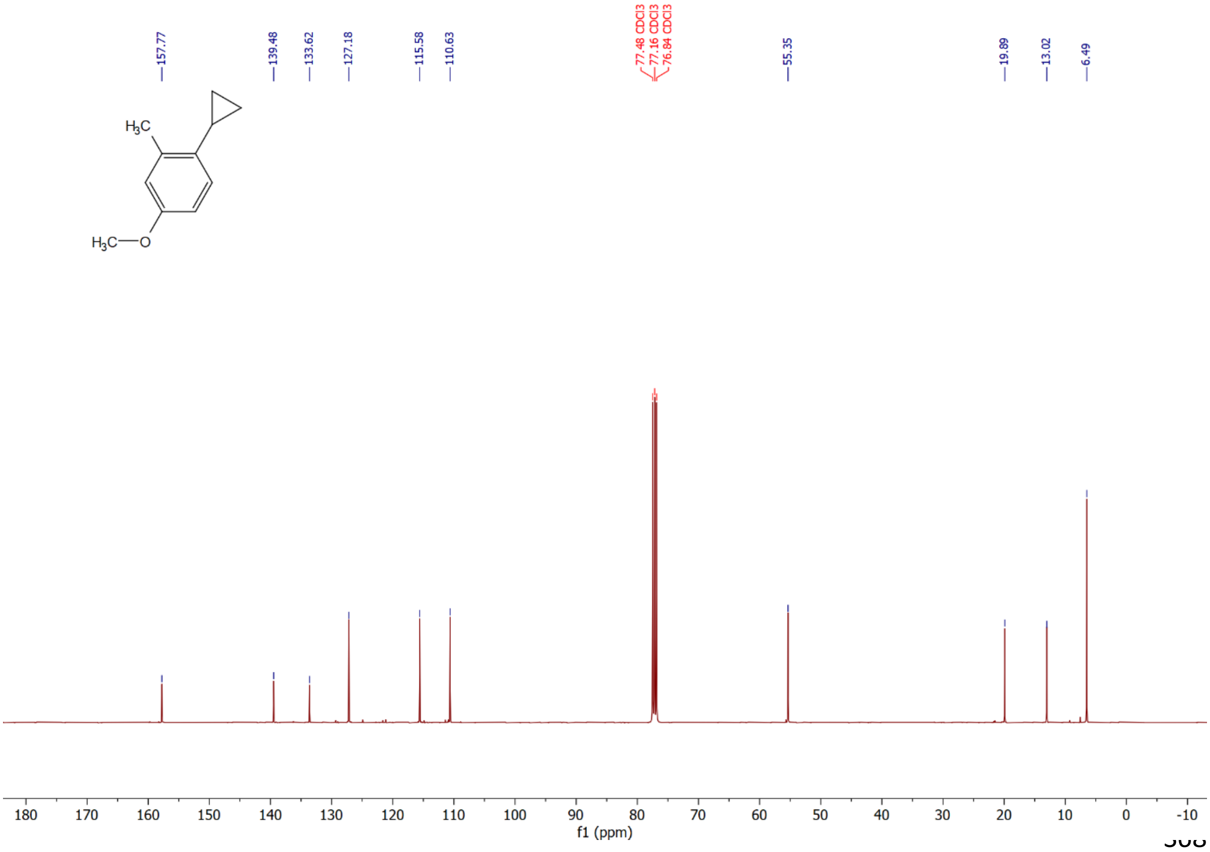

^{1}H-NMR (400 MHz, $CDCl_3$) (1c)

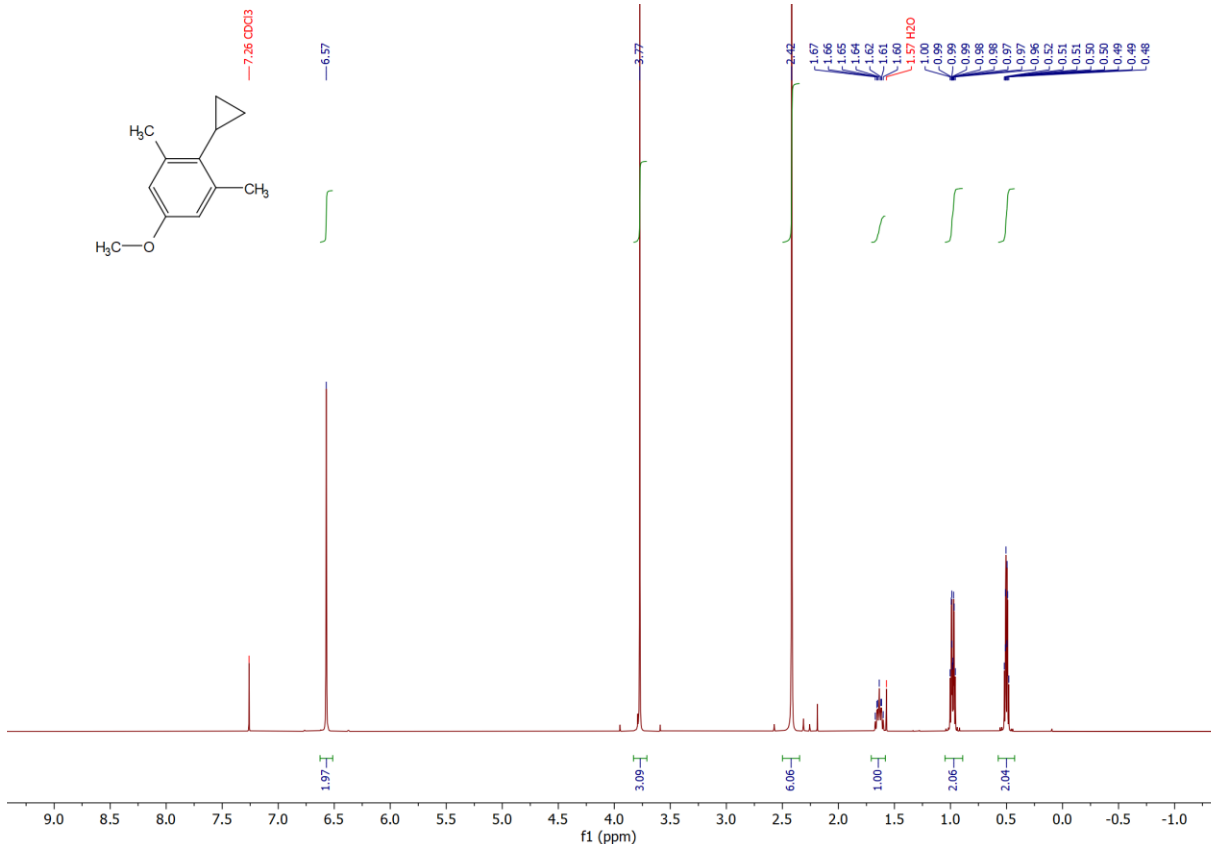

^{13}C NMR (101 MHz, $CDCl_3$) (1c)

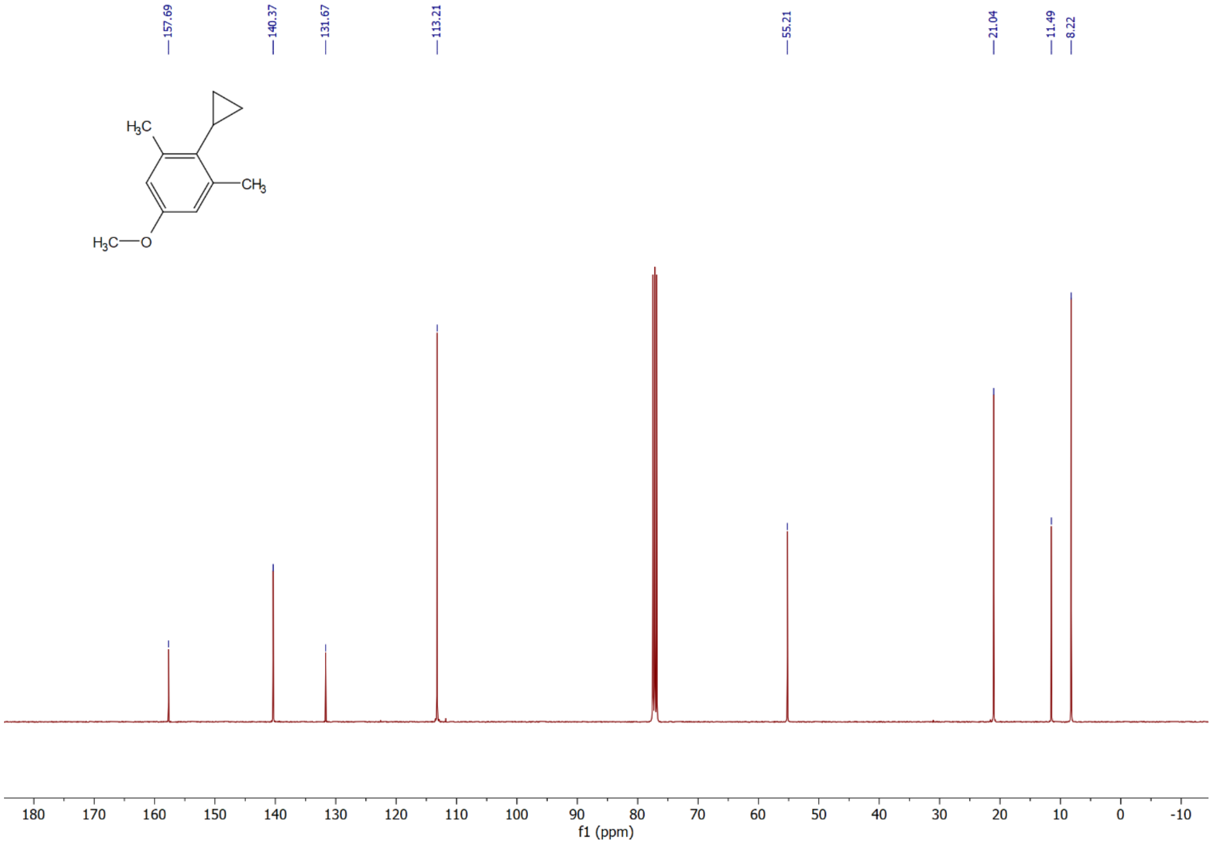

^{1}H-NMR (400 MHz, $CDCl_3$) (1f)

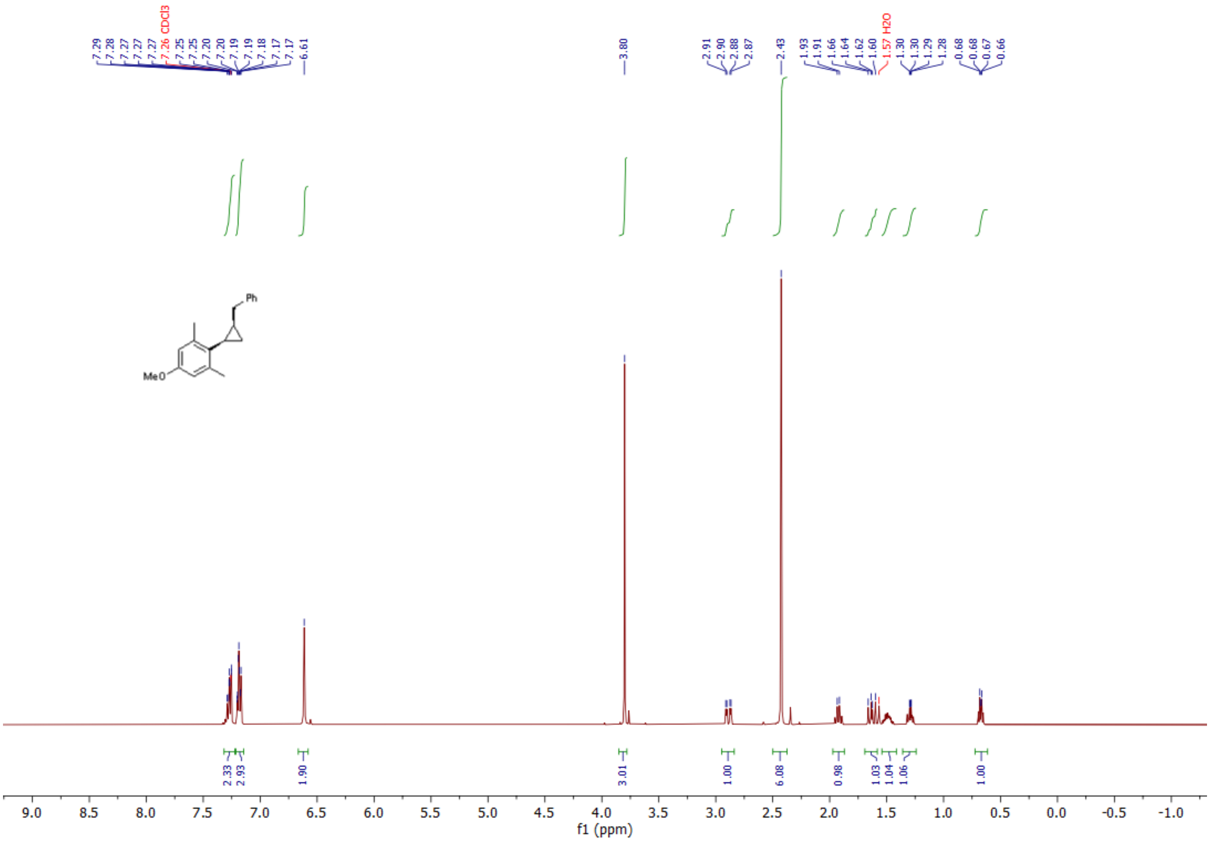

^{13}C NMR (101 MHz, $CDCl_3$) (1f)

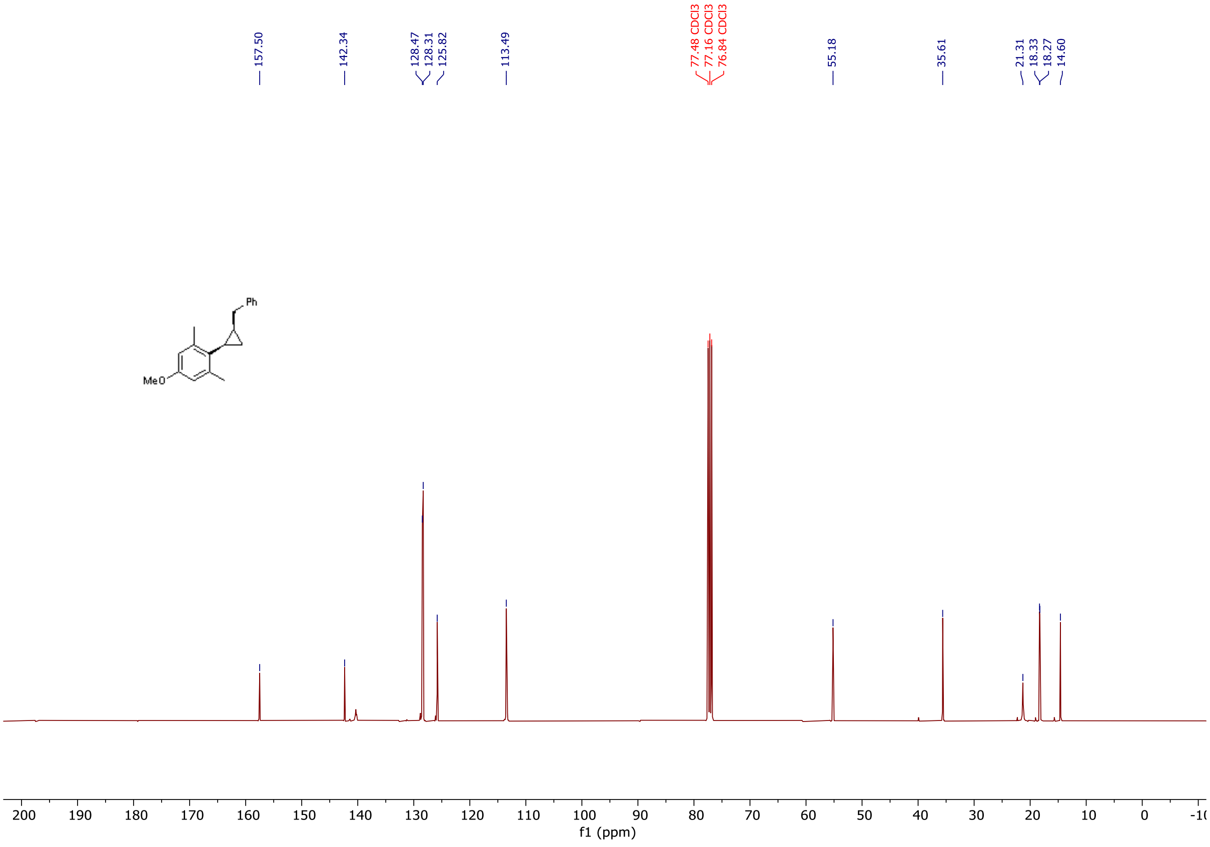

^{1}H-NMR (400 MHz, $CDCl_3$) (1g)

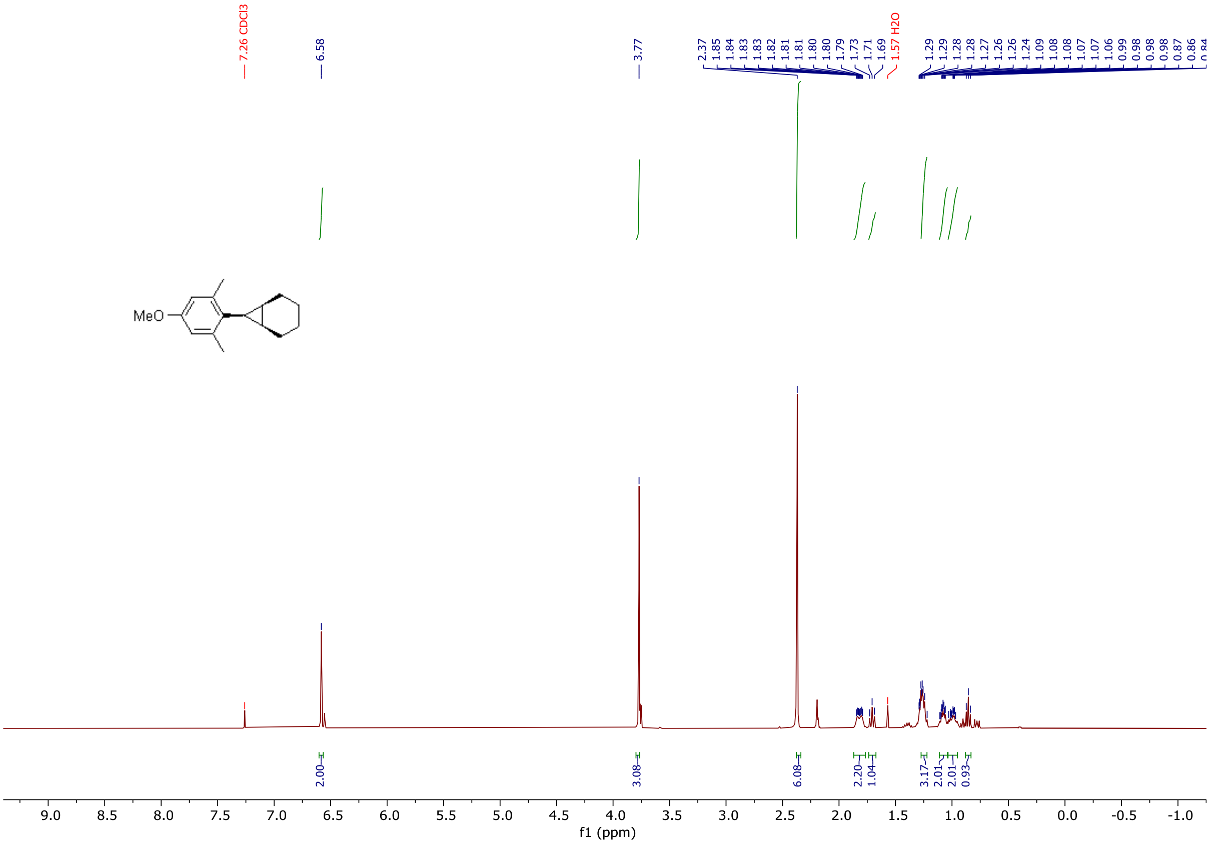

^{13}C NMR (101 MHz, $CDCl_3$) (1g)

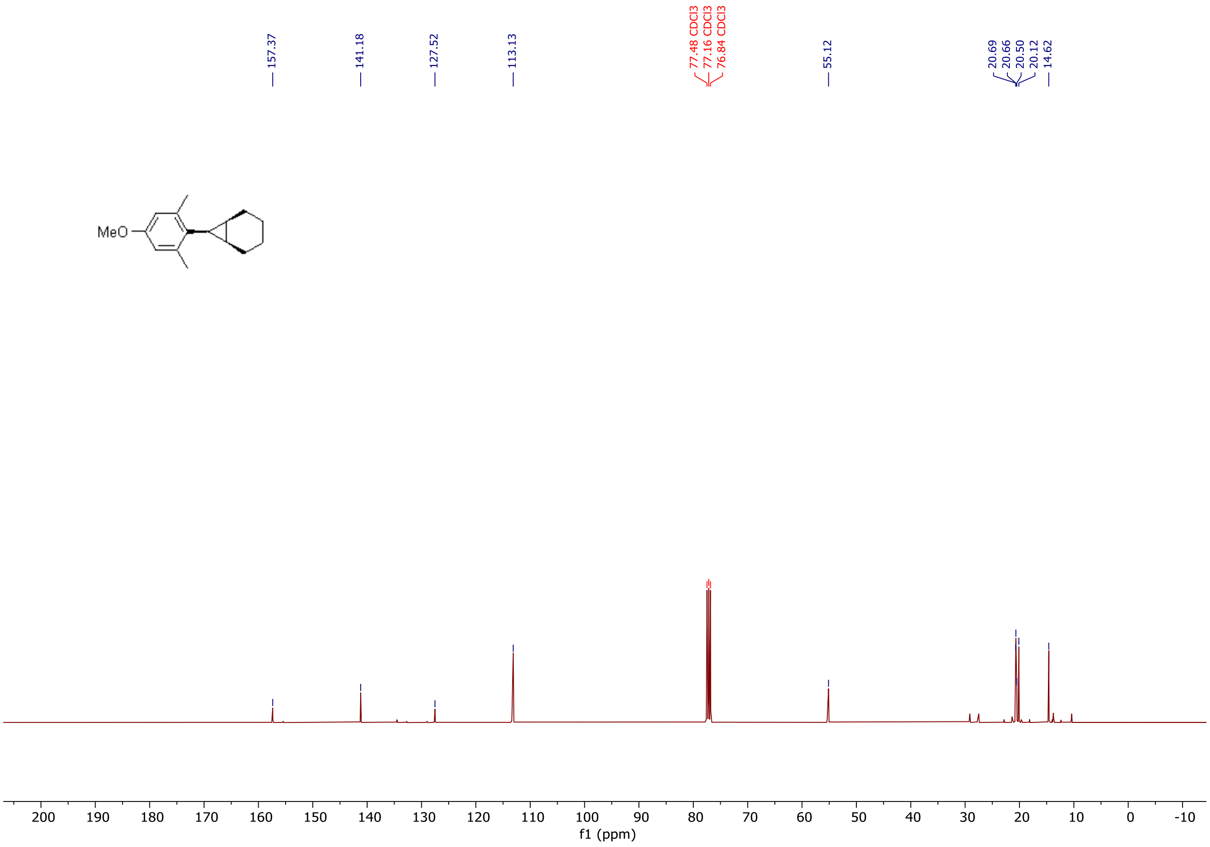

^{1}H-NMR (400 MHz, $CDCl_3$) (1h) Orange: Major diastereomer, Blue: Minor diastereomer

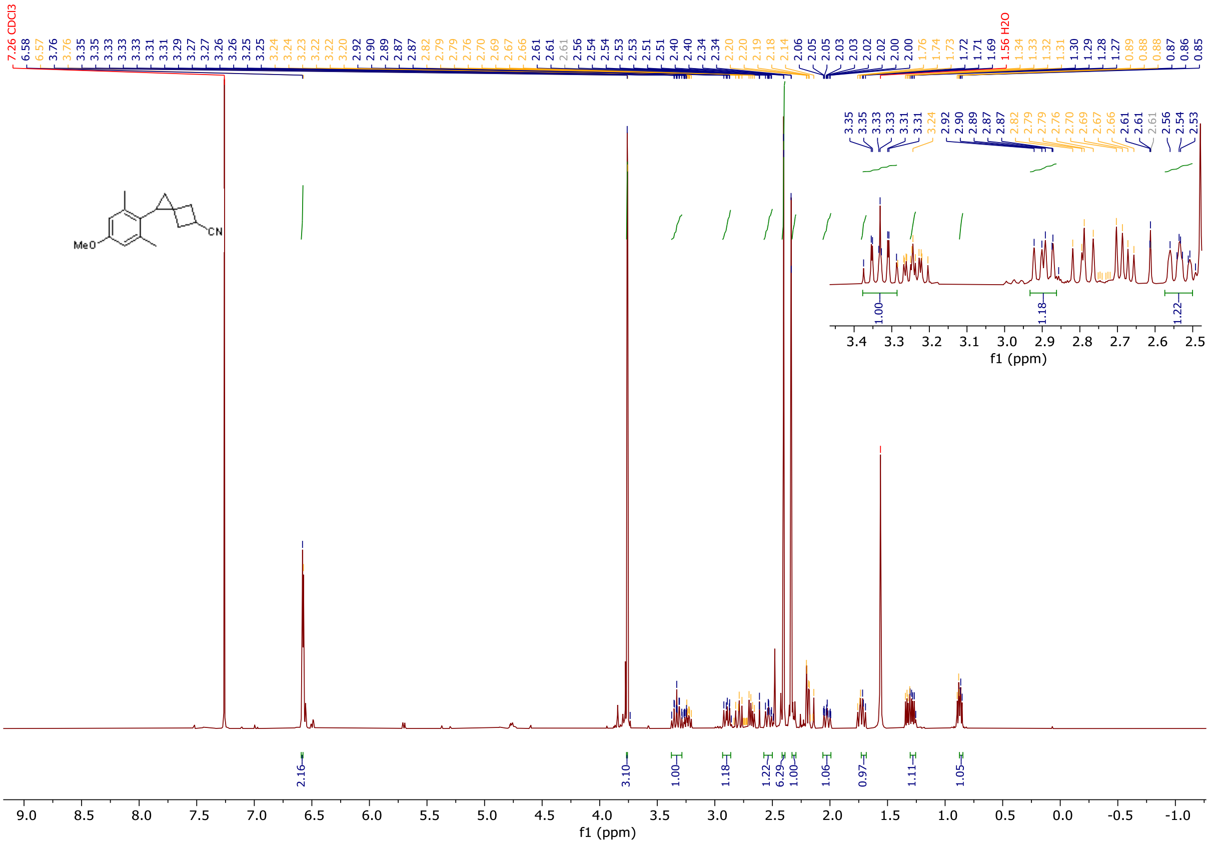

^{13}C NMR (101 MHz, $CDCl_3$) (1h)

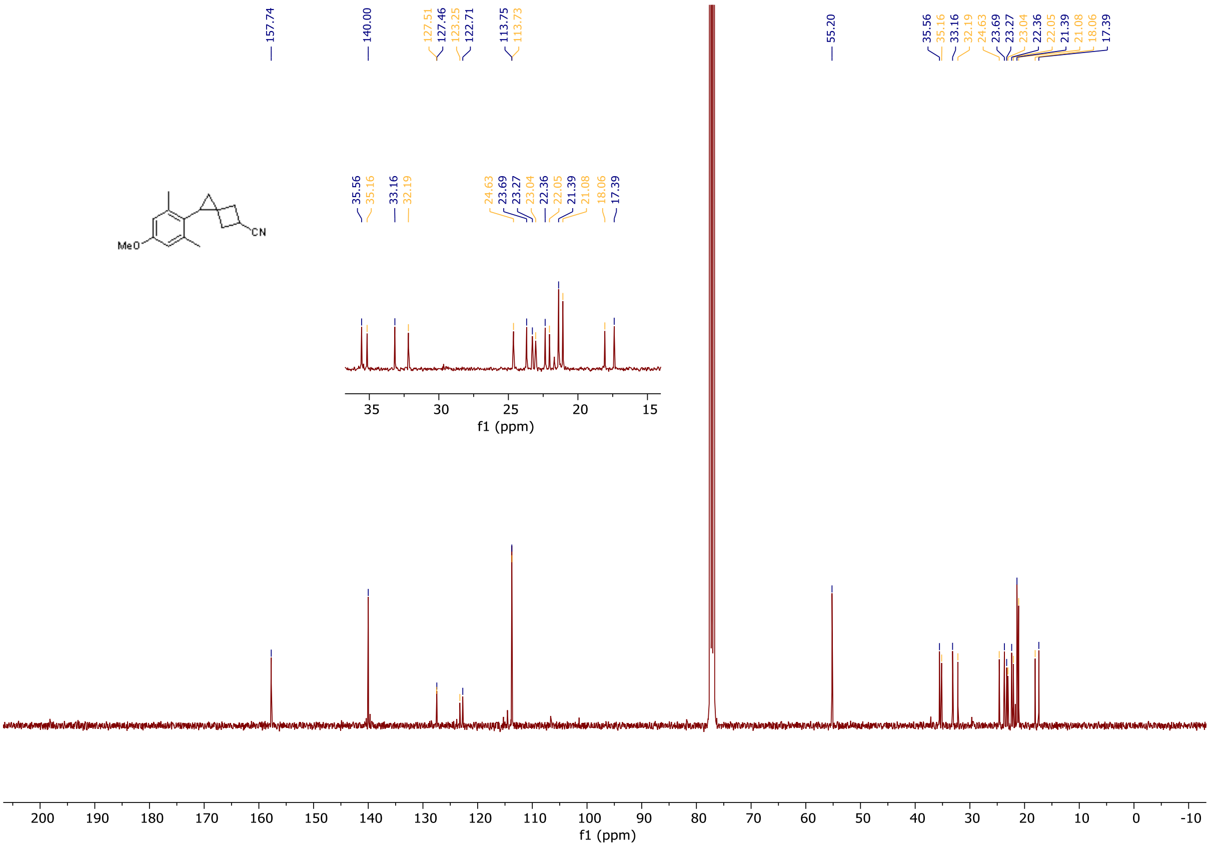

^{1}H-NMR (400 MHz, $CDCl_3$) (1i)

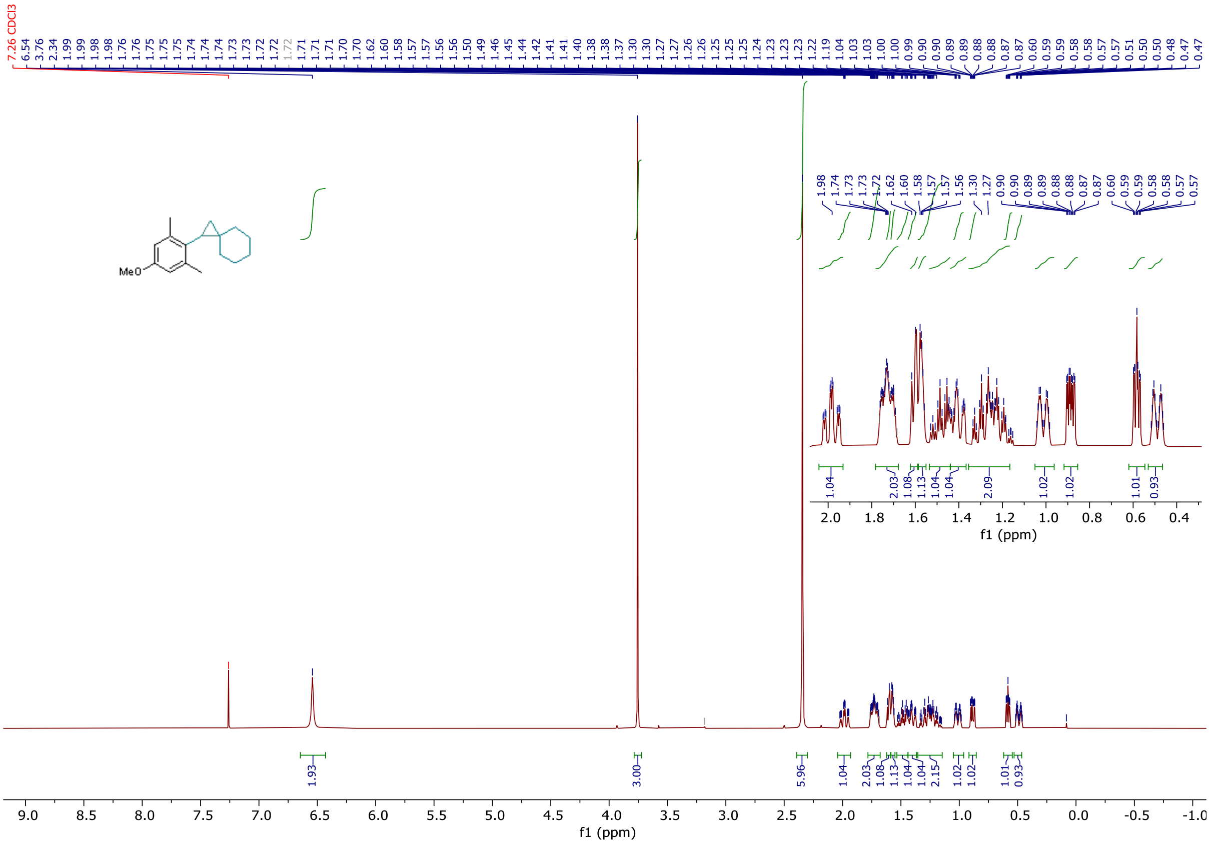

^{13}C NMR (101 MHz, $CDCl_3$) (1i)

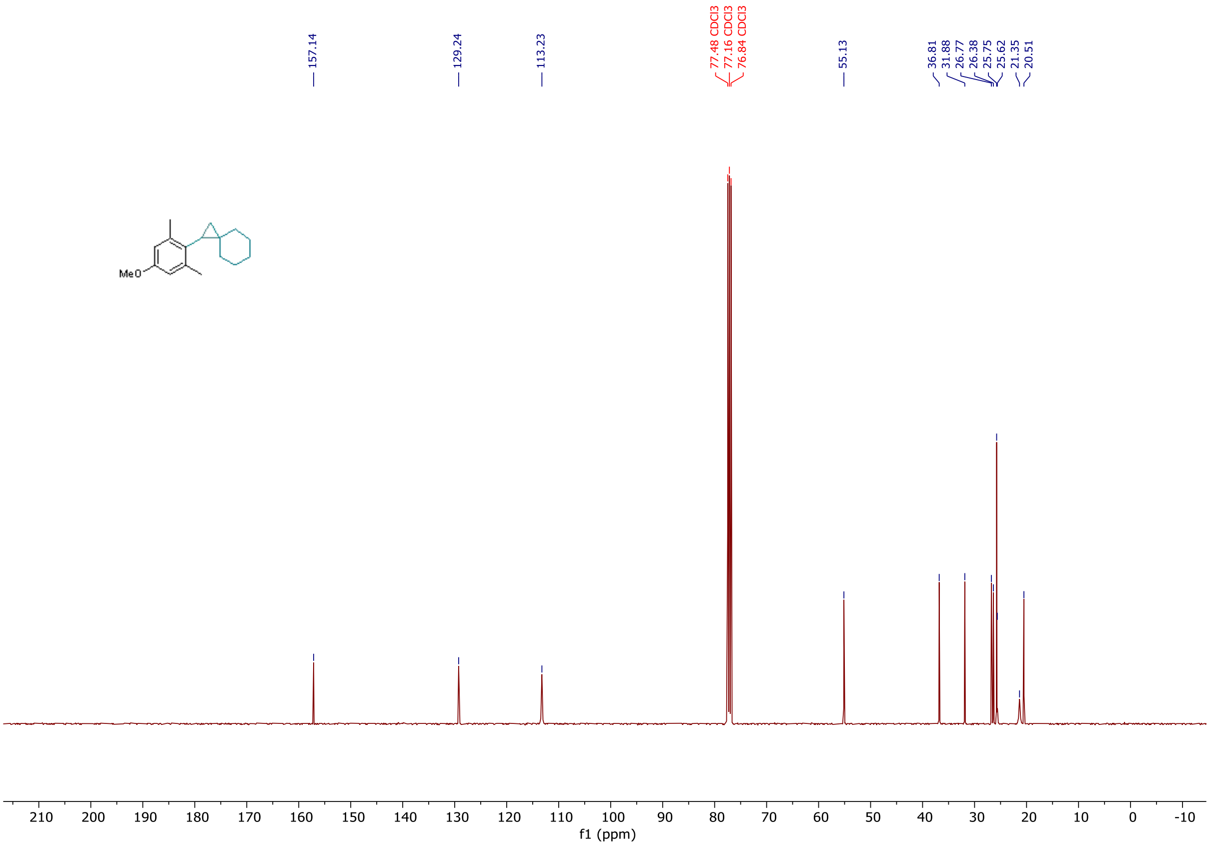

^{1}H-NMR (400 MHz, $CDCl_3$) (1k)

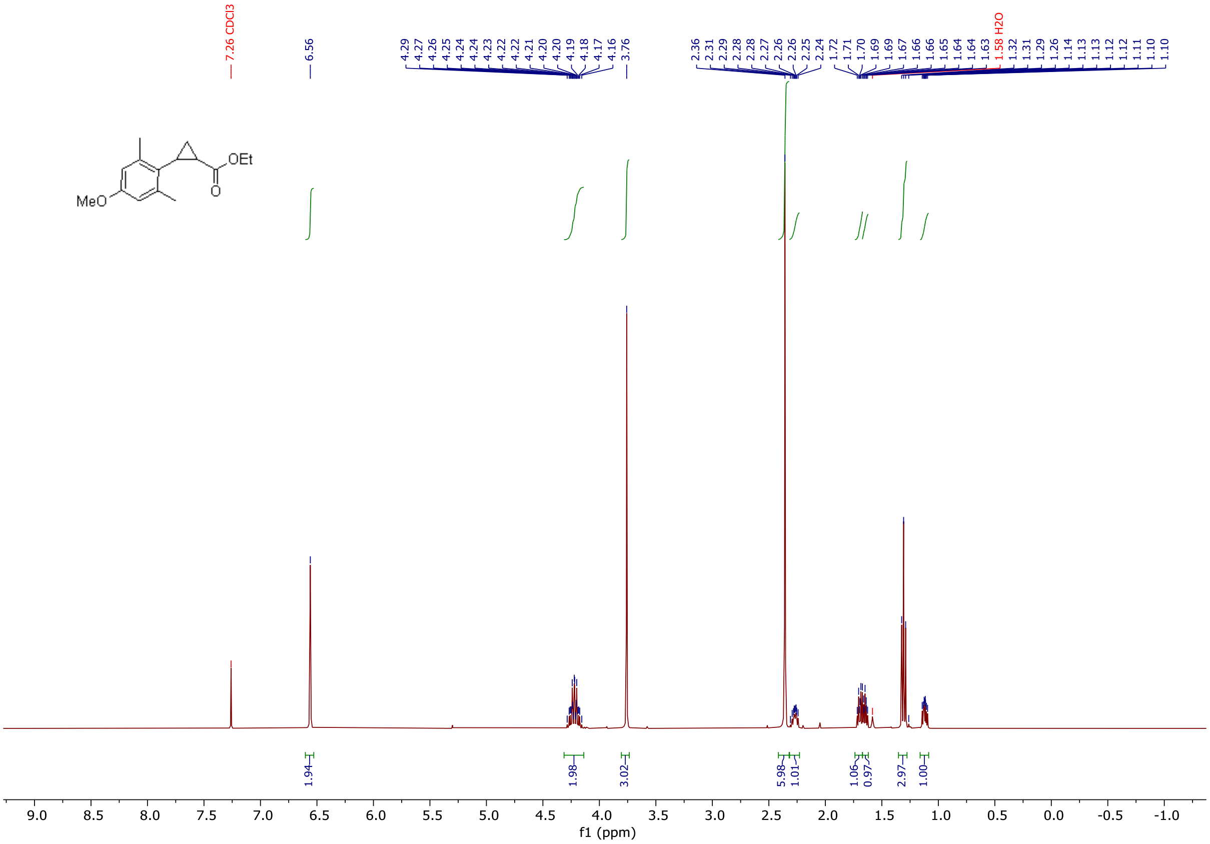

^{13}C NMR (101 MHz, $CDCl_3$) (1k)

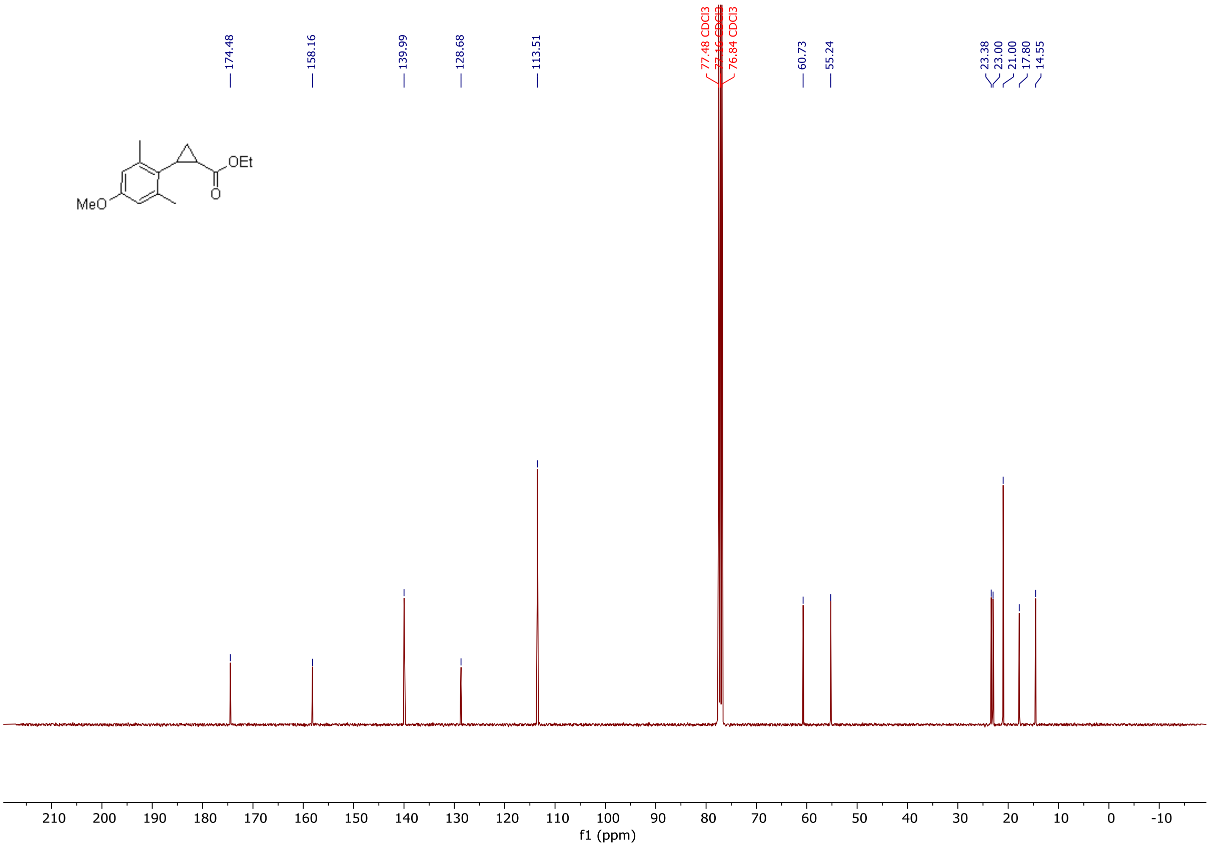

^{1}H-NMR (400 MHz, $CDCl_3$) (1l)

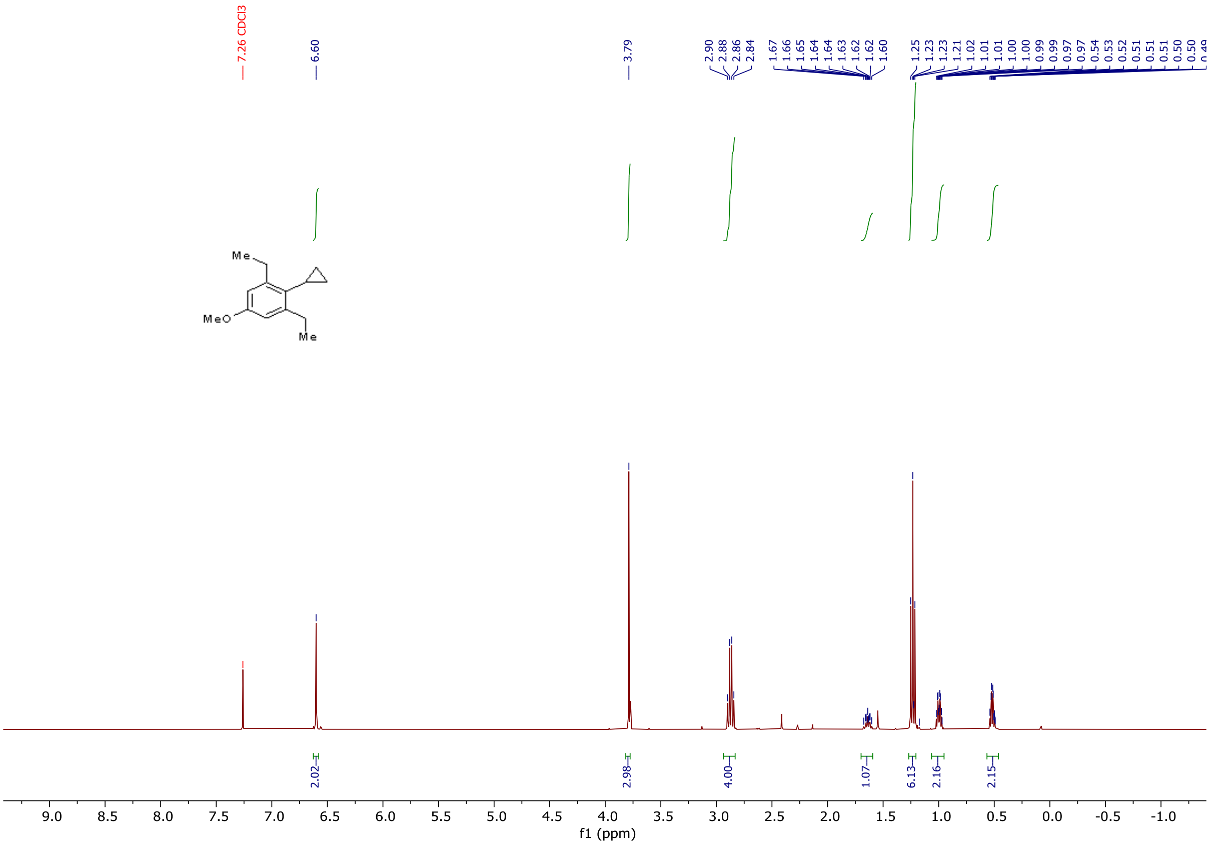

^{13}C NMR (101 MHz, $CDCl_3$) (1l)

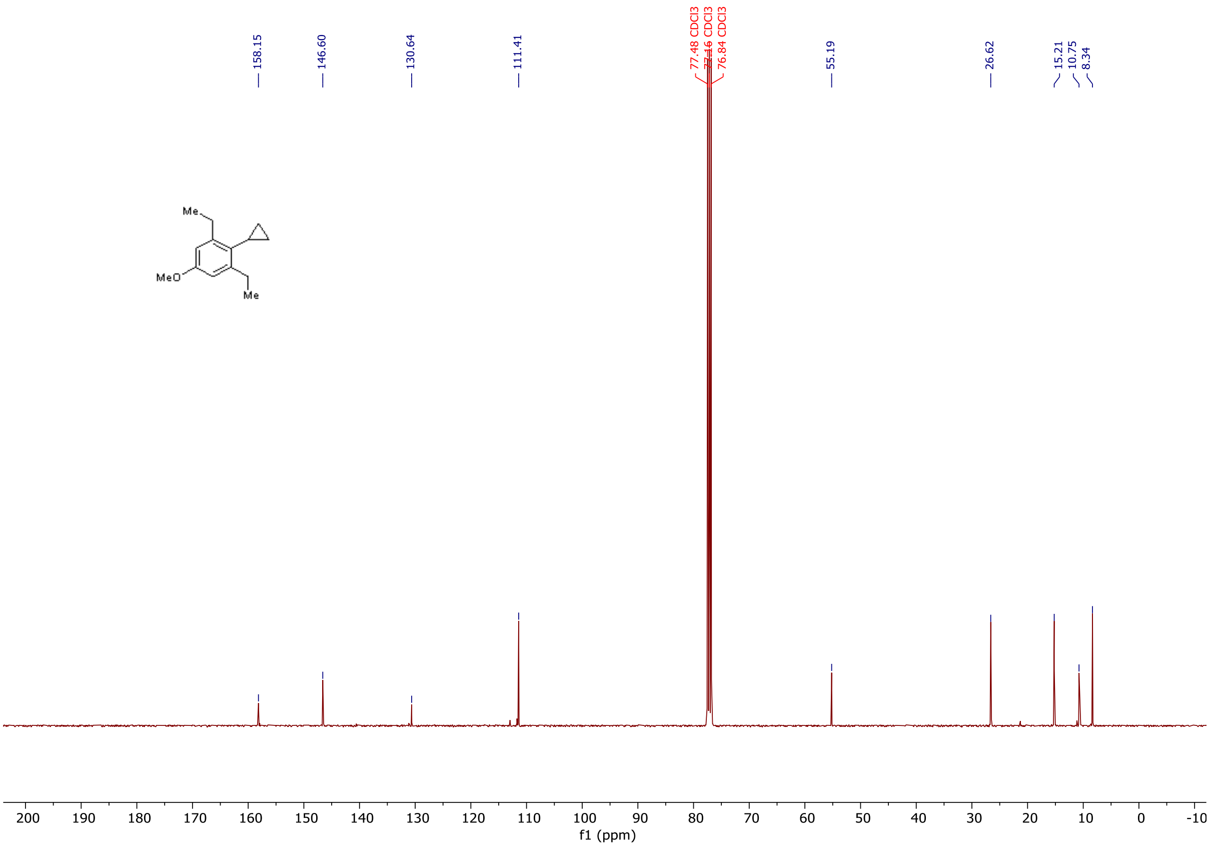

^{1}H-NMR (400 MHz, $CDCl_3$) (1m)

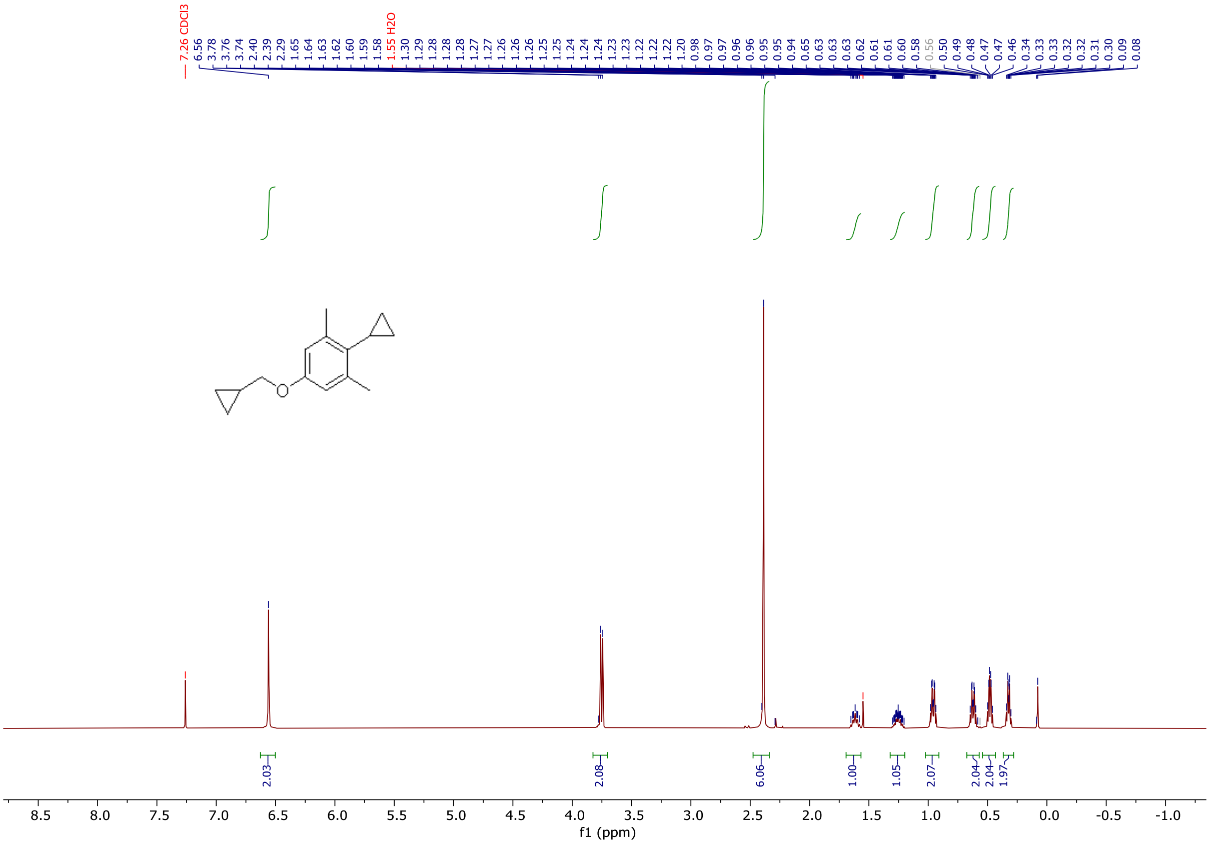

^{13}C NMR (101 MHz, $CDCl_3$) (1m)

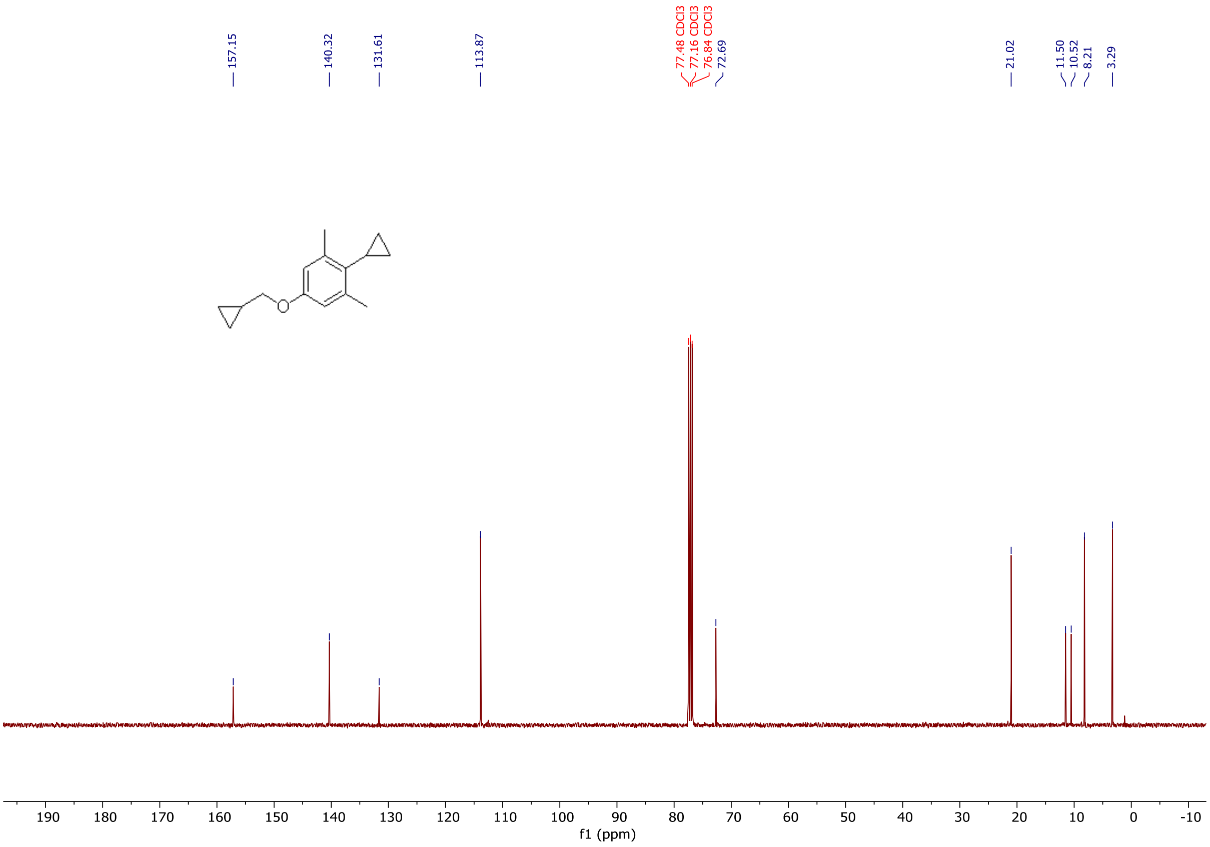

^{1}H-NMR (400 MHz, $CDCl_3$) (1n)

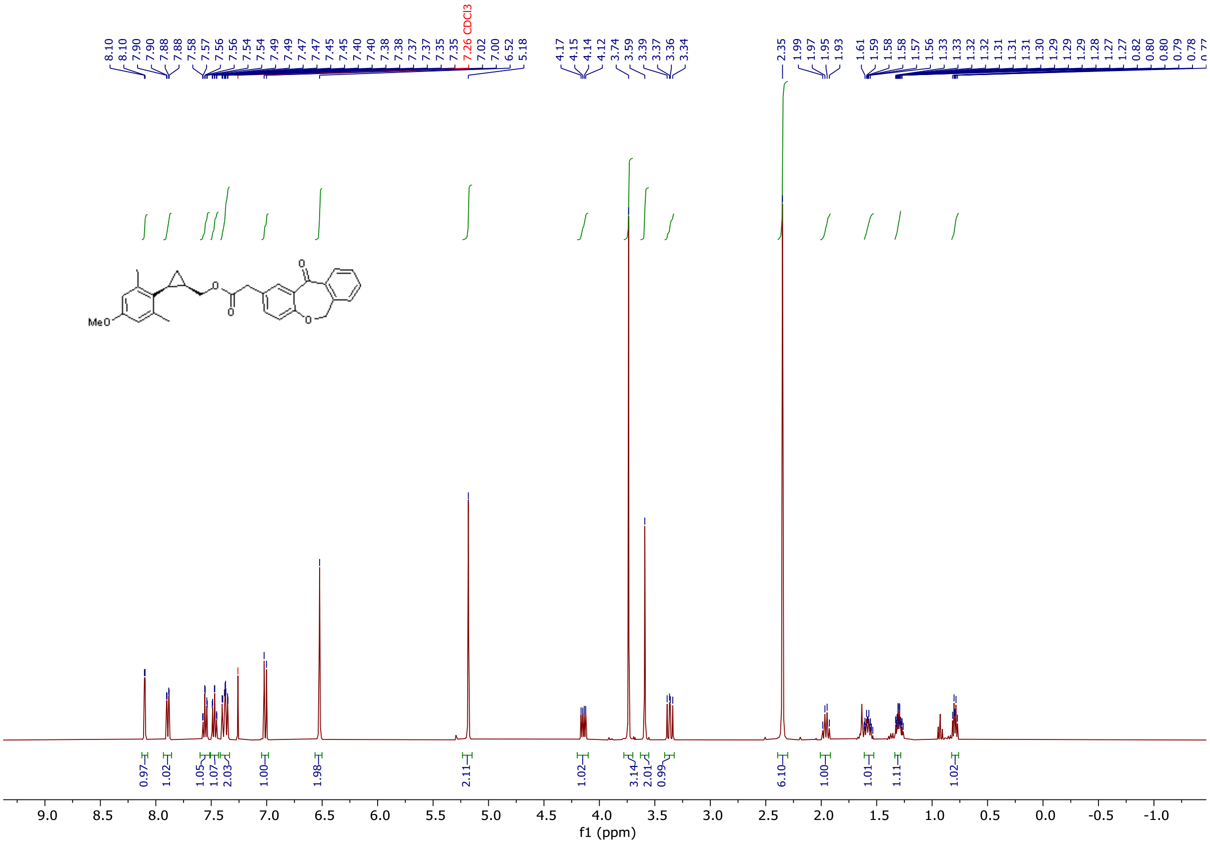

^{13}C NMR (101 MHz, $CDCl_3$) (1n)

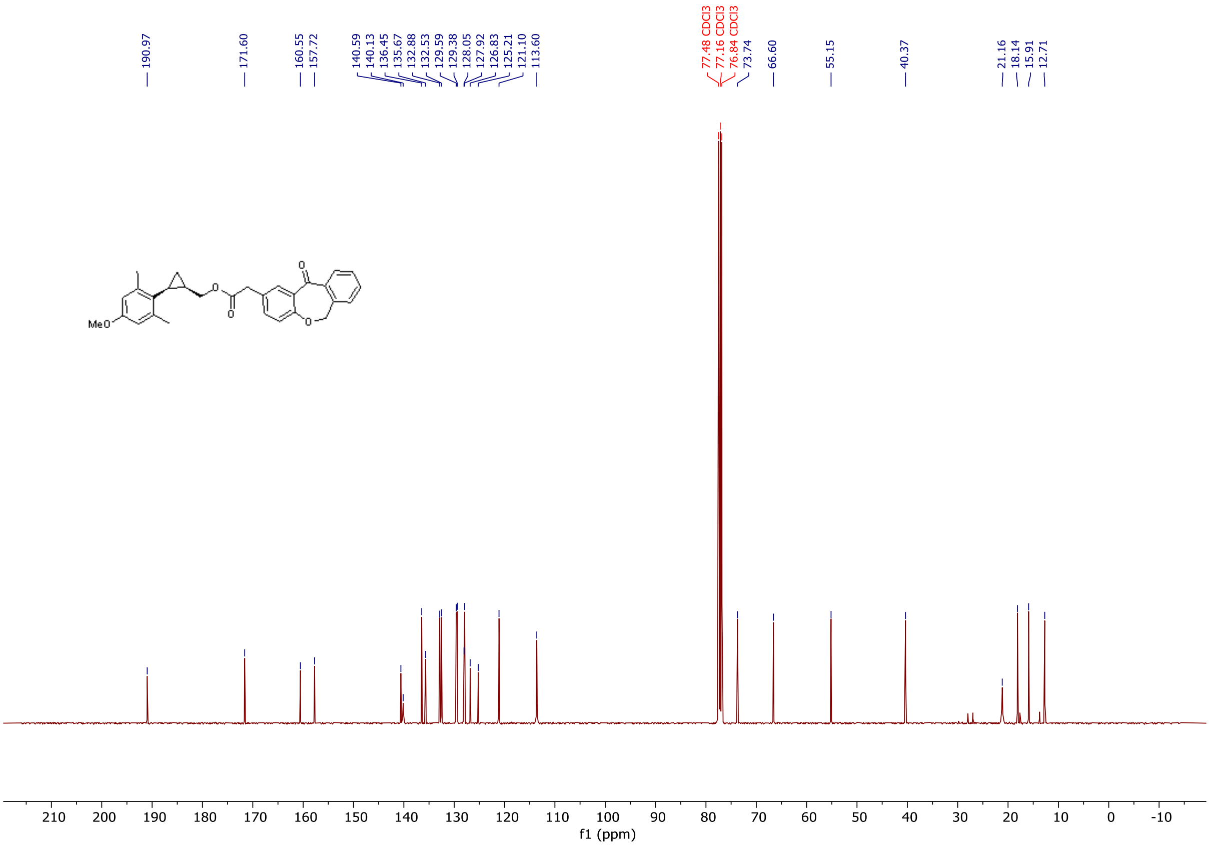

^{1}H-NMR (400 MHz, $CDCl_3$) (1p)

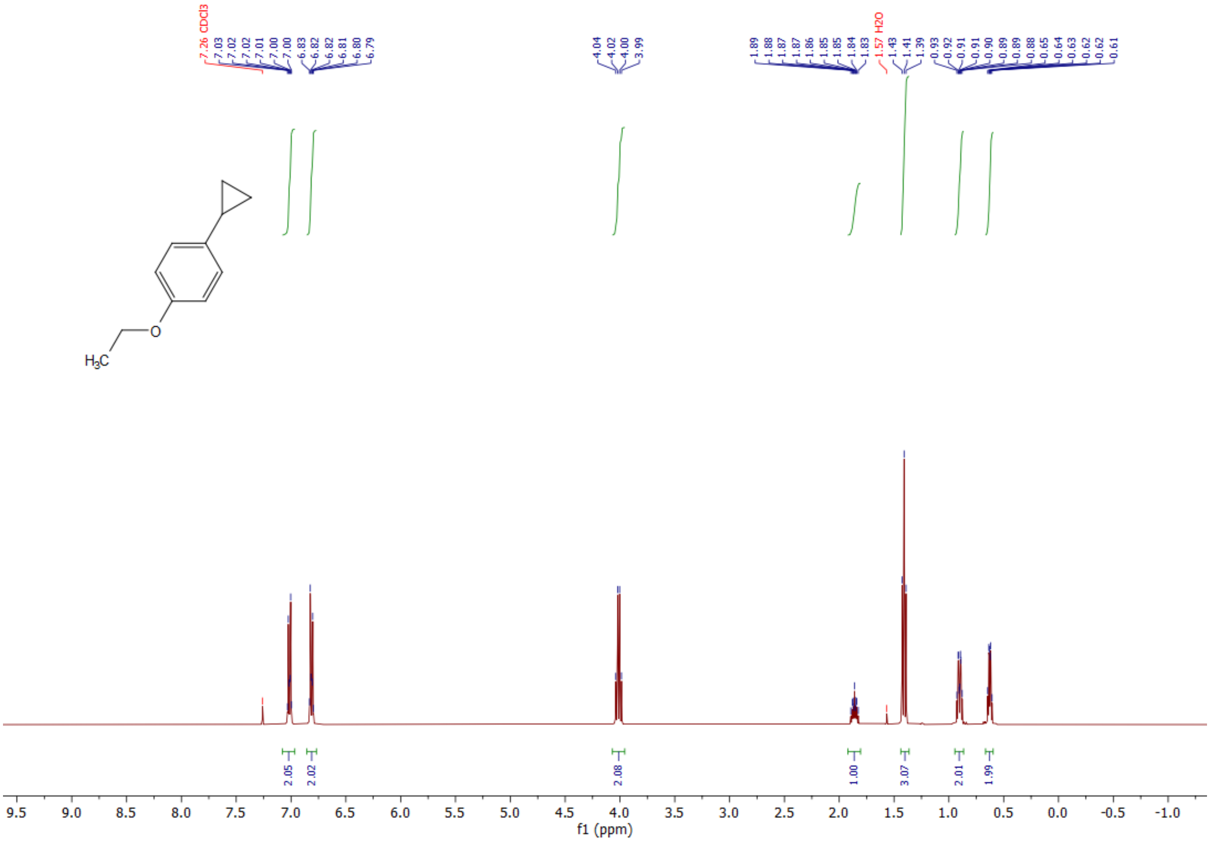

^{13}C NMR (101 MHz, $CDCl_3$) (1p)

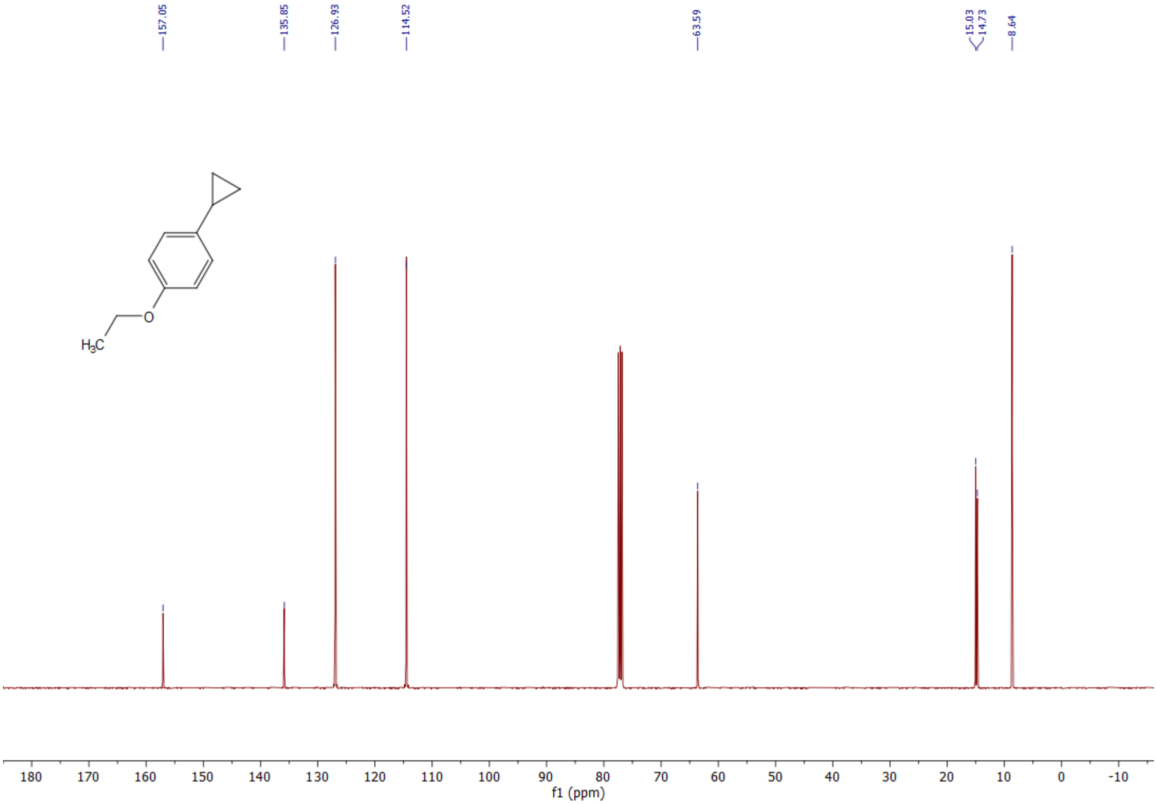

^{1}H-NMR (400 MHz, $CDCl_3$) (1r)

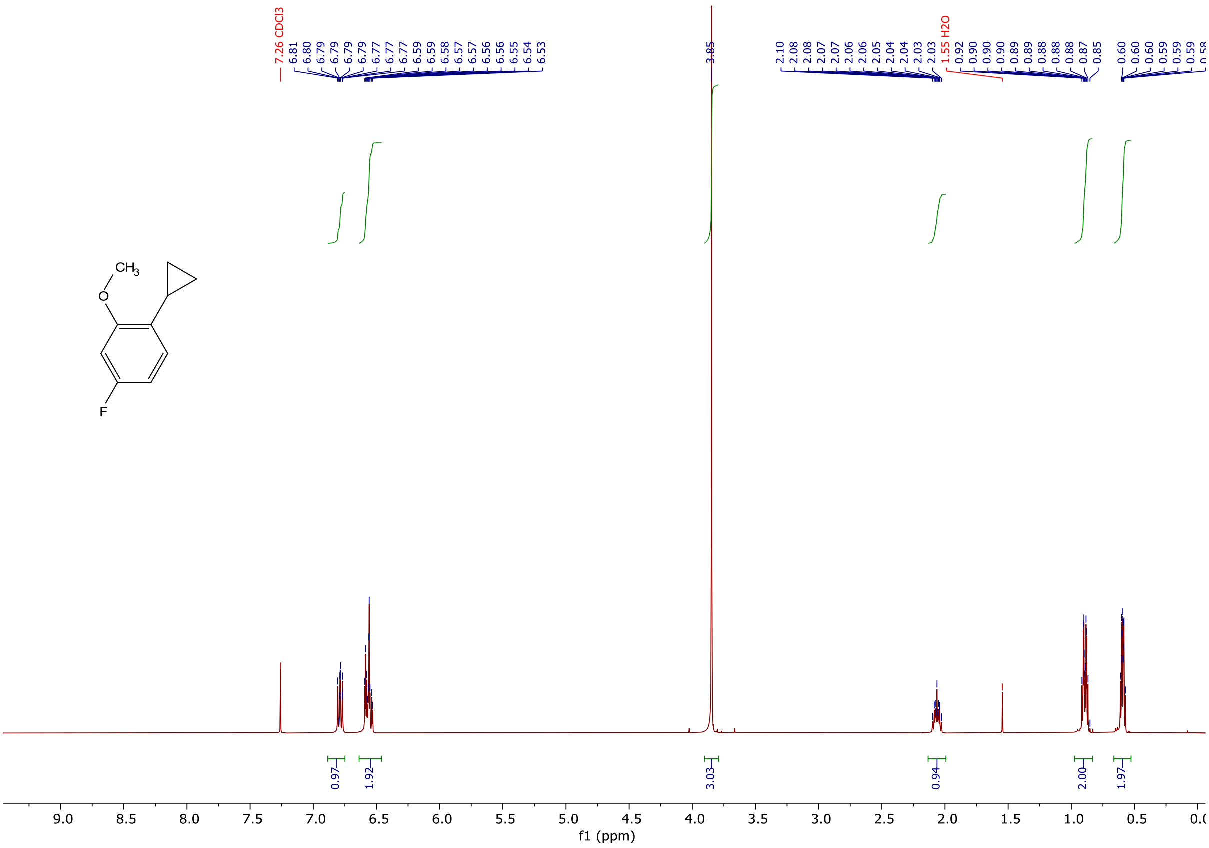

^{13}C NMR (101 MHz, $CDCl_3$) (1r)

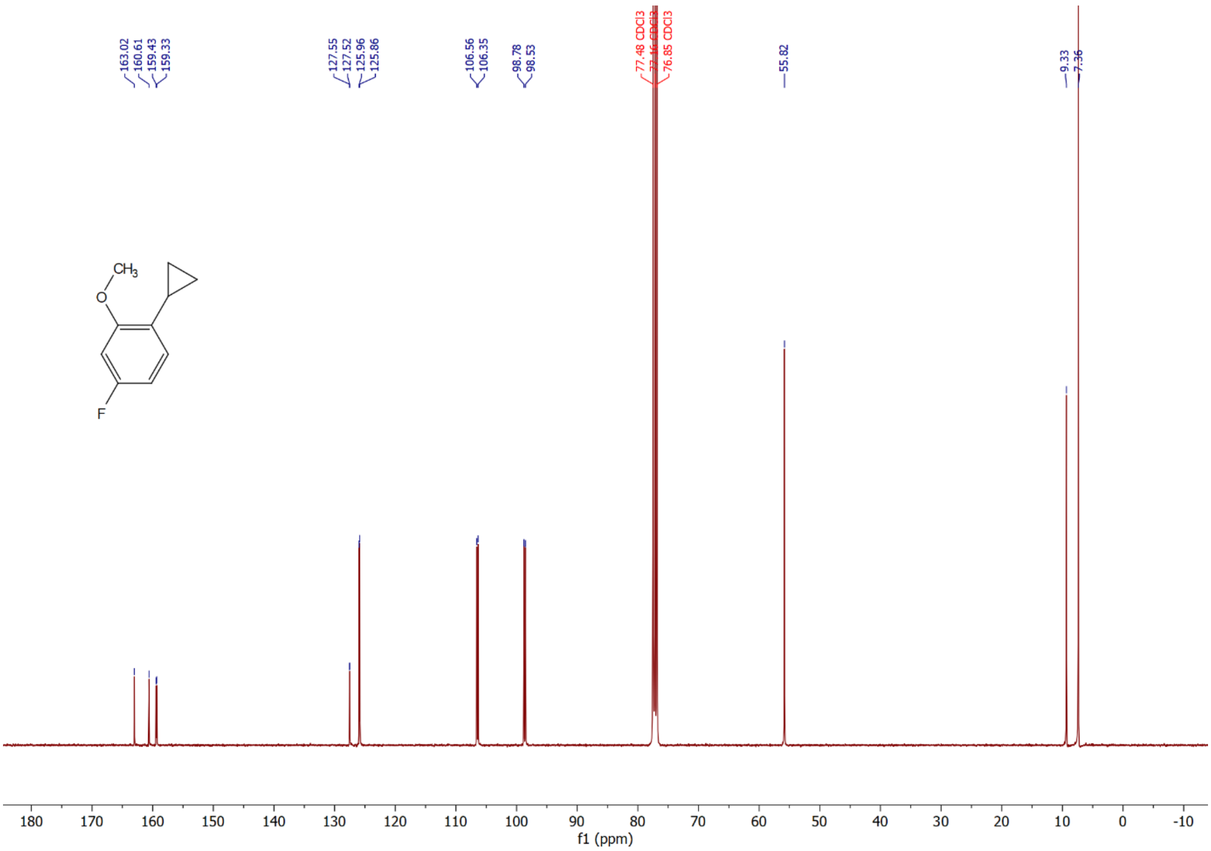

^{19}F NMR (377 MHz, $CDCl_3$) (1r)

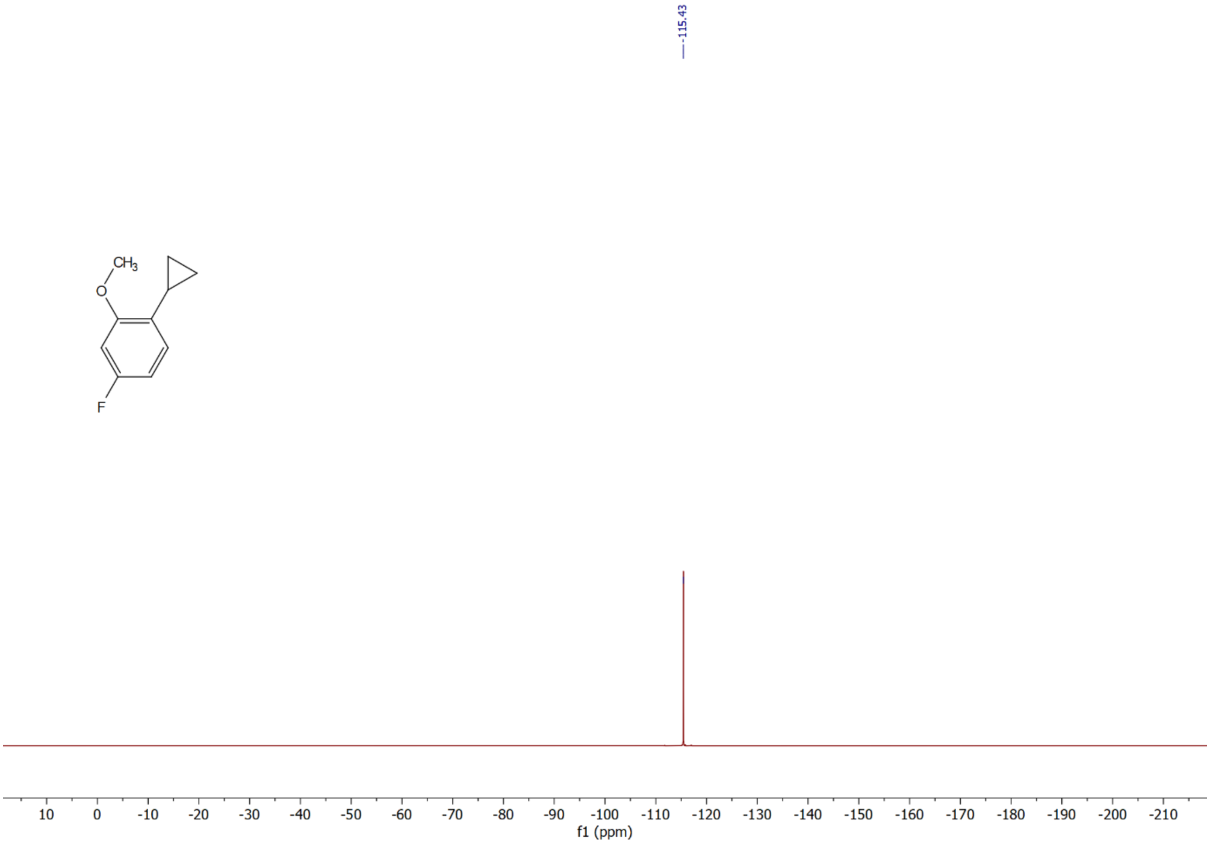

^{1}H-NMR (400 MHz, $CDCl_3$) (2g)

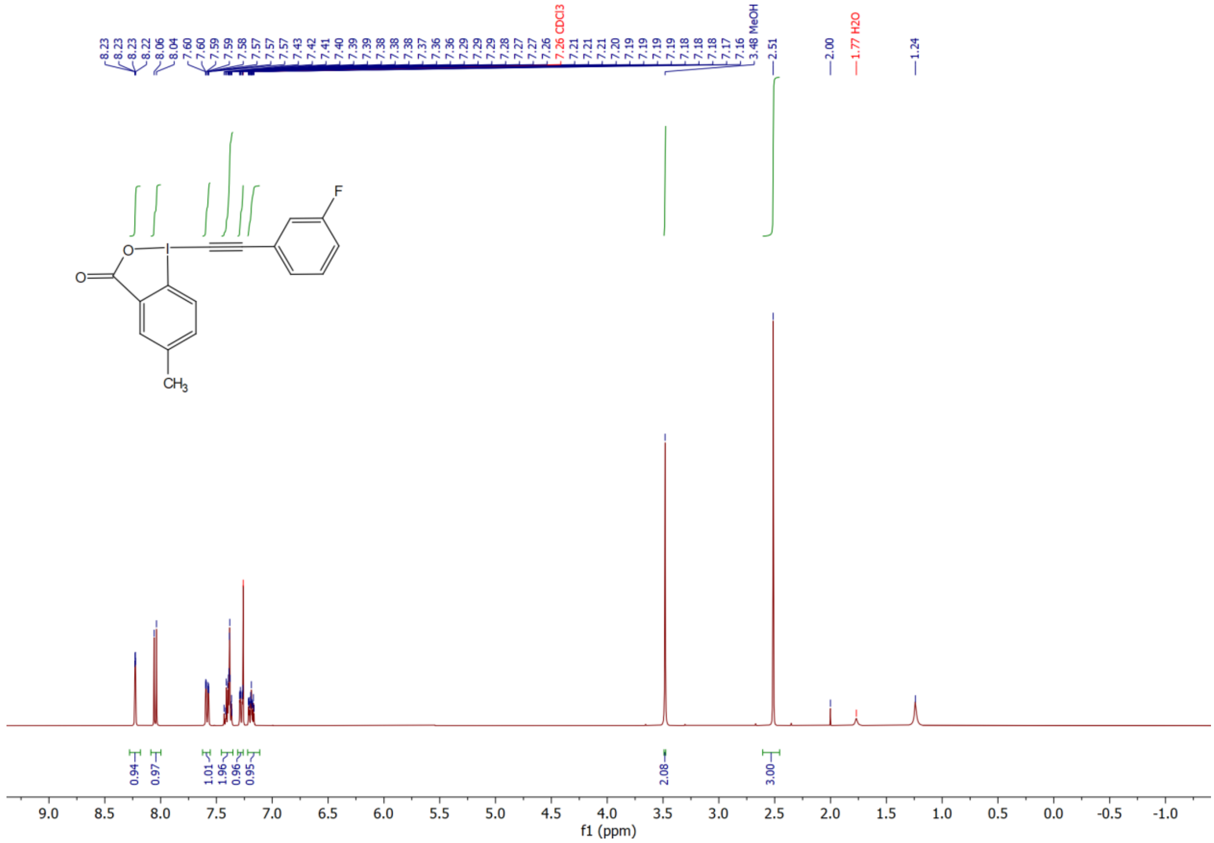

^{13}C NMR (101 MHz, $CDCl_3$) (1r)

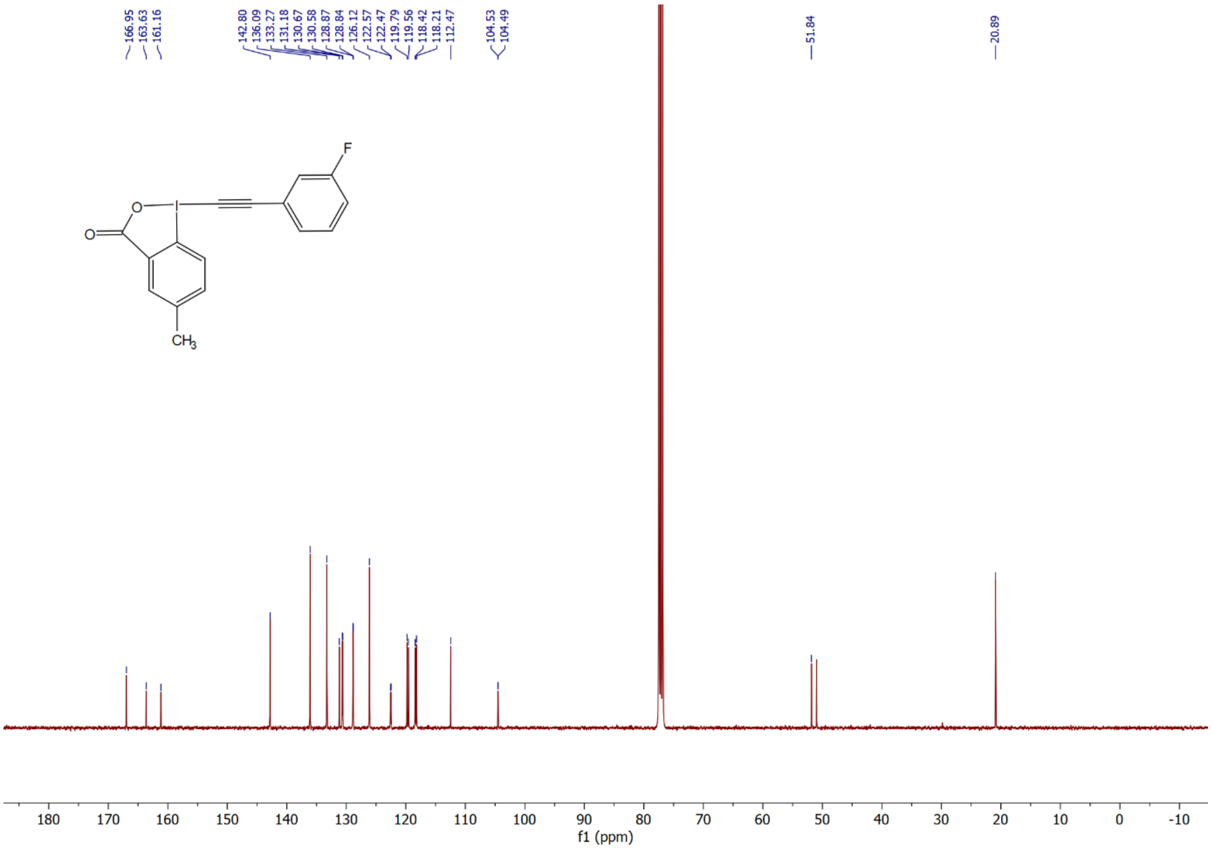

^{19}F NMR (377 MHz, $CDCl_3$) (2g)

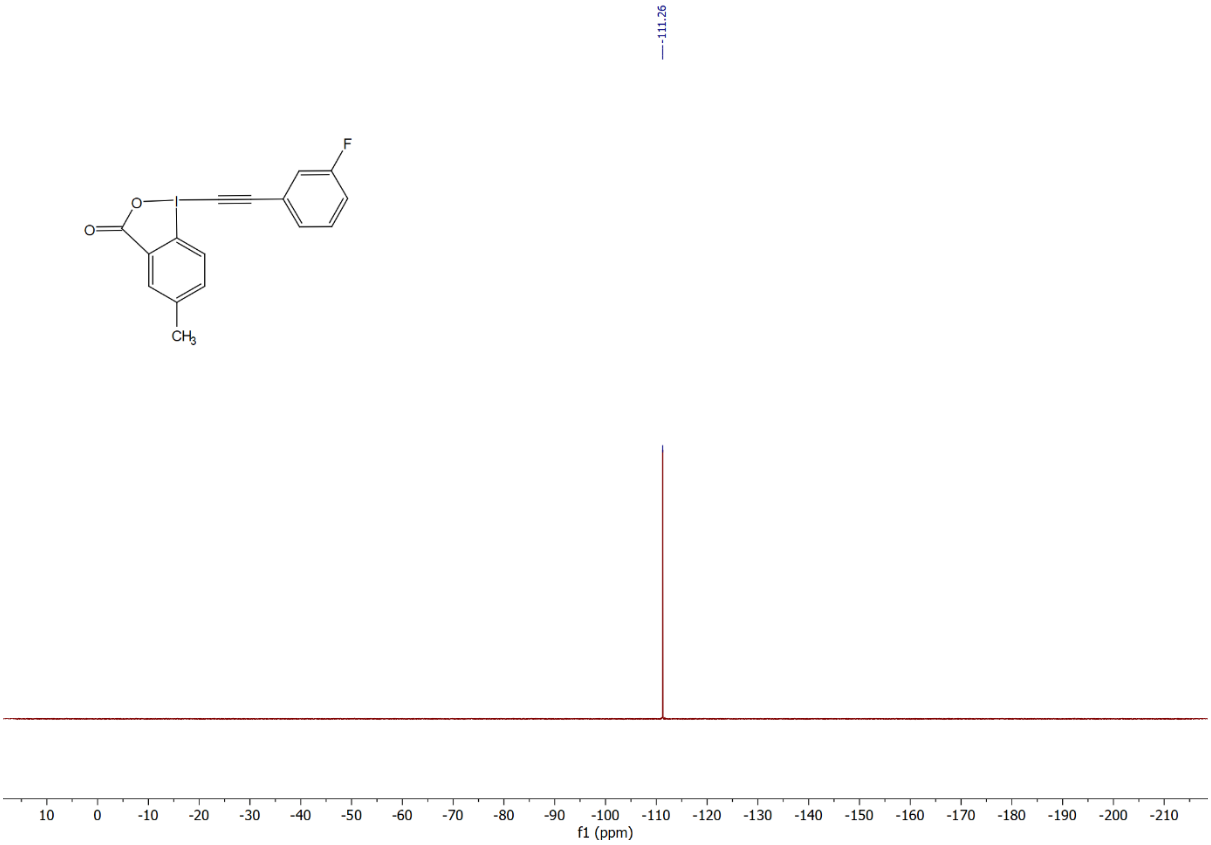

^{1}H NMR (400 MHz, $CDCl_3$) (3a)

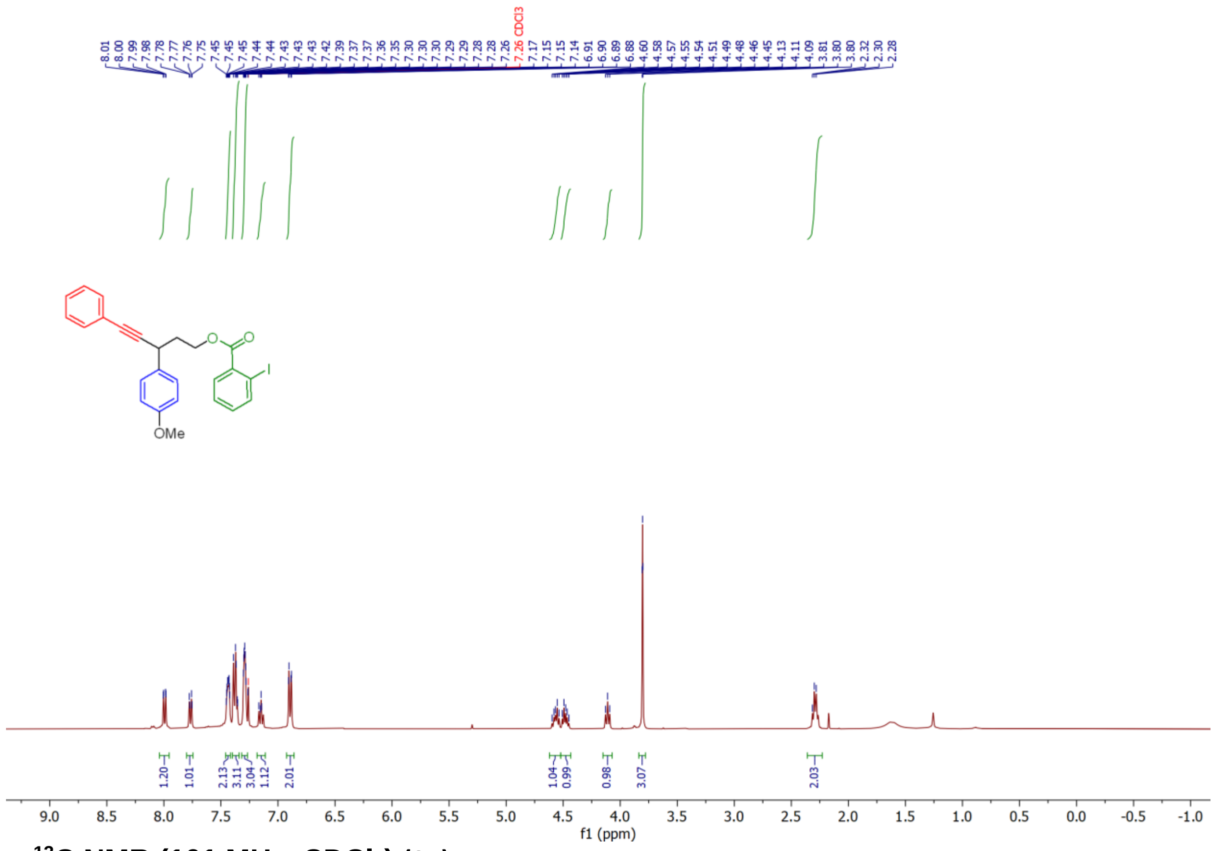

^{13}C NMR (101 MHz, $CDCl_3$) (3a)

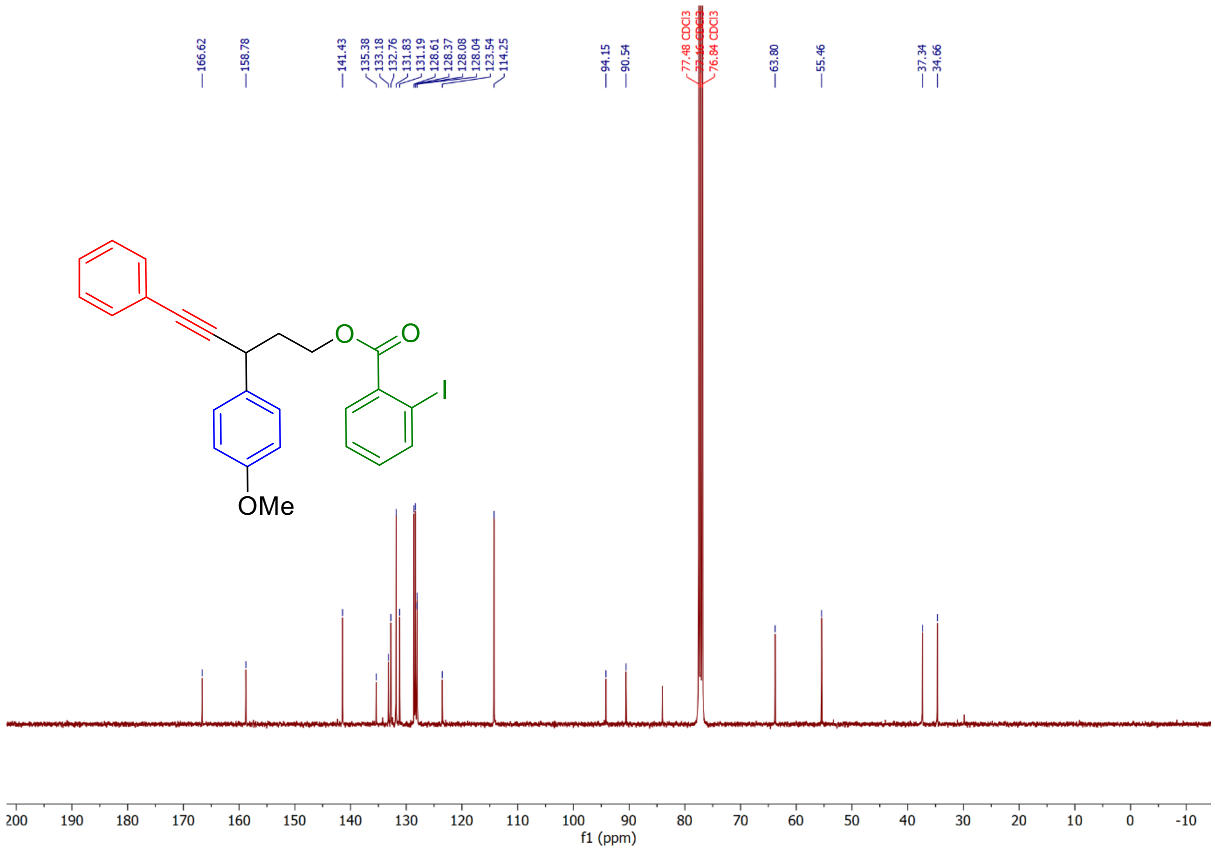

^{1}H NMR (400 MHz, $CDCl_3$) **(3b)**

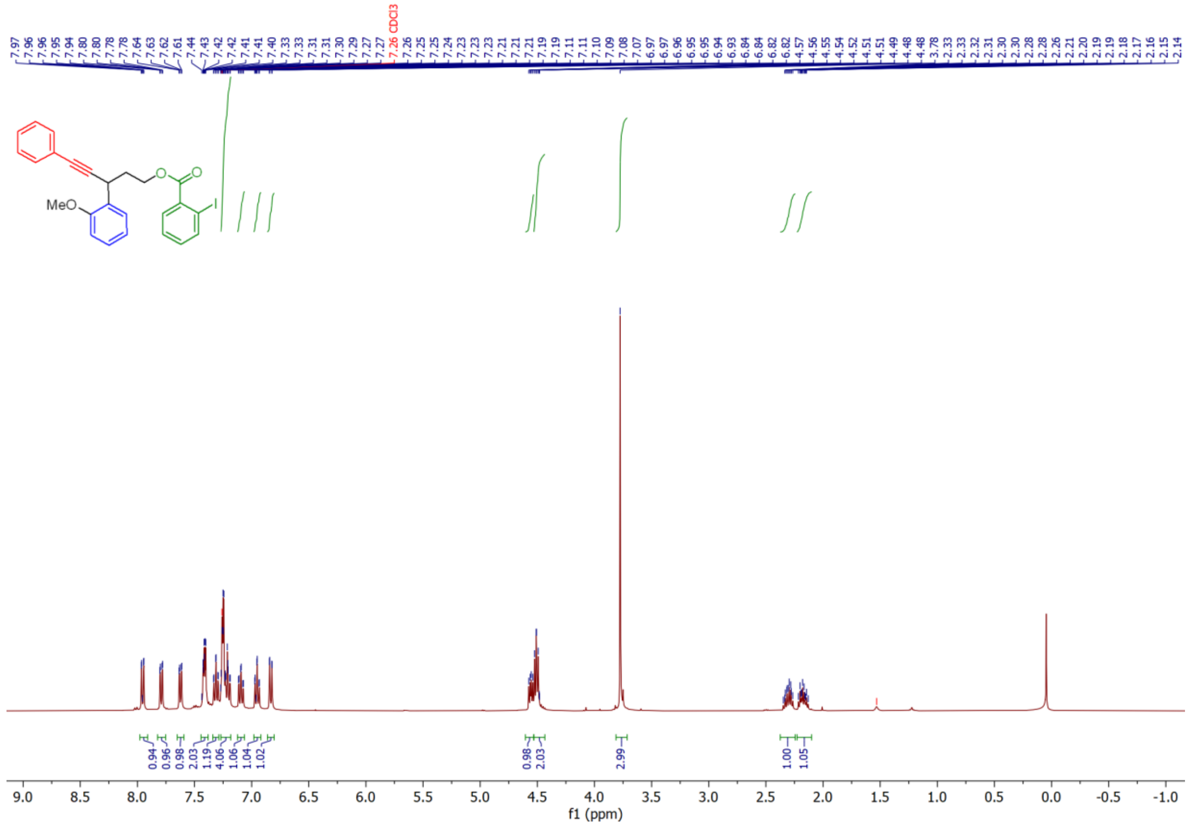

^{13}C NMR (101 MHz, $CDCl_3$) (3b)

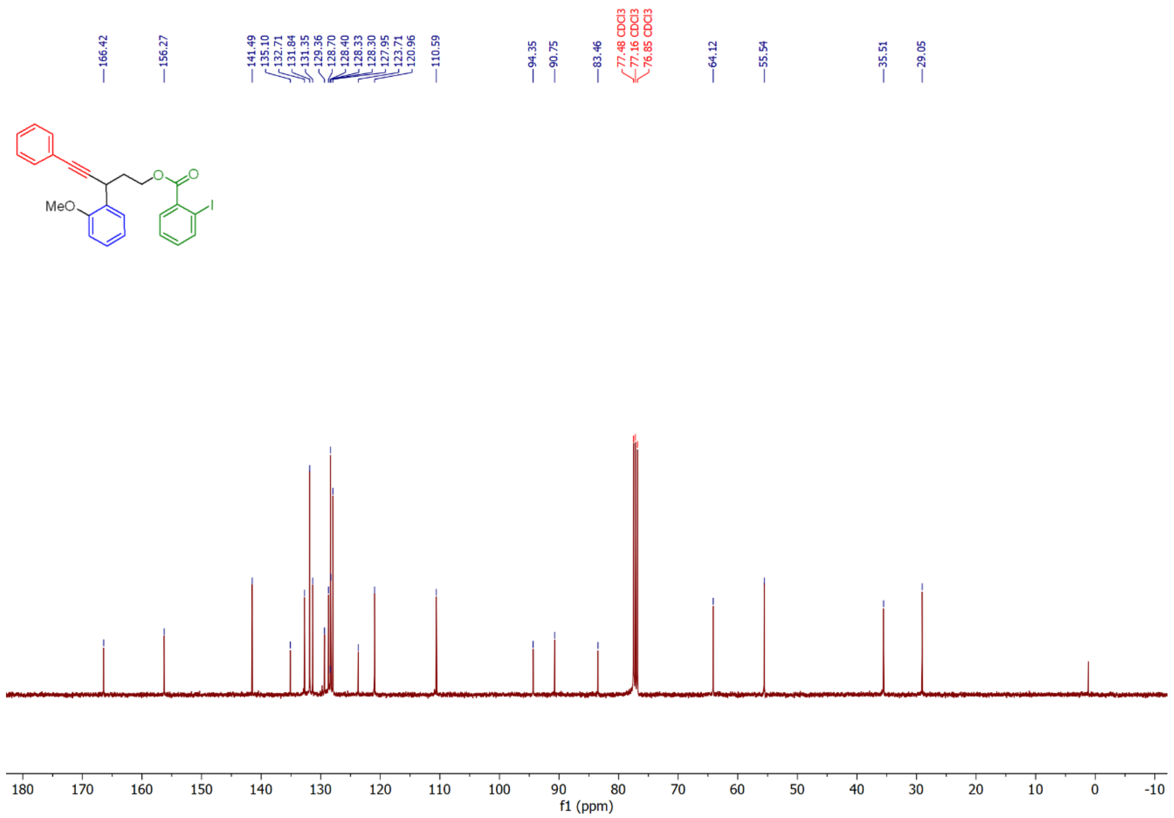

^{1}H NMR (400 MHz, $CDCl_3$) **(3c)**

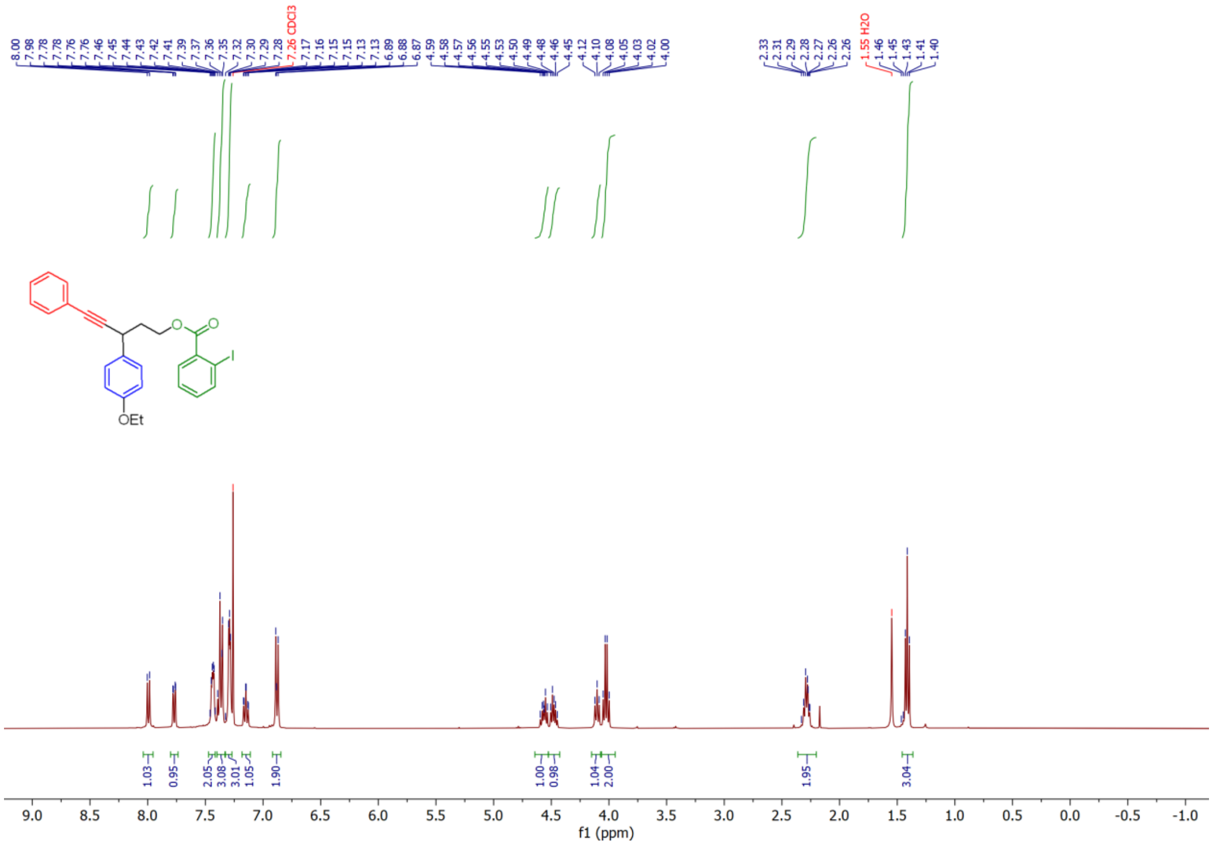

^{13}C NMR (101 MHz, $CDCl_3$) **(3c)**

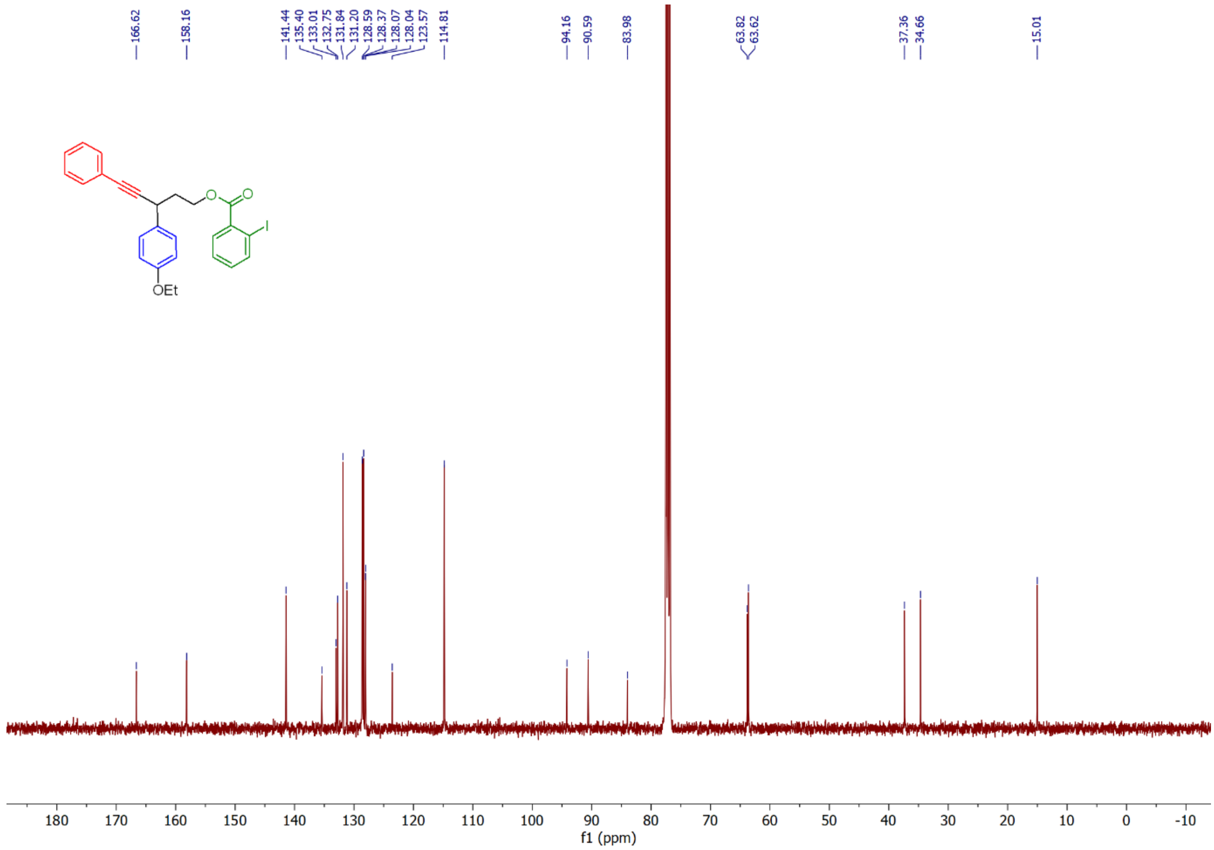

^{1}H NMR (400 MHz, $CDCl_3$) **(3d)**

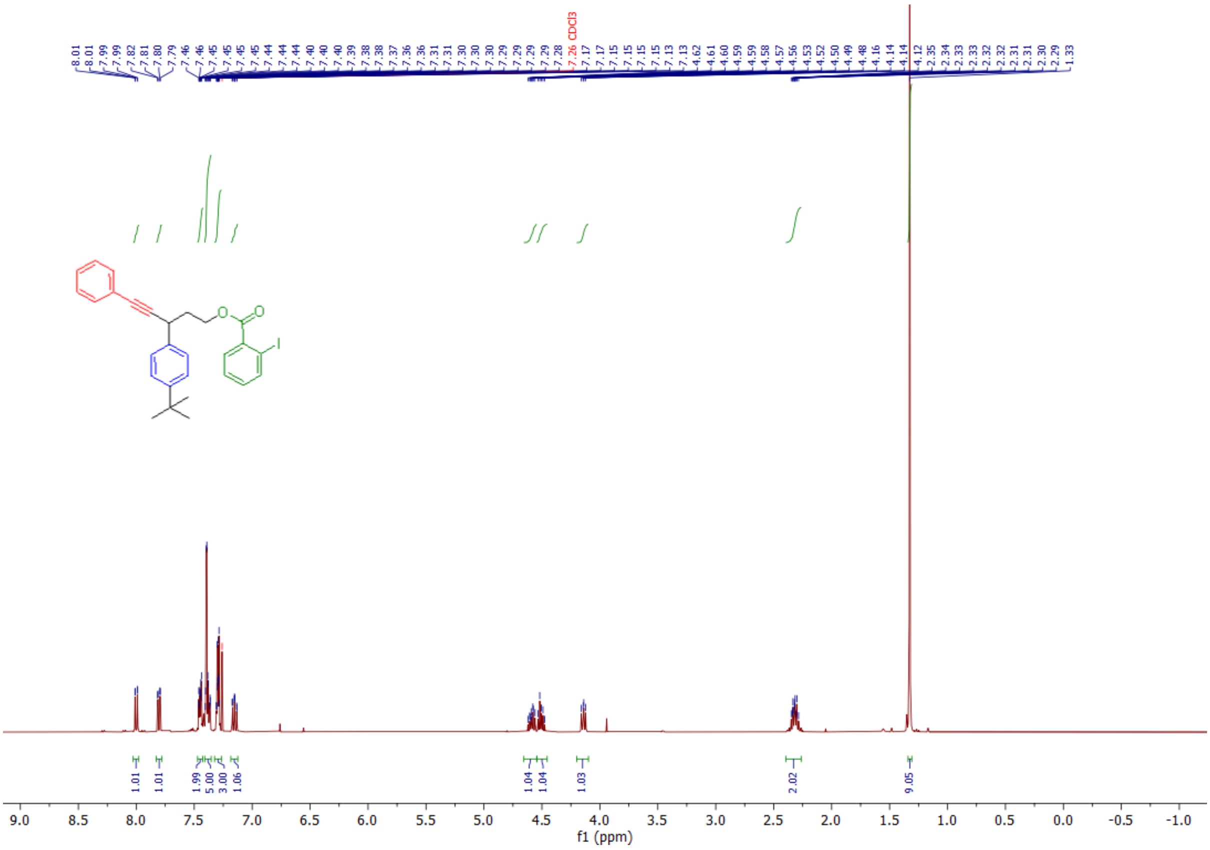

^{13}C NMR (101 MHz, $CDCl_3$) **(3d)**

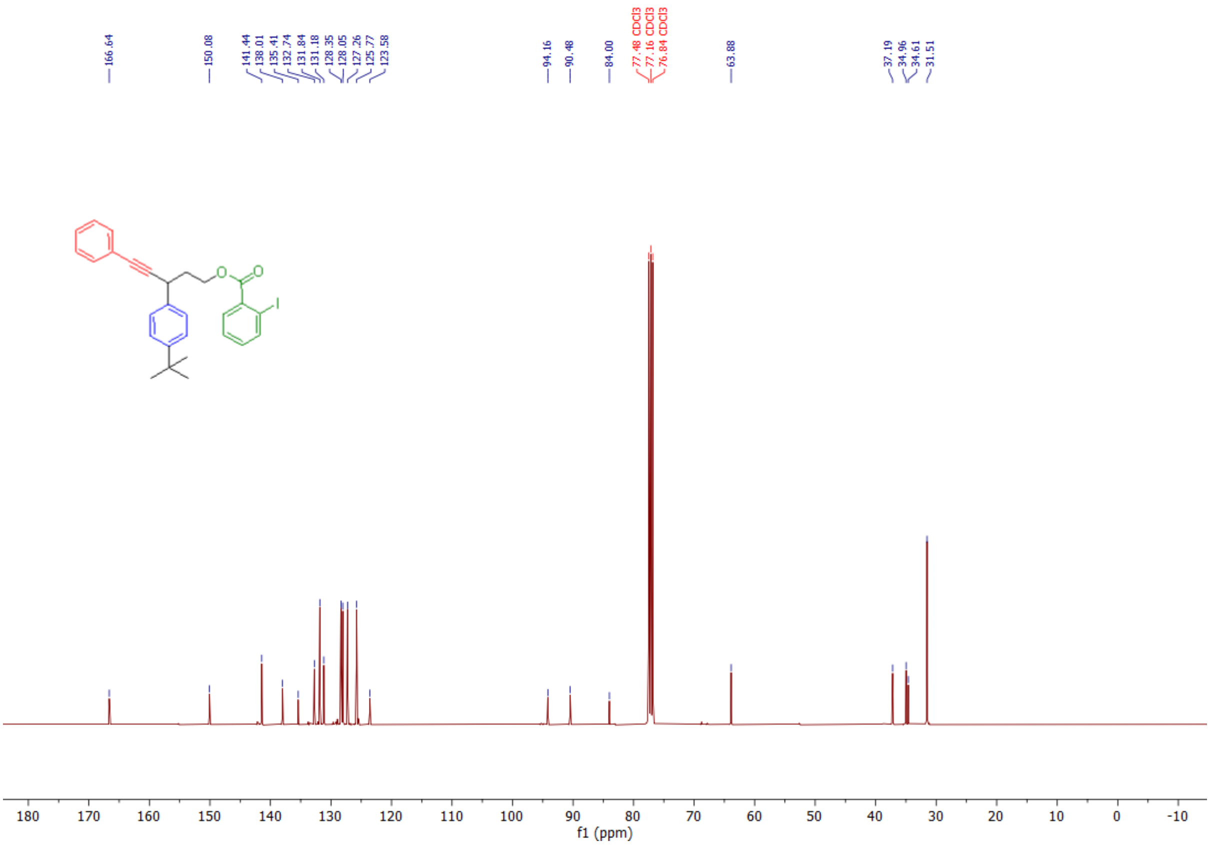

^{1}H NMR (400 MHz, $CDCl_3$) **(3e)**

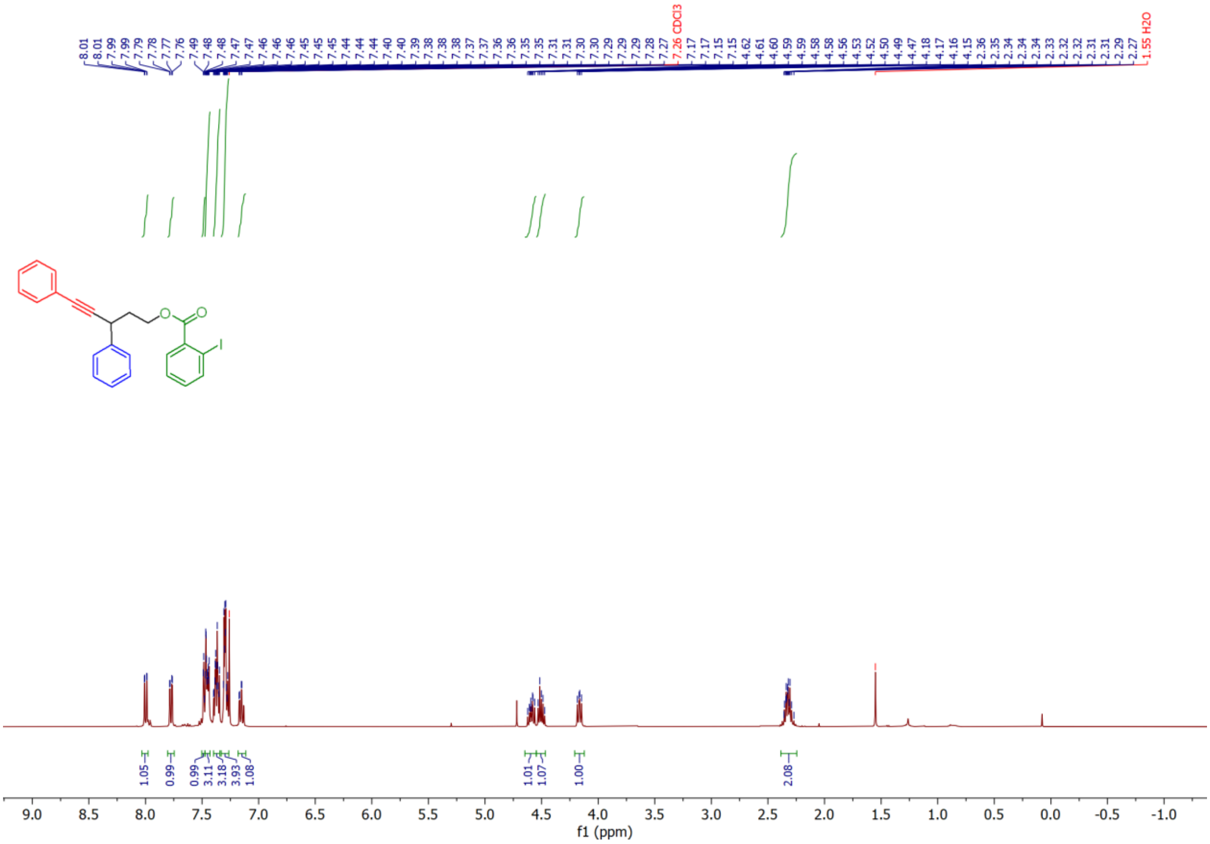

^{13}C NMR (101 MHz, $CDCl_3$): **(3e)**

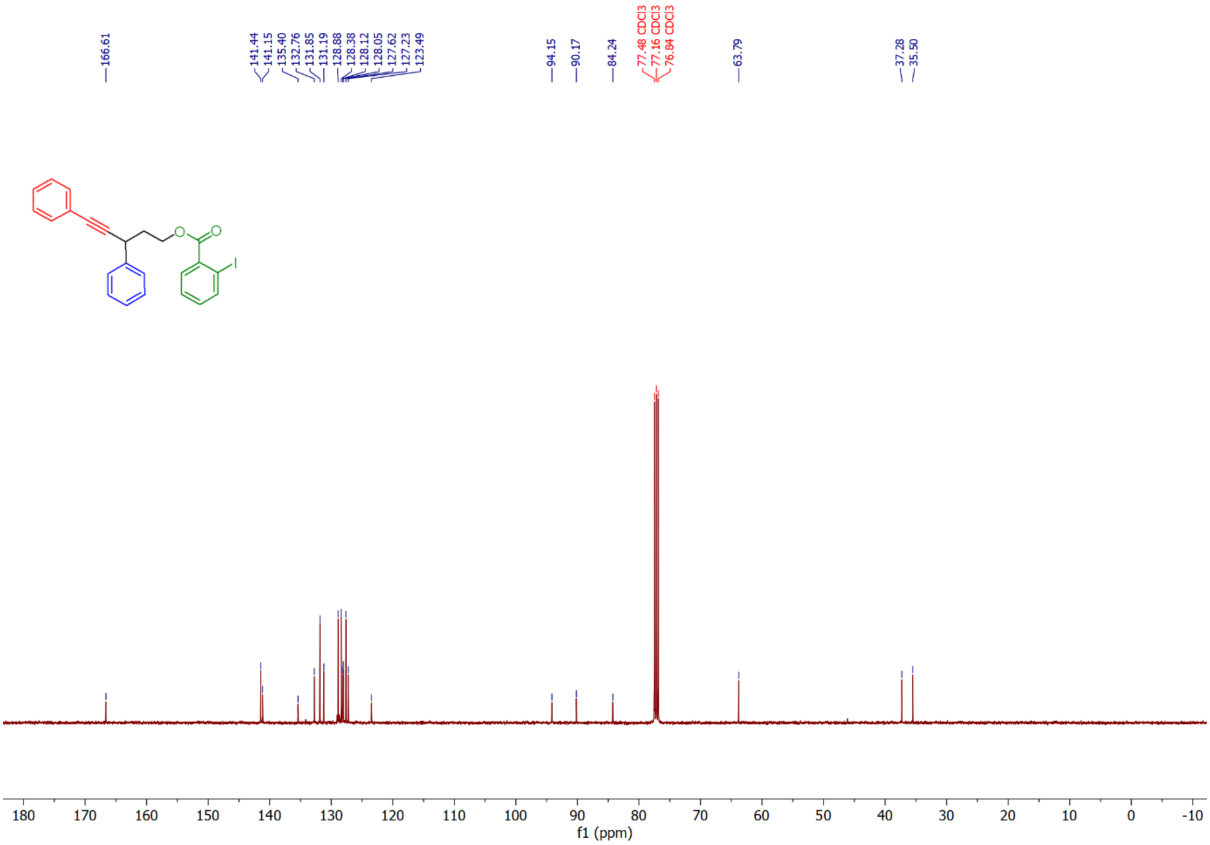

^{1}H NMR (400 MHz, $CDCl_3$) **(3f)**

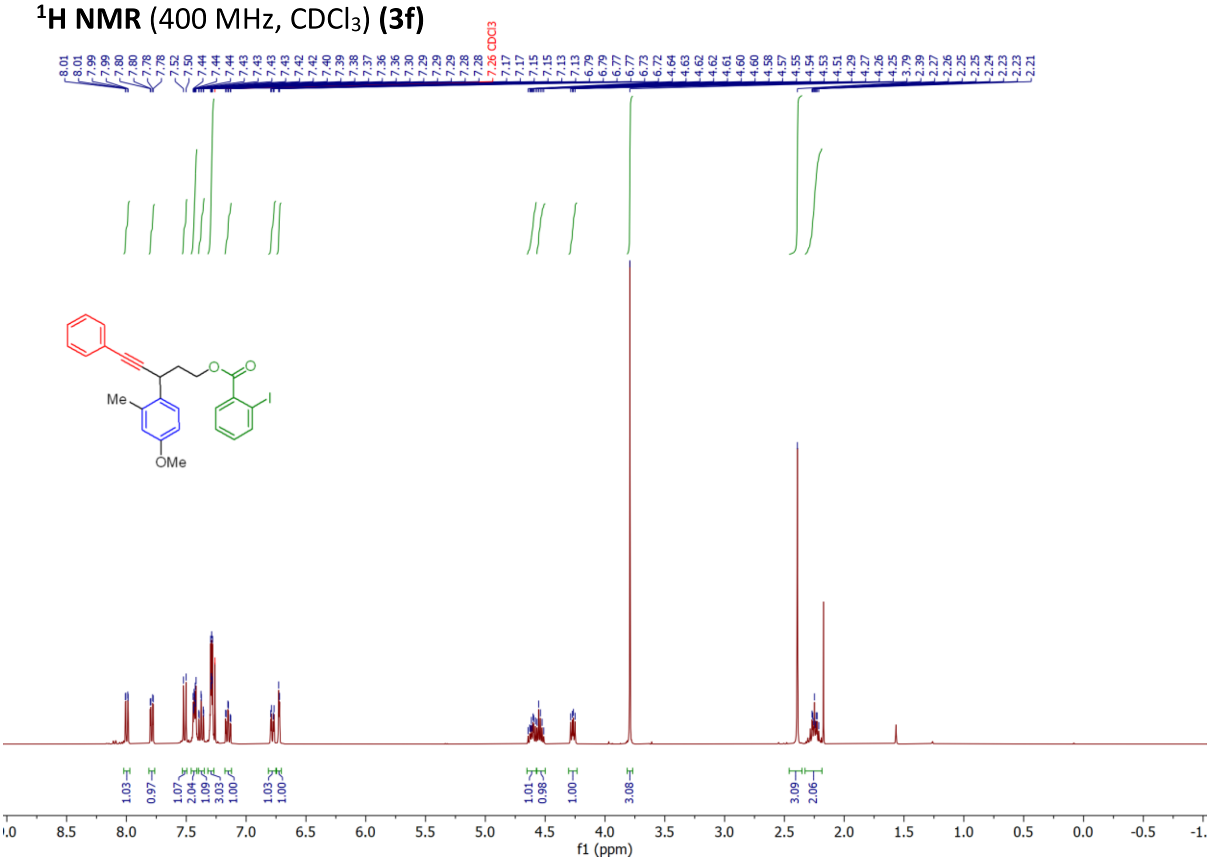

^{13}C NMR (101 MHz, $CDCl_3$) **(3f)**

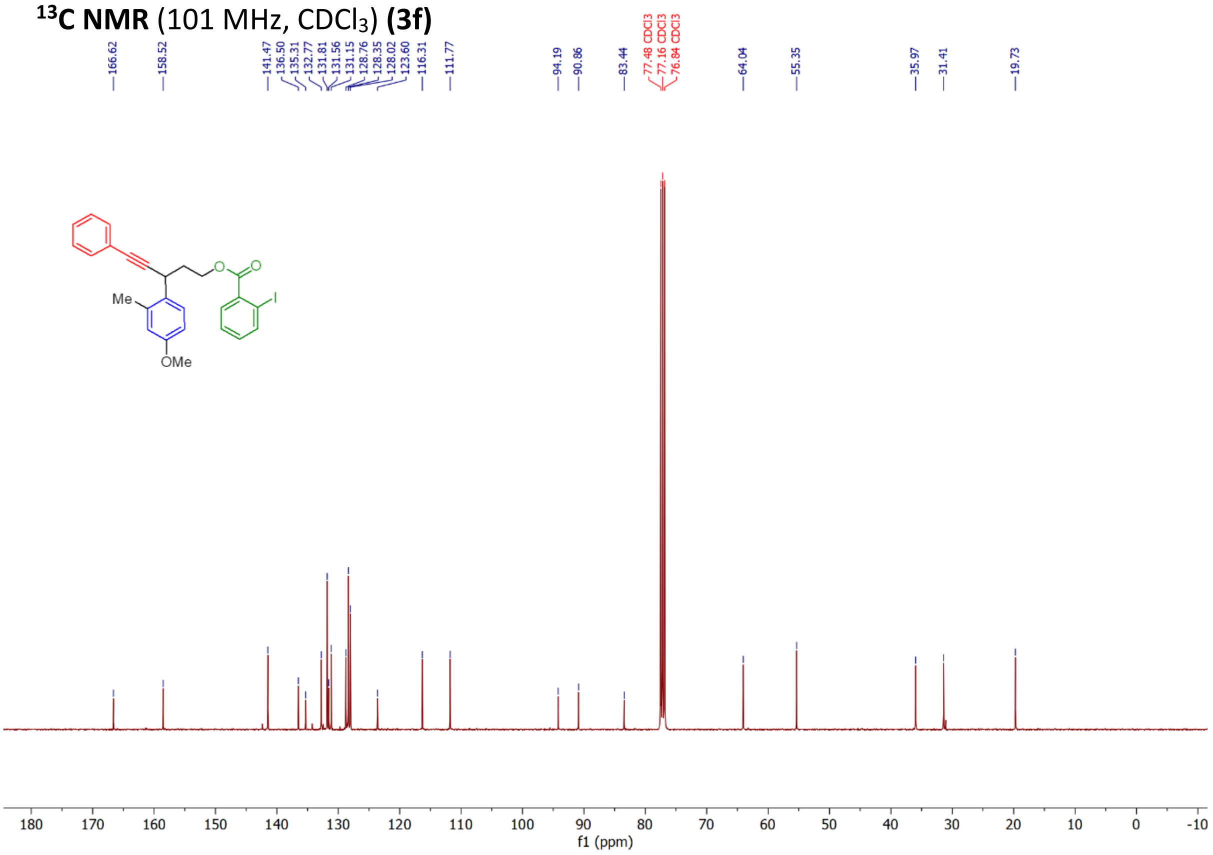

^{1}H NMR (400 MHz, $CDCl_3$) **(3h)**

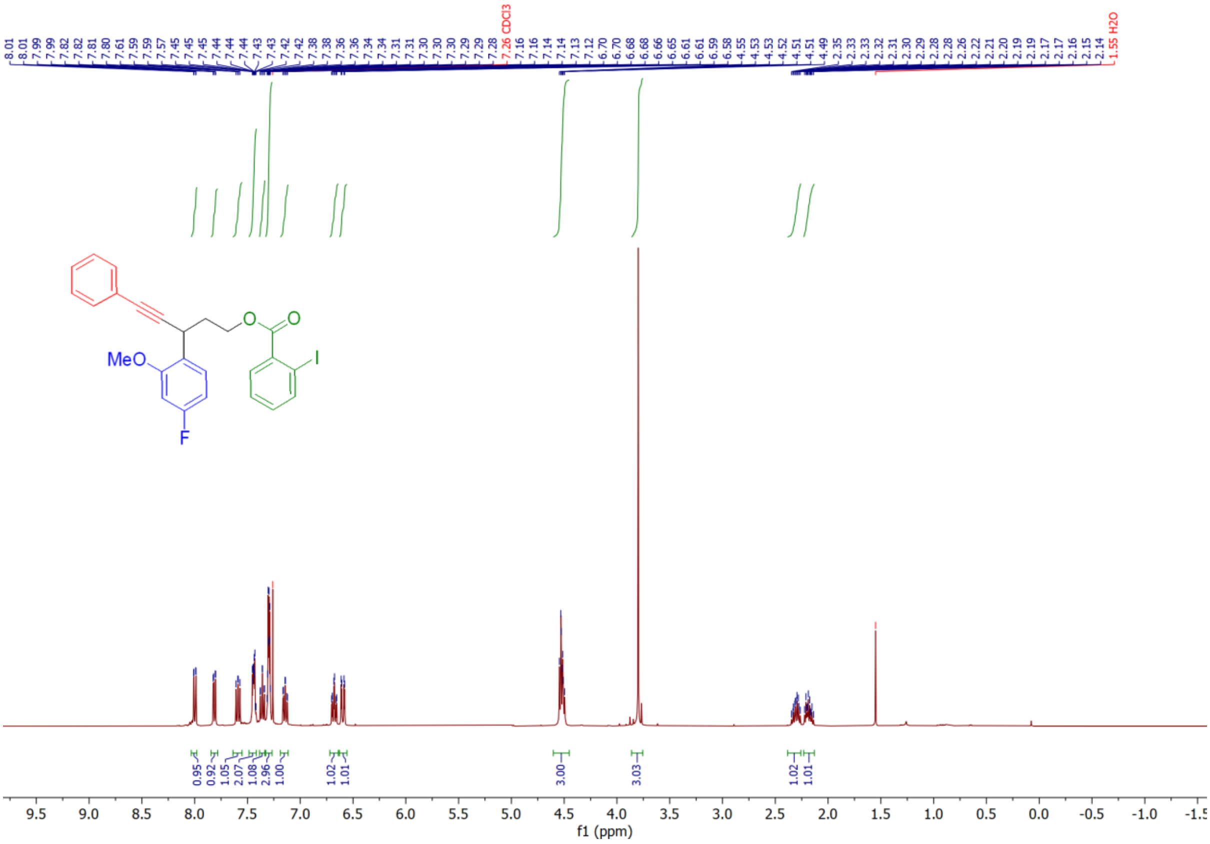

^{13}C NMR (151 MHz, $CDCl_3$) **(3h)**

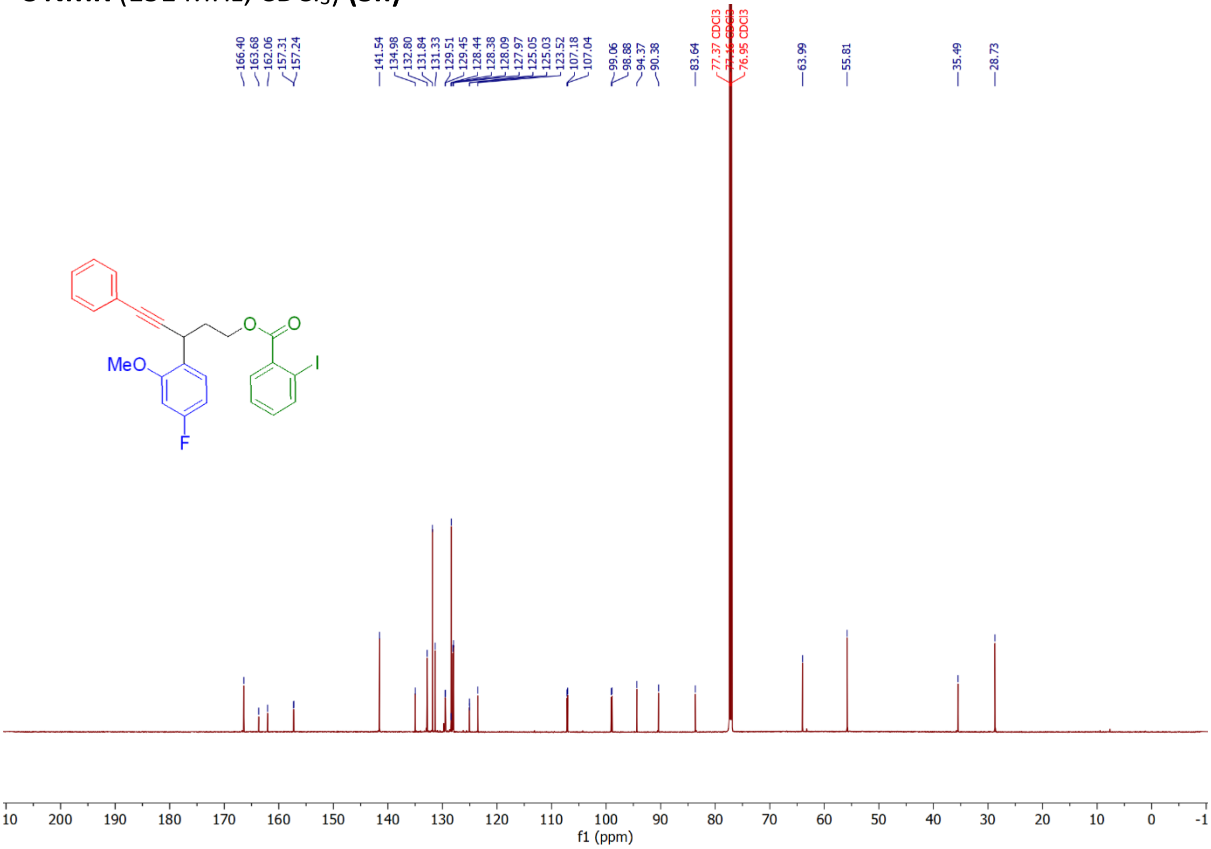

^19^F NMR (376 MHz, $CDCl_3$) **(3h)**

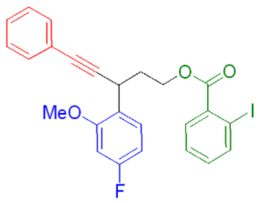

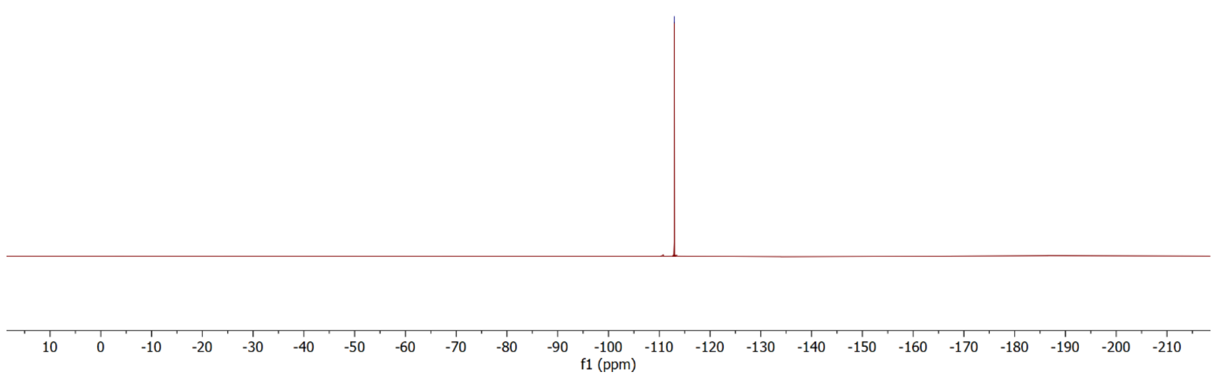

^{1}H NMR (400 MHz, CDCl$_3$) (3i) Blue : major diastereomer. Organe : minor diastereomer

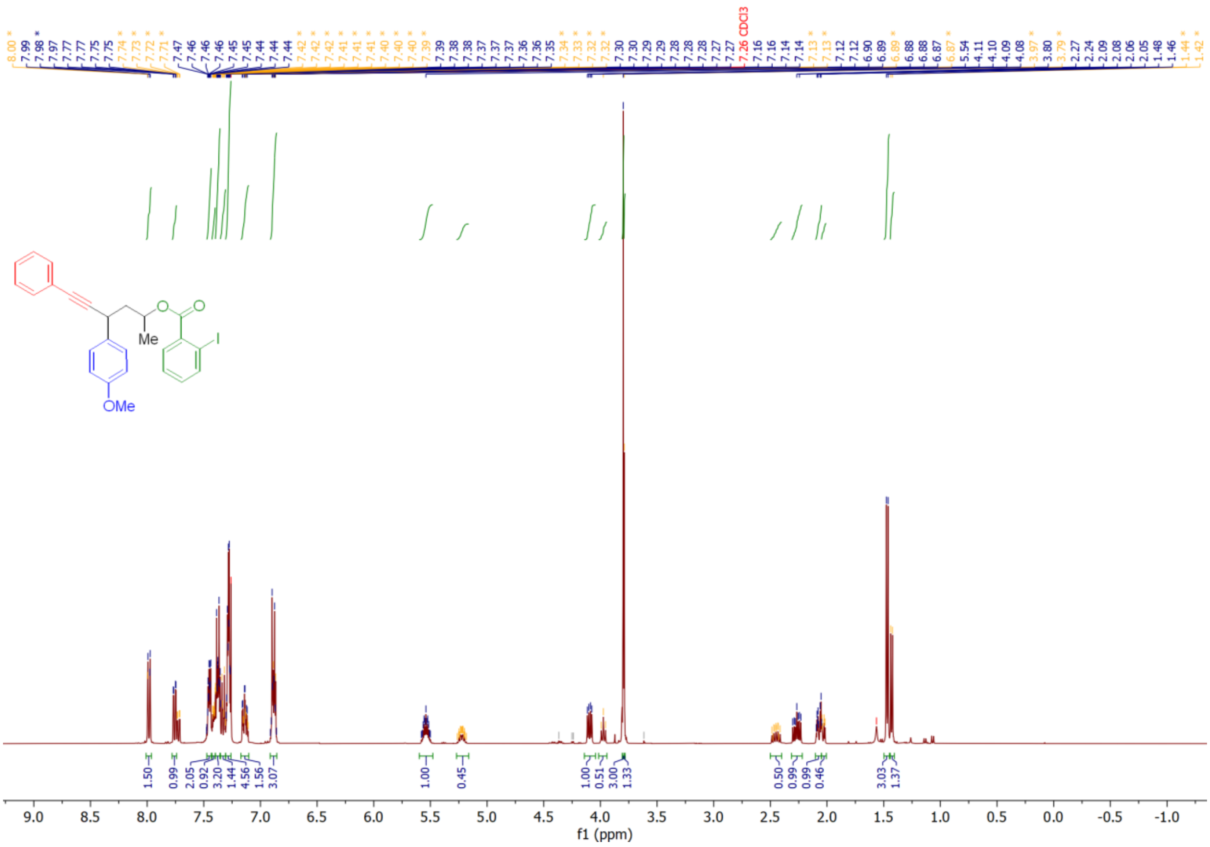

^{13}C NMR (101 MHz, CDCl$_3$) **(3i)**

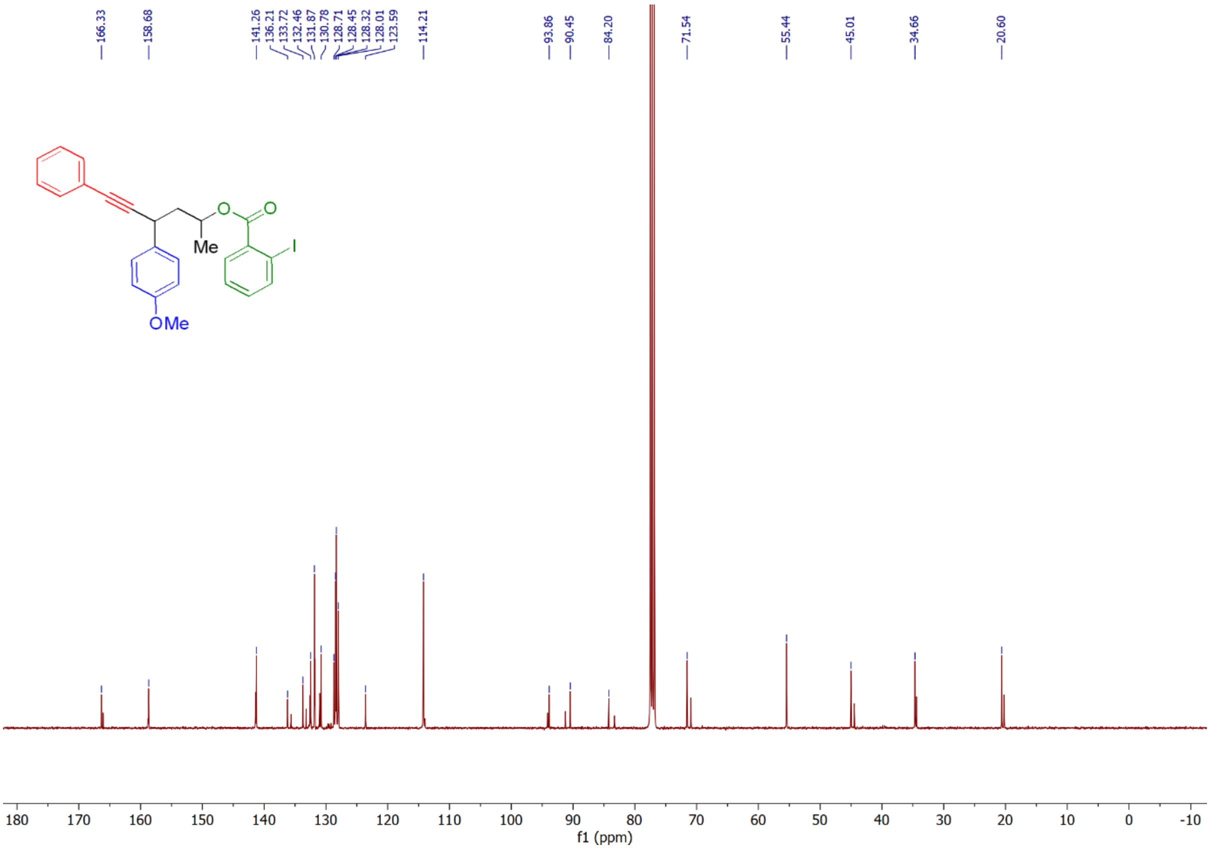

^{1}H NMR (400 MHz, CDCl$_3$) (3j)

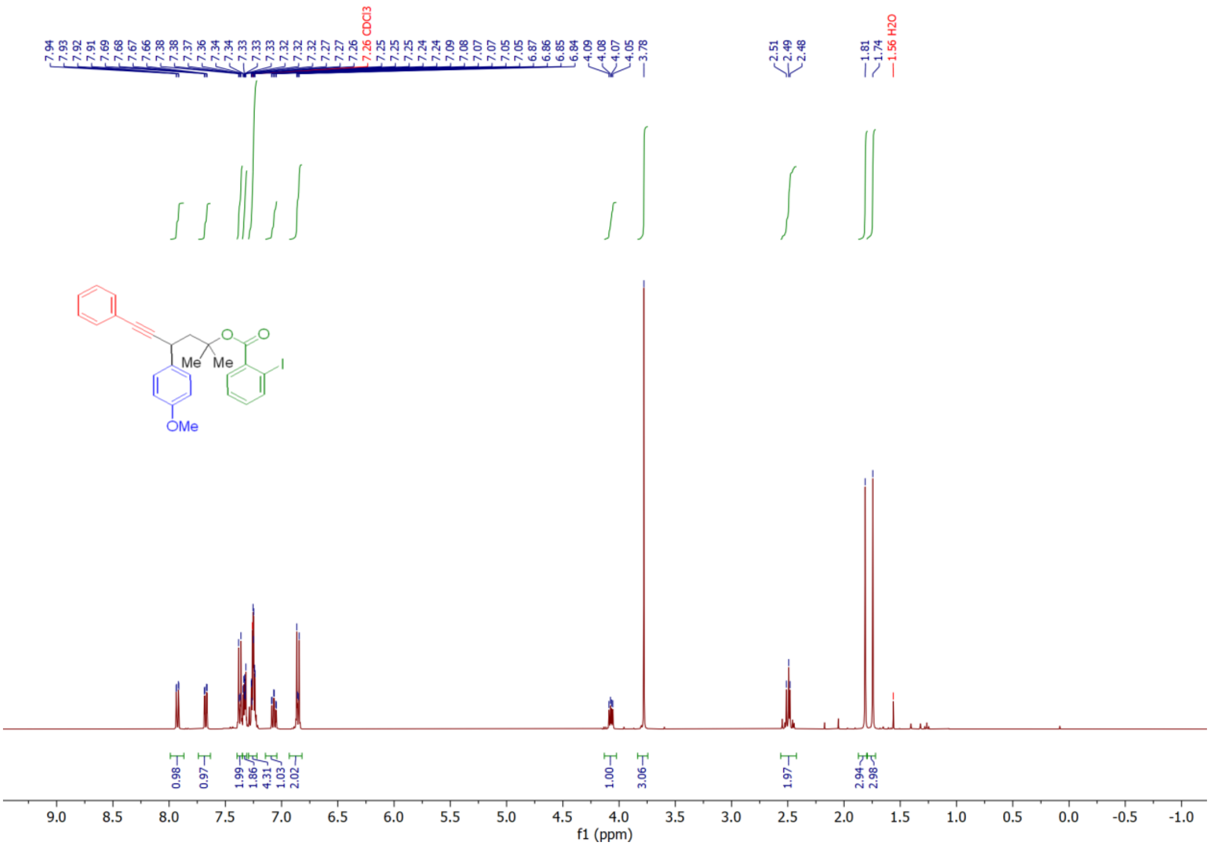

^{13}C NMR ^{13}C NMR (101 MHz, CDCl$_3$) **(3j)**

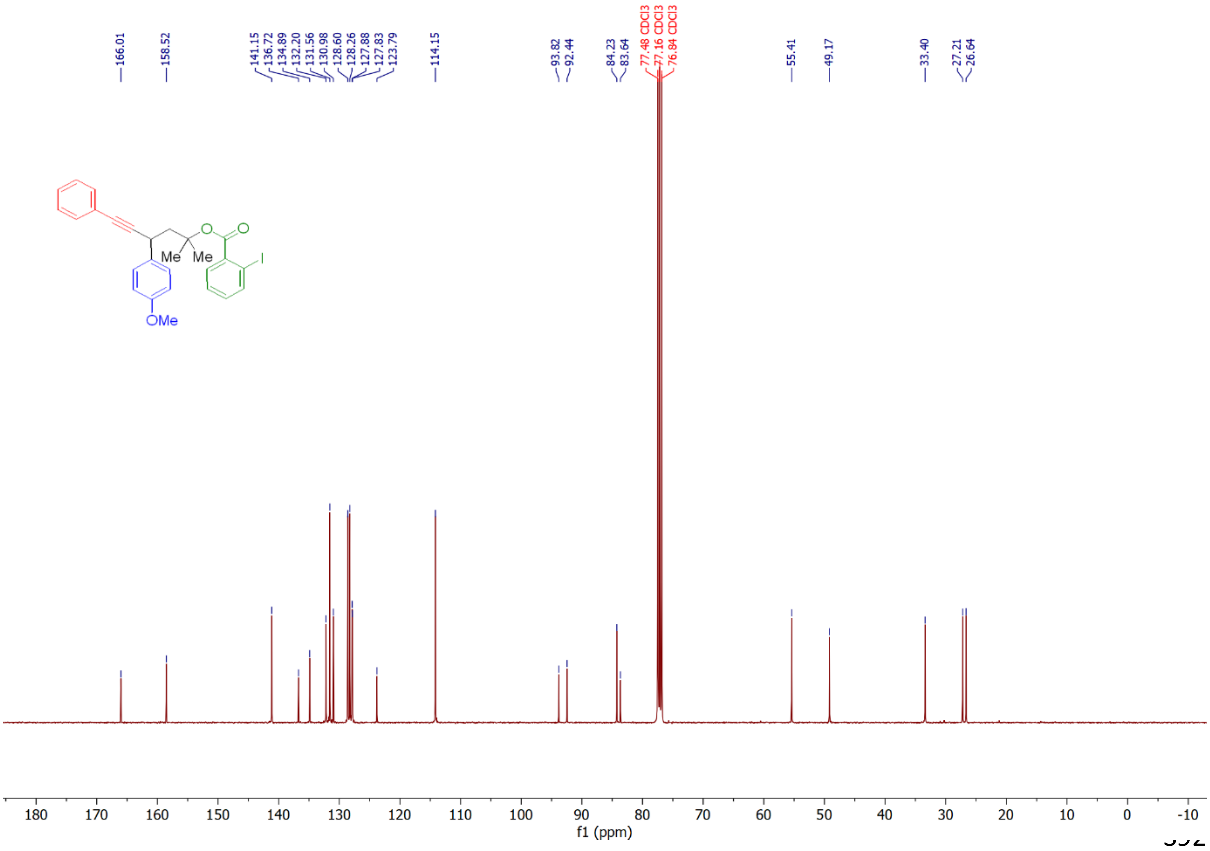

^{1}H NMR (400 MHz, $CDCl_3$) **(3k)**

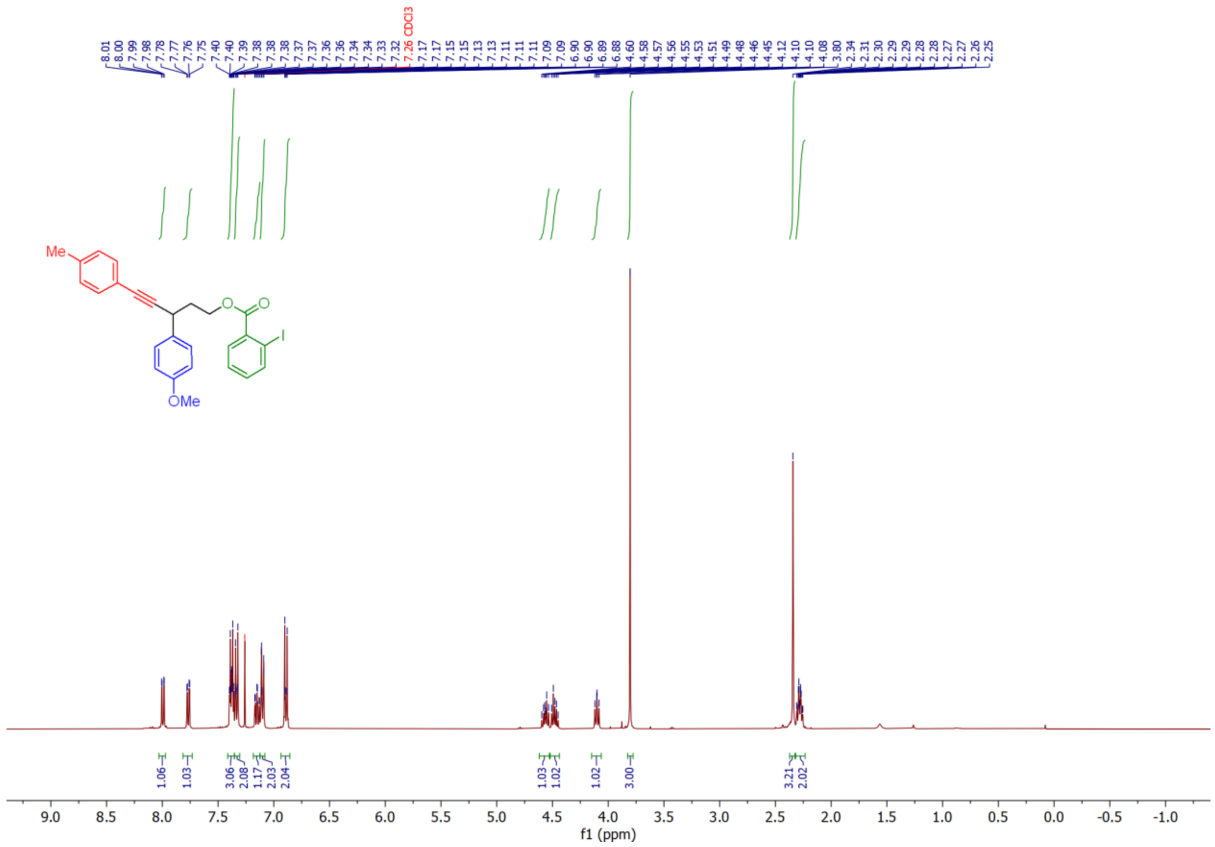

^{13}C NMR (101 MHz, $CDCl_3$) **(3k)**

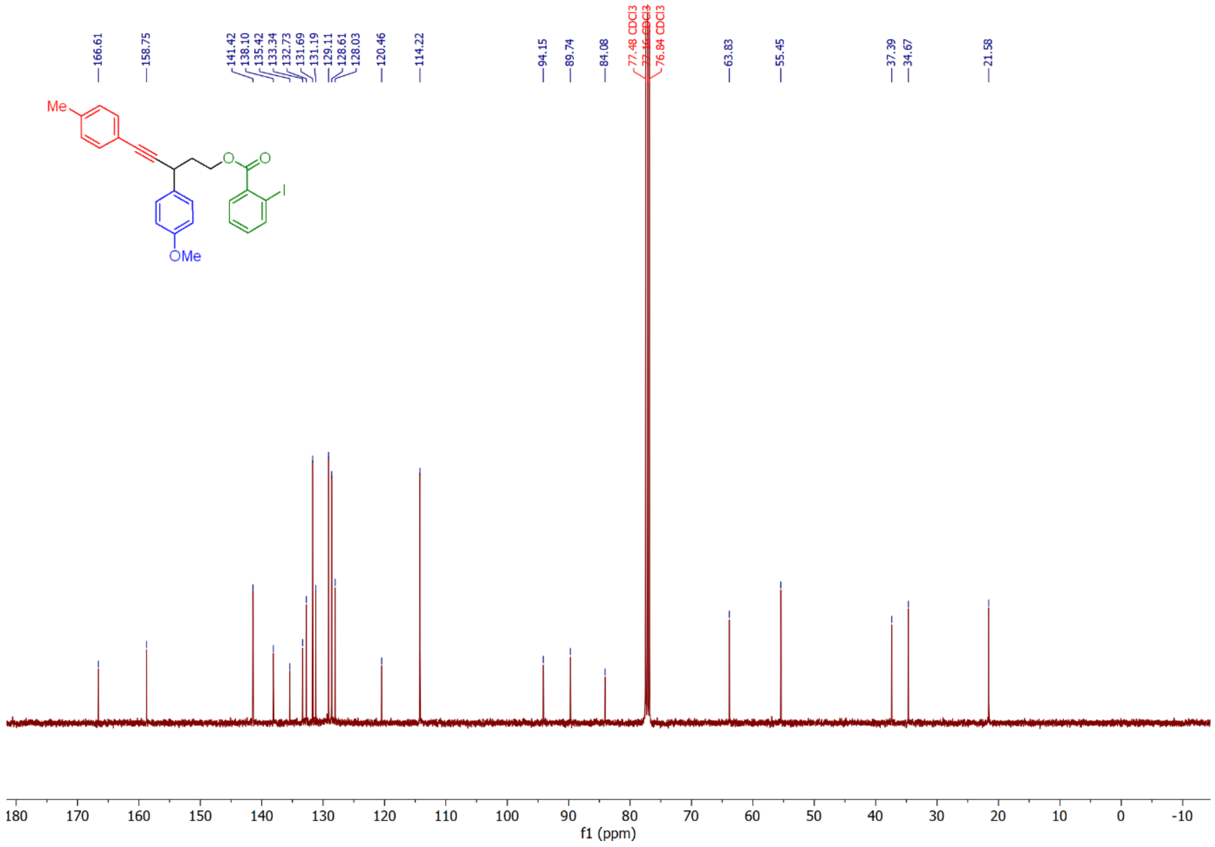

^{1}H NMR (400 MHz, $CDCl_3$) (3l)

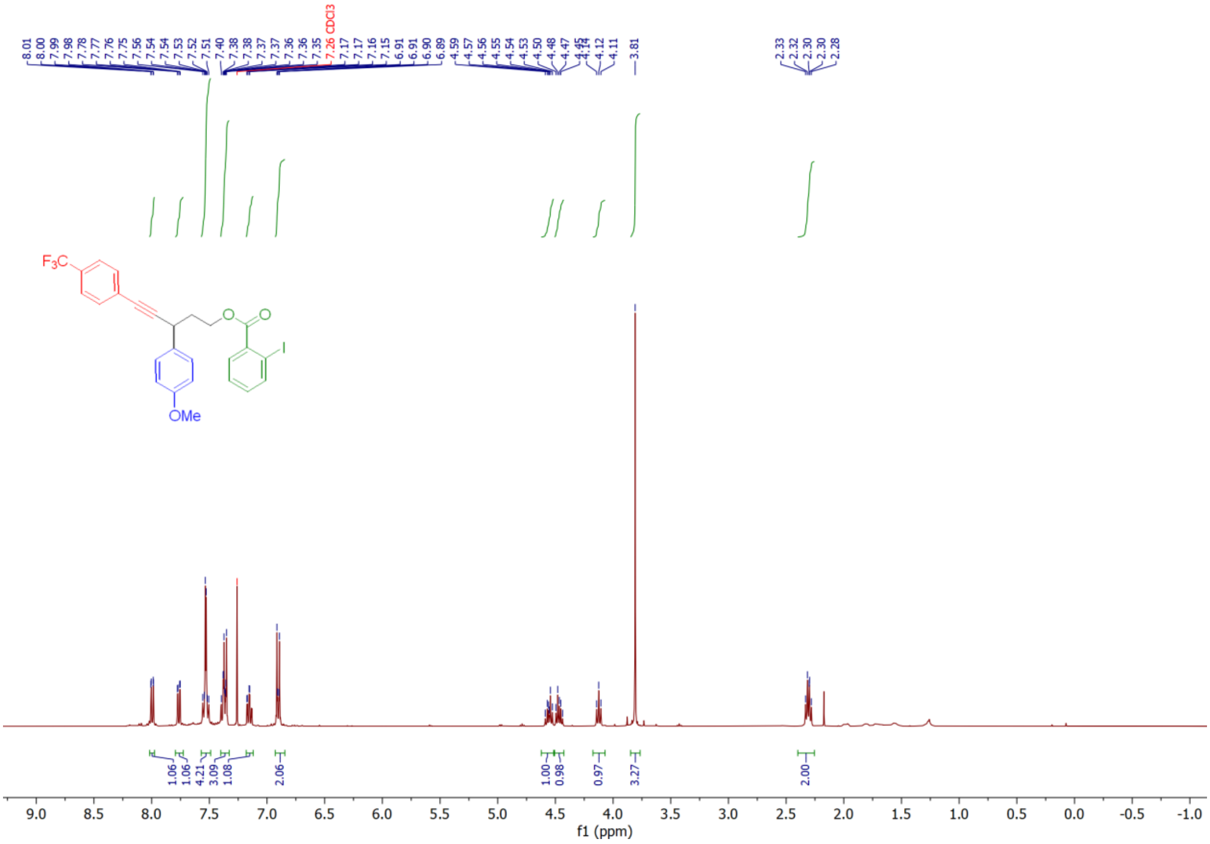

^{13}C NMR (101 MHz, $CDCl_3$) (3l)

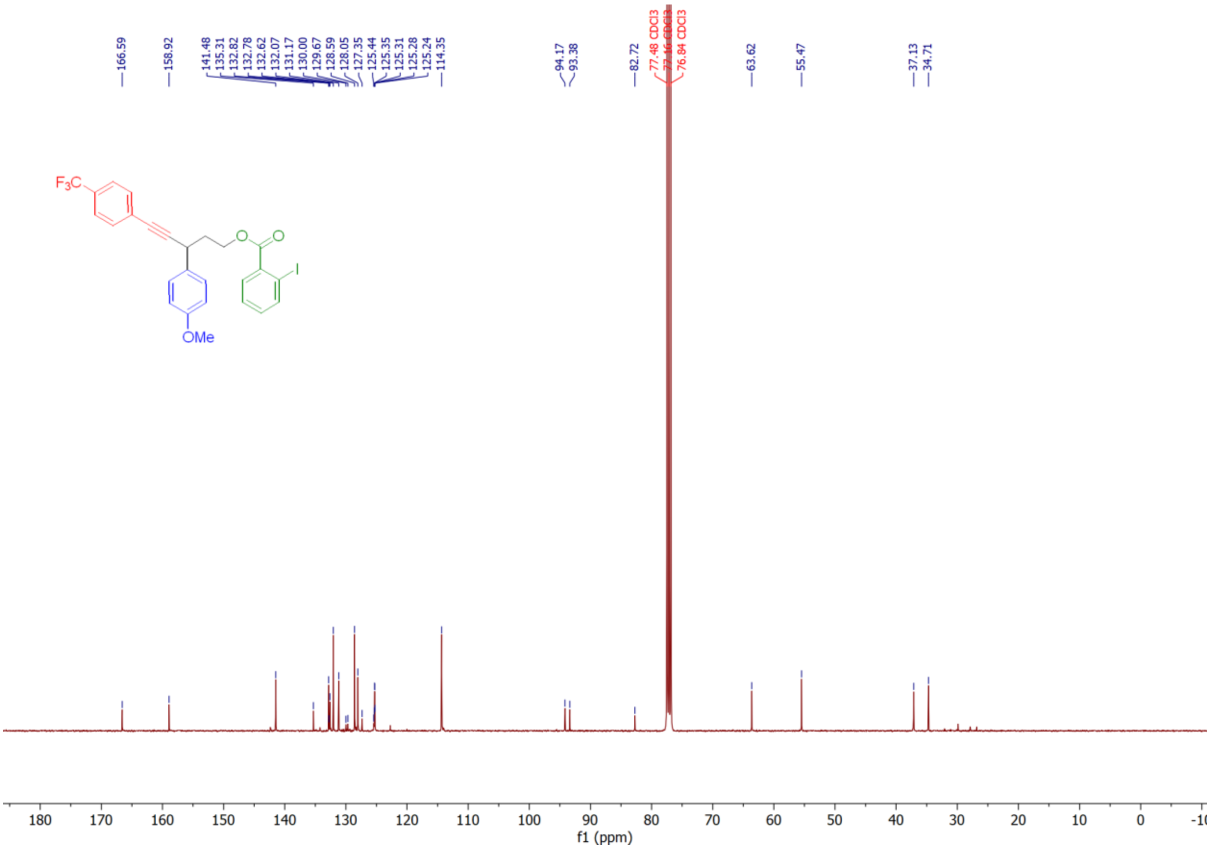

^{19}F NMR (377 MHz, $CDCl_3$) (3l)

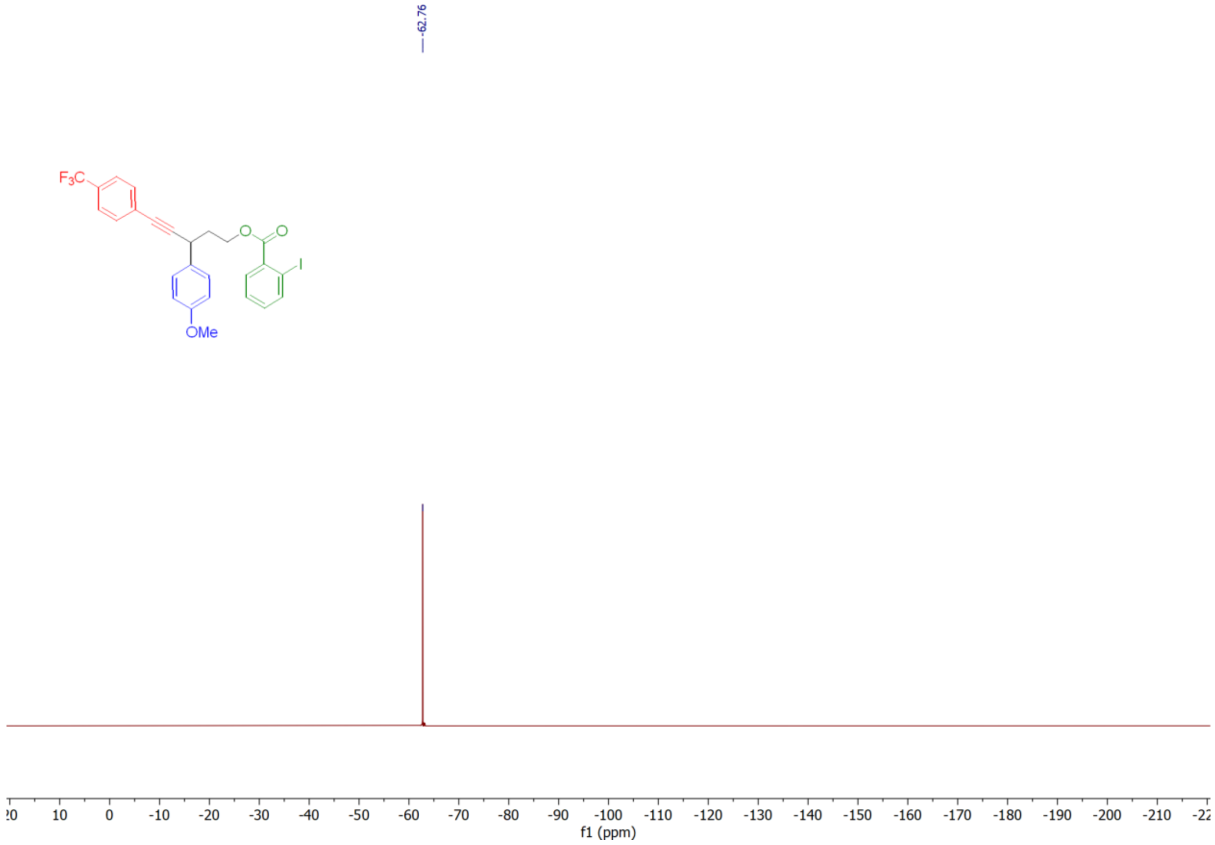

^{1}H NMR (400 MHz, $CDCl_3$) (3m)

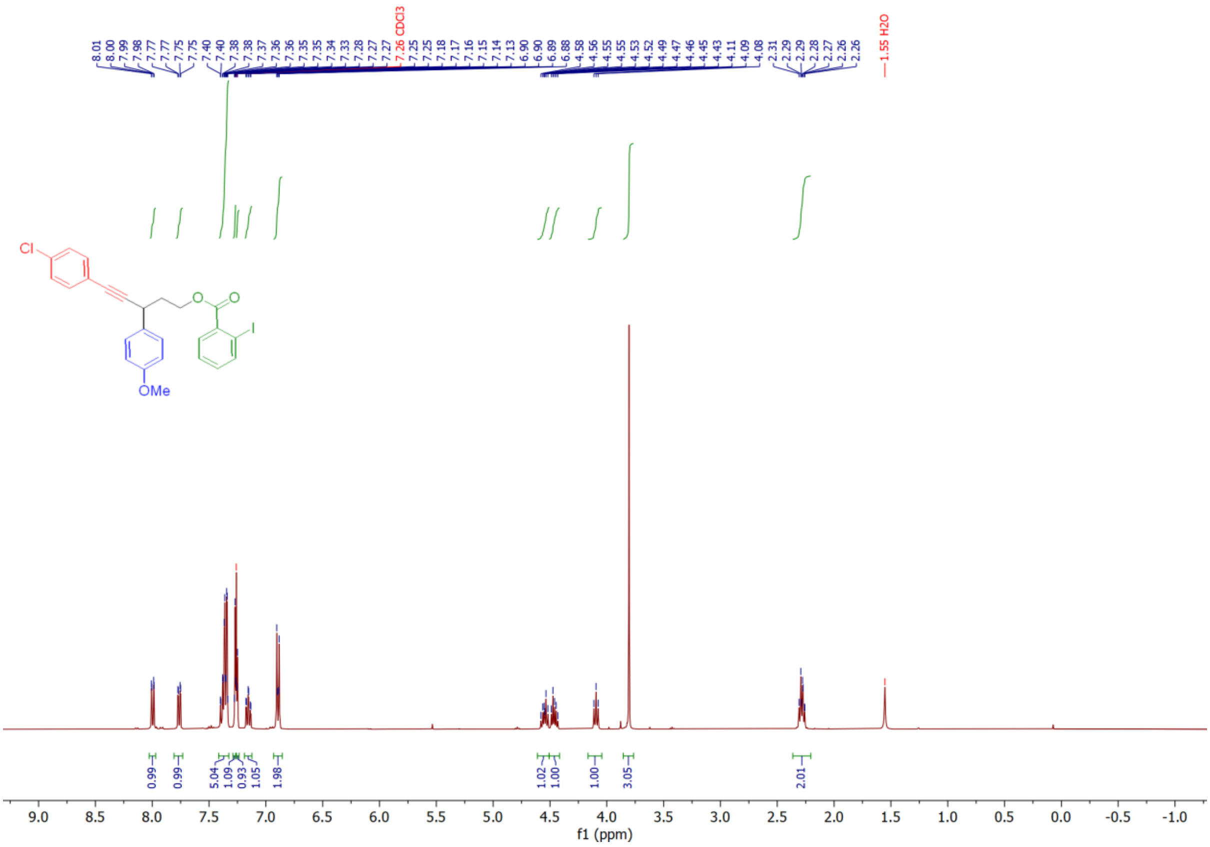

^{13}C NMR (101 MHz, $CDCl_3$) (3m)

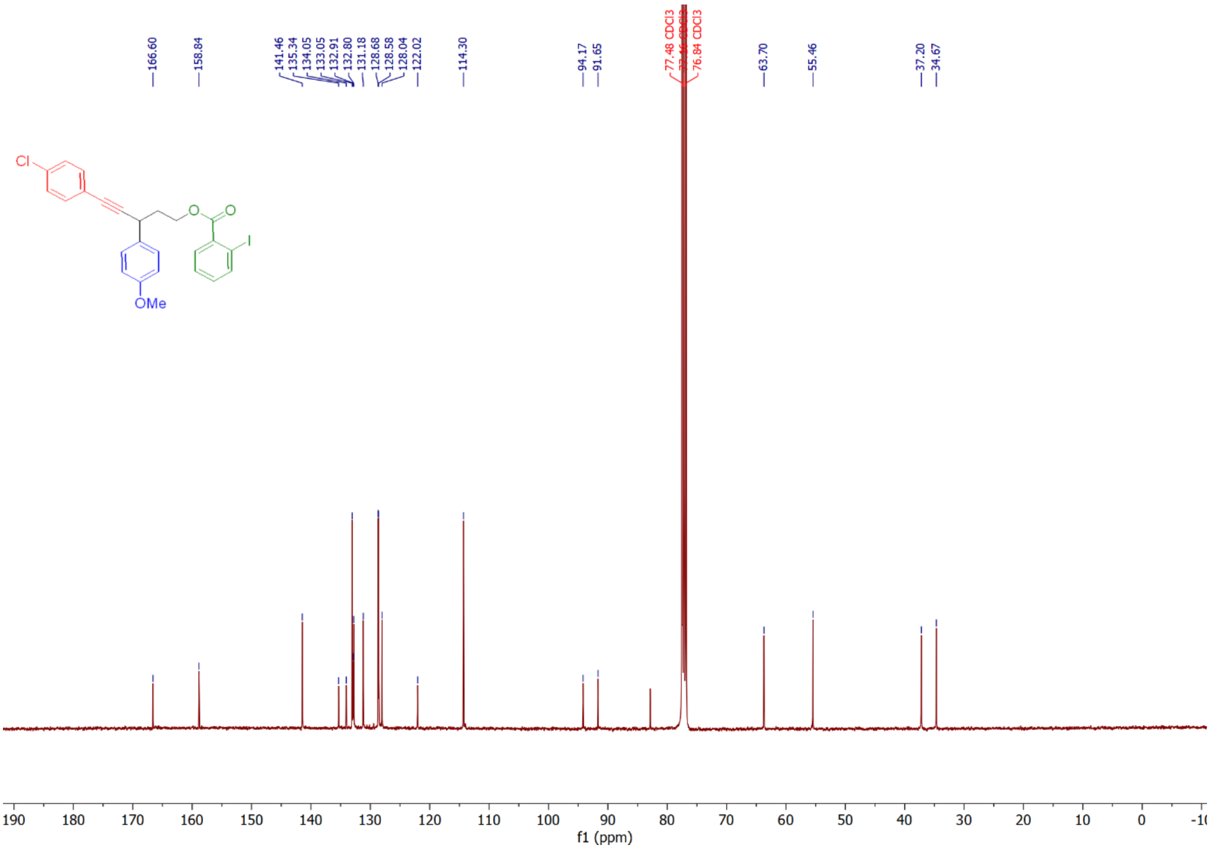

^{1}H NMR (400 MHz, $CDCl_3$) (3n)

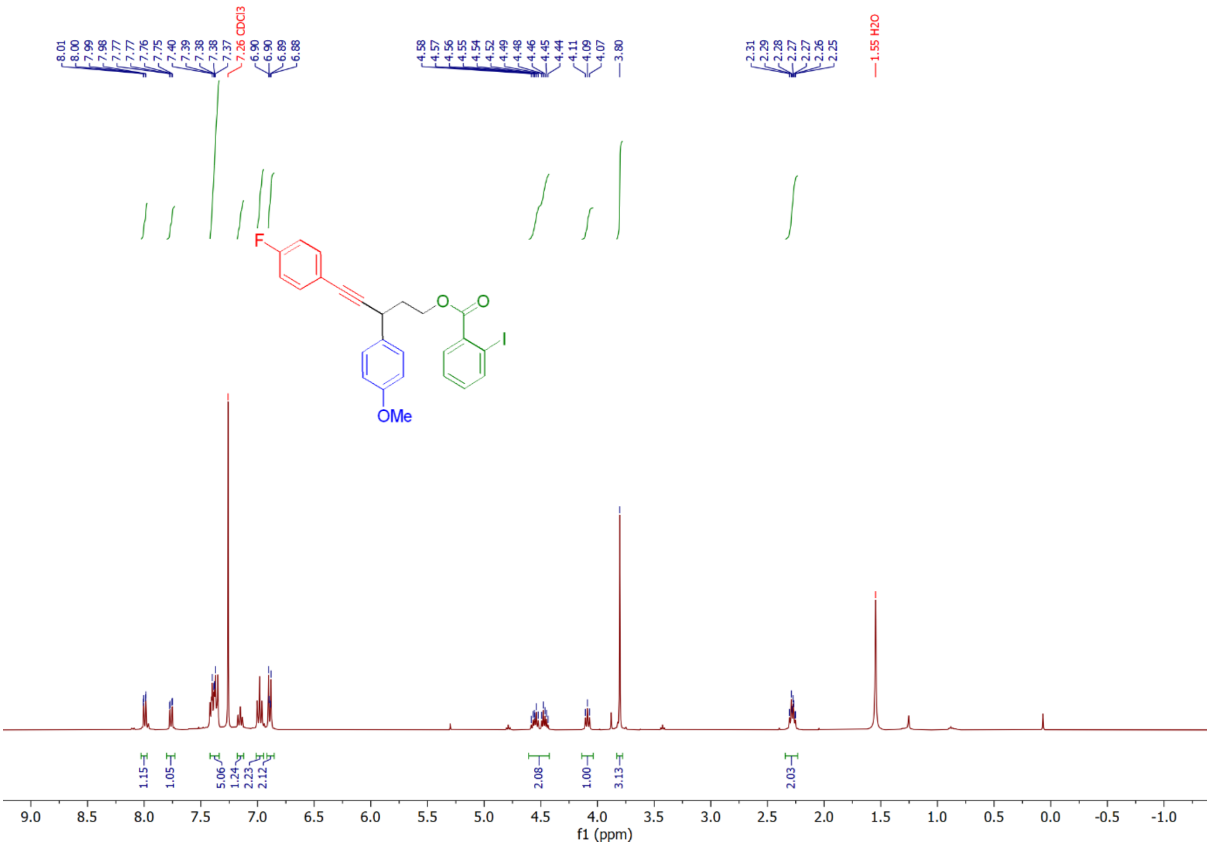

^{13}C NMR (201 MHz, $CDCl_3$) (3n)

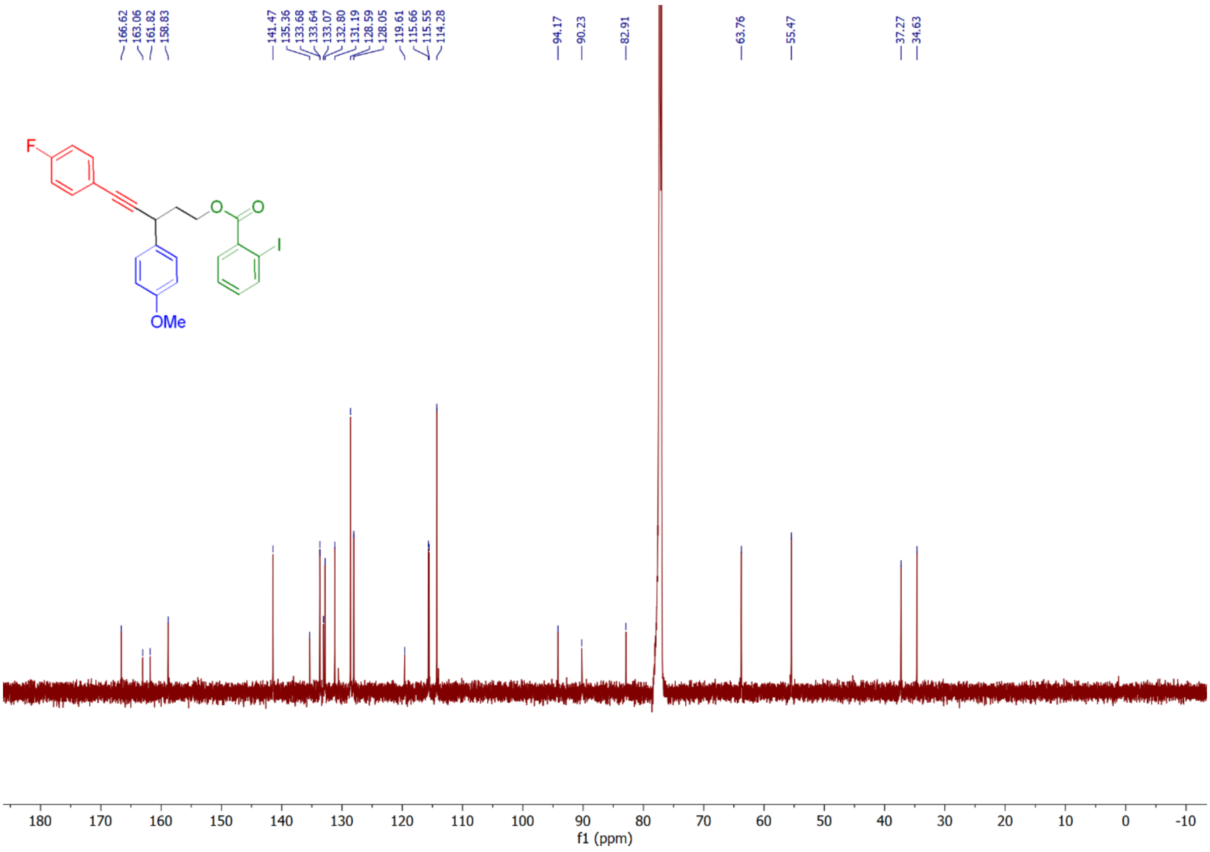

^{19}F NMR (377 MHz, $CDCl_3$) (3n)

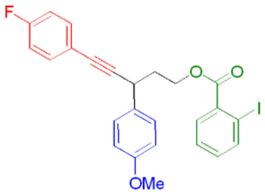

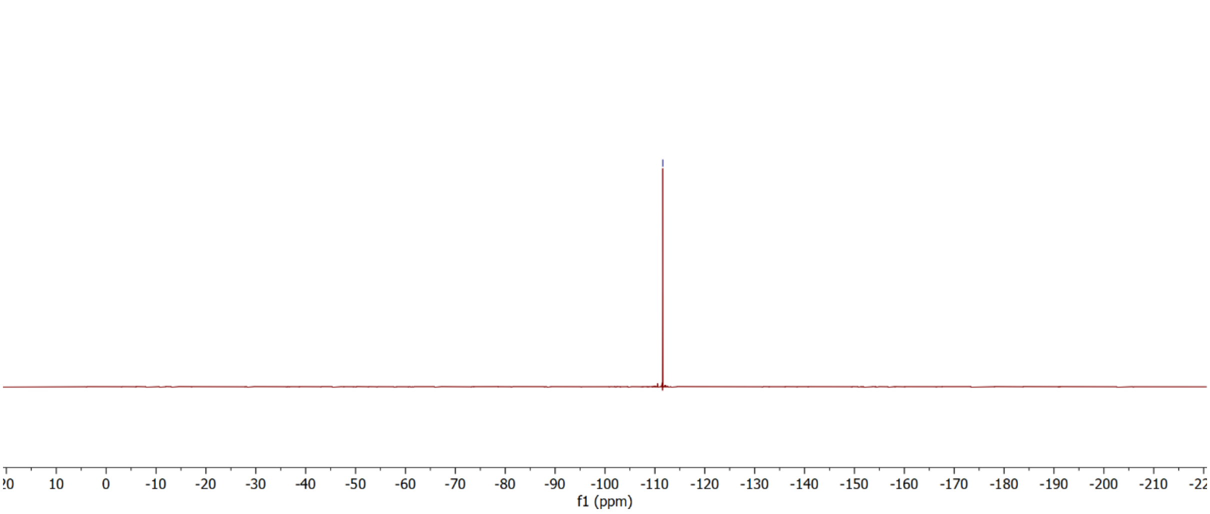

^{1}H NMR (400 MHz, $CDCl_3$) (3o)

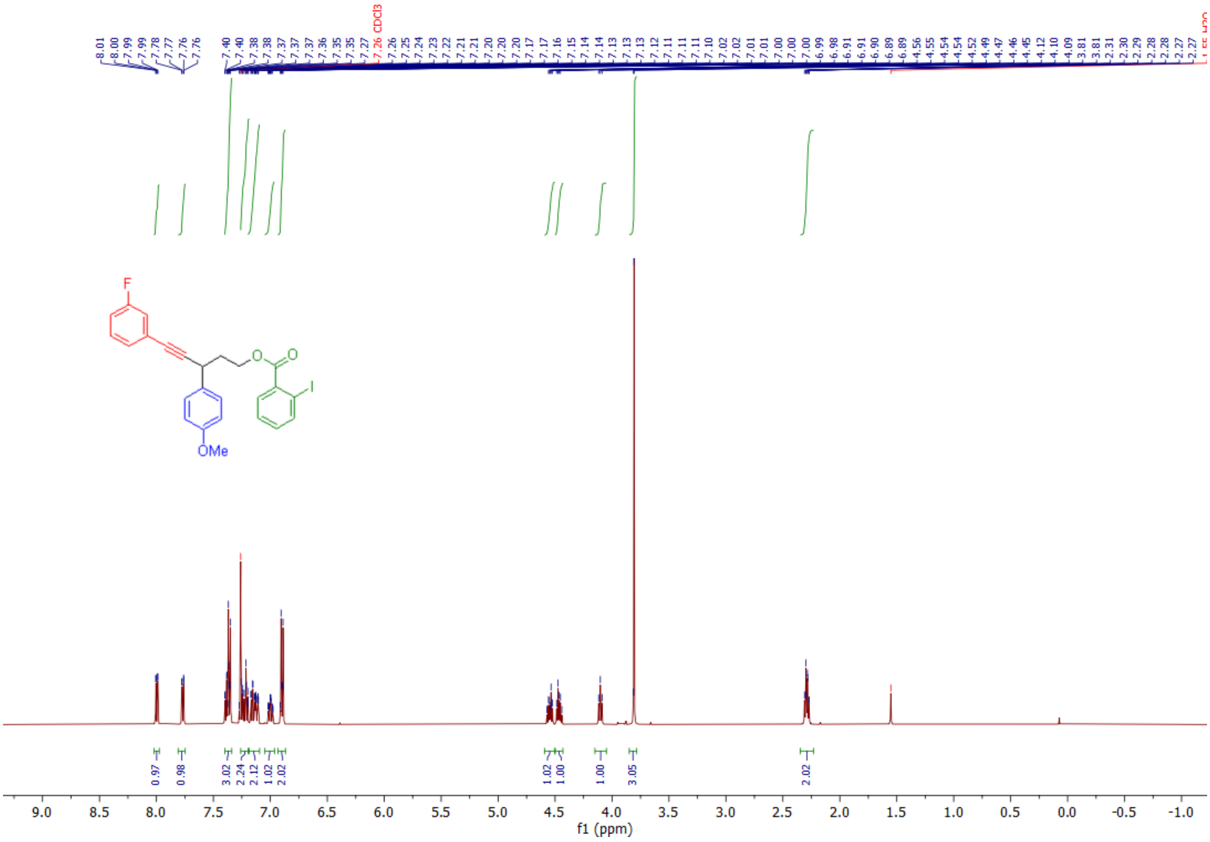

^{13}C NMR (101 MHz, $CDCl_3$) (3o)

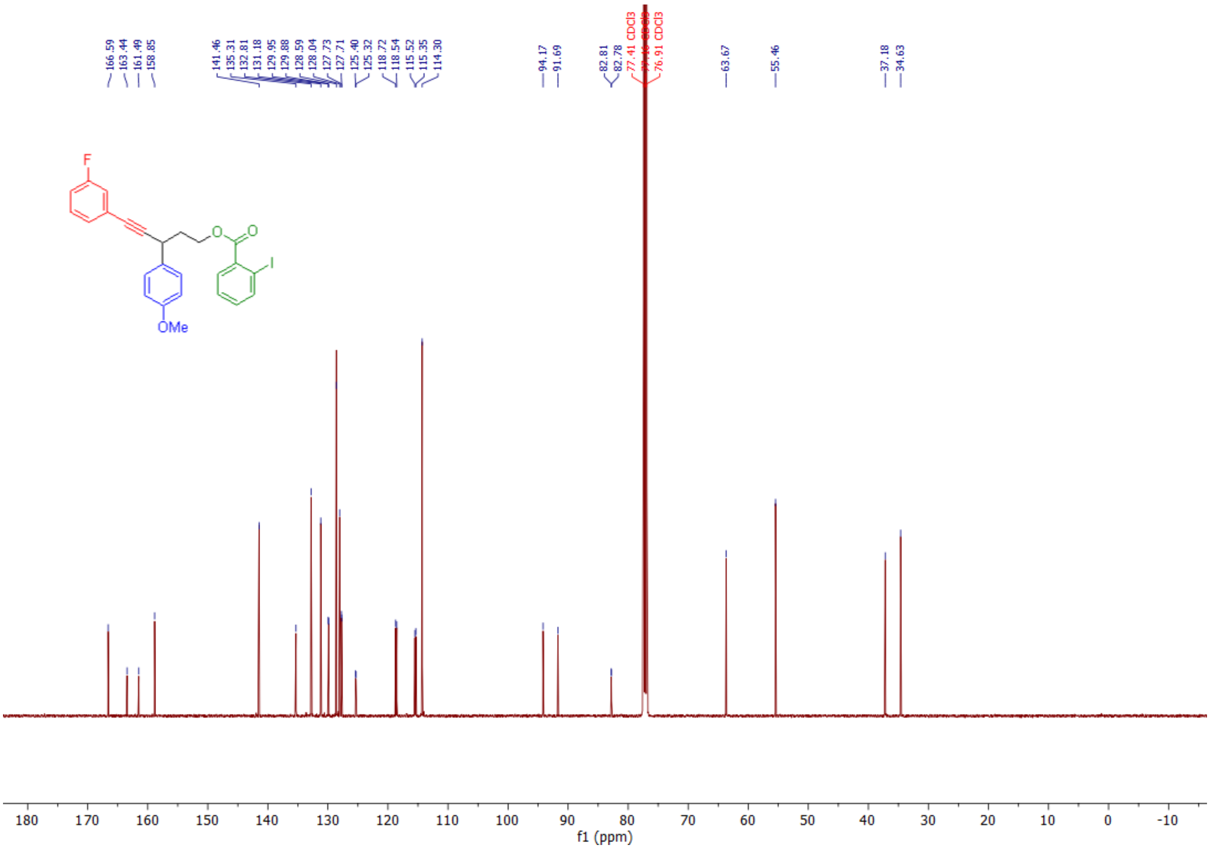

^{19}F NMR (377 MHz, $CDCl_3$) (3o)

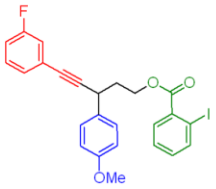

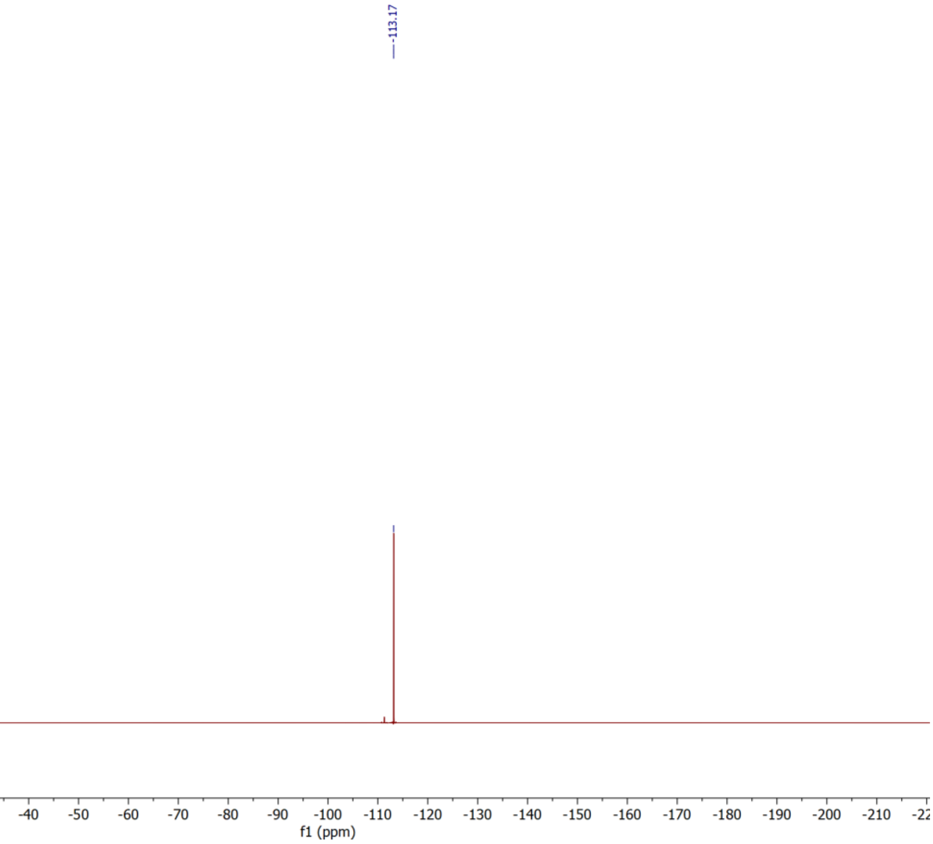

^{1}H NMR (400 MHz, $CDCl_3$) (3p)

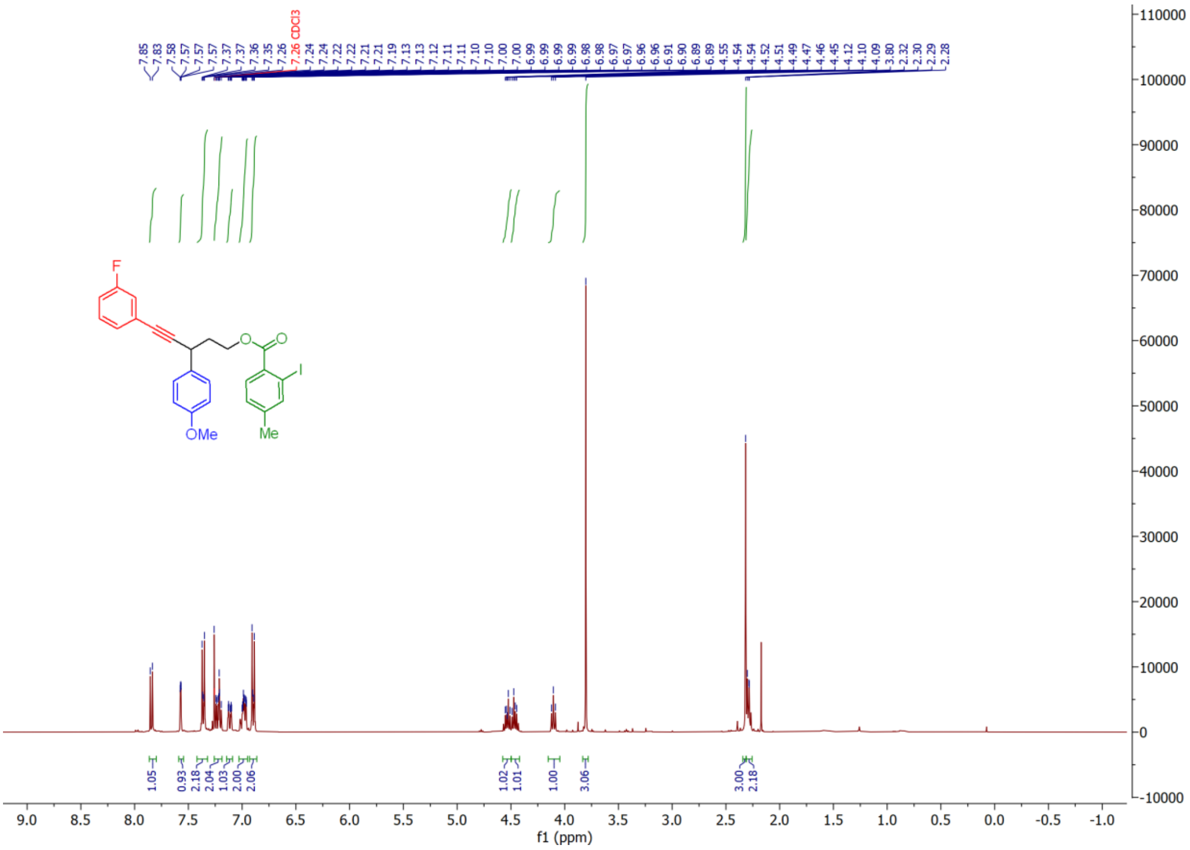

^{13}C NMR (101 MHz, $CDCl_3$) (3p)

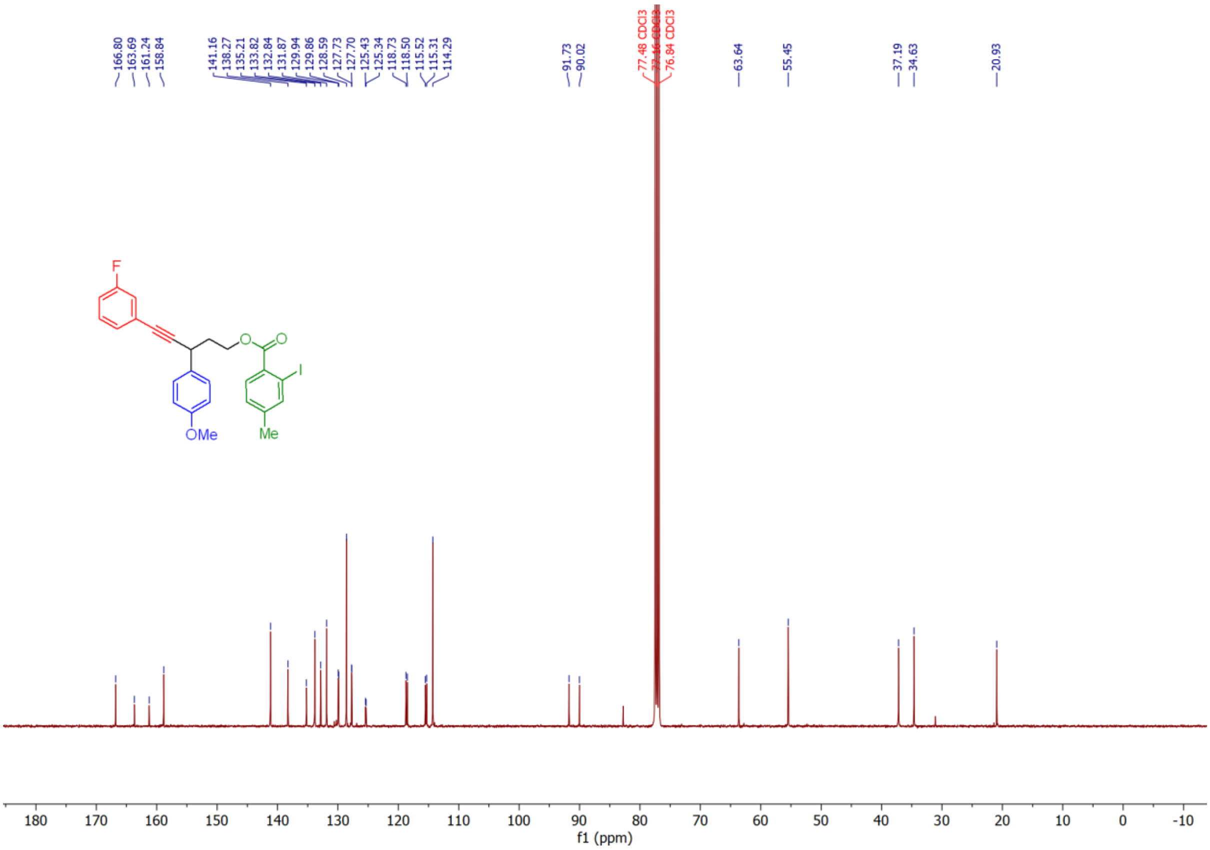

^{19}F NMR (377 MHz, CDCl$_3$) (3p)

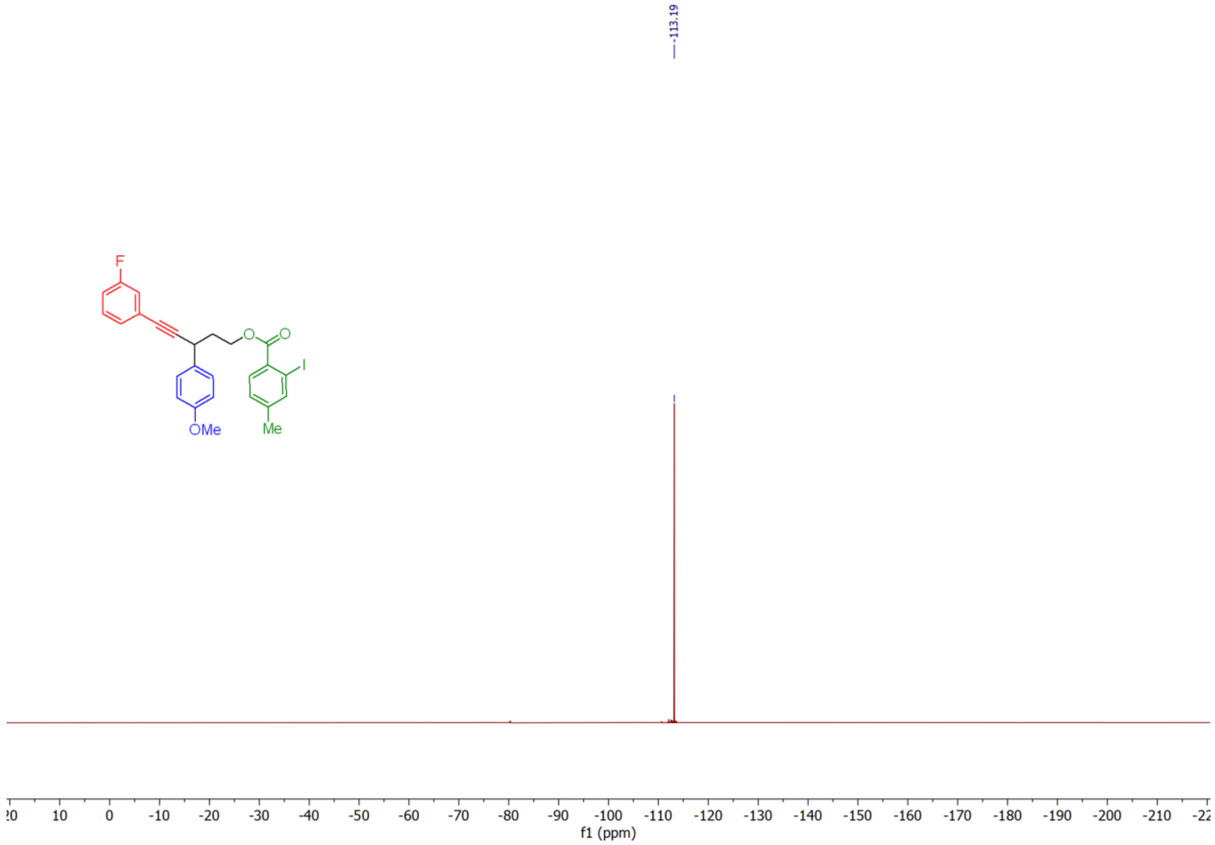

^{1}H NMR (400 MHz, $CDCl_3$) (3q)

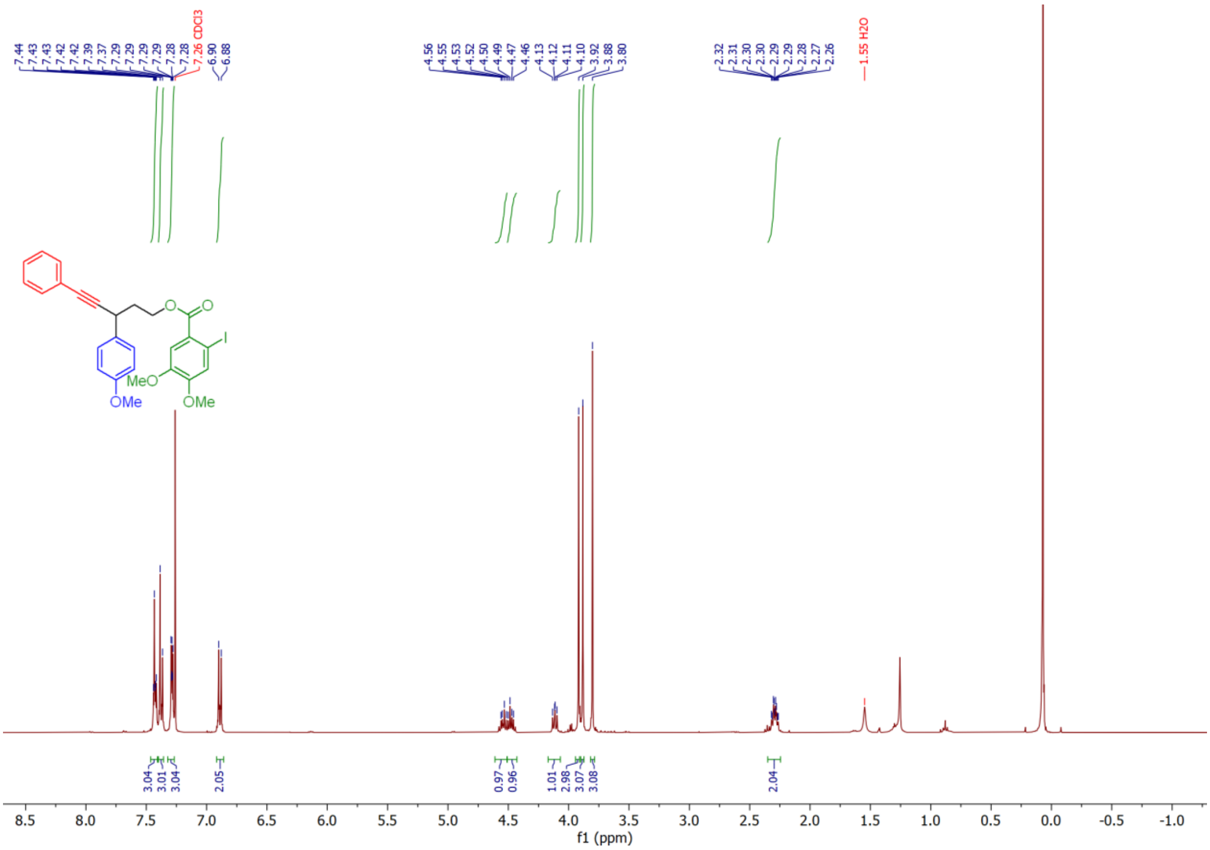

^{13}C NMR (201 MHz, $CDCl_3$) (3q)

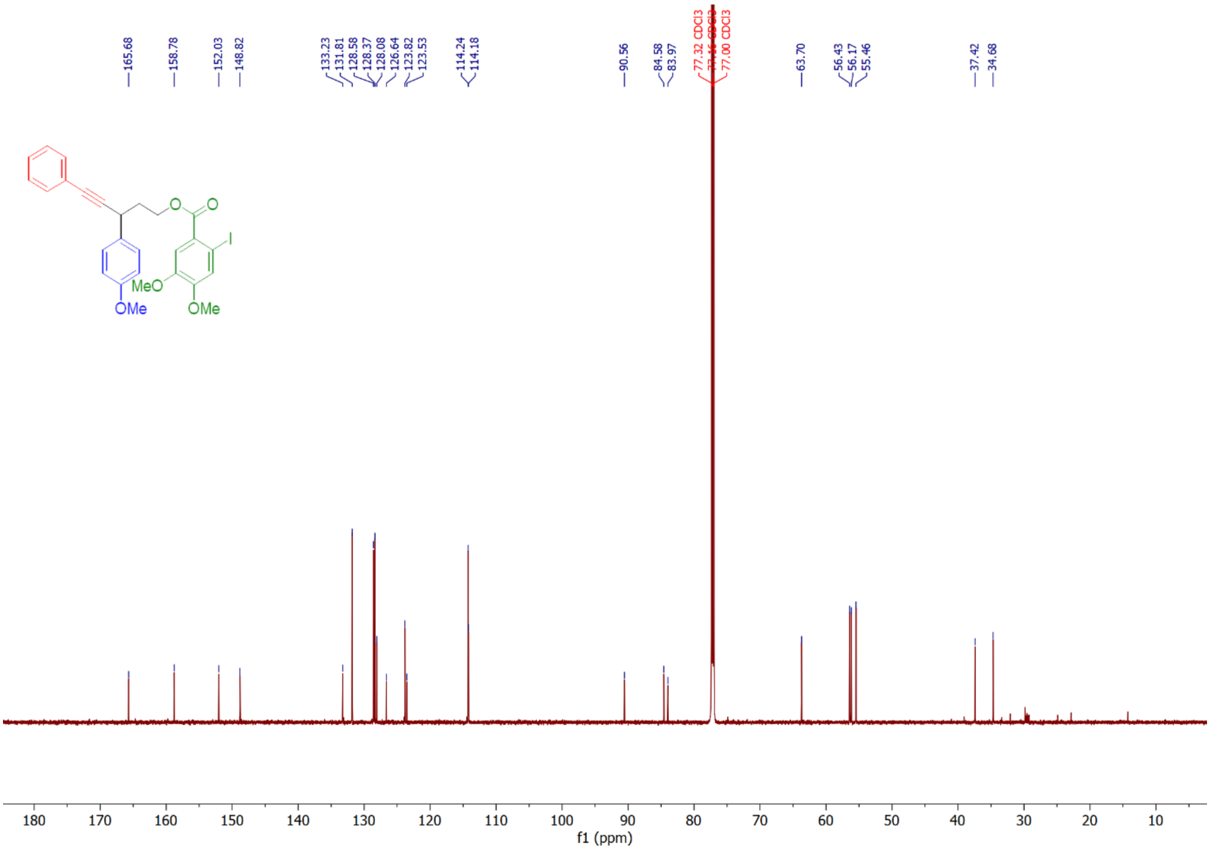

Blue: Major diastereomer, *Orange : Minor diastereomer

^{1}H NMR (400 MHz, $CDCl_3$ (8a)

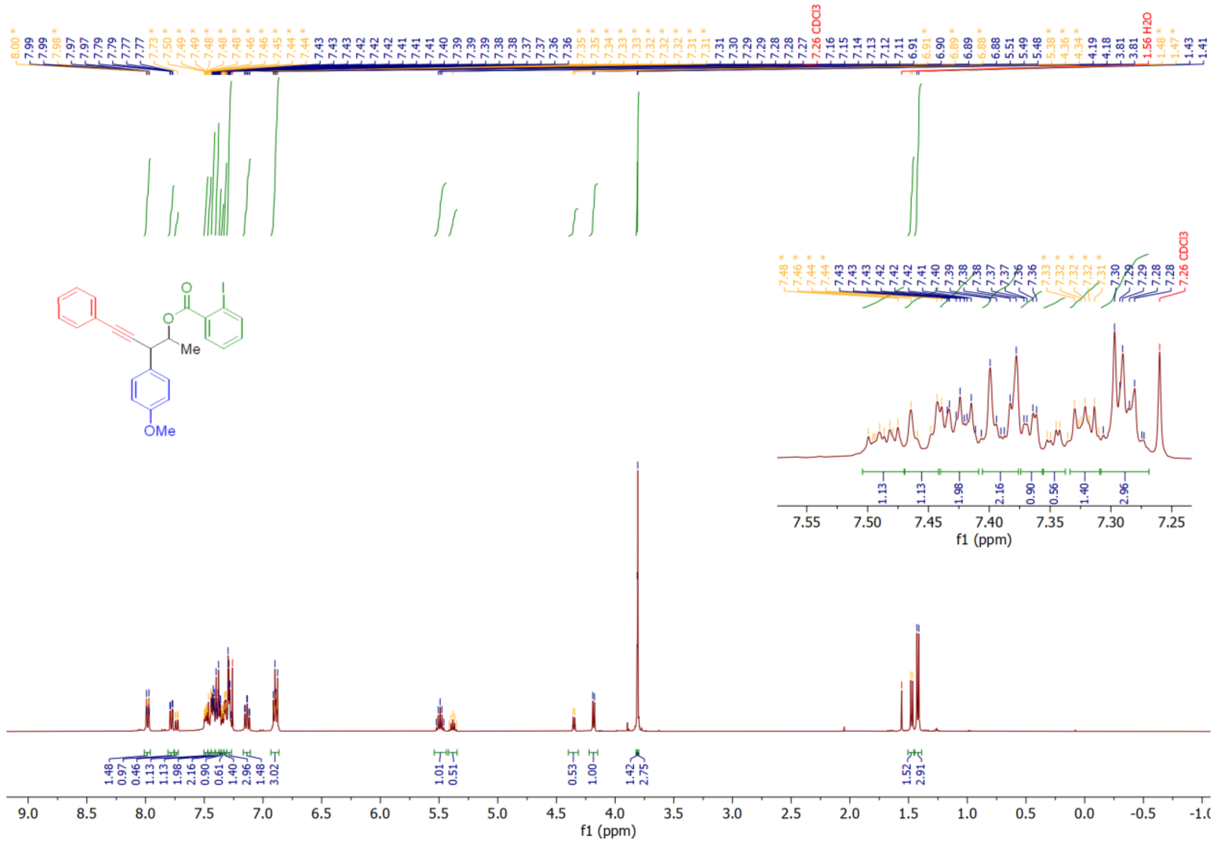

^{13}C NMR (101 MHz $CDCl_3$ (8a)

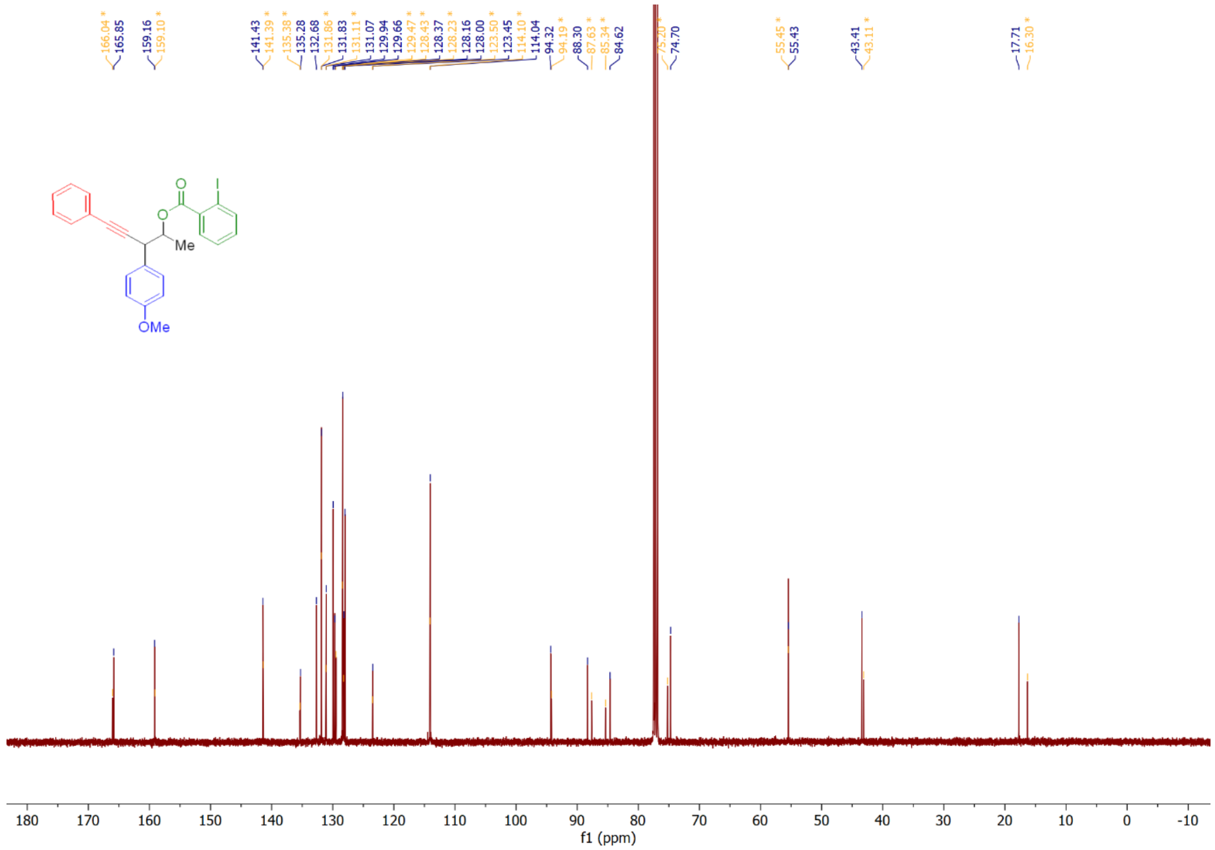

^{1}H NMR (400 MHz, $CDCl_3$) (8b)

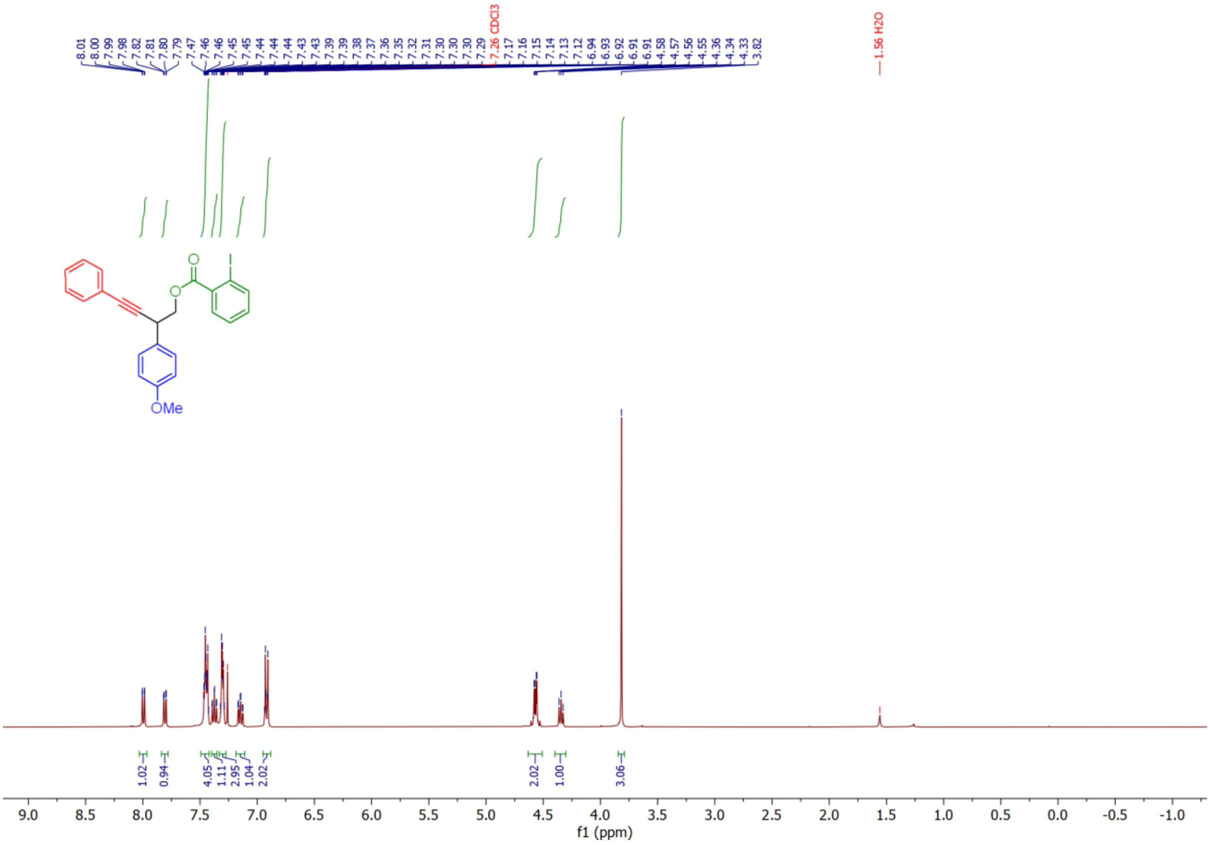

^{13}C NMR (101 MHz, $CDCl_3$) (8b)

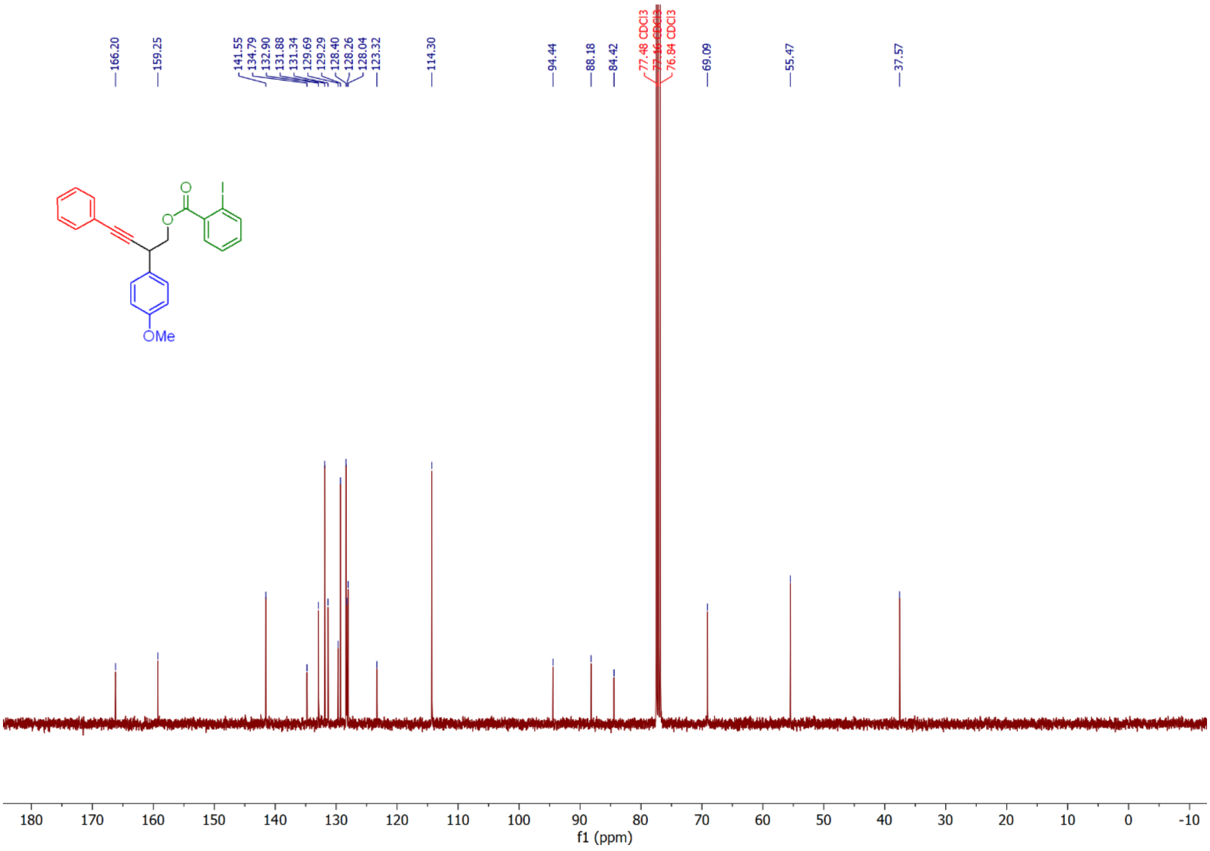

^{1}H NMR (400 MHz, $CDCl_3$ (8c)

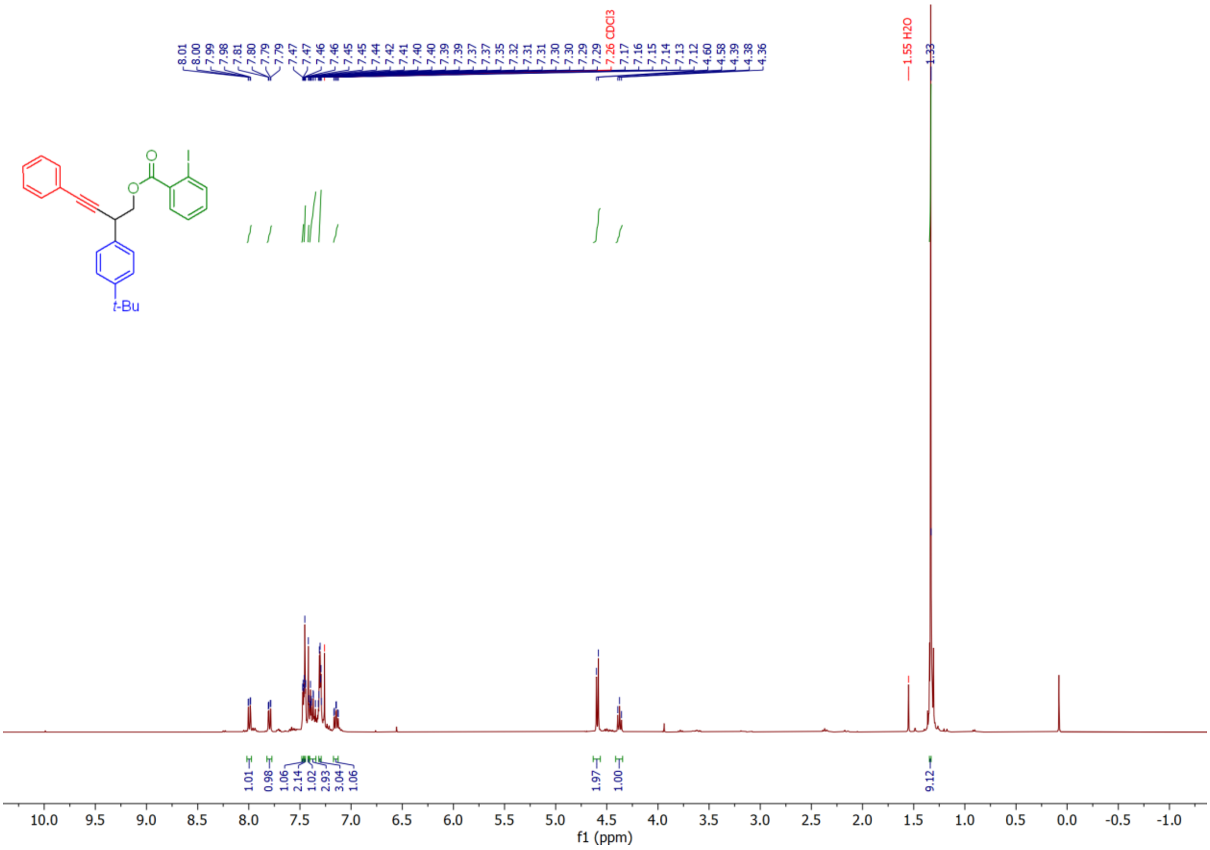

^{13}C NMR (101 MHz, $CDCl_3$) (8c)

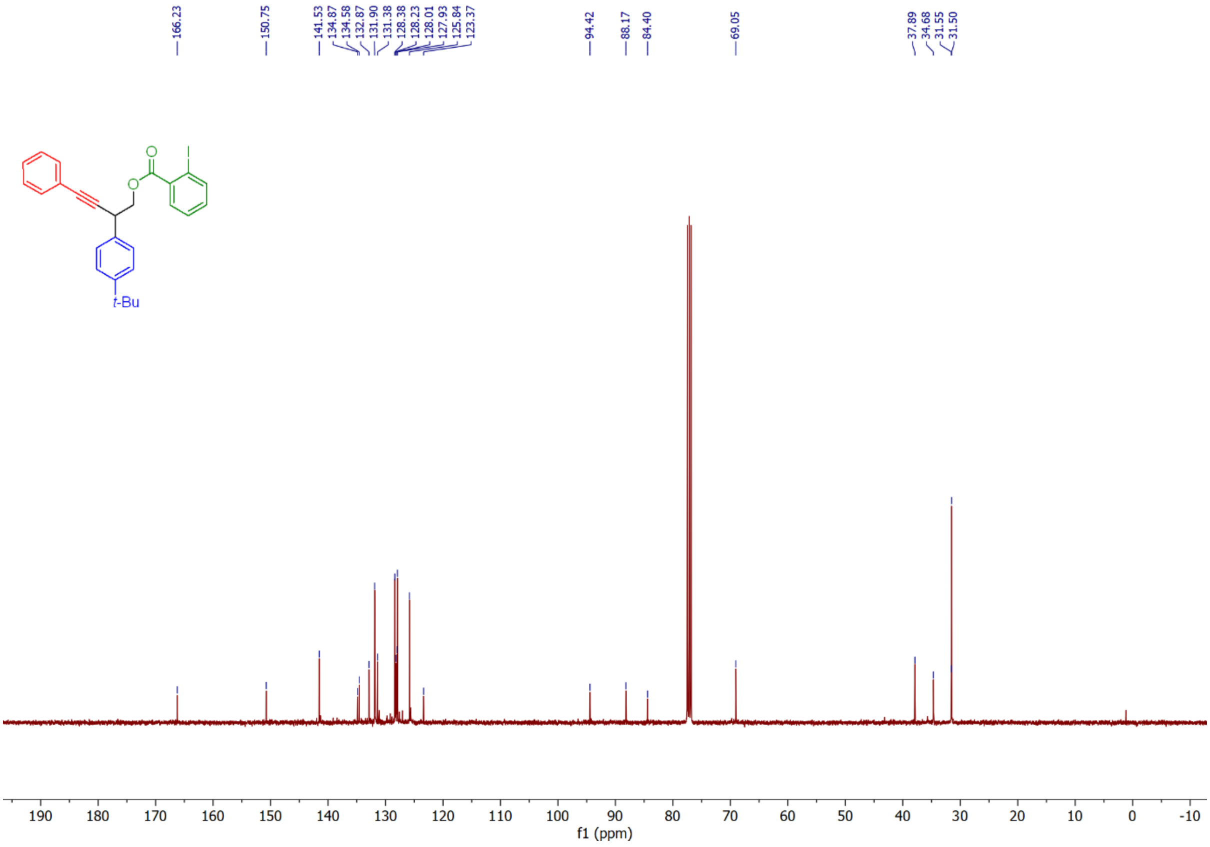

^{1}H NMR (400 MHz, $CDCl_3$) (4a)

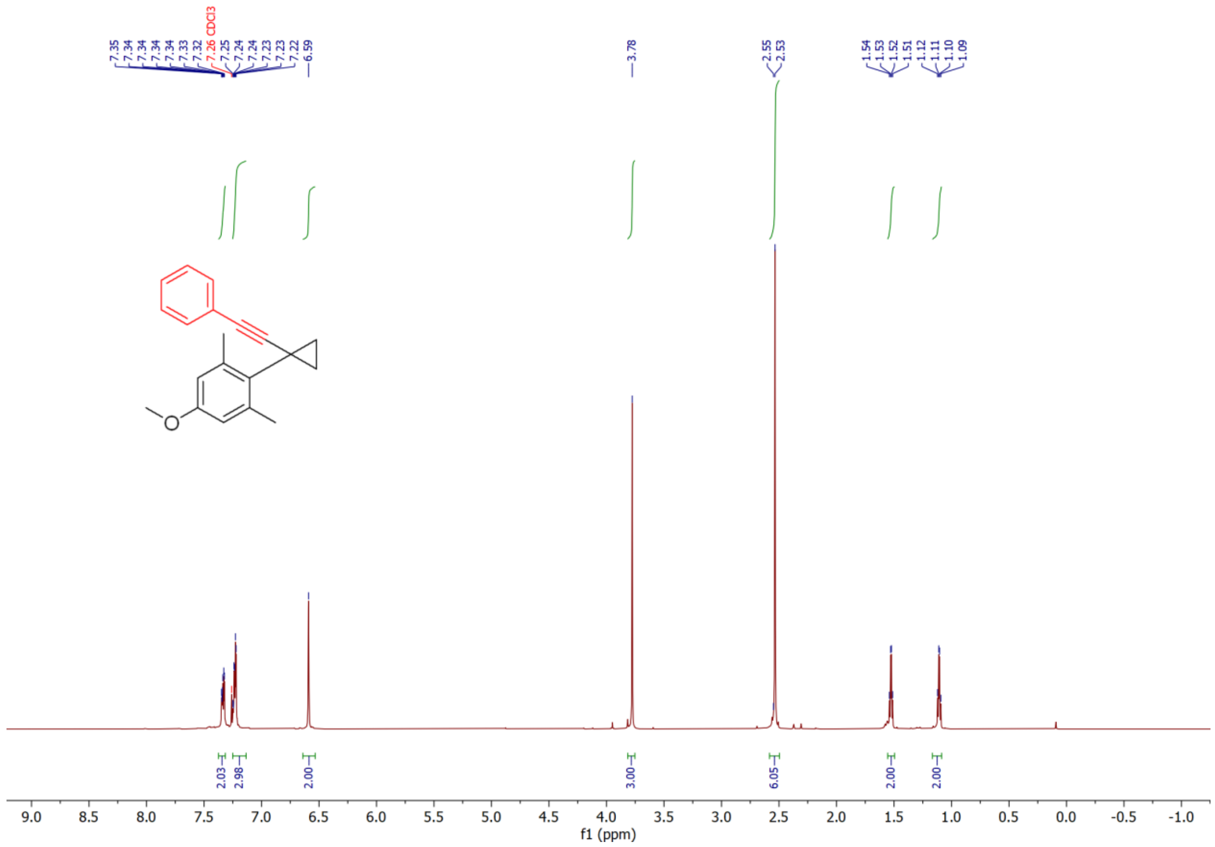

^{13}C NMR (101 MHz, $CDCl_3$) (4a)

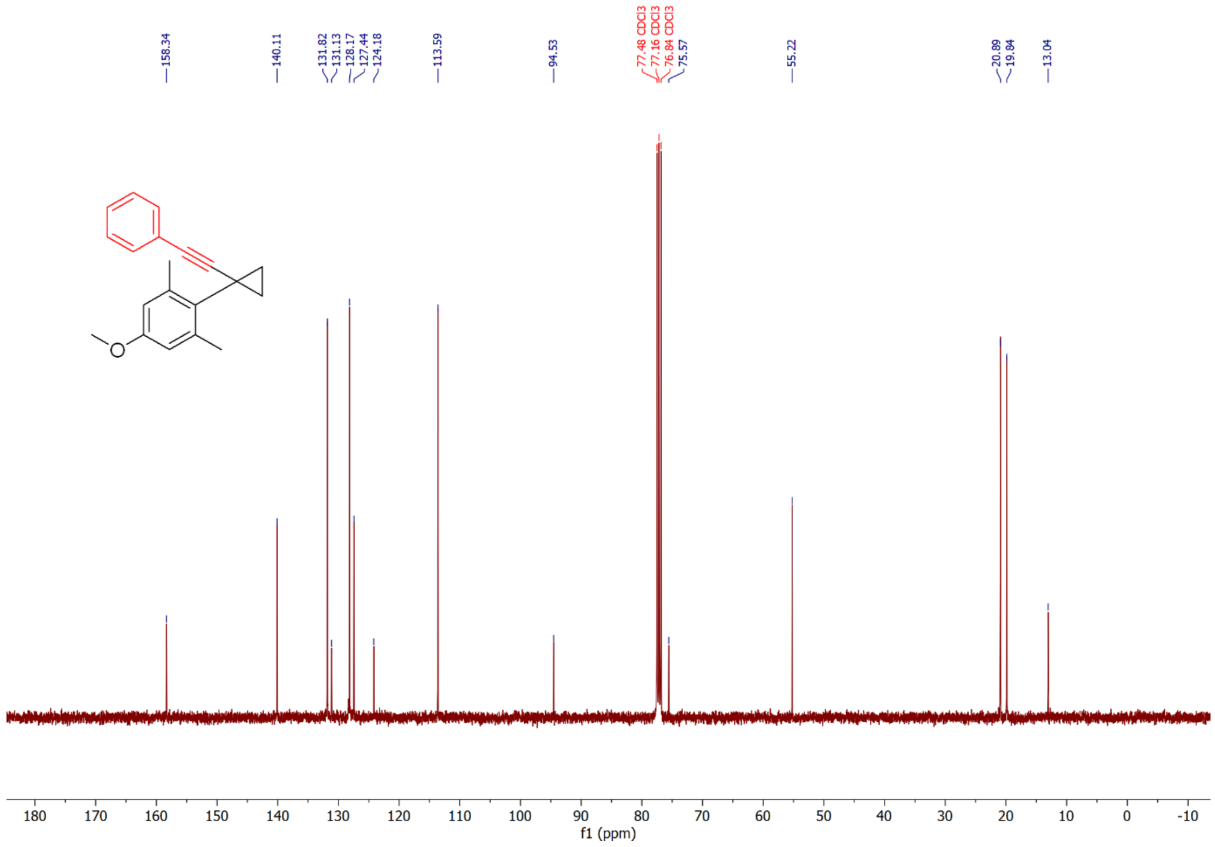

^{1}H NMR (400 MHz, $CDCl_3$) (4b)

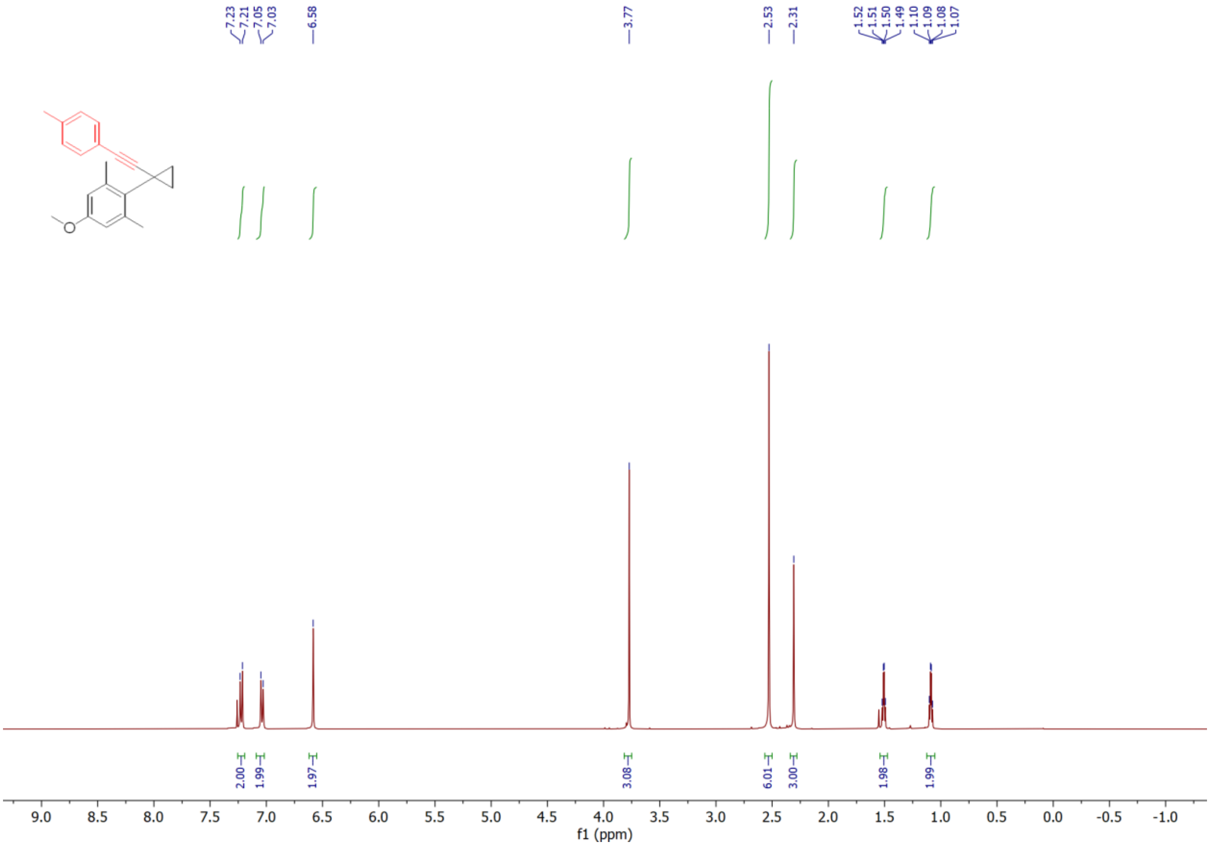

^{13}C NMR (101 MHz, $CDCl_3$) (4b)

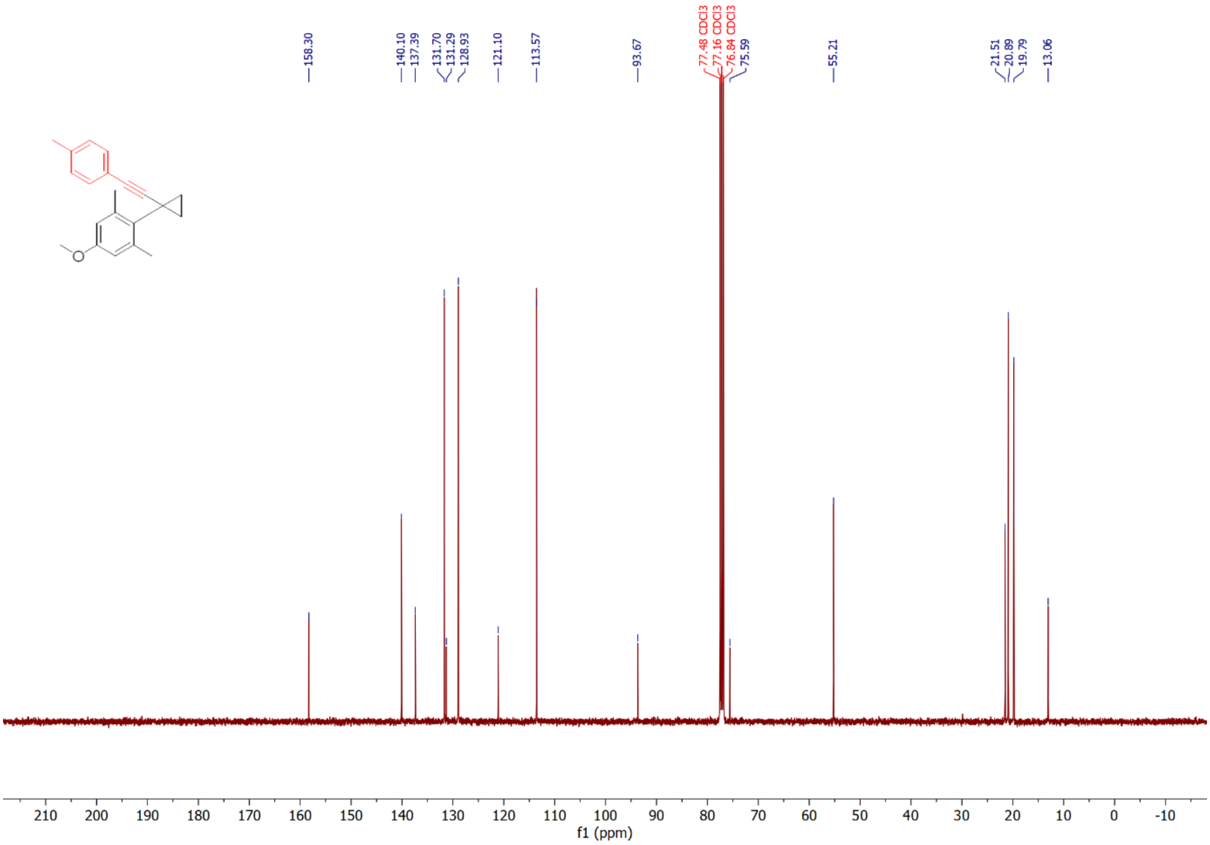

^{1}H NMR (400 MHz, $CDCl_3$) (4c)

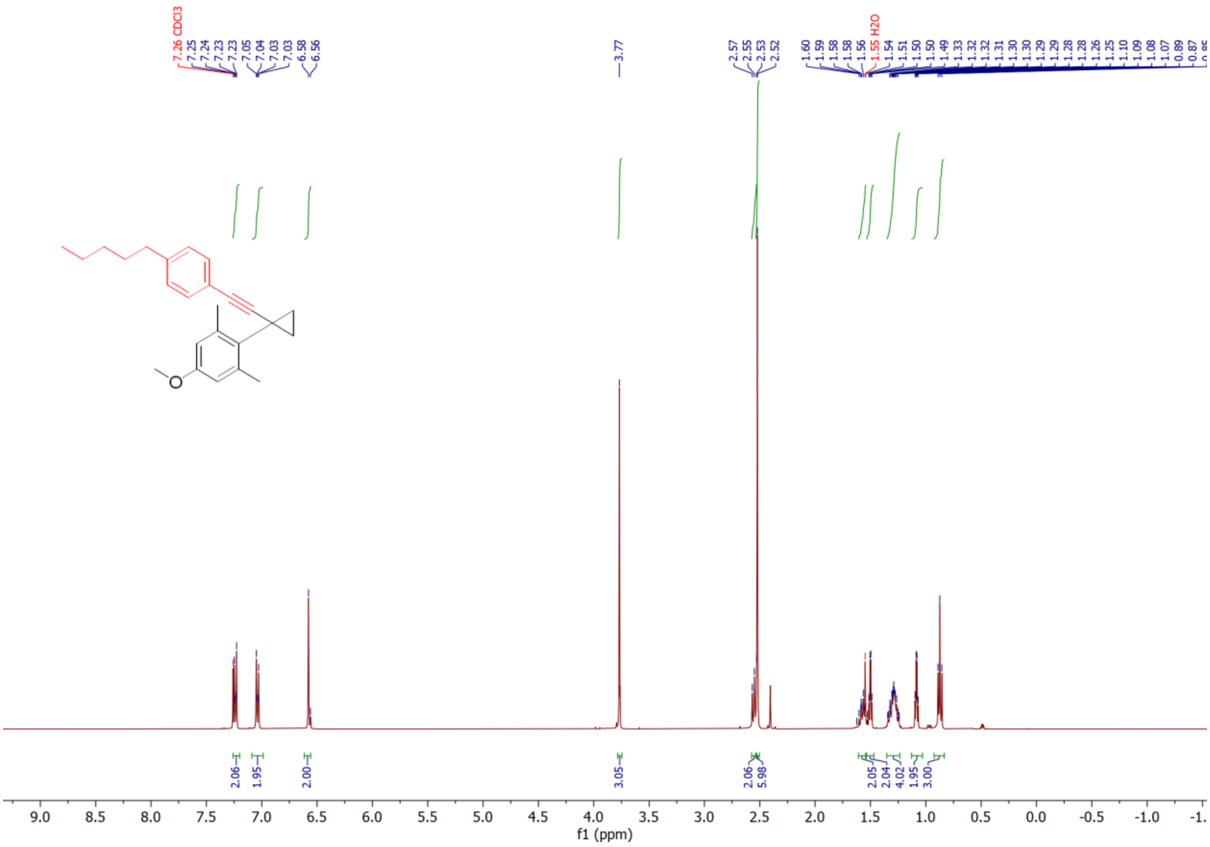

^{13}C NMR (101 MHz, $CDCl_3$) (4c)

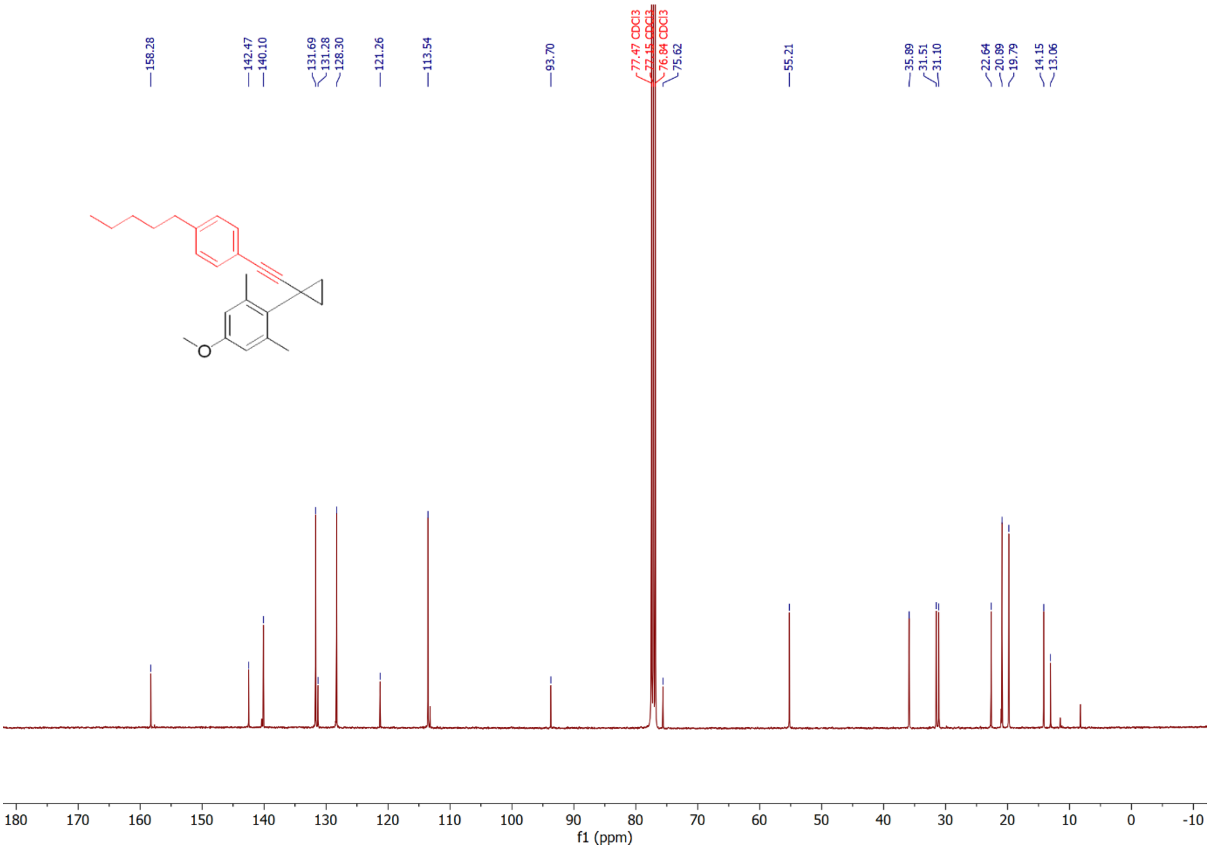

^{1}H NMR (400 MHz, $CDCl_3$) (4d)

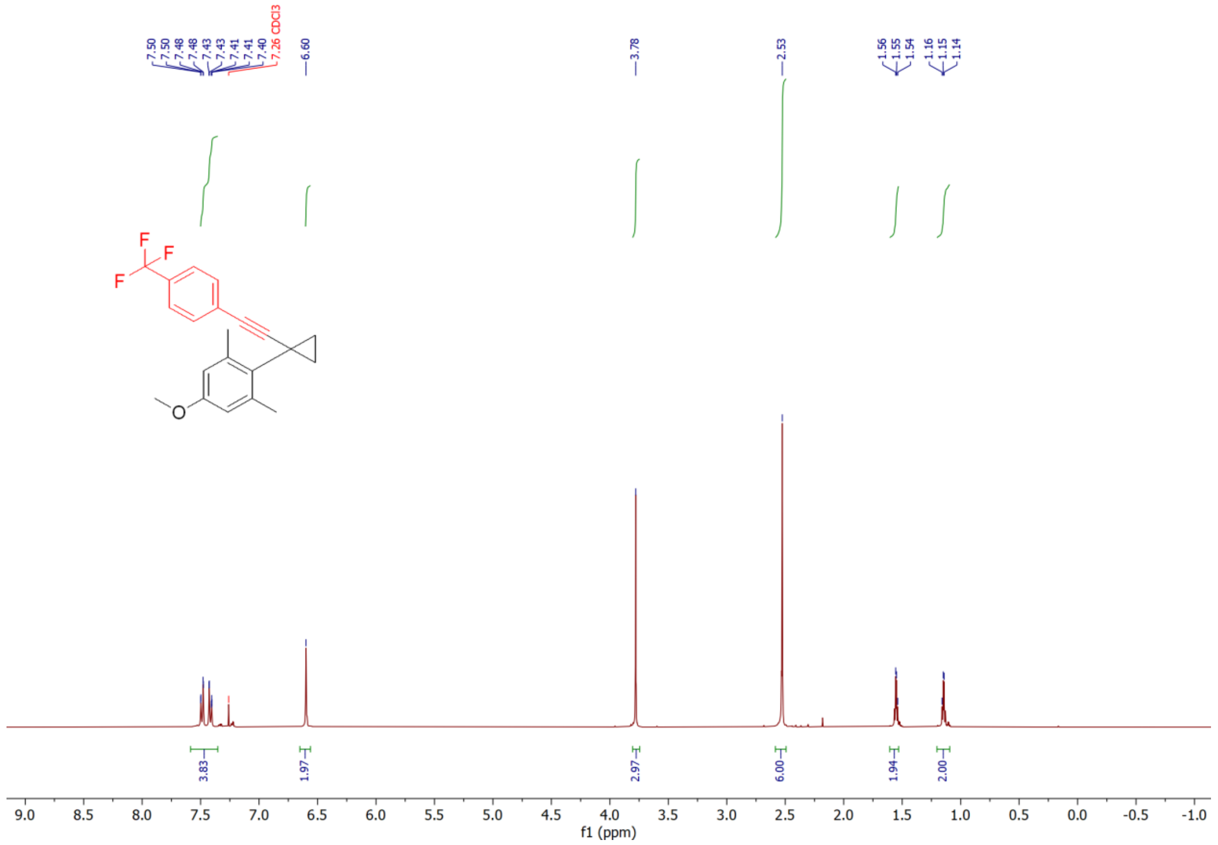

^{13}C NMR (101 MHz, $CDCl_3$) (4d)

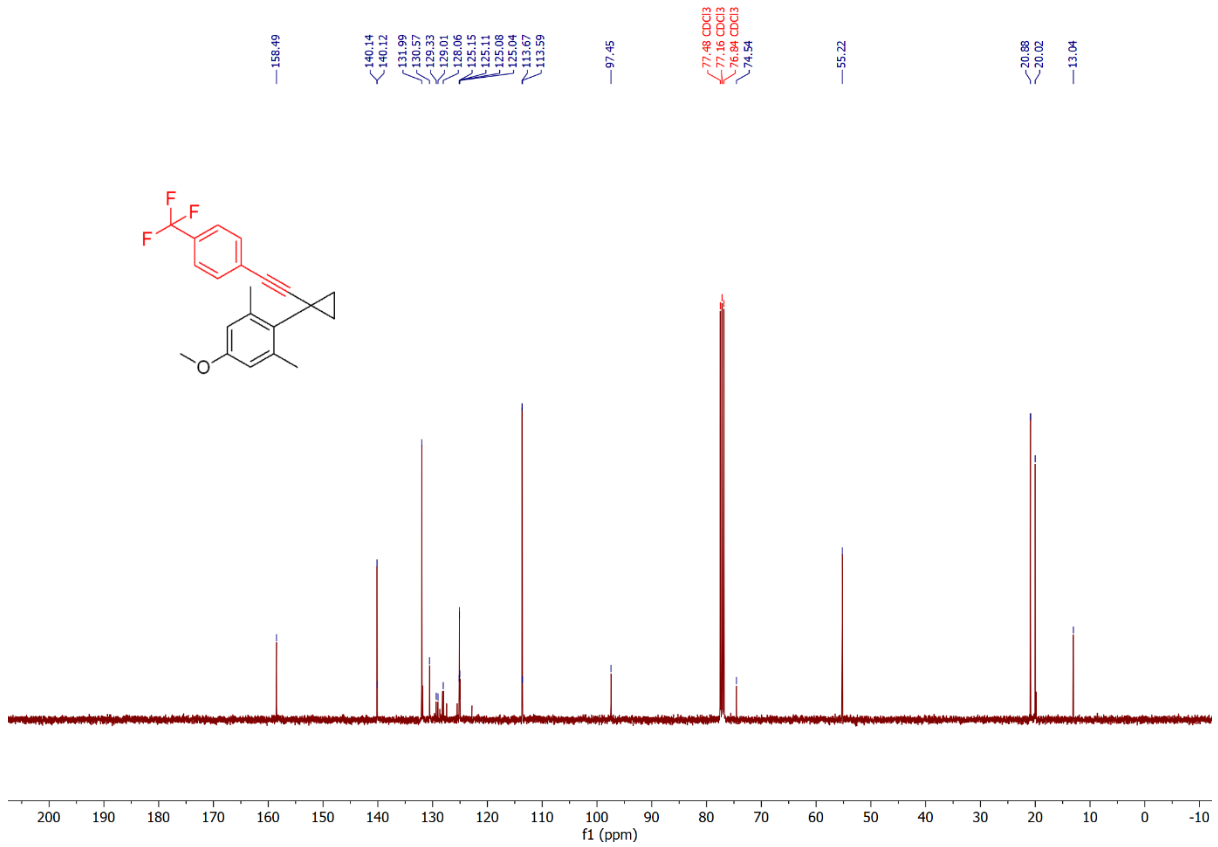

^{19}F NMR (377 MHz, $CDCl_3$) (4d)

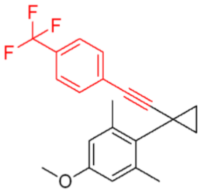

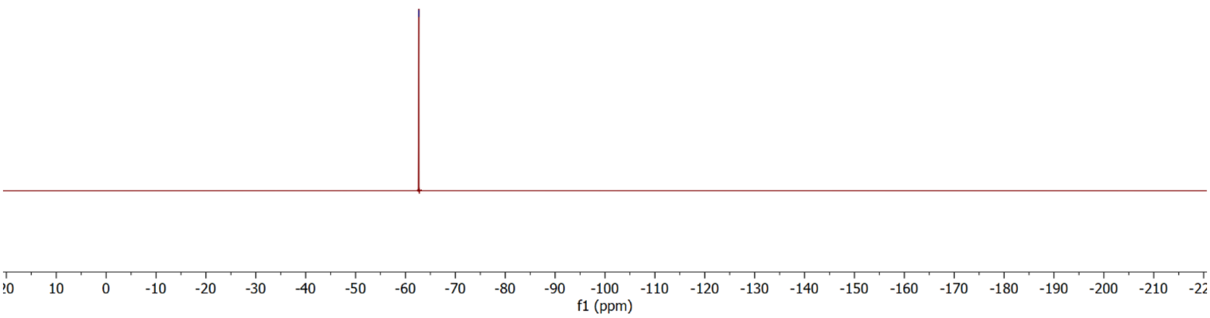

^{1}H NMR (400 MHz, $CDCl_3$) (4e)

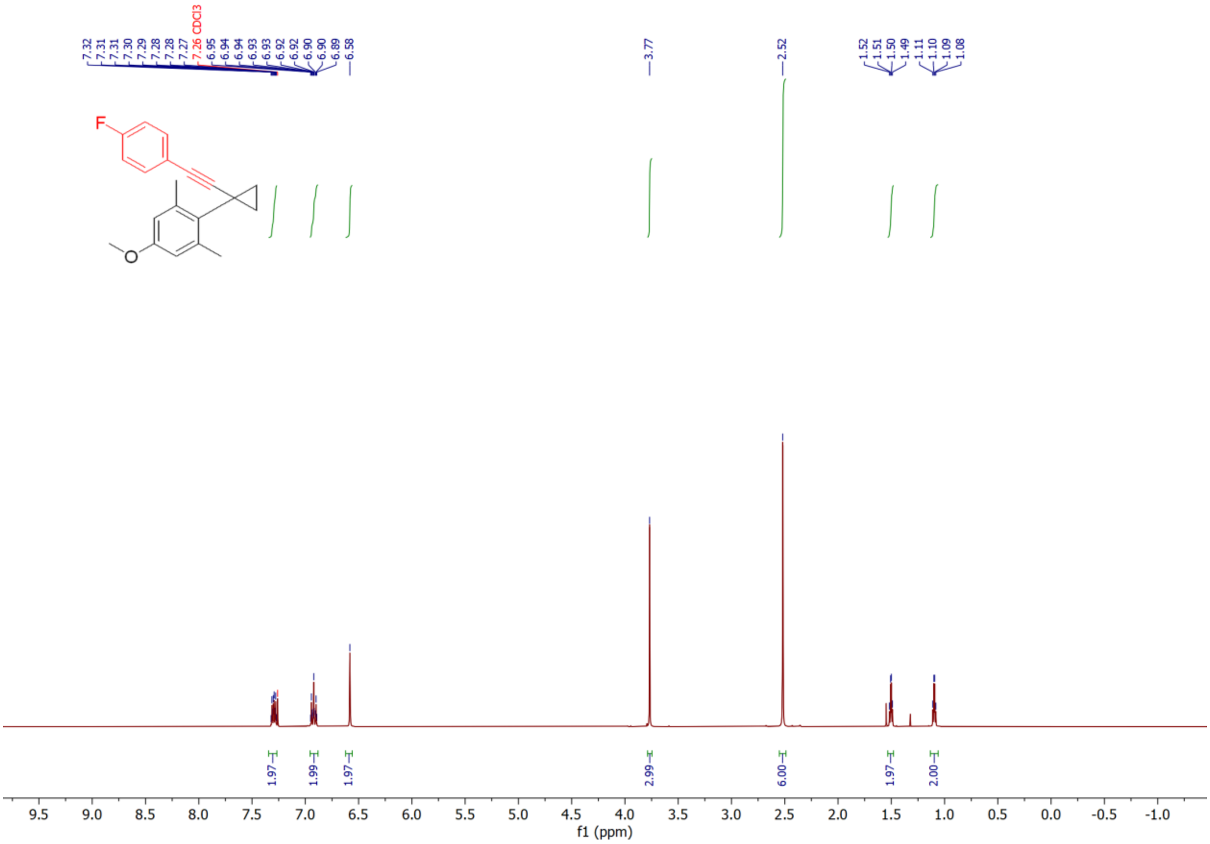

^{13}C NMR (101 MHz, $CDCl_3$) (4e)

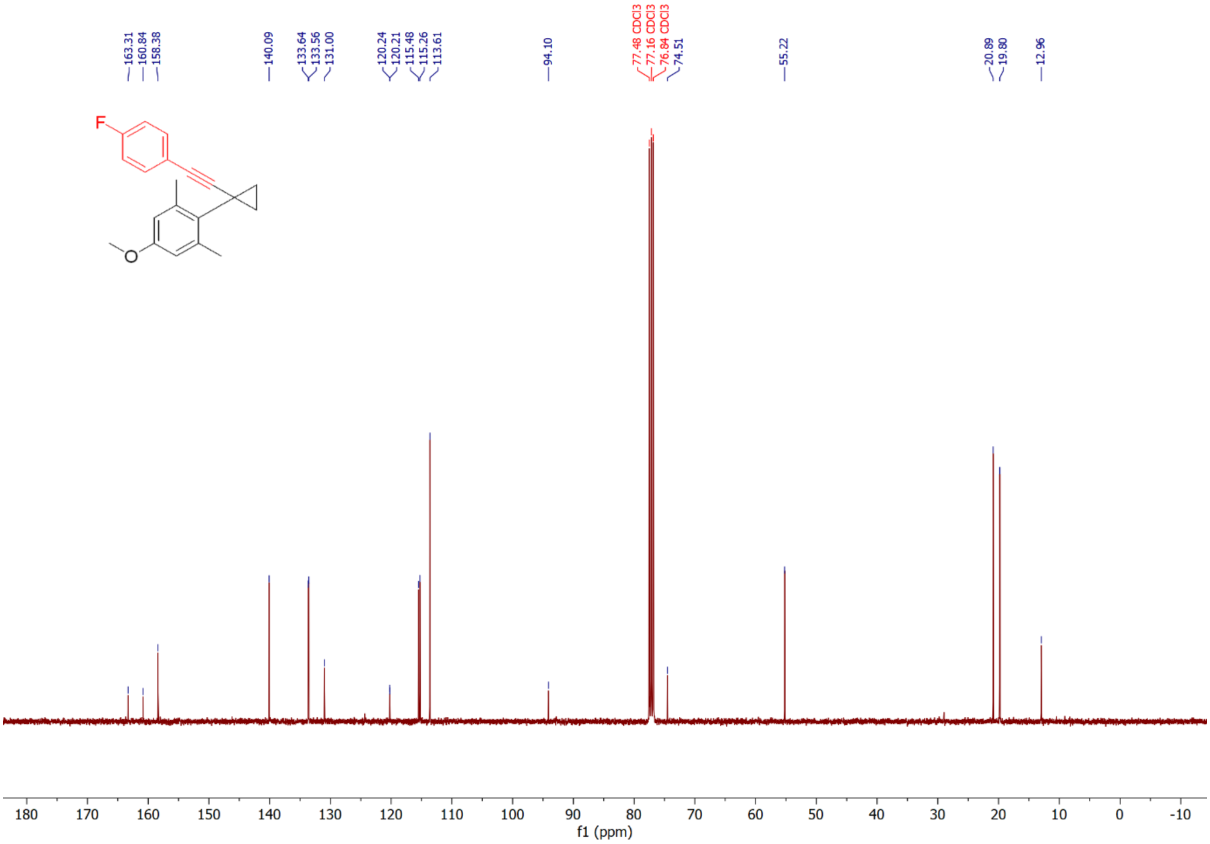

^{19}F NMR (377 MHz, $CDCl_3$) **(4e)**

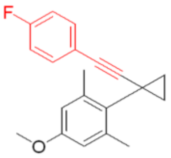

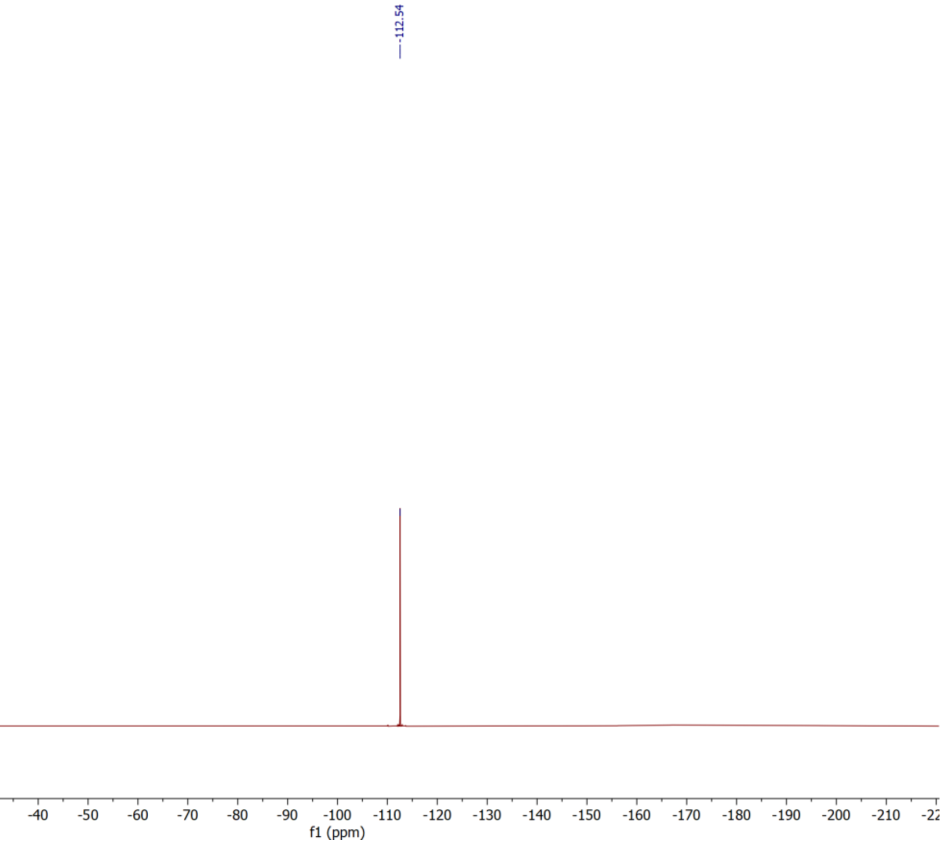

^{1}H NMR (400 MHz, $CDCl_3$) (4f)

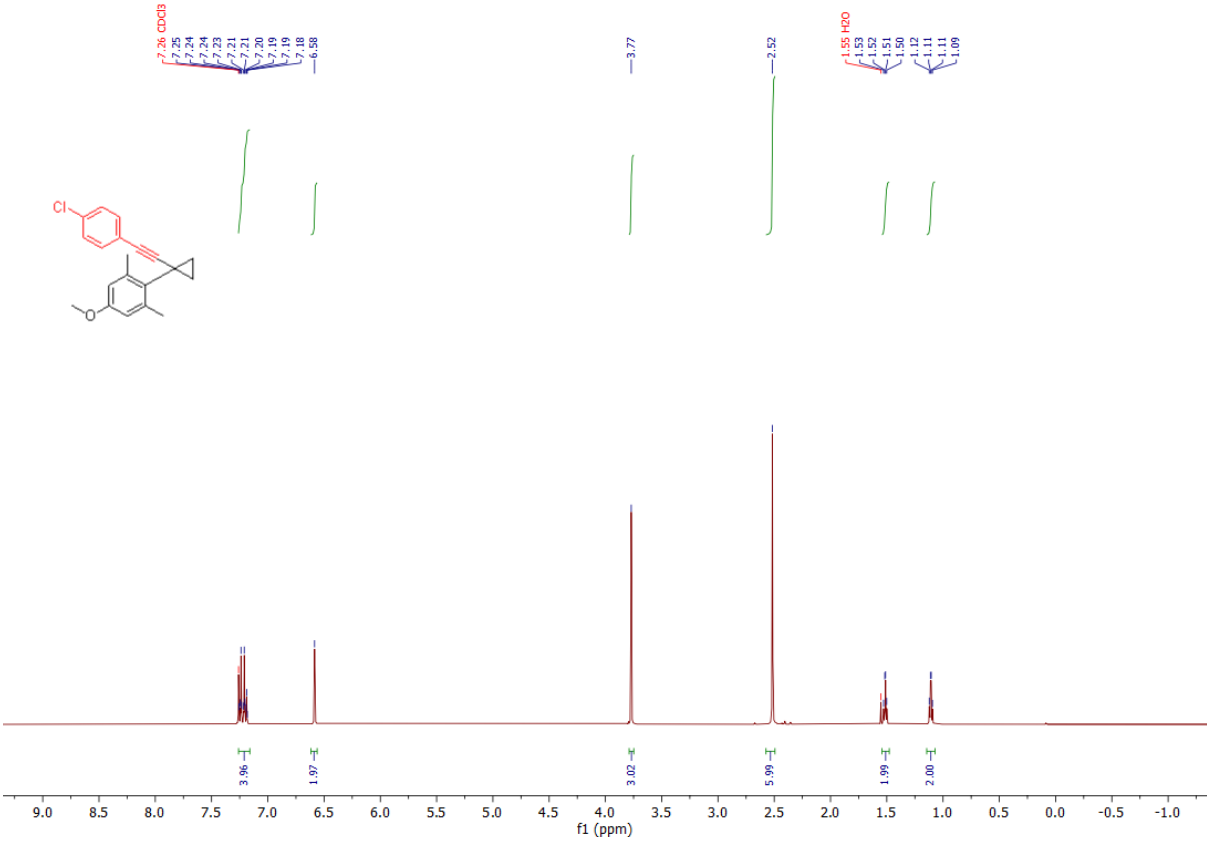

^{13}C NMR (101 MHz, $CDCl_3$) (4f)

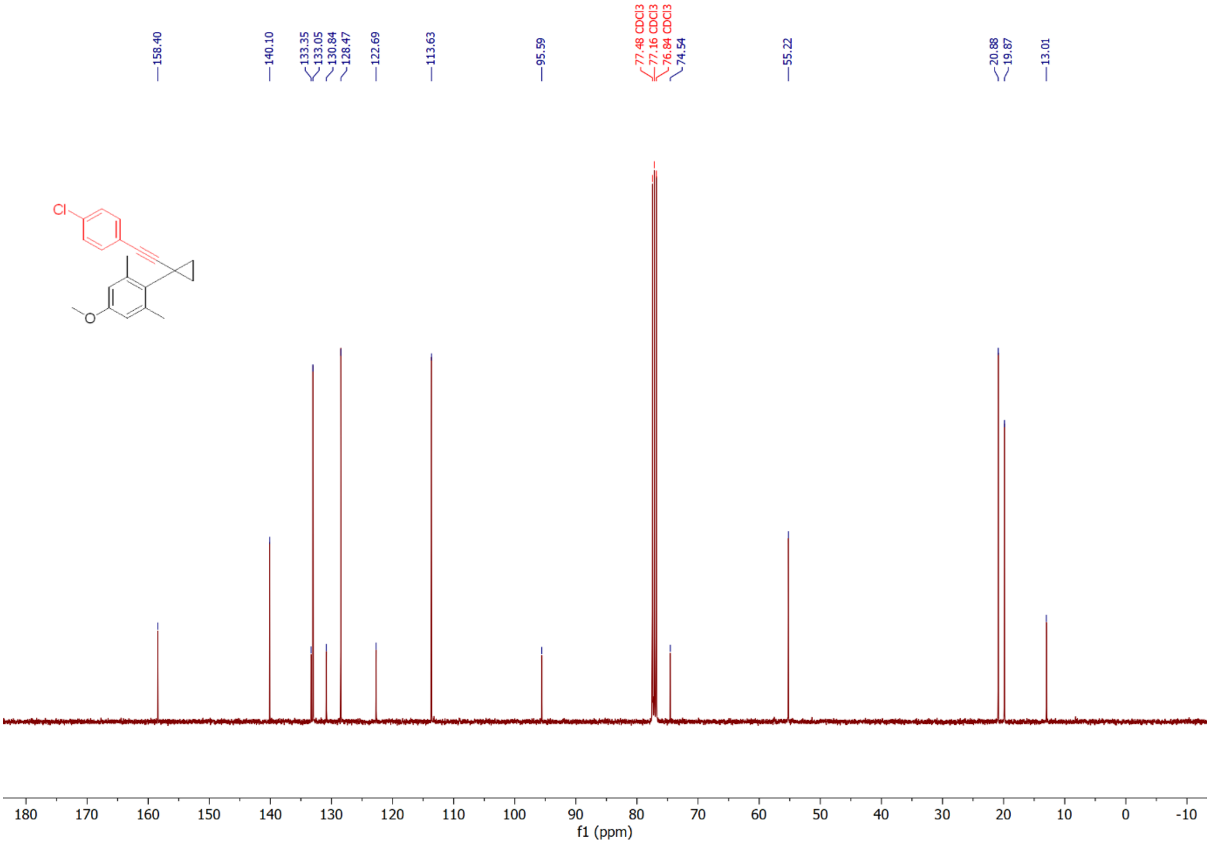

^{1}H NMR (400 MHz, $CDCl_3$) (4g)

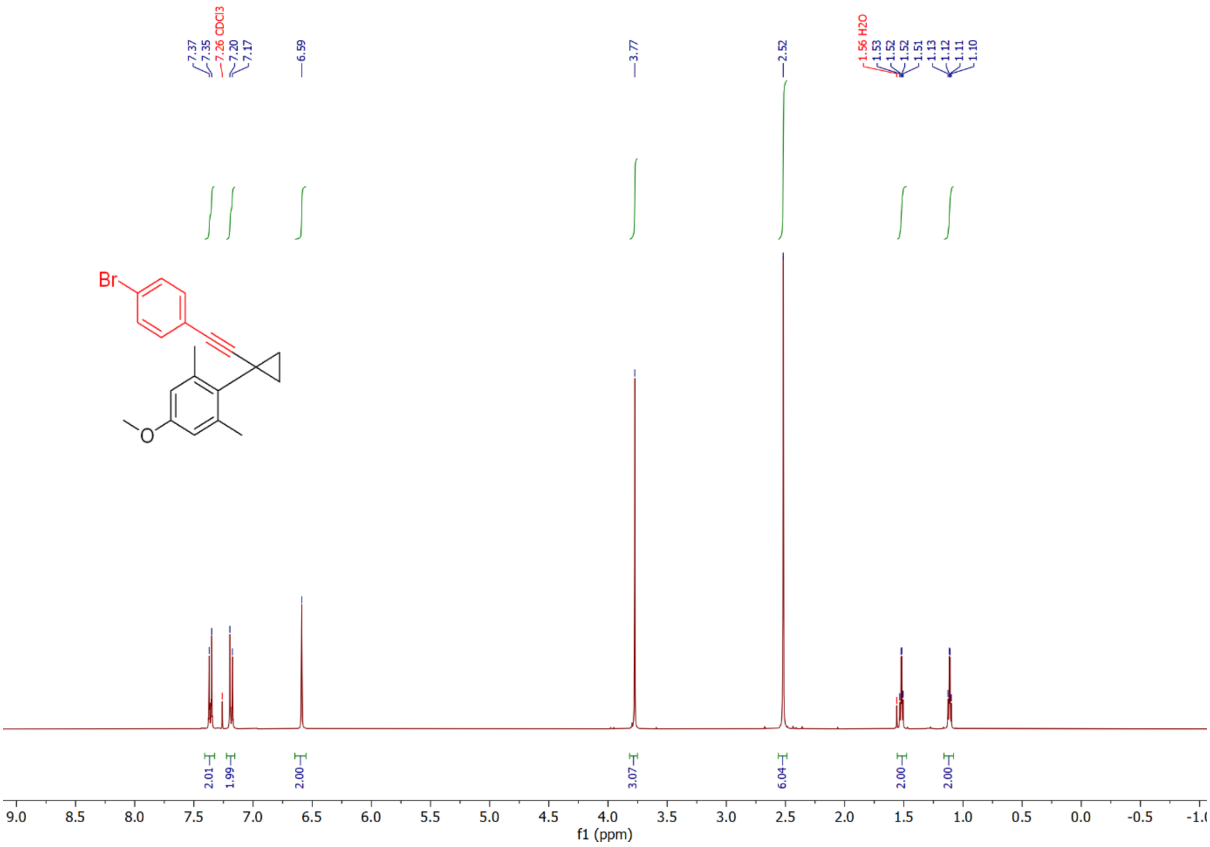

^{13}C NMR (101 MHz, $CDCl_3$) (4g)

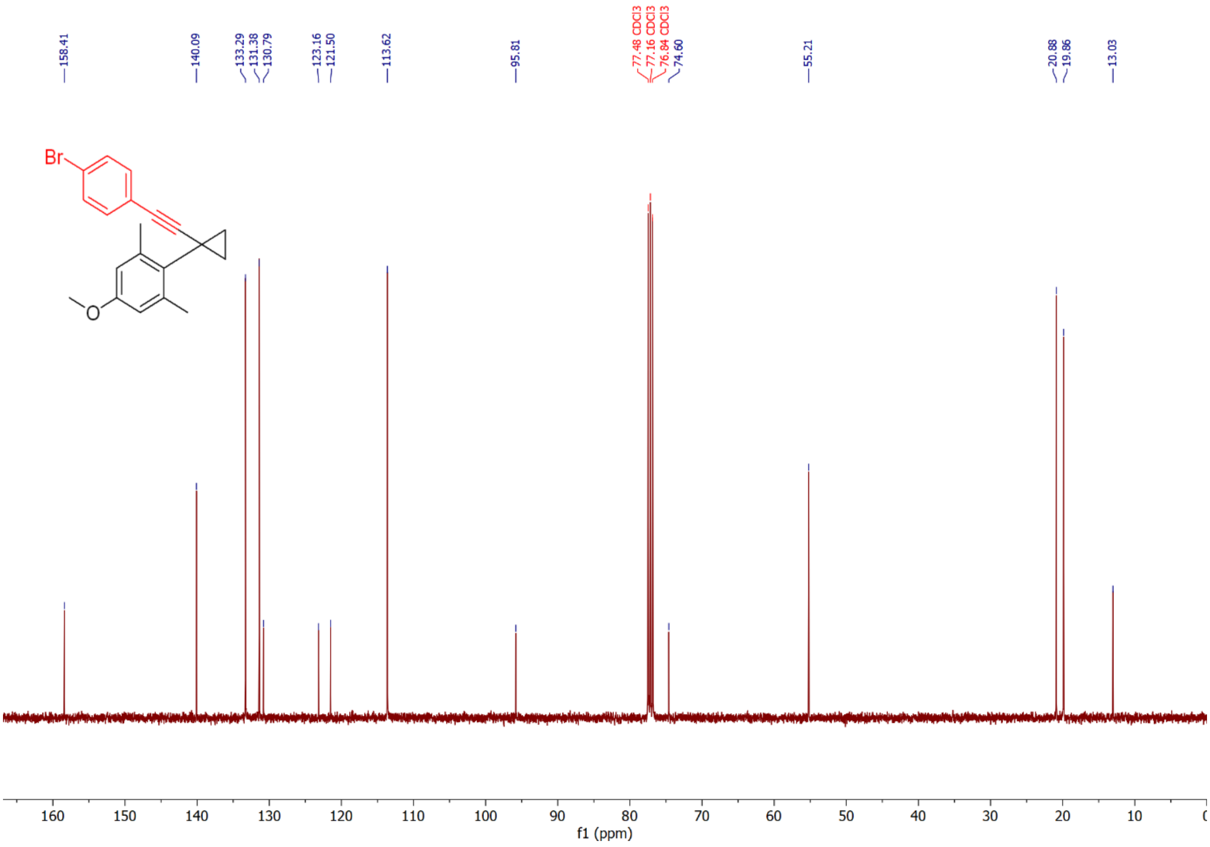

^{1}H NMR (400 MHz, $CDCl_3$) (4h)

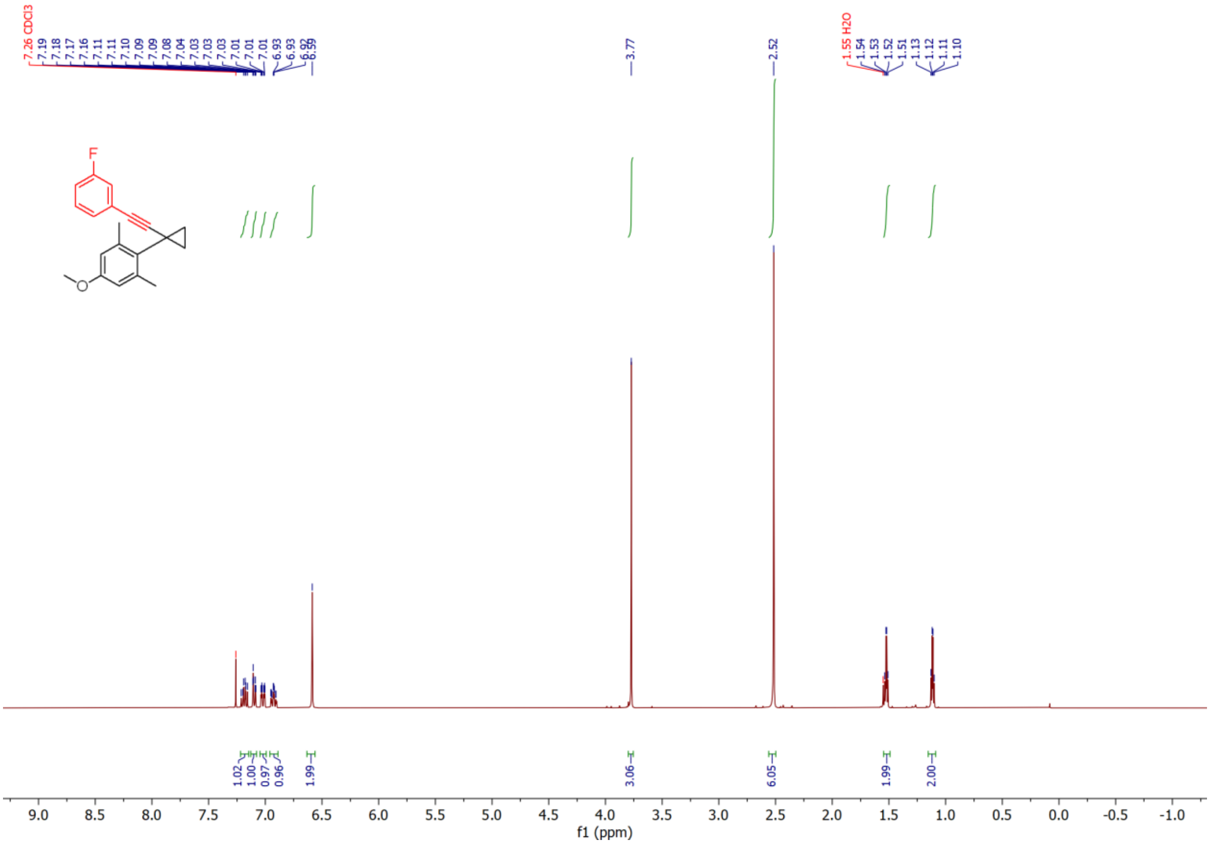

^{13}C NMR (101 MHz, $CDCl_3$) (4h)

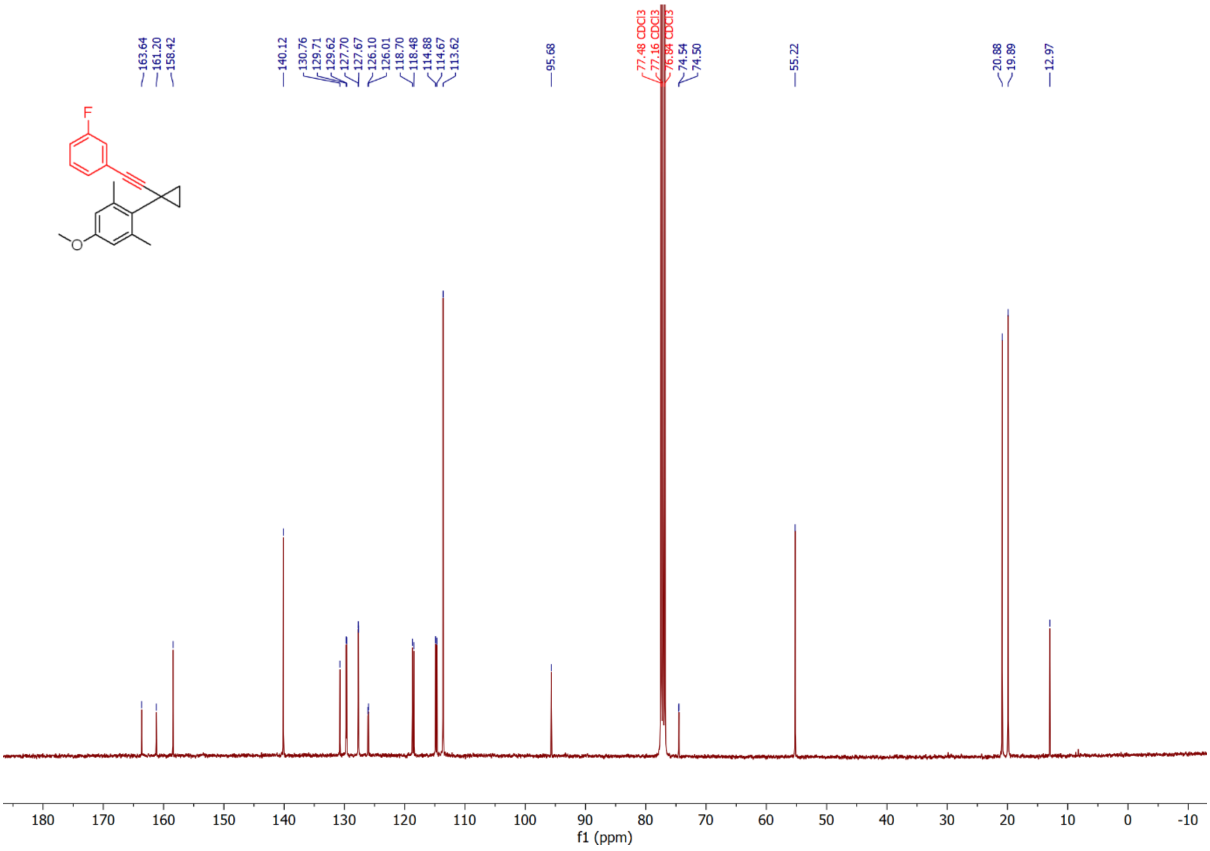

^{19}F NMR (377 MHz, $CDCl_3$) (4h)

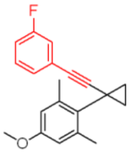

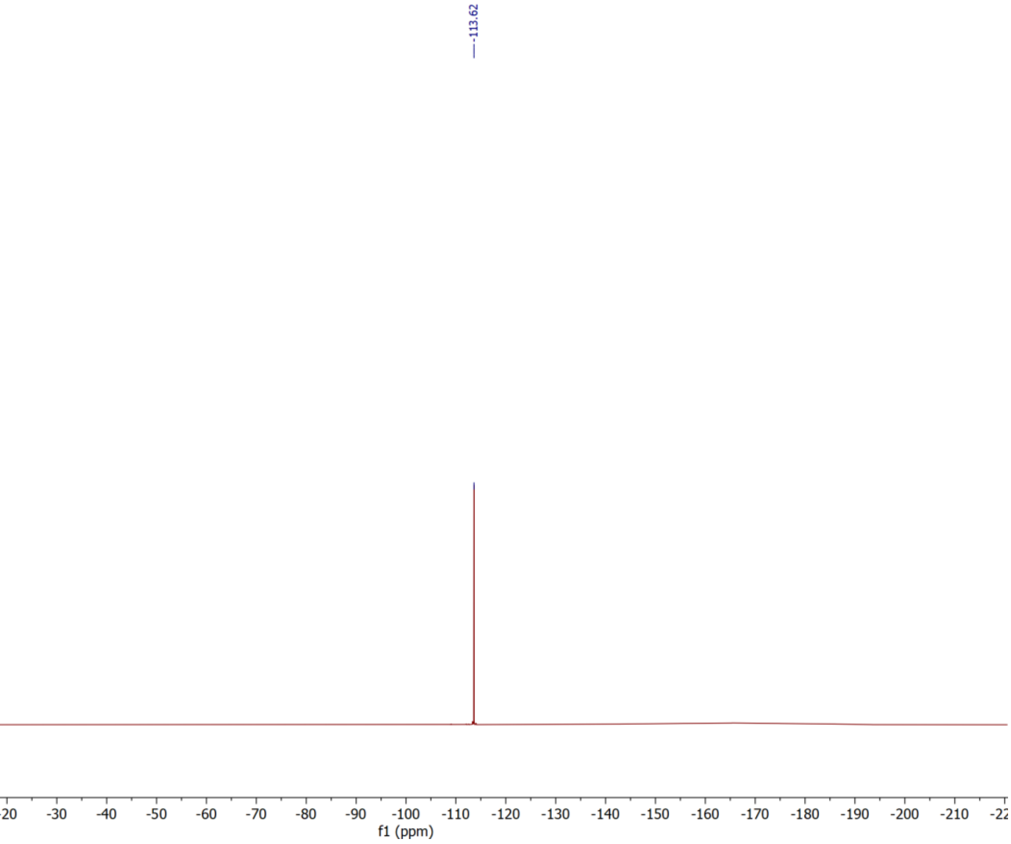

^{1}H NMR (400 MHz, $CDCl_3$) (4i)

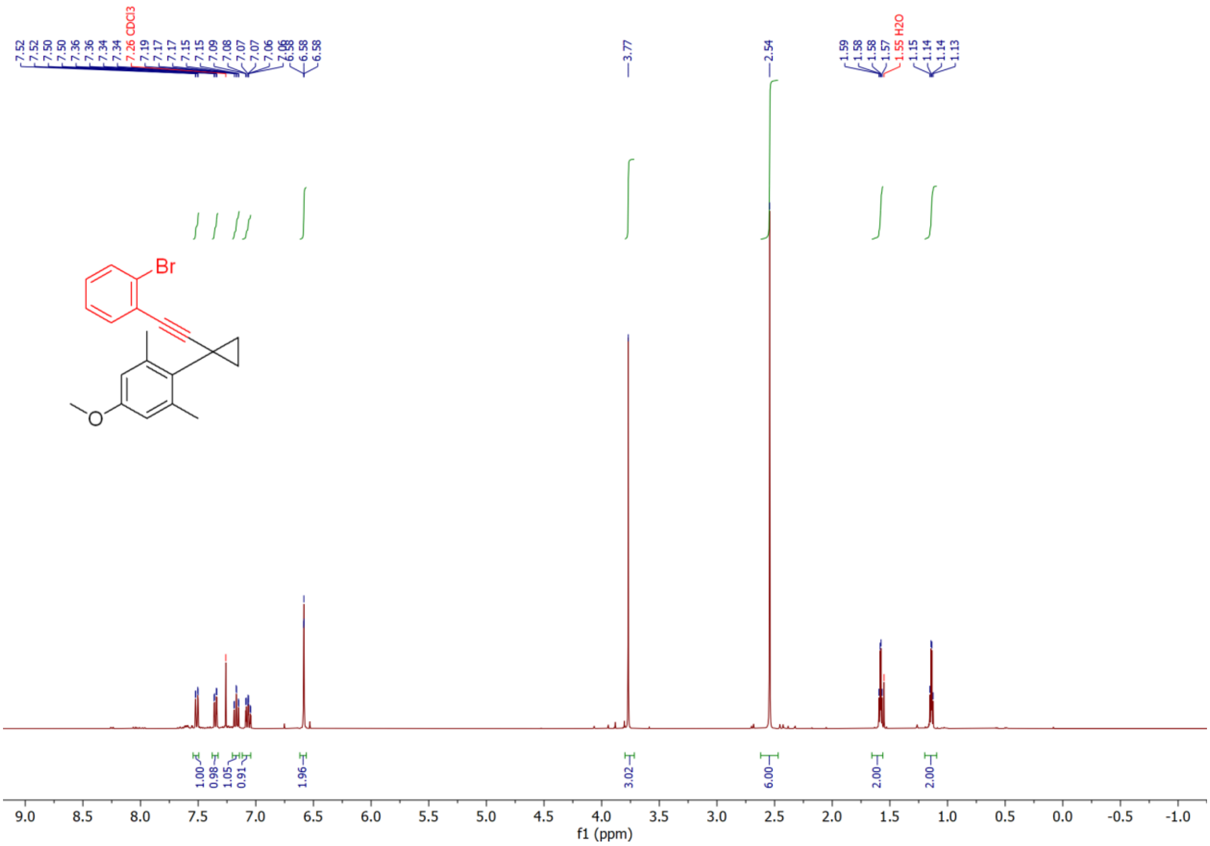

^{13}C NMR (101 MHz, $CDCl_3$) (4i)

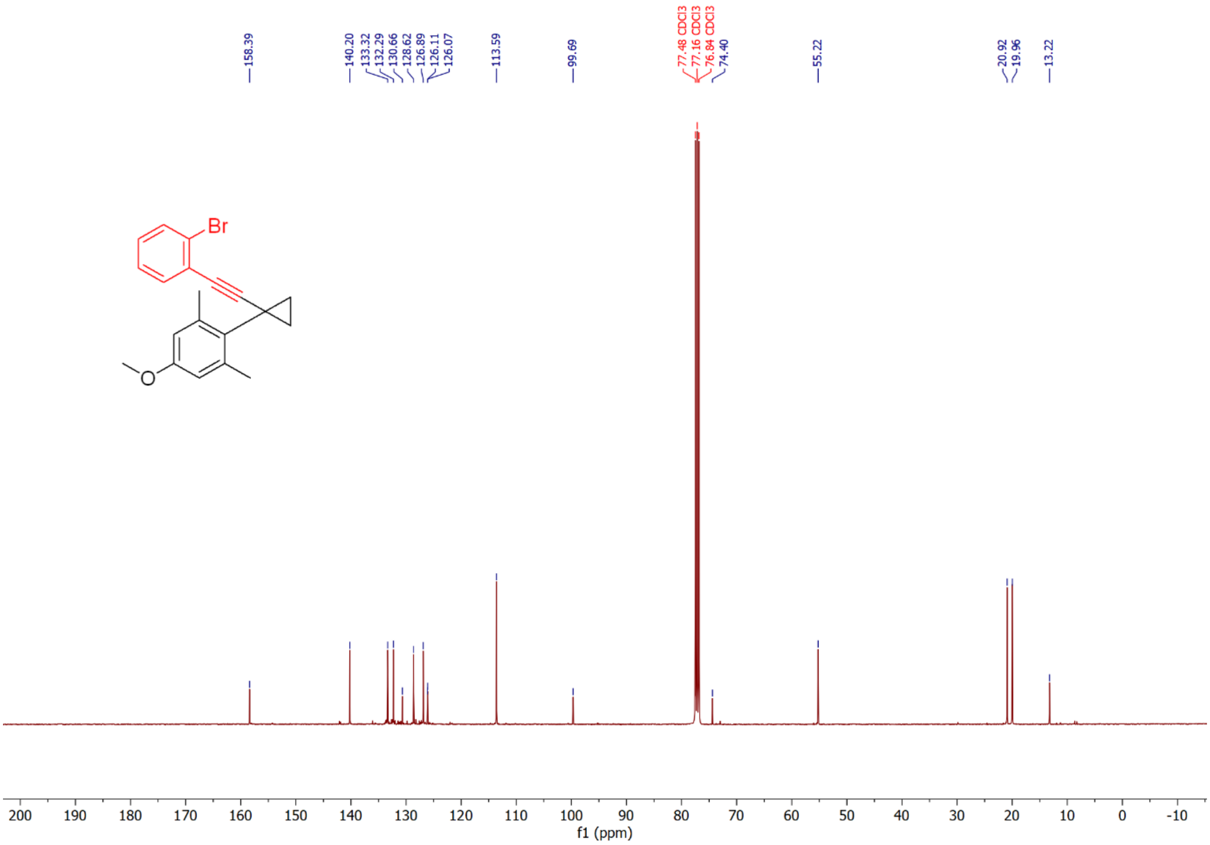

^{1}H NMR (400 MHz, $CDCl_3$) (4j)

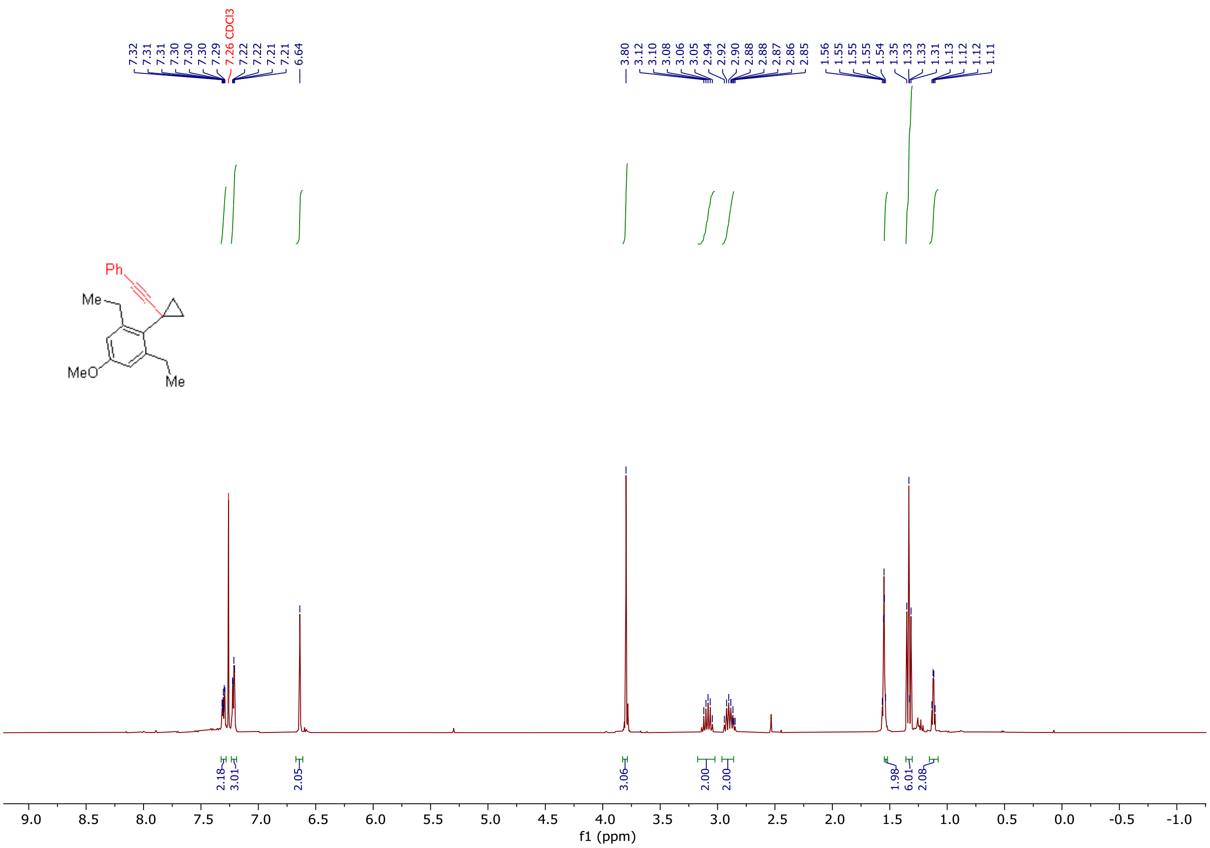

^{13}C NMR (101 MHz, $CDCl_3$) (4j)

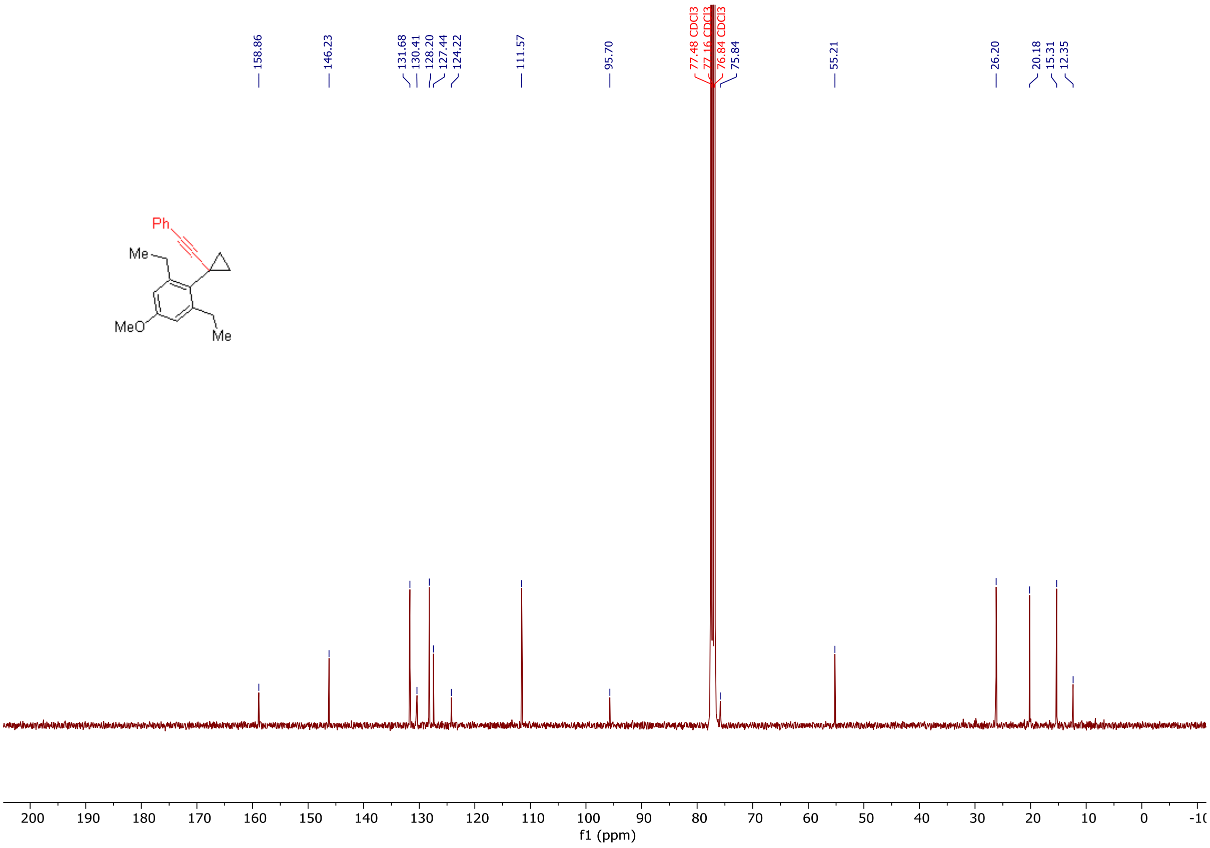

^{1}H NMR (400 MHz, $CDCl_3$) (4k)

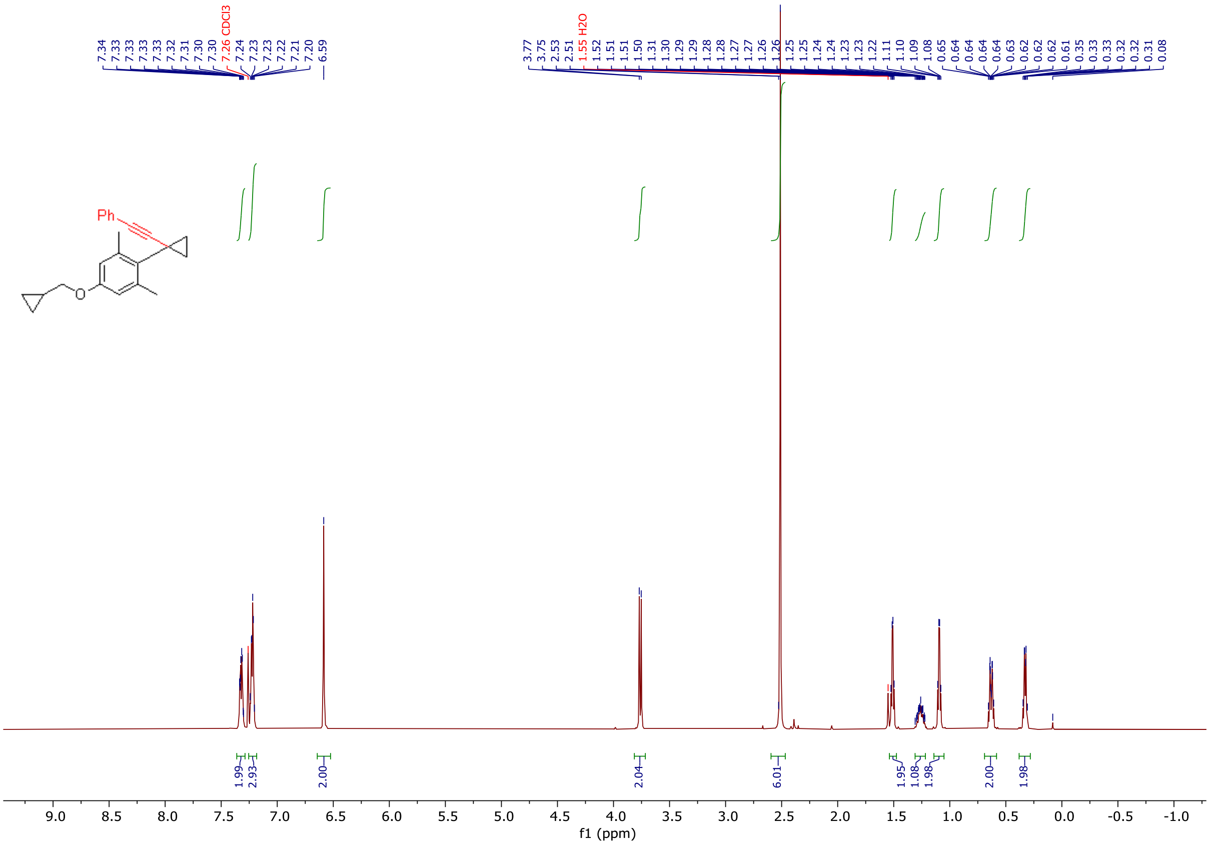

^{13}C NMR (101 MHz, $CDCl_3$) (4k)

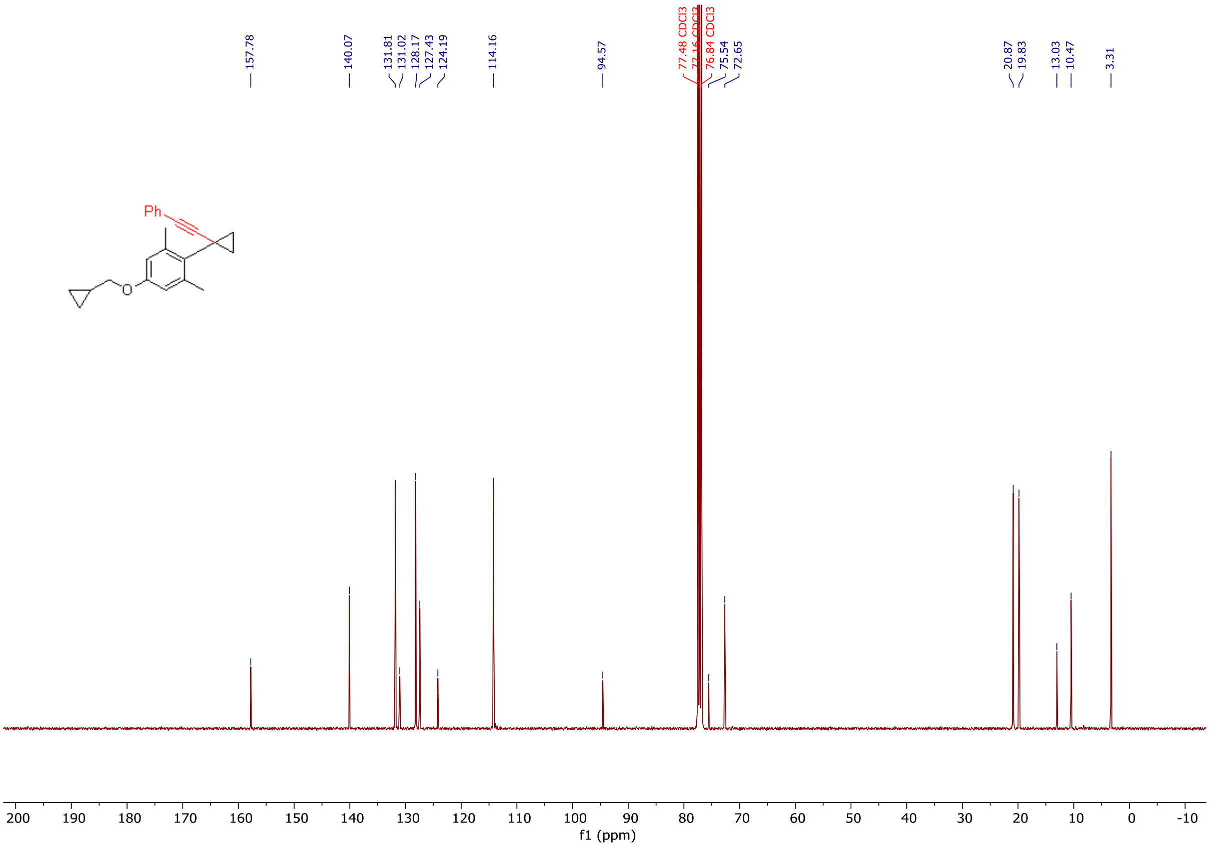

^{1}H NMR (400 MHz, $CDCl_3$) (4l): Blue : Major diastereomer, Organge: minor diastereomer

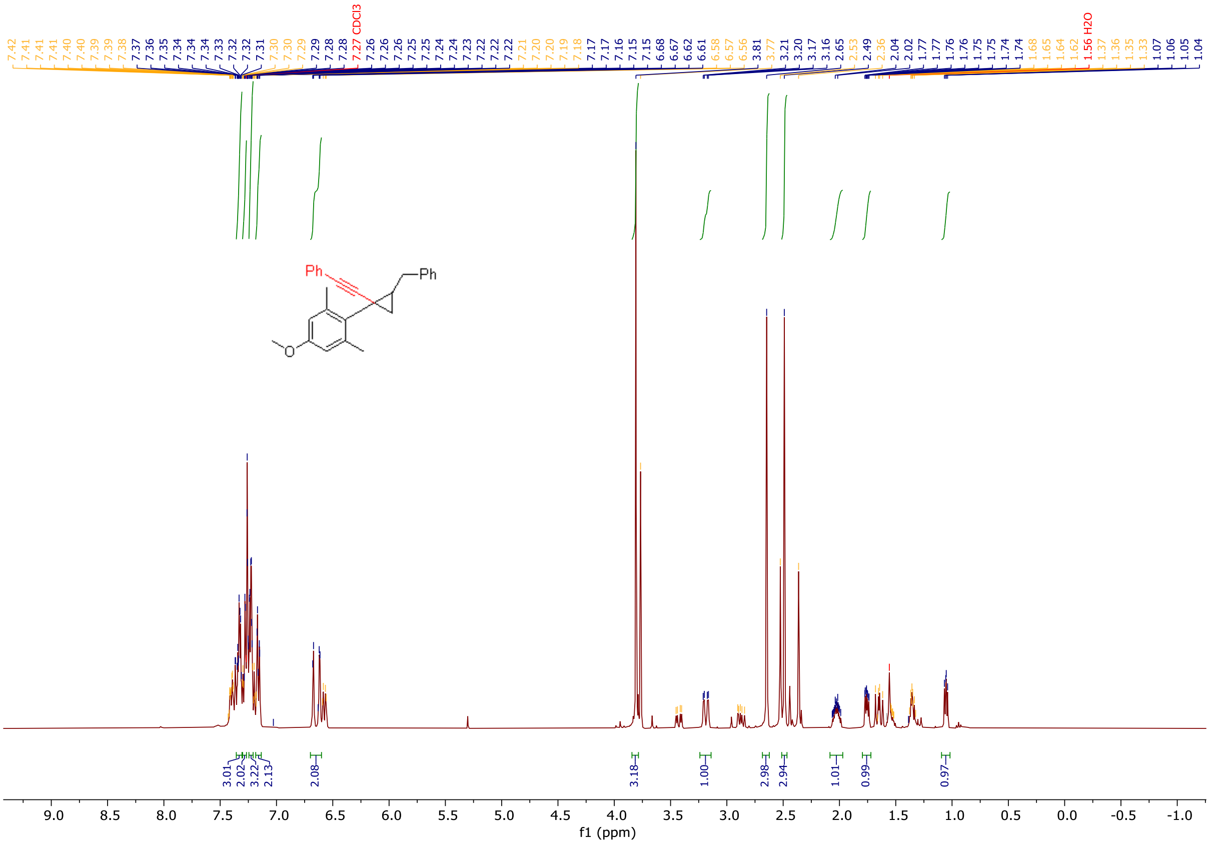

^{13}C NMR (101 MHz, $CDCl_3$) (4l)

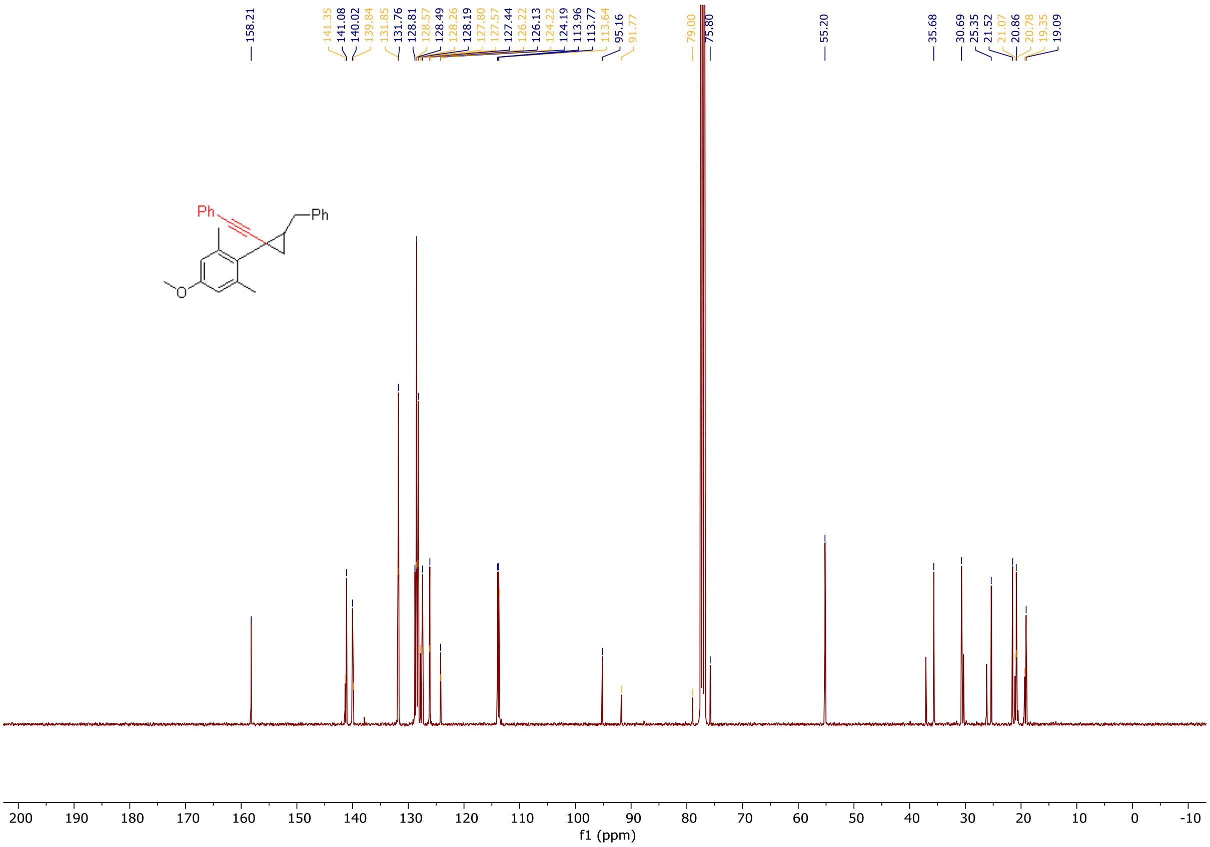

^{1}H NMR (400 MHz, $CDCl_3$) (4m) Blue : Major diastereomer, Organge: minor diastereomer

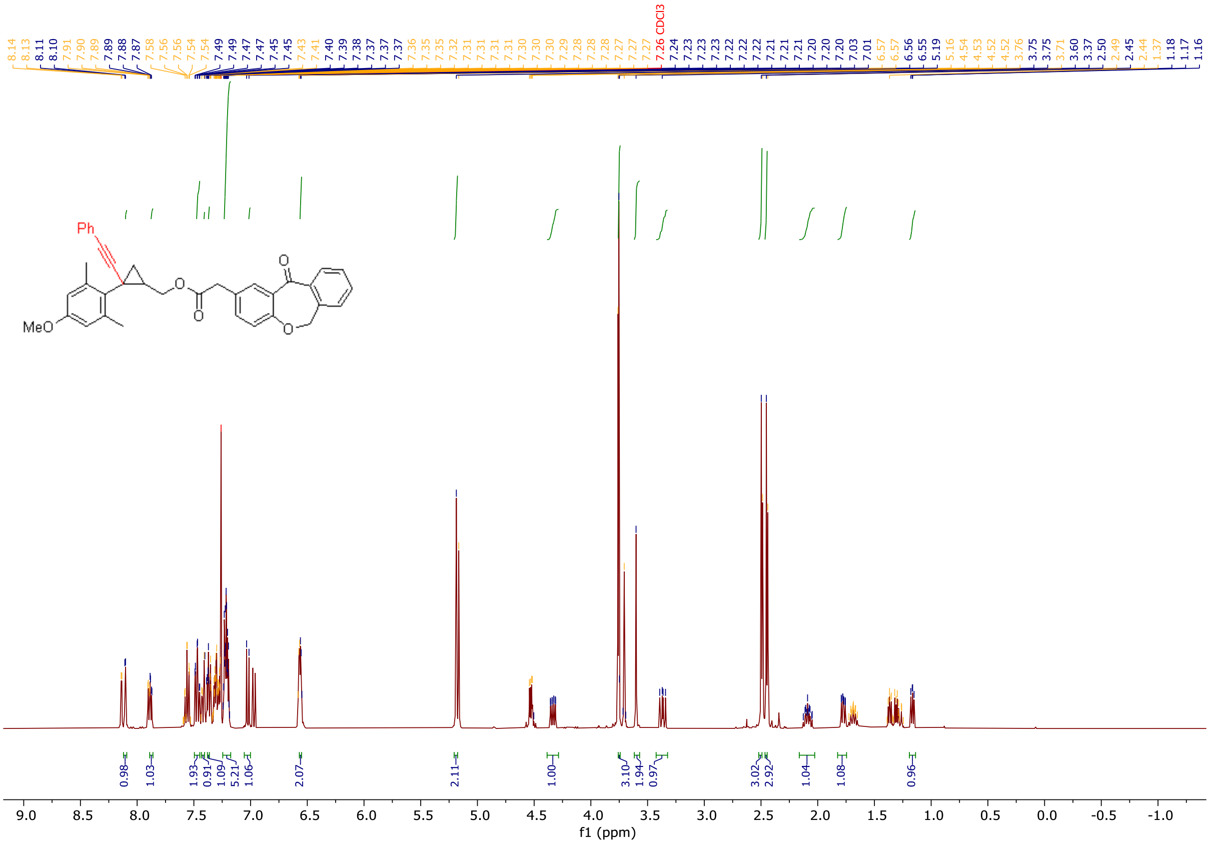

^{13}C NMR (101 MHz, $CDCl_3$) (4m)

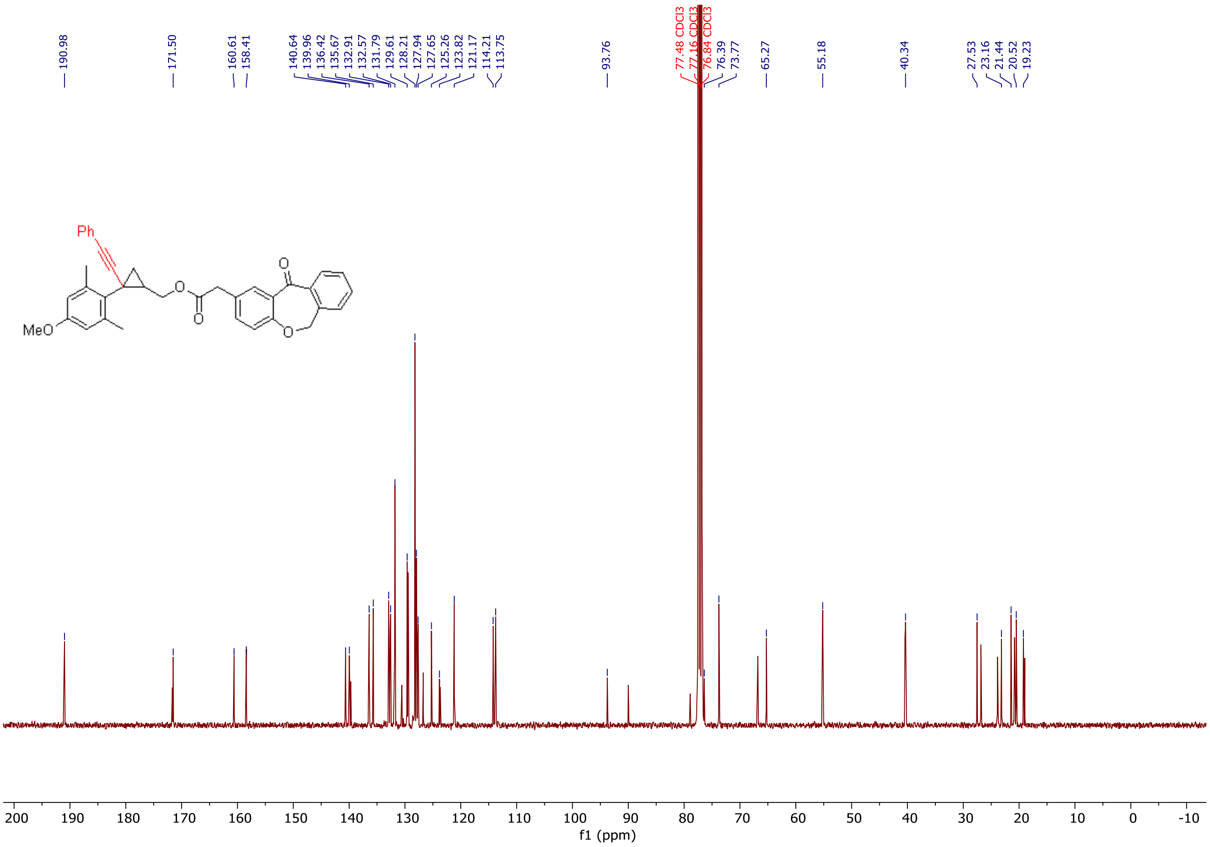

^{1}H NMR (400 MHz, $CDCl_3$) (4n)

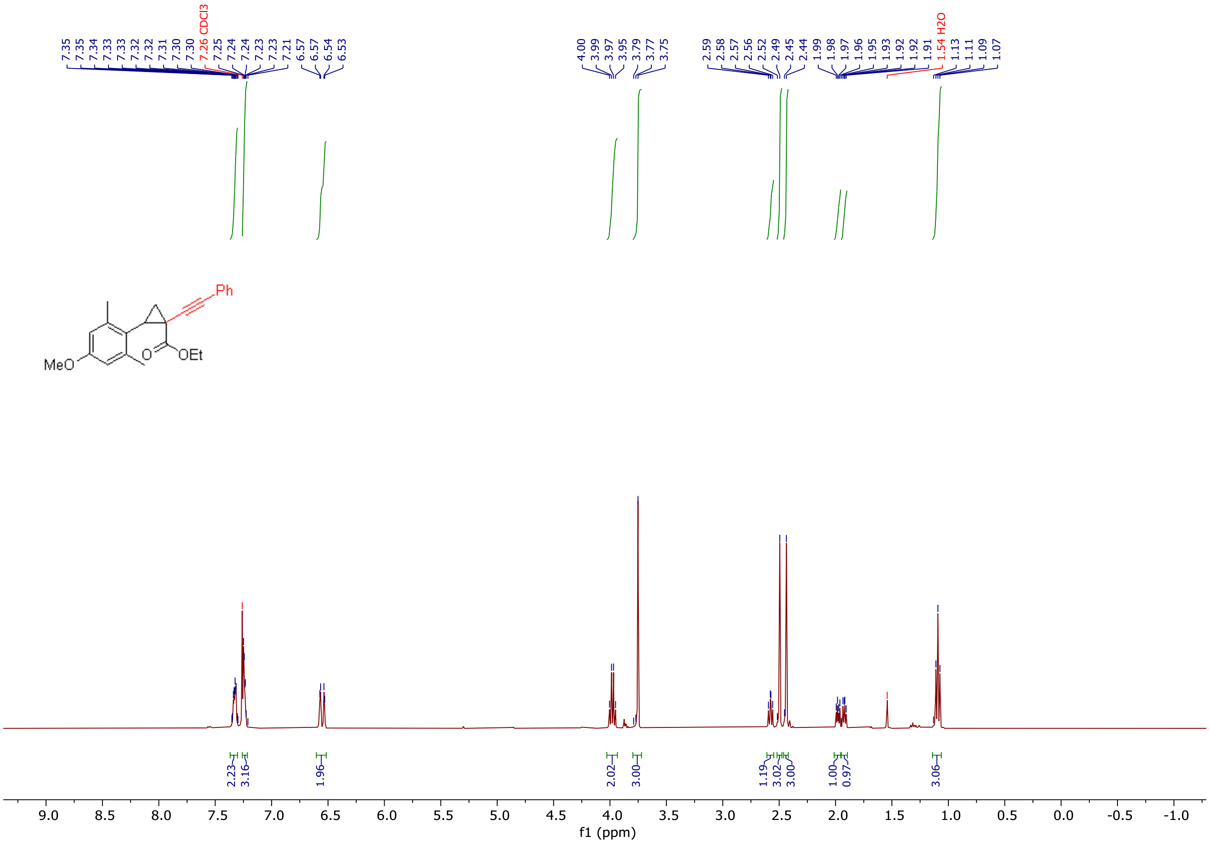

^{13}C NMR (101 MHz, $CDCl_3$) (4n)

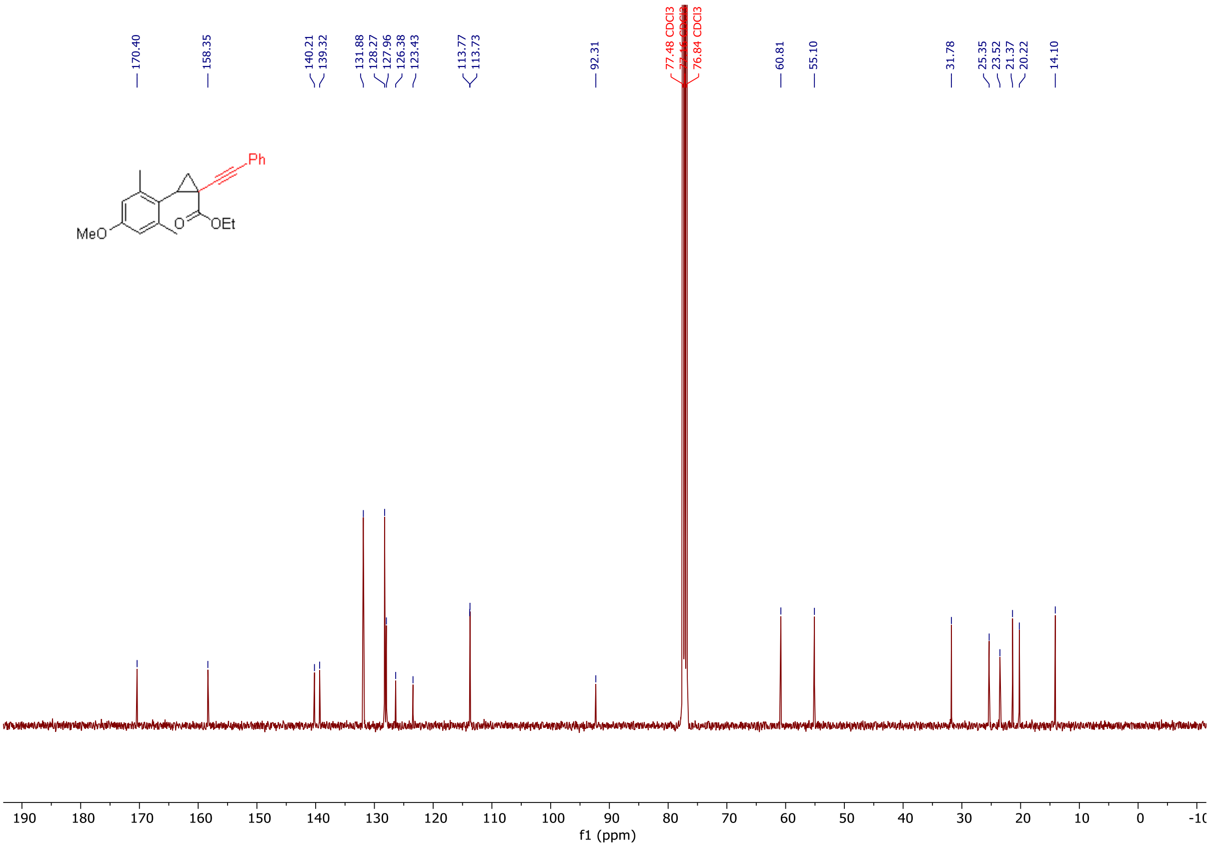

^{1}H NMR (400 MHz, $CDCl_3$) (4o1) – 1st diastereomer

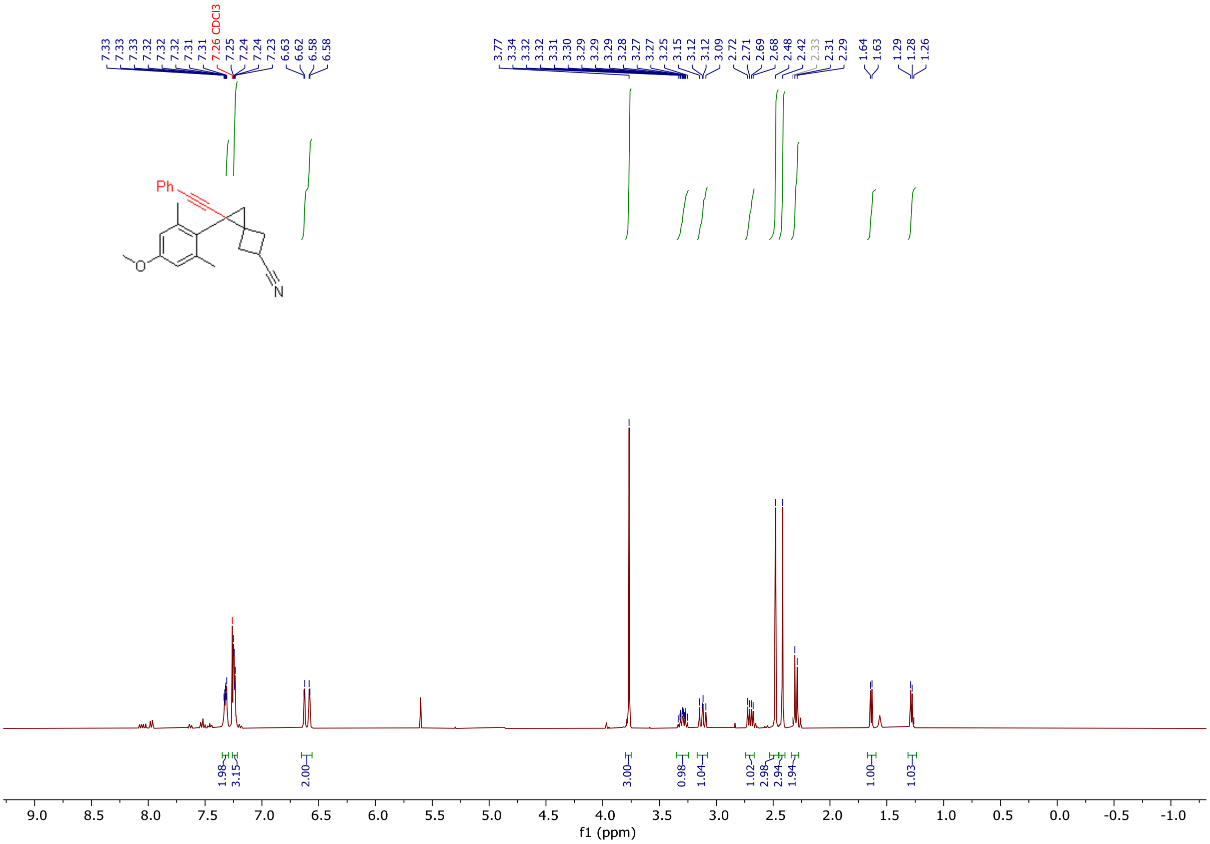

^{13}C NMR (101 MHz, $CDCl_3$) (4o1) – 1st diastereomer

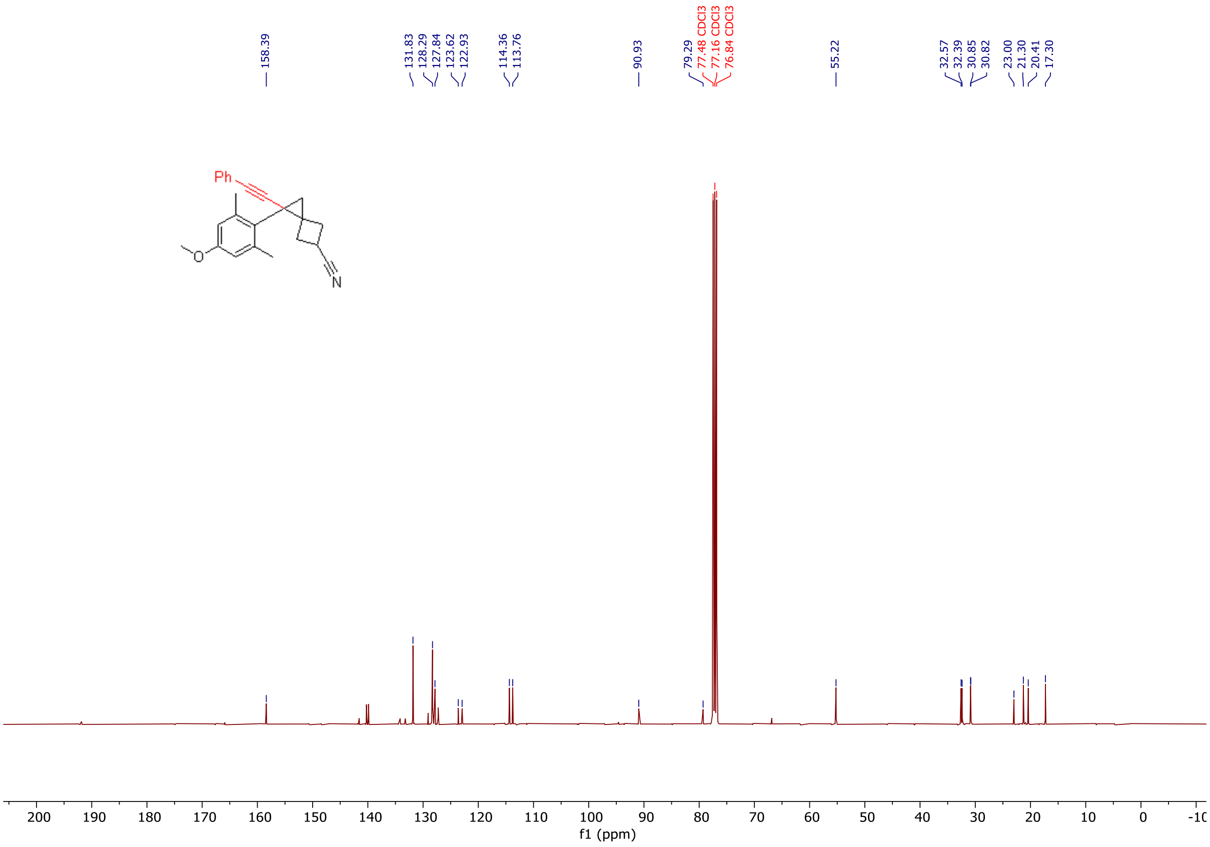

^{1}H NMR (400 MHz, $CDCl_3$) (4o2) – 2nd diastereomer

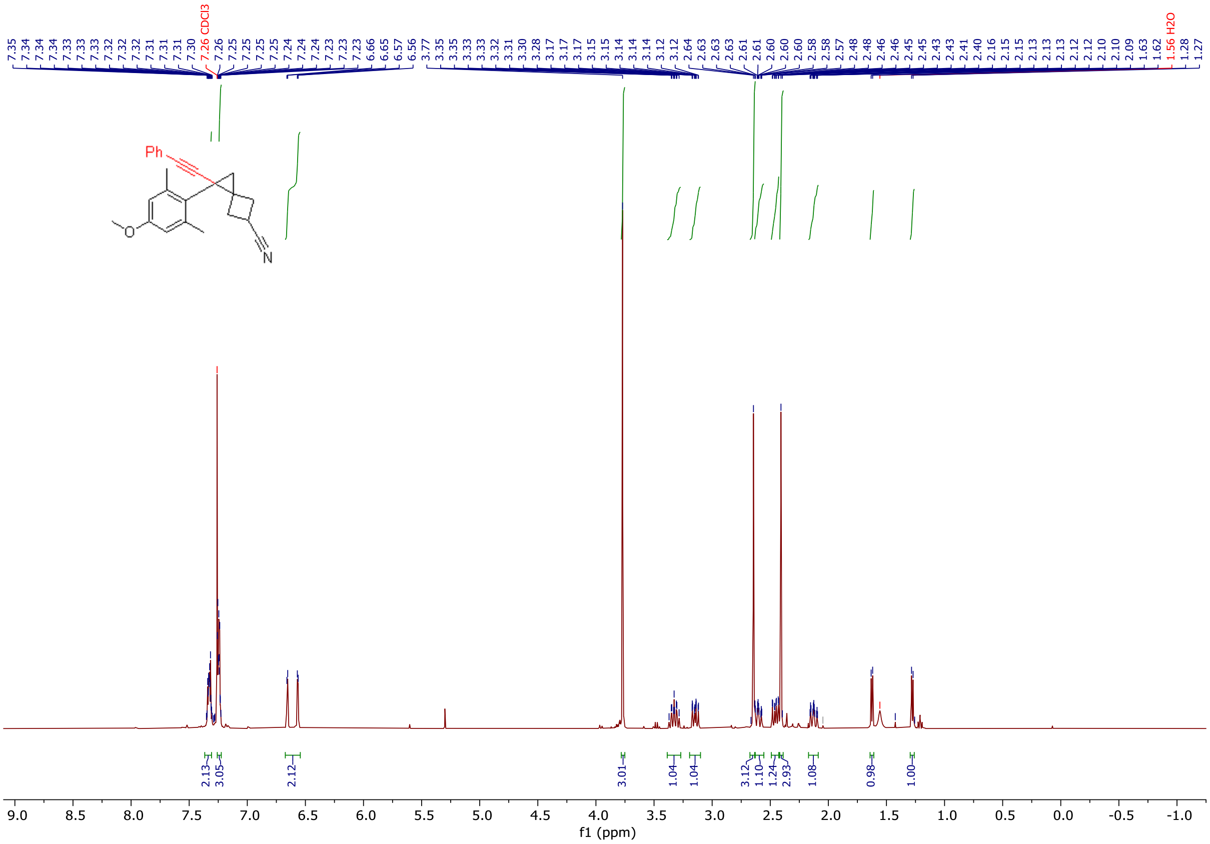

^{13}C NMR (101 MHz, $CDCl_3$) (4o2) – 2nd diastereomer

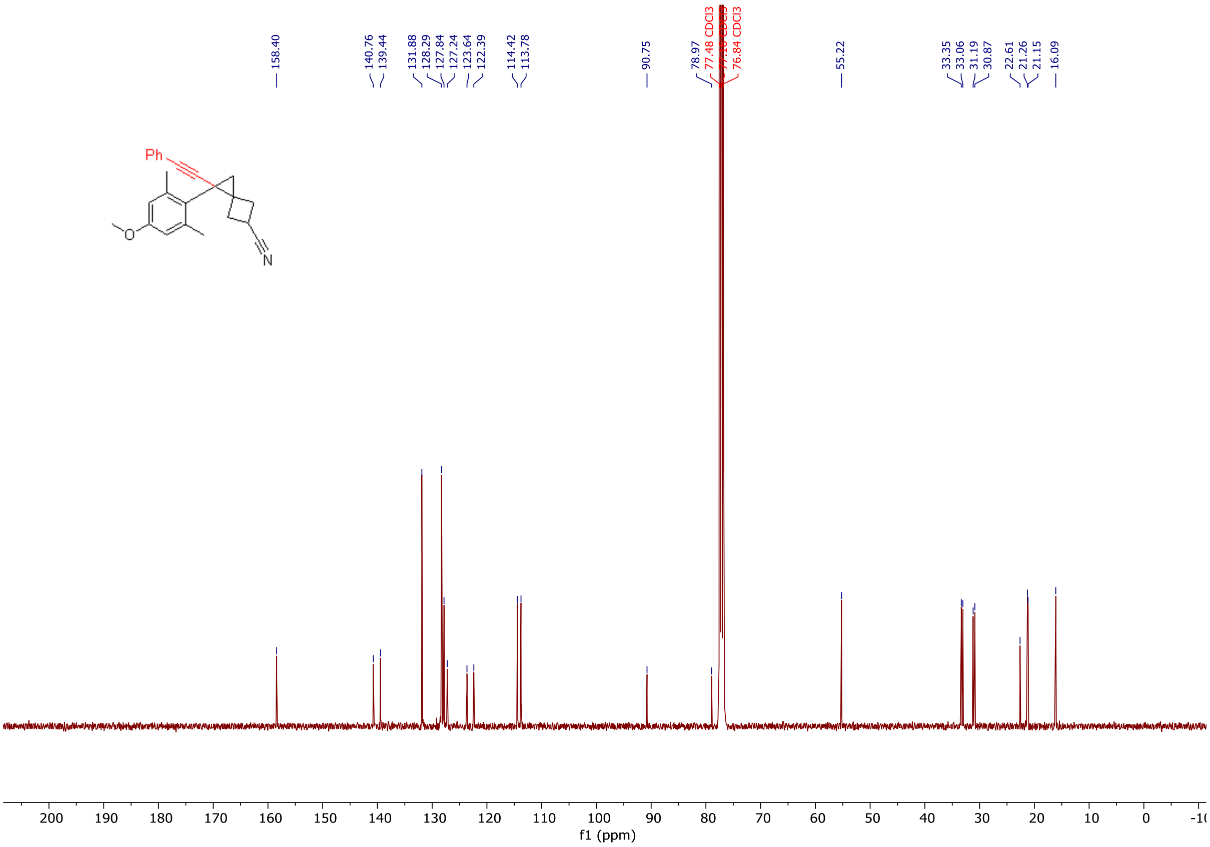

^{1}H NMR (400 MHz, $CDCl_3$) (4p)

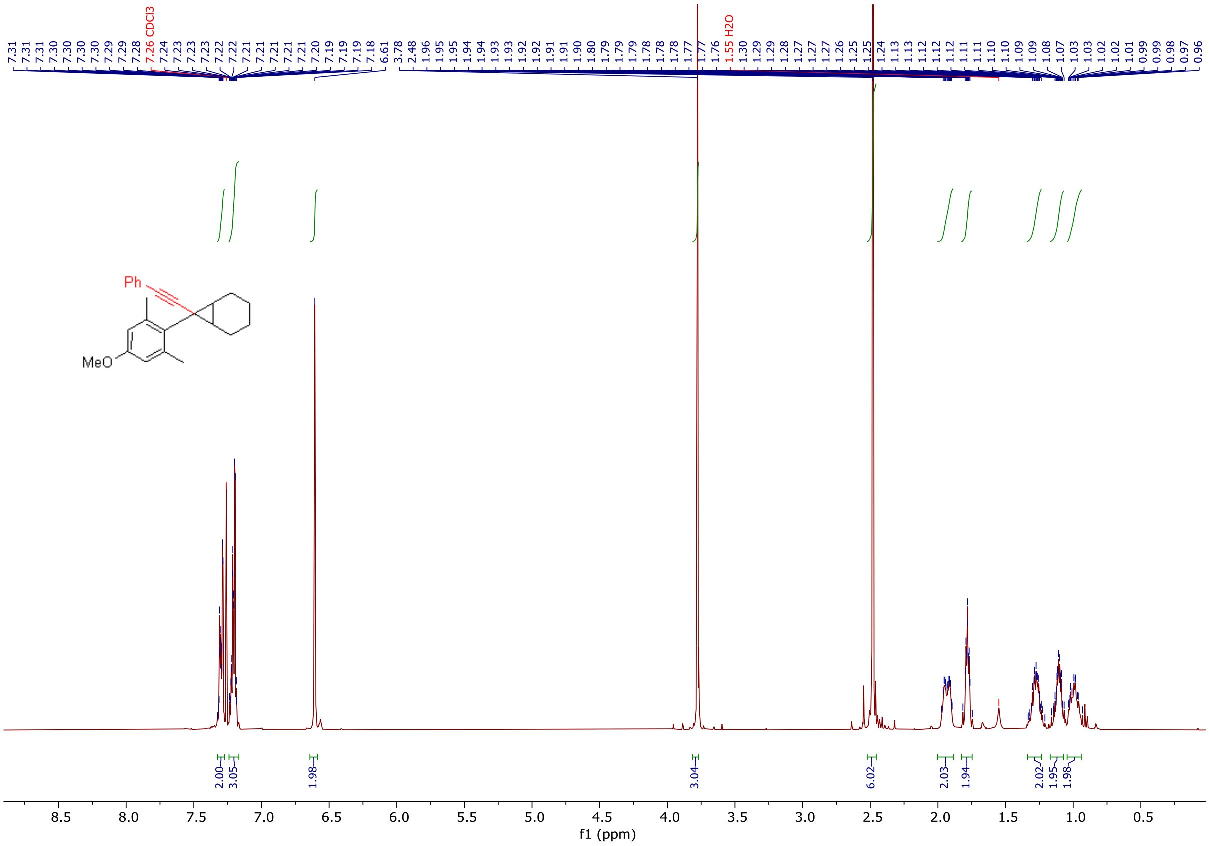

^{13}C NMR (101 MHz, $CDCl_3$) (4p)

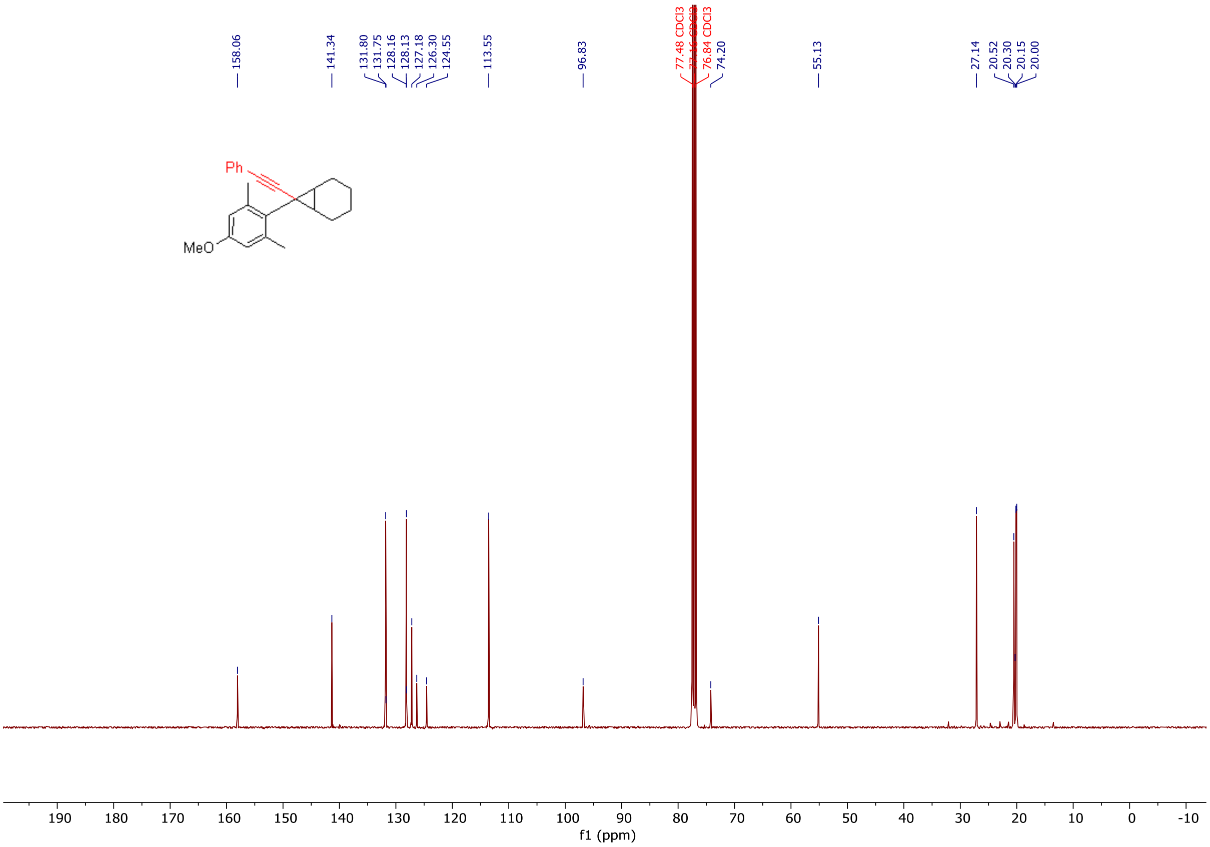

^{1}H NMR (400 MHz, $CDCl_3$) (4q1)

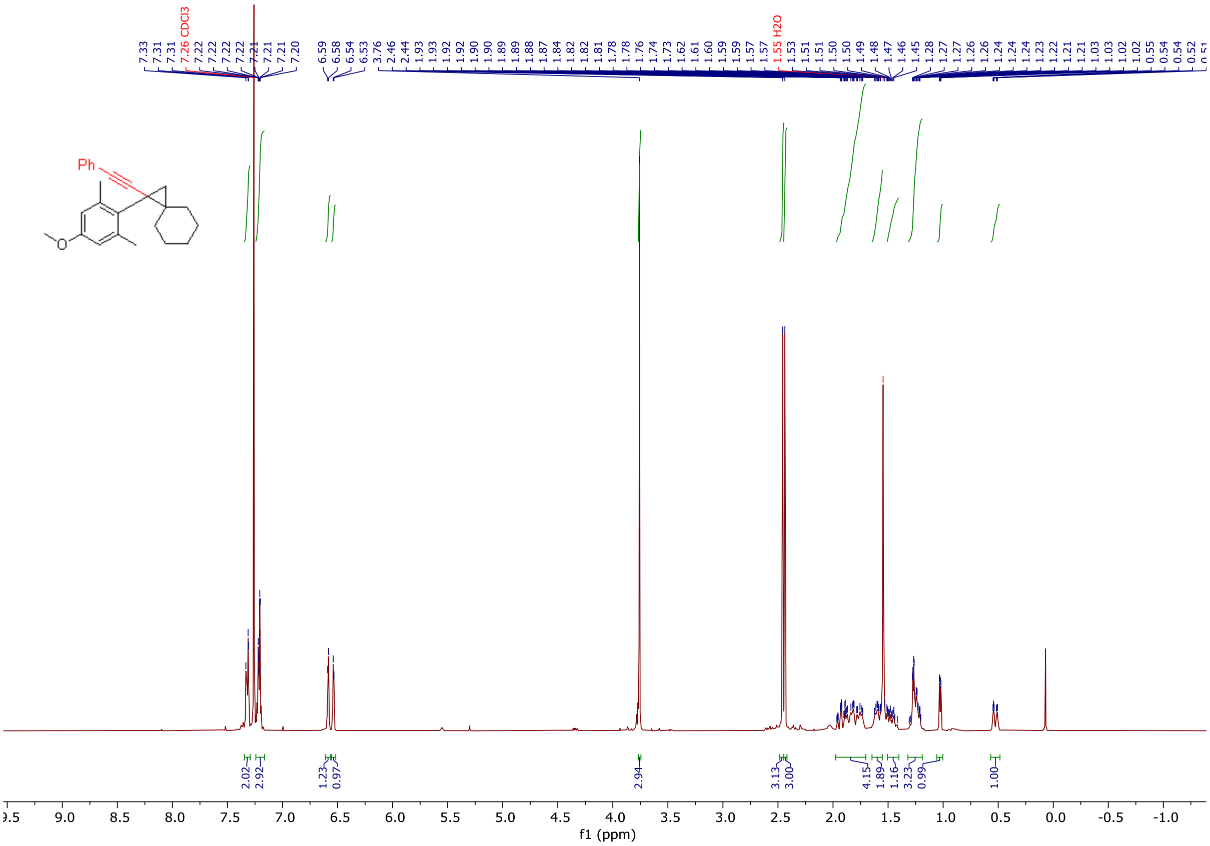

^{13}C NMR (101 MHz, $CDCl_3$) (4q1)

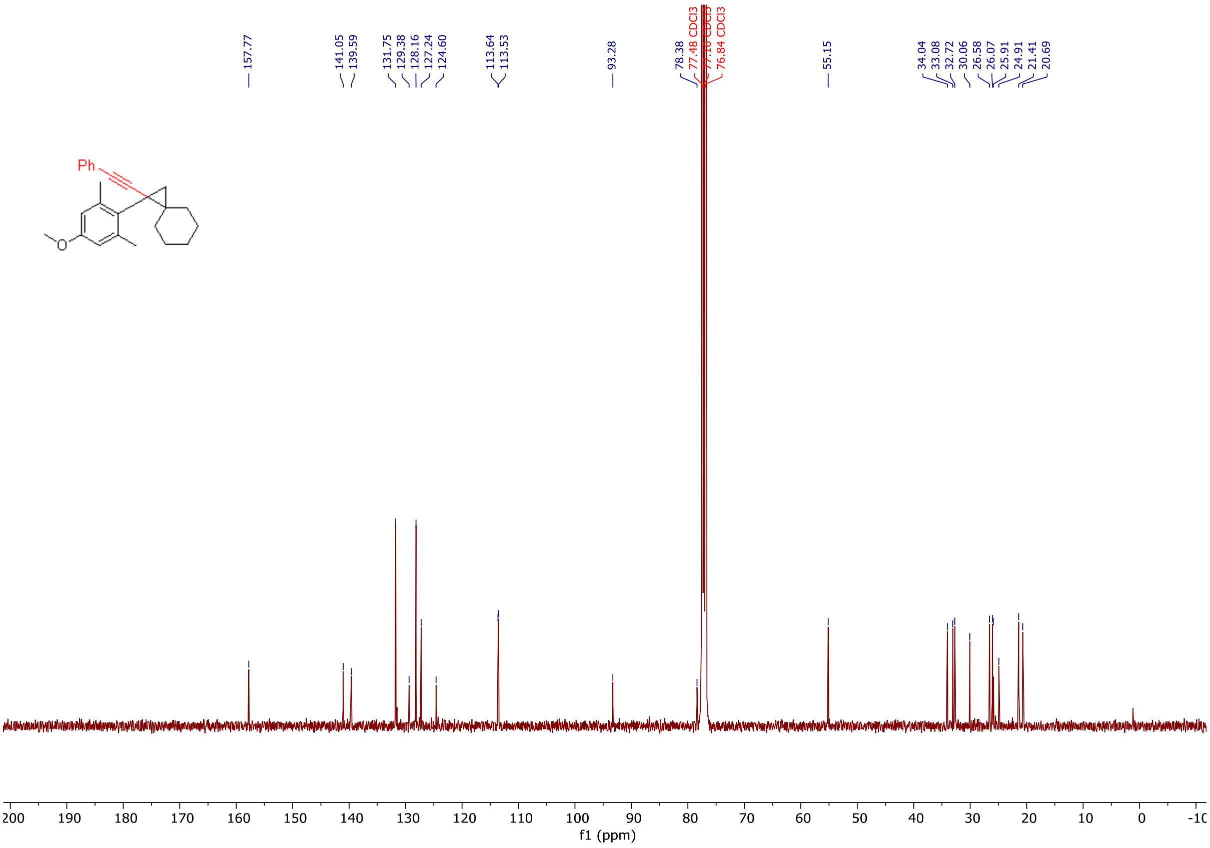

^{1}H NMR (400 MHz, $CDCl_3$) (4q2)

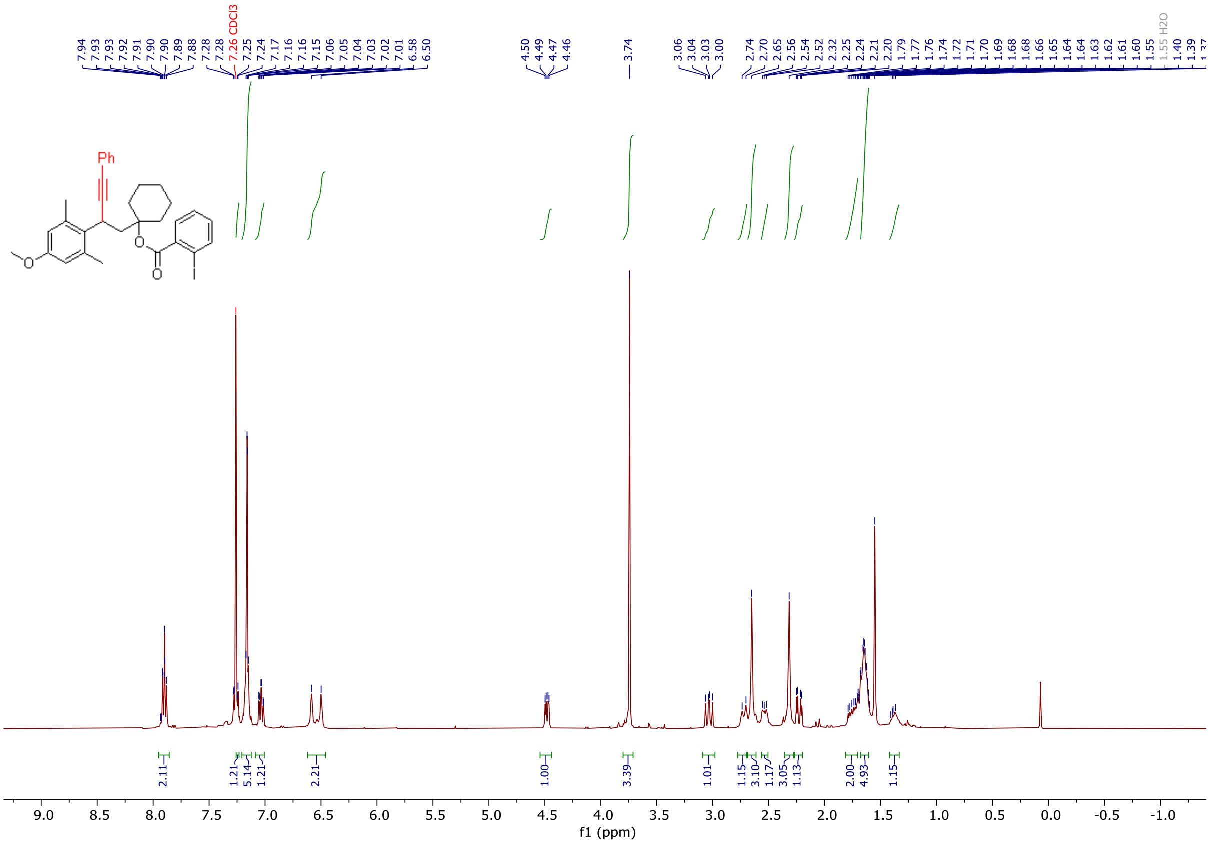

^{13}C NMR (101 MHz, $CDCl_3$) (4q2)

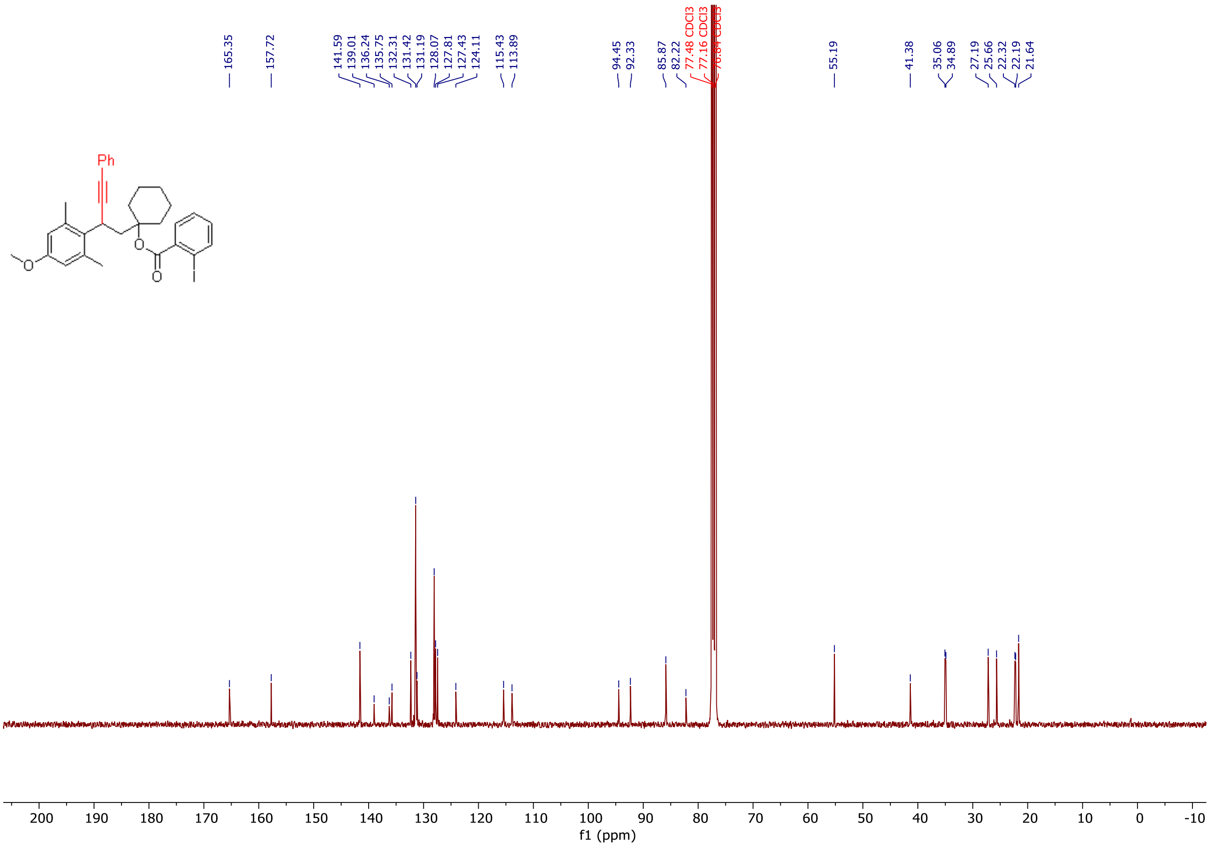

^{1}H NMR (400 MHz, $CDCl_3$) (4r)

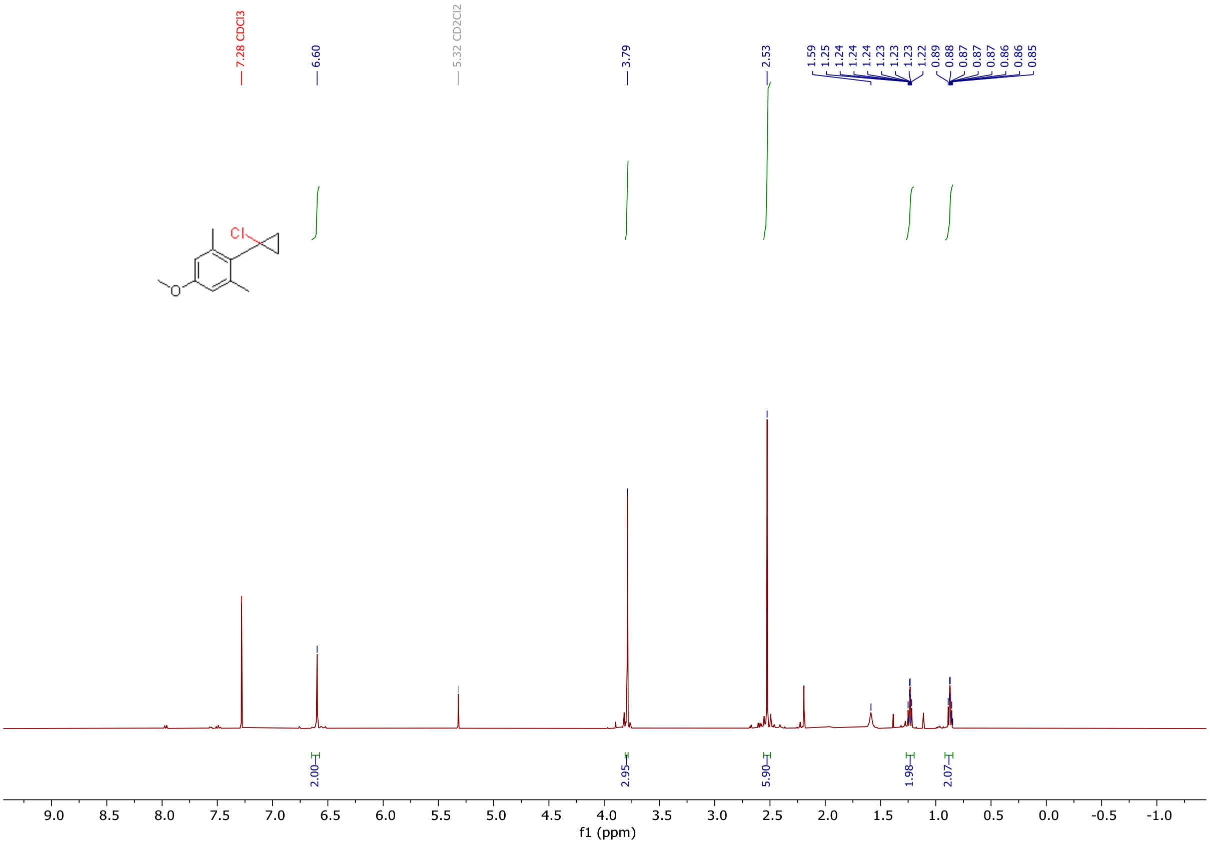

^{13}C NMR (101 MHz, $CDCl_3$) (4r)

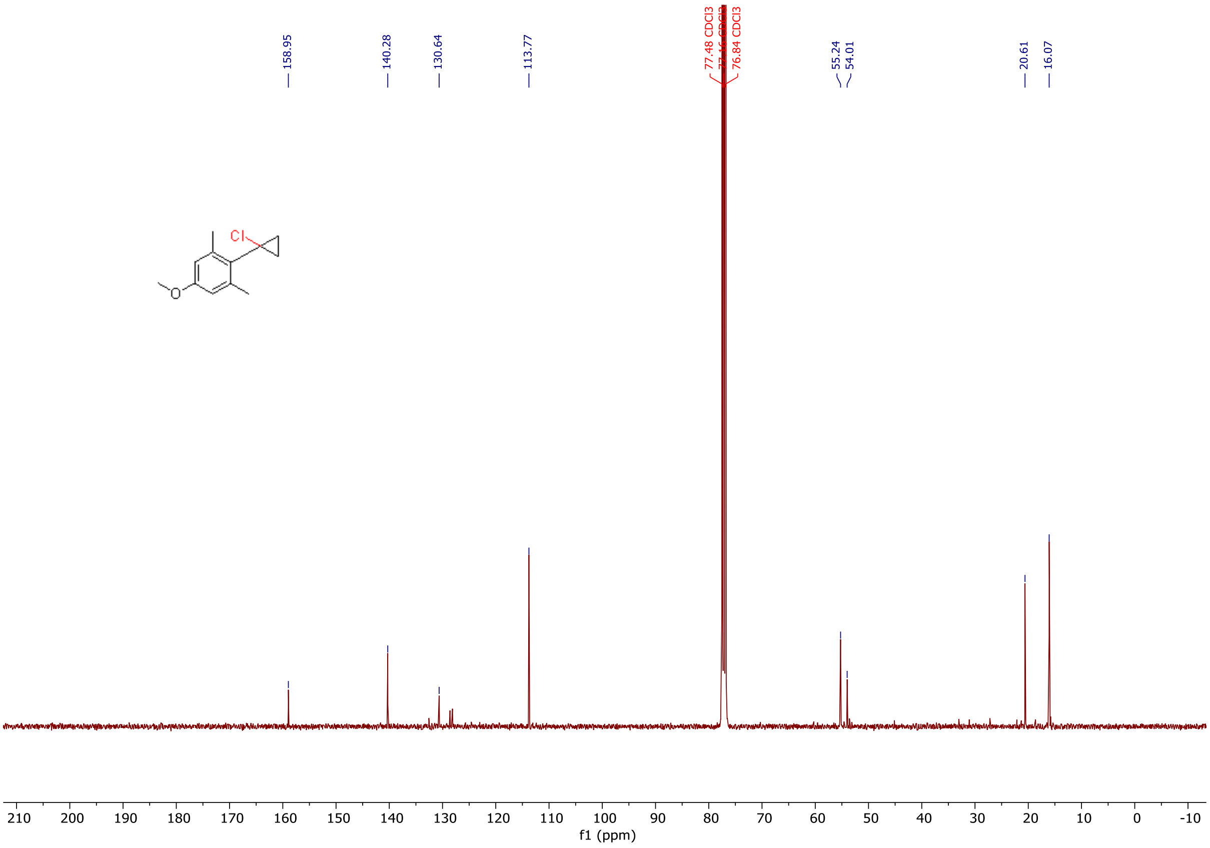

^{1}H NMR (400 MHz, $CDCl_3$) (4s1)

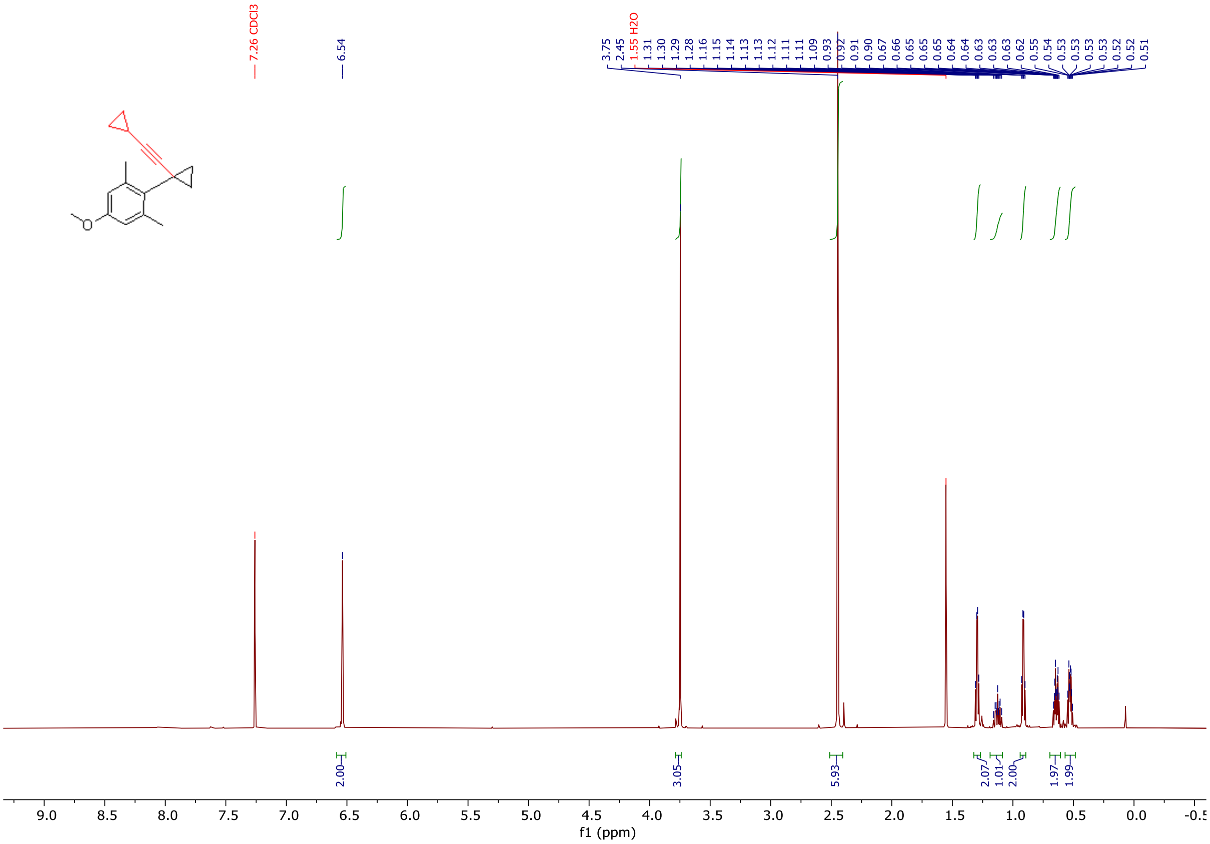

^{13}C NMR (101 MHz, $CDCl_3$) (4s1)

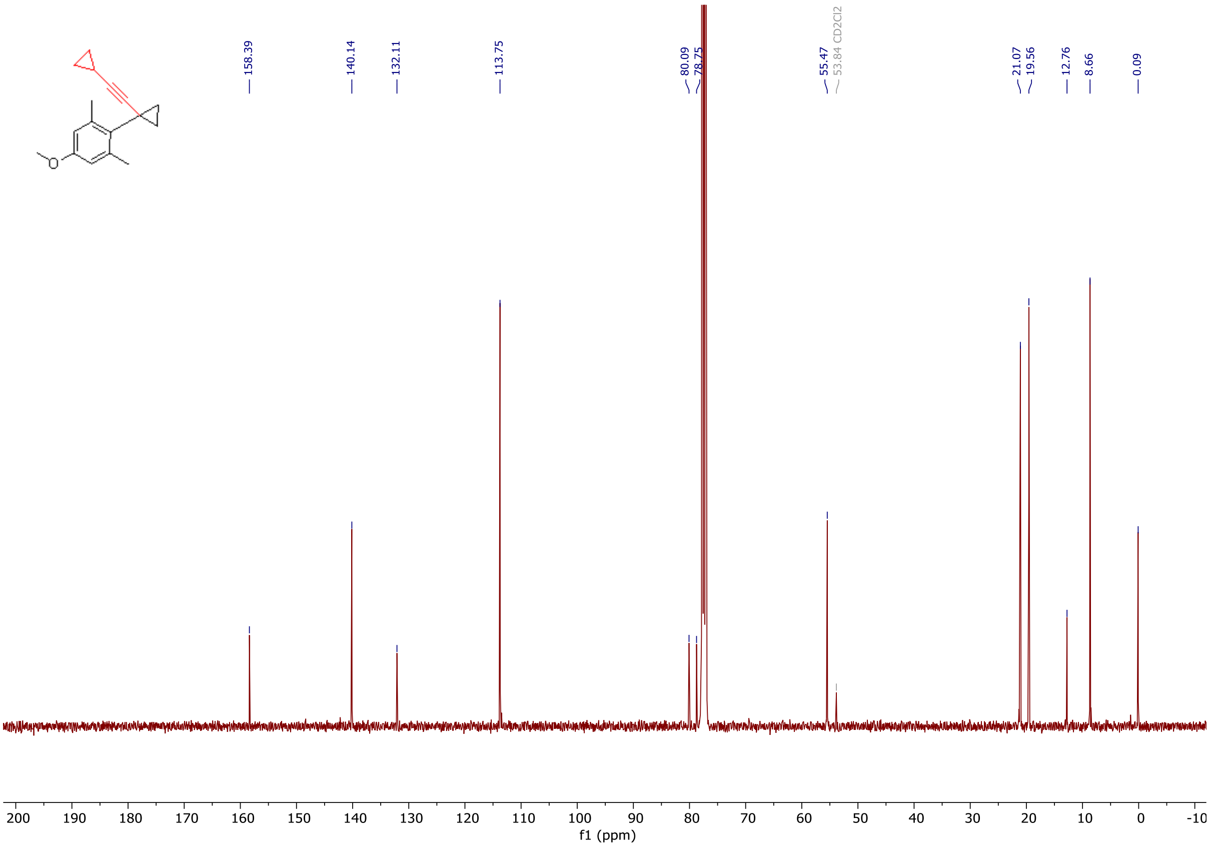

^1H NMR (400 MHz, $CDCl_3$) (4aa)

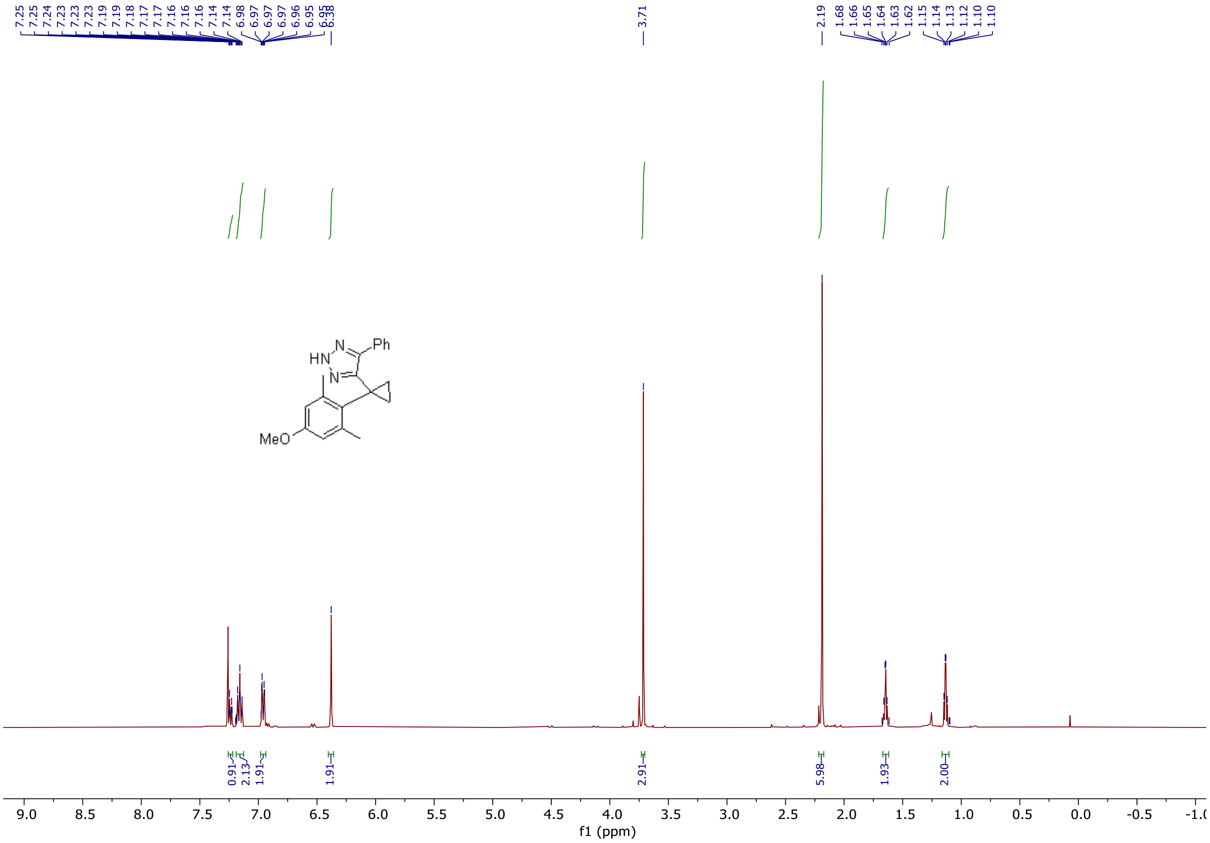

^{13}C NMR (101 MHz, $CDCl_3$) (4aa)

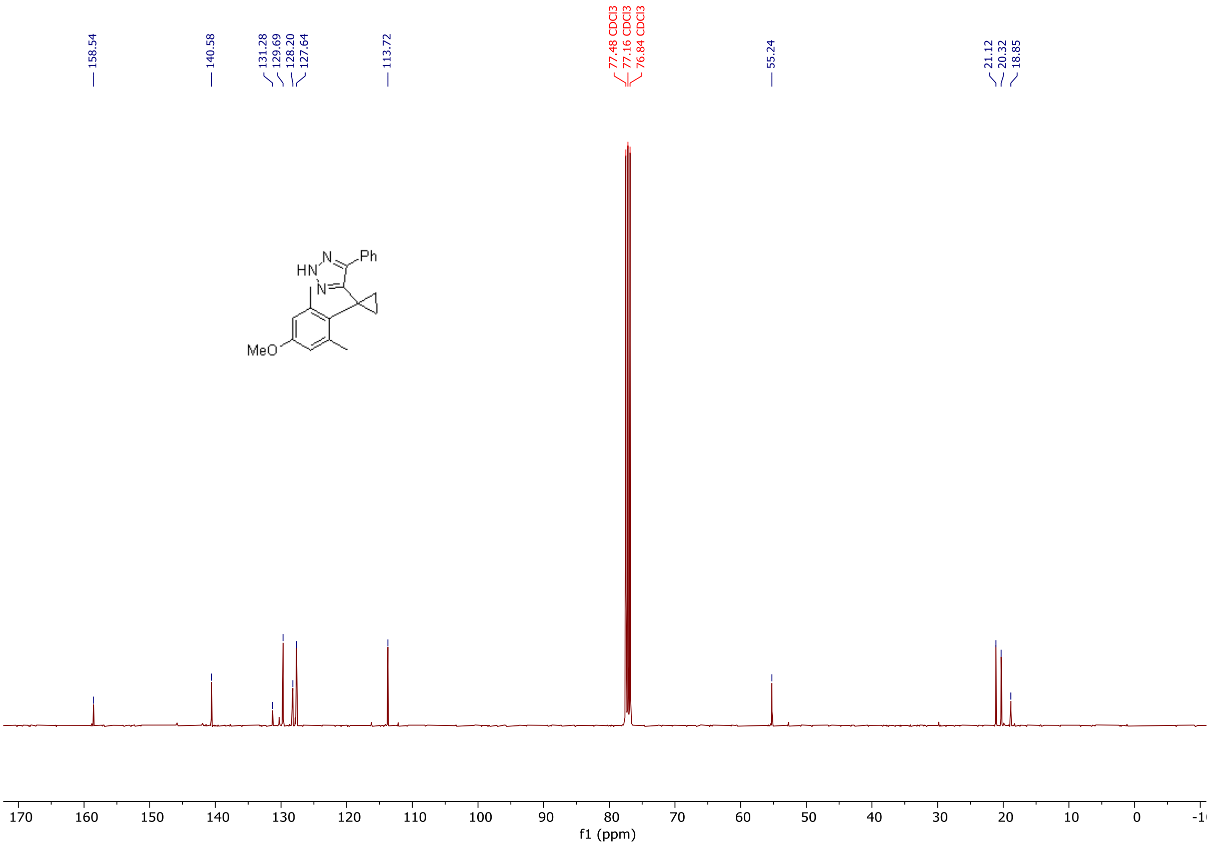

^{1}H NMR (400 MHz, $CDCl_3$) (4ab)

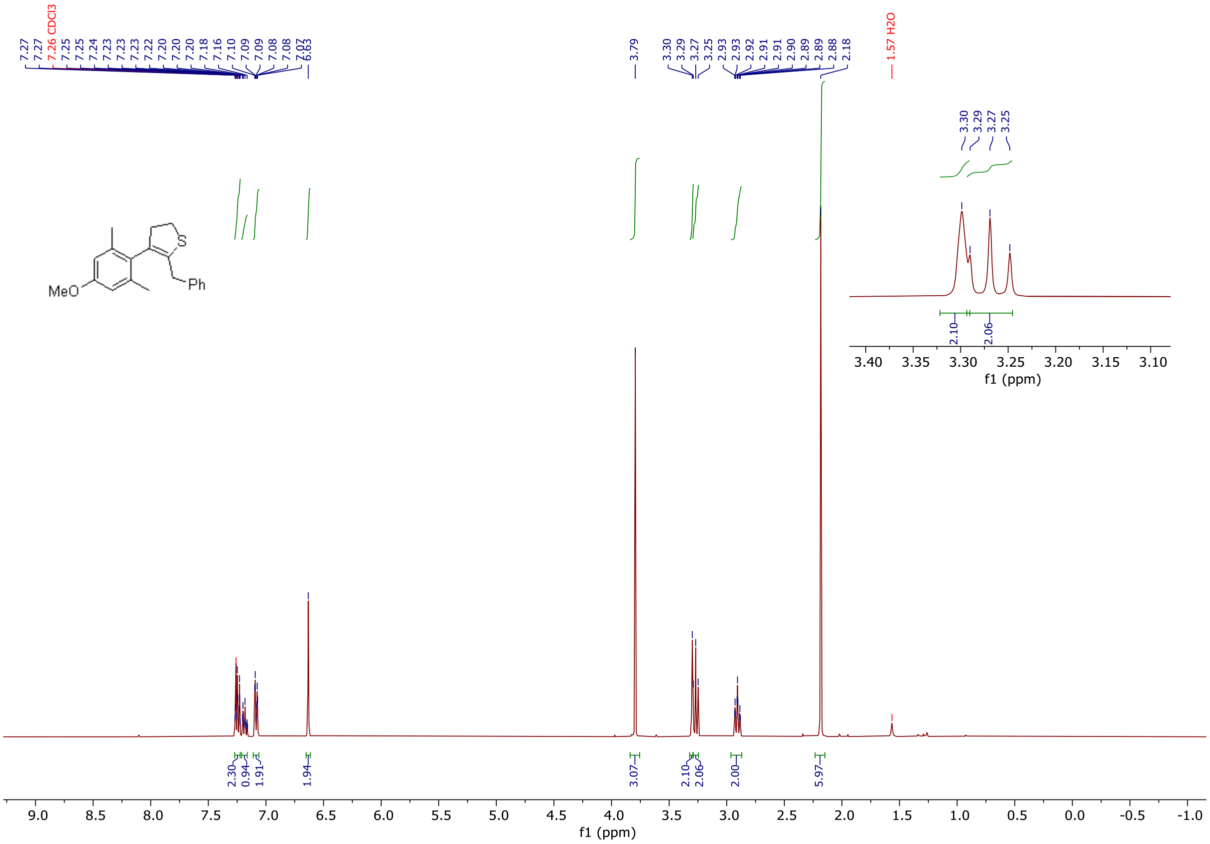

^{13}C NMR (101 MHz, $CDCl_3$) (4ab)

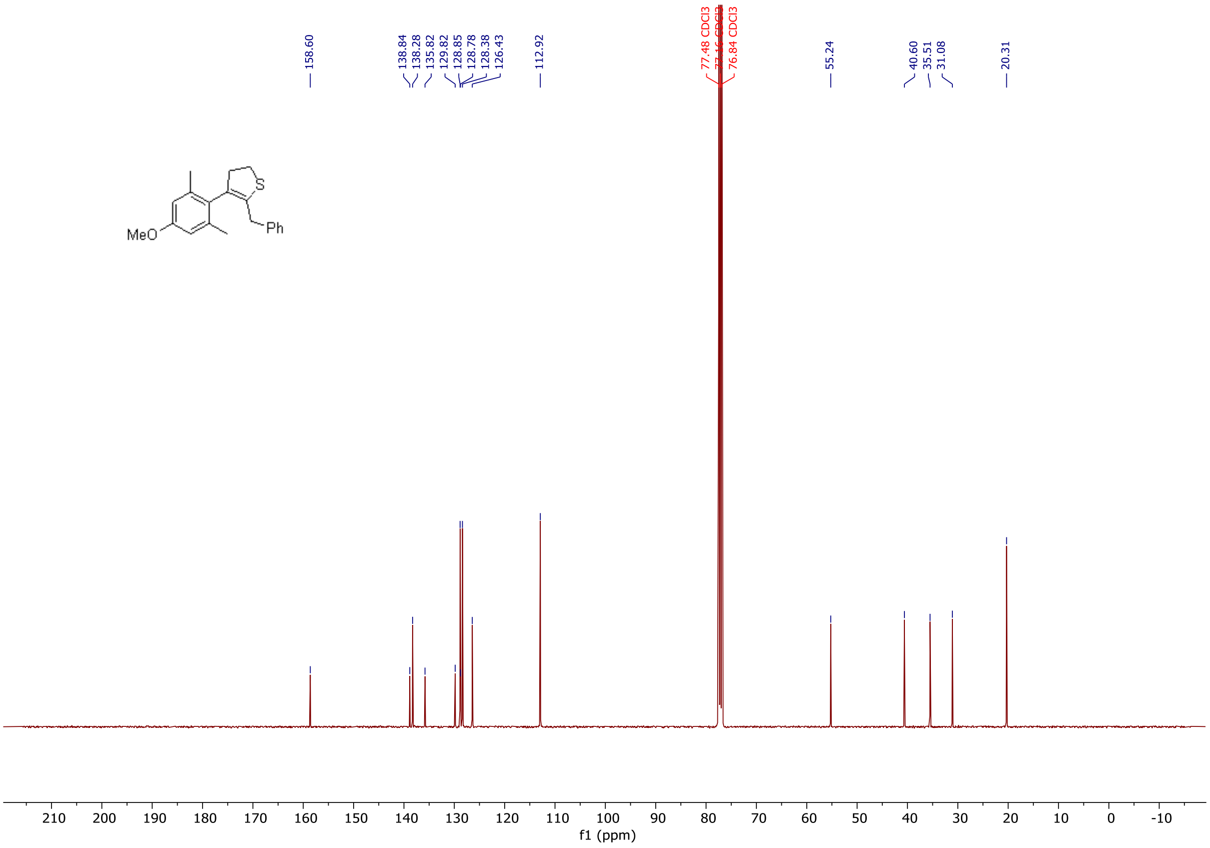

^{1}H NMR (400 MHz, $CDCl_3$) (4ac)

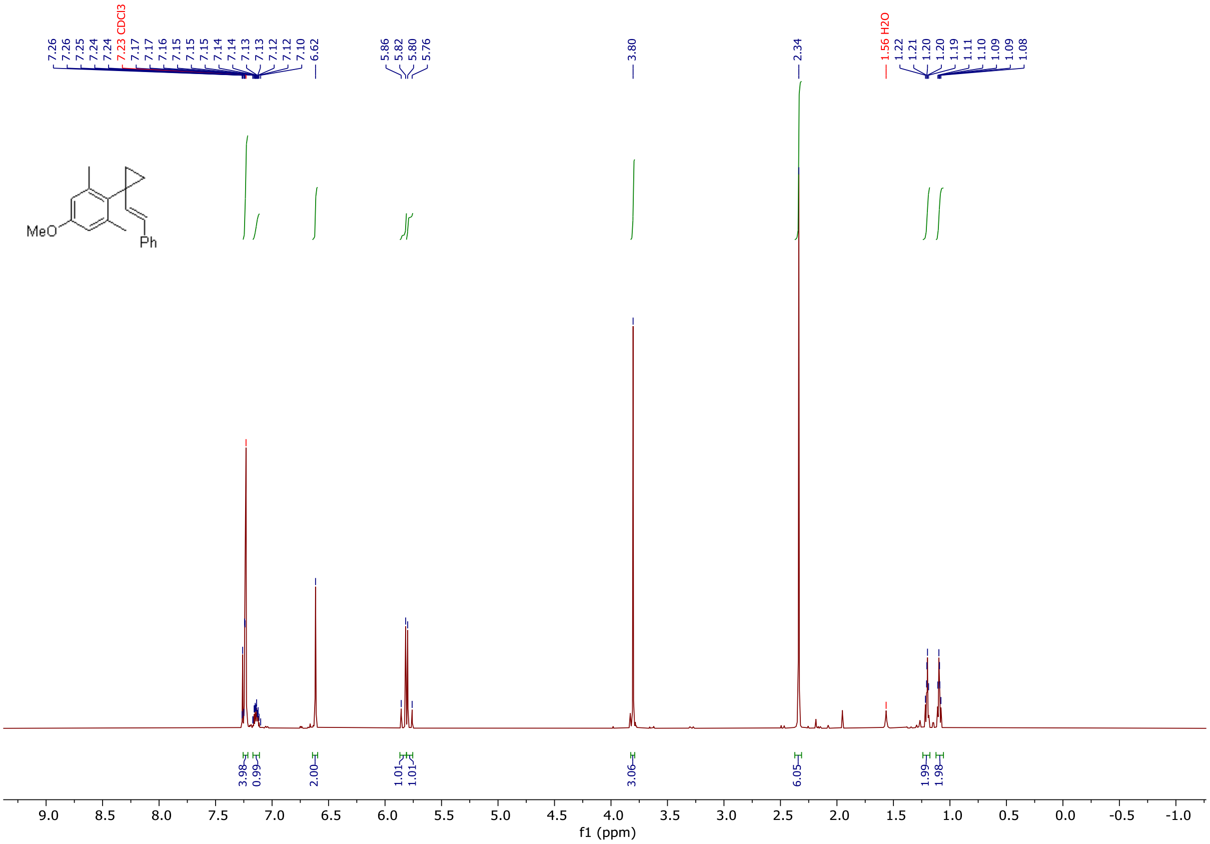

^{13}C NMR (101 MHz, $CDCl_3$) (4ac)

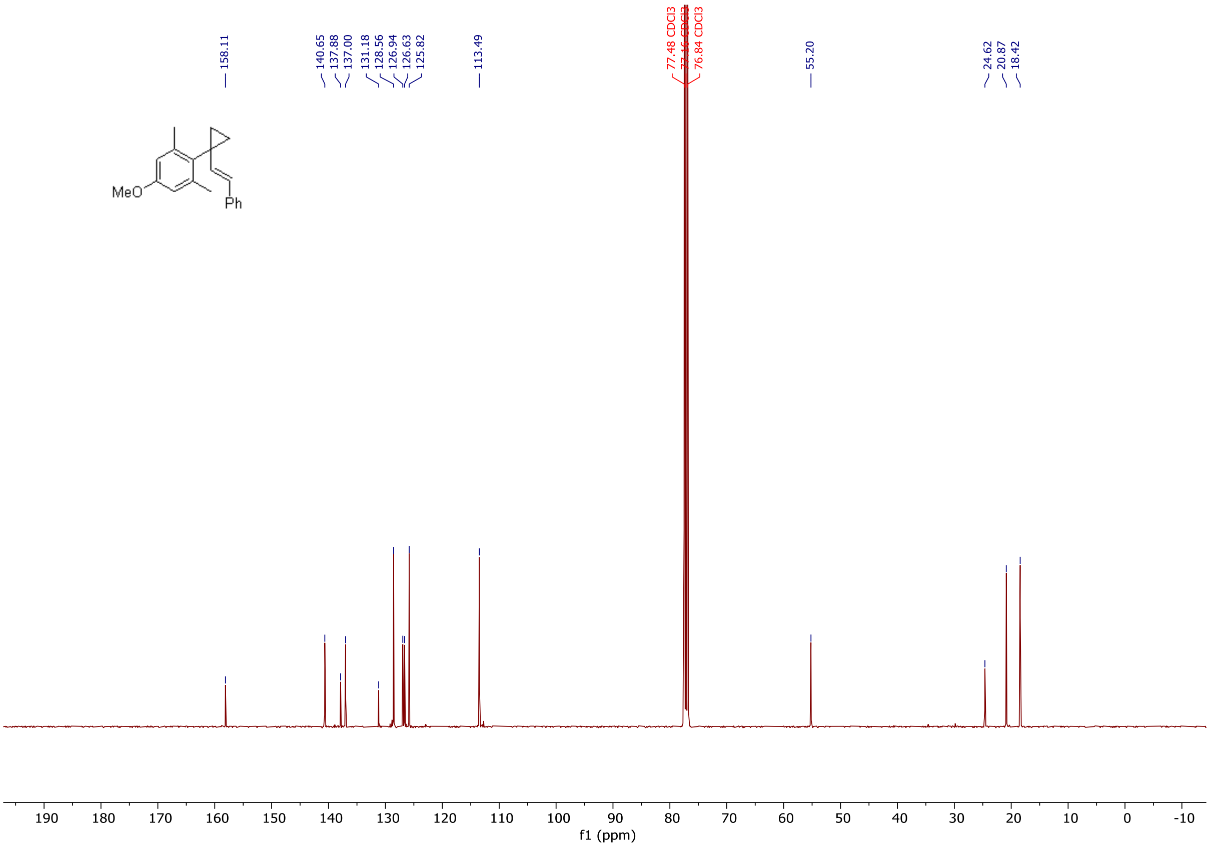

References

1 M. H. Gieuw, Z. Ke and Y. Yeung, *Angew. Chemie - Int. Ed.*, 2018, **57**, 3782–3786.

2 L. Ernst, T. Rieck and M. Soliven, *Can. J. Chem.*, 1999, **77**, 1697–1706.

3 R. M. Moslin, T. L. Andrew, S. E. Kooi and T. M. Swager, *J. Am. Chem. Soc.*, 2009, **131**, 20–21.

4 M. Sugawara and J. Yoshida, *J. Am. Chem. Soc.*, 1997, **119**, 11986–11987.

5 X. Qian, K. C. Russell, L. W. Boteju and V. J. Hruby, *Stereoselective Total Synthesis of Topographically Constrained Designer Amino Acids: 2', 6'-Dimethyl-~-methyltyrosines*, 1995, vol. 51.

6 R. Dey, S. Rajput and P. Banerjee, *Tetrahedron*, 2020, **76**, 131080.

7 H. Fan, H. Sun and X. Peng, *Chem. - A Eur. J.*, 2018, **24**, 7671–7682.

8 Y. Hua, Z. Liu, P. Xie, B. Ding, H. Cheng, X. Hong and Q. Zhou, *Angew. Chemie*, 2021, **133**, 12934–12938.

9 D. Petzold, P. Singh, F. Almqvist and B. König, *Angew. Chemie - Int. Ed.*, 2019, 8577–8580.

10 L. Ge, D. X. Wang, R. Xing, D. Ma, P. J. Walsh and C. Feng, *Nat. Commun.*, , DOI:10.1038/s41467-019-12403-2.

11 M.-M. Wang, T. V. T. Nguyen and J. Waser, *J. Am. Chem. Soc.*, 2021, **143**, 11969–11975.

12 F. Le Vaillant, T. Courant and J. Waser, *Angew. Chemie - Int. Ed.*, 2015, **54**, 11200–11204.

13 S. G. E. Amos, S. Nicolai and J. Waser, *Chem. Sci.*, 2020, **11**, 11274–11279.

14 S. G. E. Amos, D. Cavalli, F. Le Vaillant and J. Waser, *Angew. Chemie - Int. Ed.*, 2021, **60**, 23827–23834.

15 R. Y. Zhang, L. Y. Xi, L. Shi, X. Z. Zhang, S. Y. Chen and X. Q. Yu, *Org. Lett.*, 2016, **18**, 4024–4027.

16 D. Fernández González, J. P. Brand, R. Mondière and J. Waser, *Adv. Synth. Catal.*, 2013, **355**, 1631–1639.

17 F. Le Vaillant, M. Garreau, S. Nicolai, G. Gryn'Ova, C. Corminboeuf and J. Waser, *Chem. Sci.*, 2018, **9**, 5883–5889.

18 V. Matoušek, E. Pietrasiak, R. Schwenk and A. Togni, *J. Org. Chem.*, 2013, **78**, 6763–6768.

19 Z. Liu, S. Wu and Y. Chen, *ACS Catal.*, 2021, 10565–10573.

20 J. Ke, W. C. C. Lee, X. Wang, Y. Wang, X. Wen and X. P. Zhang, *J. Am. Chem. Soc.*, 2022, **144**, 2368–2378.

21 P. Pracht, F. Bohle, S. Grimme, *Phys. Chem. Chem. Phys.* 2020, **22**, 7169.

22 (a) J. Da Chai and M. Head-Gordon, *Phys. Chem. Chem. Phys.*, 2008, **10**, 6615–6620. (b) J. Da Chai and M. Head-Gordon, *J. Chem. Phys.*, , DOI:10.1063/1.2834918.

23 F. Weigend and R. Ahlrichs, *Phys. Chem. Chem. Phys.*, 2005, **7**, 3297–3305.

24 A. V. Marenich, C. J. Cramer and D. G. Truhlar, *J. Phys. Chem. B*, 2009, **113**, 6378–6396.

25 Gaussian 16, Revision A.03, Frisch, M. J.; Trucks, G. W.; Schlegel, H. B.; Scuseria, G. E.; Robb, M. A.; Cheeseman, J. R.; Scalmani, G.; Barone, V.; Petersson, G. A.; Nakatsuji, H.; Li, X.; Caricato, M.; Marenich, A. V.; Bloino, J.; Janesko, B. G.; Gomperts, R.; Mennucci, B.; Hratchian, H. P.; Ortiz, J. V.; Izmaylov, A. F.; Sonnenberg, J. L.; Williams-Young, D.; Ding, F.; Lipparini, F.; Egidi, F.; Goings, J.; Peng, B.; Petrone, A.; Henderson, T.; Ranasinghe, D.; Zakrzewski, V. G.; Gao, J.; Rega, N.; Zheng, G.; Liang, W.; Hada, M.; Ehara, M.; Toyota, K.; Fukuda, R.; Hasegawa, J.; Ishida, M.; Nakajima, T.; Honda, Y.; Kitao, O.; Nakai, H.; Vreven, T.; Throssell, K.; Montgomery, J. A., Jr.; Peralta, J. E.; Ogliaro, F.; Bearpark, M. J.; Heyd, J. J.; Brothers, E. N.; Kudin, K. N.; Staroverov, V. N.; Keith, T. A.; Kobayashi, R.; Normand, J.; Raghavachari, K.; Rendell, A. P.; Burant, J. C.; Iyengar, S. S.; Tomasi, J.; Cossi, M.; Millam, J. M.; Klene, M.; Adamo, C.; Cammi, R.; Ochterski, J. W.; Martin, R. L.; Morokuma, K.; Farkas, O.; Foresman, J. B.; Fox, D. J. Gaussian, Inc., Wallingford CT, 2016.

26 (a) C. Riplinger, B. Sandhoefer, A. Hansen and F. Neese, *J. Phys. Chem., 2013, **138**, 034106.* (b) C. Riplinger and F. Neese, *J. Chem. Phys., 2013, **139**, 134101*.

27 D. G. Liakos, M. Sparta, M. K. Kesharwani, J. M. L. Martin and F. Neese, *J. Chem. Theory Comput.*, 2015, **11**, 1525–1539.

28 F. Neese, F. Wennmohs, U. Becker and C. Riplinger, *J. Chem. Phys., 2020, **152**, 224108.*.

29 R. L. Martin, P. J. Hay and L. R. Pratt, *J. Phys. Chem. A*, 1998, **102**, 3565–3573.

30 (a) G. Luchini, J. V. Alegre-Requena, I. Funes-Ardoiz and R. S. Paton, *F1000Research*, 2020, **9**, 291. (b) G. Luchini, J. Alegre-Requena, I. Funes-Ardoiz and R. S. Paton, *GoodVibes v3.0.1, 2019.*.
